# Effects of El Niño and the Positive Indian Ocean Dipole (+IOD) on Health, Food Security, Economics, and Conflict in Low‐ and Middle‐Income Countries in the Indo‐Pacific: A Systematic Review

**DOI:** 10.1002/cl2.70038

**Published:** 2025-04-17

**Authors:** Andrea Floridi, María Daniela Anda‐León, Tomasz Kozakiewicz, Megha Bhattacharyya, Anilkrishna Thota, Peter Burt, Luca Tasciotti, Jan Selby, Zahra Premji, Shannon Shisler

**Affiliations:** ^1^ International Initiative for Impact Evaluation (3ie) London UK; ^2^ International Institute of Social Studies Erasmus University of Rotterdam Rotterdam the Netherlands; ^3^ School of International Development University of East Anglia Norwich UK; ^4^ Natural Resources Institute University of Greenwich London UK; ^5^ School of Accounting, Finance and Economics University of Greenwich London UK; ^6^ University of Leeds Leeds UK; ^7^ Independent Information Specialist Vancouver British Columbia Canada

## Abstract

Climate drivers such as the El Niño Southern Oscillation (ENSO) and the Indian Ocean Dipole (IOD) can impact multiple sectors globally. We are currently witnessing the effects of these teleconnections against the backdrop of a changing climate. This systematic review takes stock of the available evidence on compounding and cascading effects of El Niño and the Positive IOD on health, economic, migration, conflicts, and nutrition outcomes in low‐ and middle‐income countries from the Indo‐Pacific region. The review sheds light on how effects vary between and within the considered countries and explores potential sources of heterogeneity. The search of studies was carried out in January 2024 in 12 major databases/search engines and 14 institutional websites, using English keywords, and paired by forward and backward citation tracking of the included studies. The review's inclusion criteria encompassed quantitative studies as long as they provide an estimate of relationship between the climate driver and outcome, and qualitative studies that aim to infer causation such as realist evaluation or process tracing. The analysis used a combination of meta‐analysis with random‐effects models, median effects from correlational and regression studies, and narrative synthesis. We found that El Niño is likely to decrease agricultural production and productivity at the Indo‐Pacific level, although the analysed studies are highly diverse. The absence of evidence on the effects of the considered climate drivers on migration, conflict, food security and nutrition is an important evidence gap. We found limited evidence on the differential effects by El Niño's and +IOD's magnitude and no studies examining their combined impact or qualitative effectiveness studies. The high risk of bias detected across studies calls for more thorough attention to study design, conduct, and reporting in answering questions about effects. Despite remaining evidence gaps, this review highlights potential effects of El Niño and +IOD in the Indo‐Pacific and underscores the need for context‐specific policy responses to mitigate risks at local and regional levels. Caution is warranted in interpreting the overall findings given the generally high risk of bias of evidence.

**Abbreviations**
ENSO: El Niño Southern Oscillation
L&MICs: Low‐ and middle‐income countries
SMD: Standardised mean difference
SST: Sea surface temperature
+IOD: Positive Indian Ocean Dipole

## 1 Plain Language Summary

El Niño reduces agricultural production and productivity across Indo‐Pacific low‐ and middle‐income countries (LMICs), though effects are context specific.

### 1.1 The Review in Brief

El Niño reduces agricultural production and productivity in the Indo‐Pacific, though its effects vary between and within countries. There is no clear evidence that El Niño and +IOD affect disease incidence or market prices consistently across the Indo‐Pacific. There is no available evidence to assess effects of El Niño and +IOD on migration, conflict, and nutrition in the region.

### 1.2 What Is This Review About?

Climate drivers such as the El Niño Southern Oscillation (ENSO) and the Indian Ocean Dipole (IOD) can impact multiple sectors globally. El Niño is a climate driver that occurs in the tropical Pacific Ocean, and it is characterised by a seesaw in the sea‐level pressure between the western and eastern Pacific and with a weakening of the easterly trade winds over the tropical Pacific. The IOD is the difference in sea surface temperature (SST) between the western (near East Africa) and the eastern (near Indonesia) sides of the Indian Ocean. A +IOD is characterised by higher SSTs in the western Indian Ocean region. These climate drivers may affect economic outcomes, health, migration, conflict and violence, and food security in the Indo‐Pacific.

### 1.3 What Is the Aim of This Review?

This systematic review examines the effects of El Niño and +IOD on economic outcomes, health, migration, conflict and violence, food and nutrition security across LMICs in the Indo‐Pacific region. The review summarises evidence from 89 non‐randomised studies published after 1990.

### 1.4 What Are the Main Findings of This Review?

#### 1.4.1 What Studies Are Included?

In January 2024, we searched 12 academic databases and 14 institutional websites and found 10,113 records. Of these, 89 unique studies (and 12 linked publications) met our inclusion criteria. The included studies spanned the period from 1992 to 2023 and were mostly from India, Indonesia, the Philippines, Malaysia, and Bangladesh. Most included studies had a high risk of bias which means their results need to be interpreted with caution. The main concerns were related to study design, specifically the failure to isolate effects of El Niño from other factors such as changes in temperature or precipitation, technological innovations, or changes in population immunity.

#### 1.4.2 What Are the Effects of El Niño and +IOD on Health, Food Security, Socioeconomic Outcomes, Migration, Social Conflict, and Violence?

**Figure 1:**
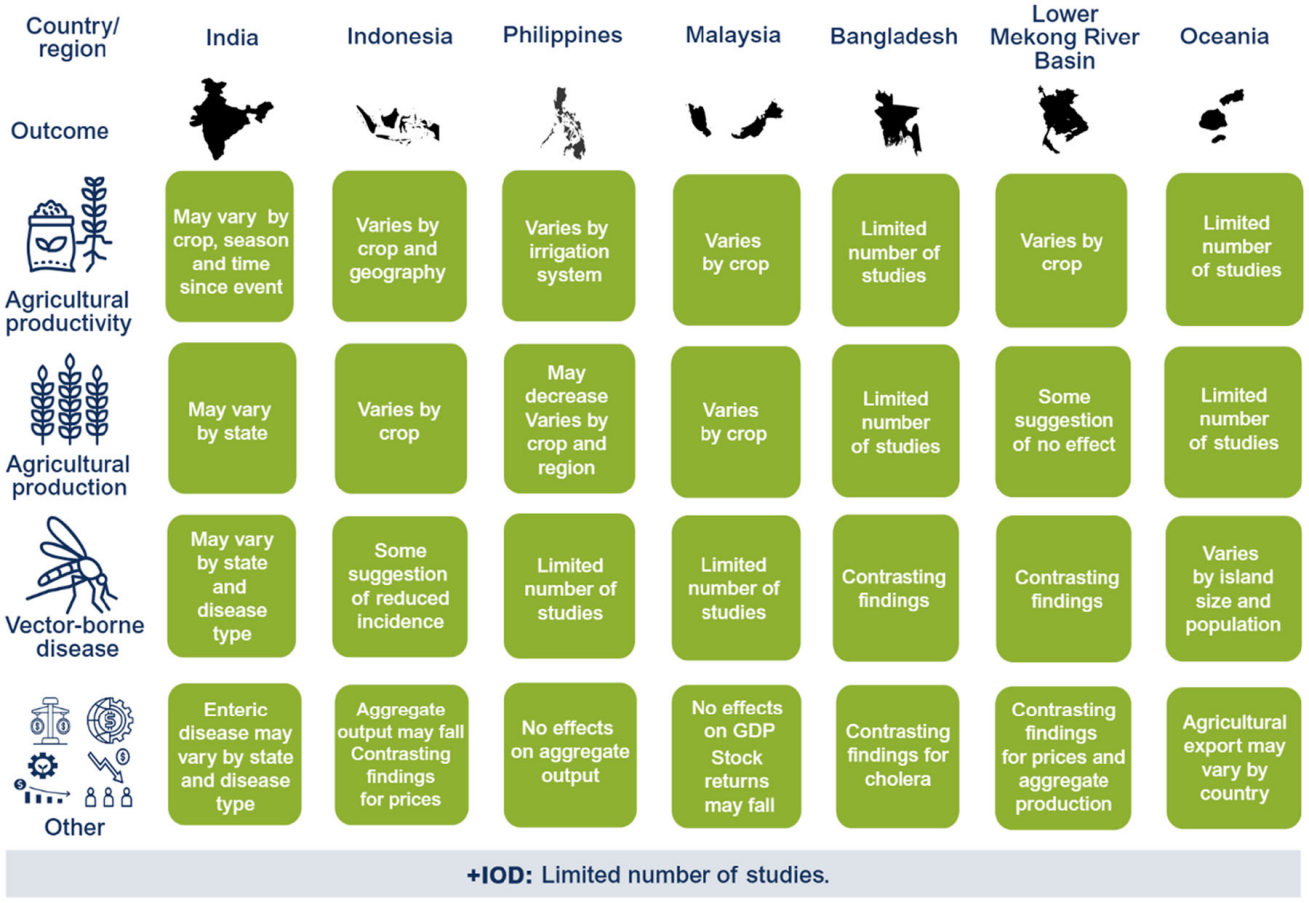
El Niño‐related findings from narrative synthesis. *Source:* 3ie (2025).

El Niño likely decreases agricultural production and productivity for LMICs in the Indo‐Pacific region but has no clear effect on prices or the incidence of vector‐borne diseases. Limited studies prevent conclusions on other outcomes such as aggregate production, trade, investment, household welfare, incidence of cholera, other enteric infections, direct injuries or fatalities, and respiratory infections. The findings greatly vary within and between countries. Very few studies assess the effects of +IOD in the Indo‐Pacific, which makes it difficult to draw any conclusions on the effects. No studies were found on the effects of El Niño and +IOD on migration, conflicts and violence, food security, and nutrition (Figure 1).

### 1.5 What Do the Findings of This Review Mean?

This review highlights potential effects of El Niño and +IOD in the Indo‐Pacific and underscores the need for context‐specific policy responses to mitigate risks at local and regional levels.

### 1.6 How Up‐to‐Date Is This Review?

The review authors searched for studies up to January 2024. This Campbell Systematic Review was published in March 2025.

## 2 Background

The scope of this systematic review is to gather, assess, and synthesise evidence published after 1990 on the effects of El Niño and the positive Indian Ocean Dipole (+IOD) for LMICs in the Indo‐Pacific region. The review aims to *synthesise evidence on their direct and indirect effects on health, food security, socioeconomic outcomes, migration, social conflict, and domestic violence*.

The review addressed the following questions:

1. What does the available evidence indicate about the direction and magnitude of the effects of El Niño and +IOD across Indo‐Pacific LMICs over the past 34 years?
2. How do effects vary by type of outcome (i.e., health, food security, economics, migration, and conflict outcomes)?
3. How do effects vary with respect to a stronger or weaker El Niño and +IOD and how do they vary between climate drivers (e.g., Canonical El Niño or El Niño Modoki^1^)?
4. What are the main factors of variation accounting for the diversity of findings (e.g., geographical area and type of measurement)?
5. What is the risk of bias of the available evidence?
6. What are the evidence gaps and how can future research address these?

Section 2 provides background information and presents a theory of change of the effects of El Niño and +IOD in the Indo‐Pacific. Section 3 elucidates the approach and methodology adopted in the report. Section 4 presents the findings from the analysis, followed by a discussion in Section 5. Section 6 includes implications for policymaking and insights for future research.

### 2.1 ENSO and the IOD

Drivers of climate variability such as the ENSO and the IOD affect regions around the globe through various pathways known as teleconnections (Domeisen et al. 2019). Teleconnections can be broadly defined as low‐frequency variability in the atmosphere and oceans that denote significant correlations of climatological variables at geographically distant points (Goodrich 2016).

**Figure 2:**
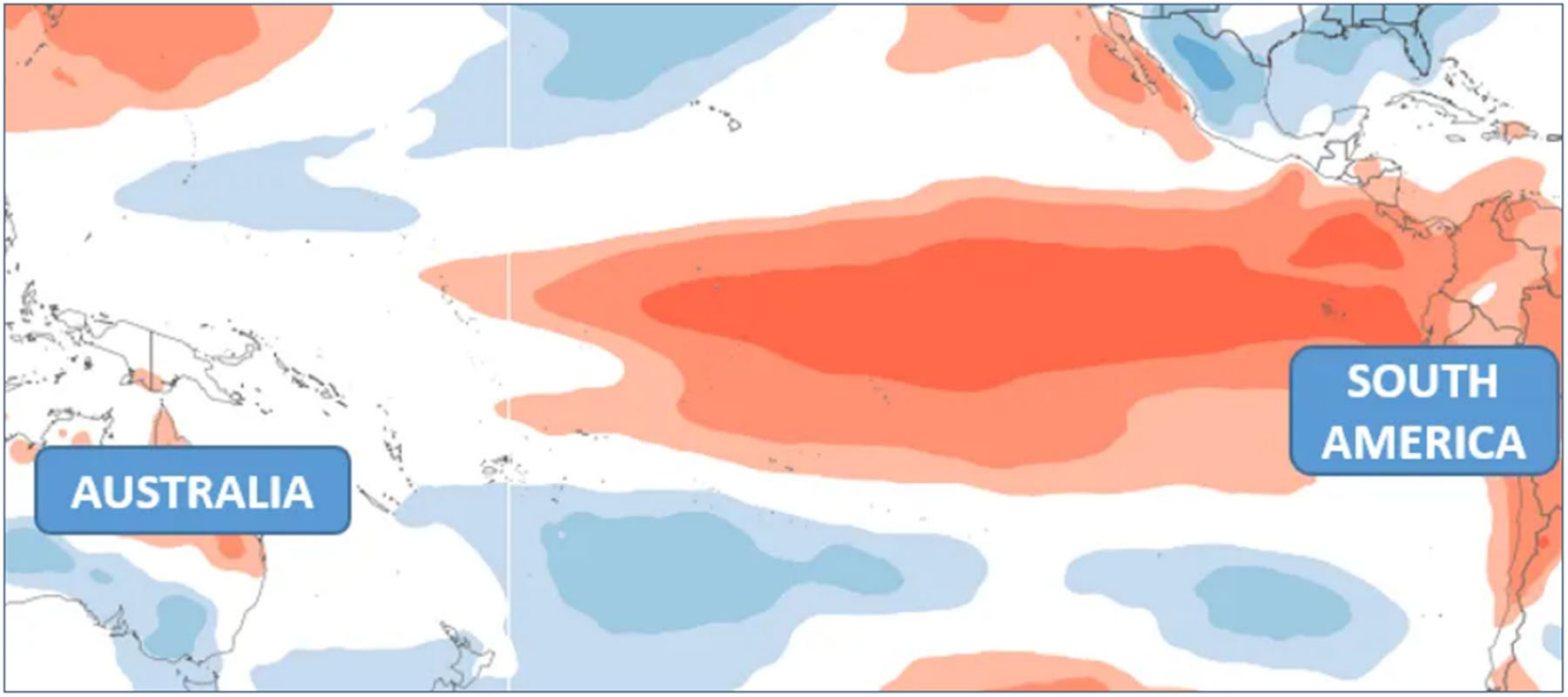
Positive sea surface temperature anomalies in the equatorial Pacific (El Niño). *Source:* World Climate Service (2021).

ENSO comprises coupled oscillations in both the ocean and the atmosphere (Southern Oscillation). An El Niño is a large‐scale oceanic warming event that occurs in the tropical Pacific Ocean, whilst the Southern Oscillation is characterised by a seesaw in tropical sea‐level pressure between the western and eastern Pacific, associated with a weakening and strengthening of the easterly trade winds over the tropical Pacific (Wang et al. 2017) (Figure 2).

**Figure 3:**
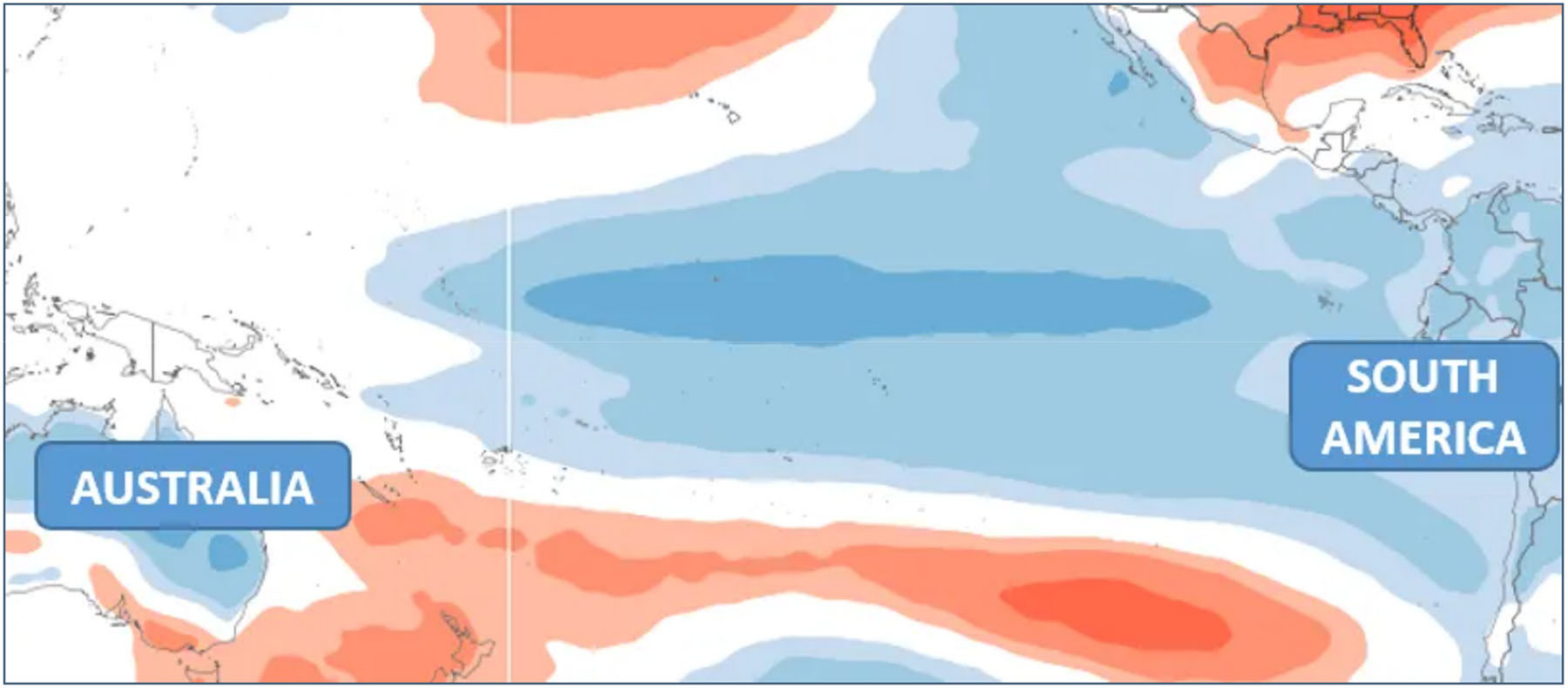
Negative sea surface temperature anomalies in the equatorial Pacific (La Niña). *Source:* World Climate Service (2021).

During an El Niño, trade winds weaken and can reverse direction, hindering the upwelling of nutrient‐rich cold water along the South American coasts and preventing warmer water from reaching Oceania and East Asia (Rony et al. 2024). The concomitant Southern Oscillation lowers the air pressure over the South American coast (Bjerknes 1969), leading to more abundant rainfall and increased flooding. Higher air pressure over the East Asian and Oceanian regions, on the other hand, leads to less rainfall and an increase in drought (Figure 3).

An opposite phenomenon to El Niño is La Niña, a strengthening of the tropical trade winds that force warm surface water towards the western Pacific. This results in cooling of the central and eastern tropical Pacific Ocean, with greater rainfall over the maritime continent and parts of South East Asia, but less rainfall over the central and eastern tropical Pacific Ocean.

The meteorological effects of these drivers generally hold true, although exceptions do occur. For example, a variation of El Niño is known as the El Niño Modoki (Ashok et al. 2007), which occurs when there is upwelling of colder water on both the west and east coasts of the Pacific Ocean. This results in drier weather with reduced rainfall and increased risk of drought in the western Pacific Ocean and the western Indian Ocean (Marathe and Karumuri 2021; Salimun et al. 2014).

**Figure 4:**
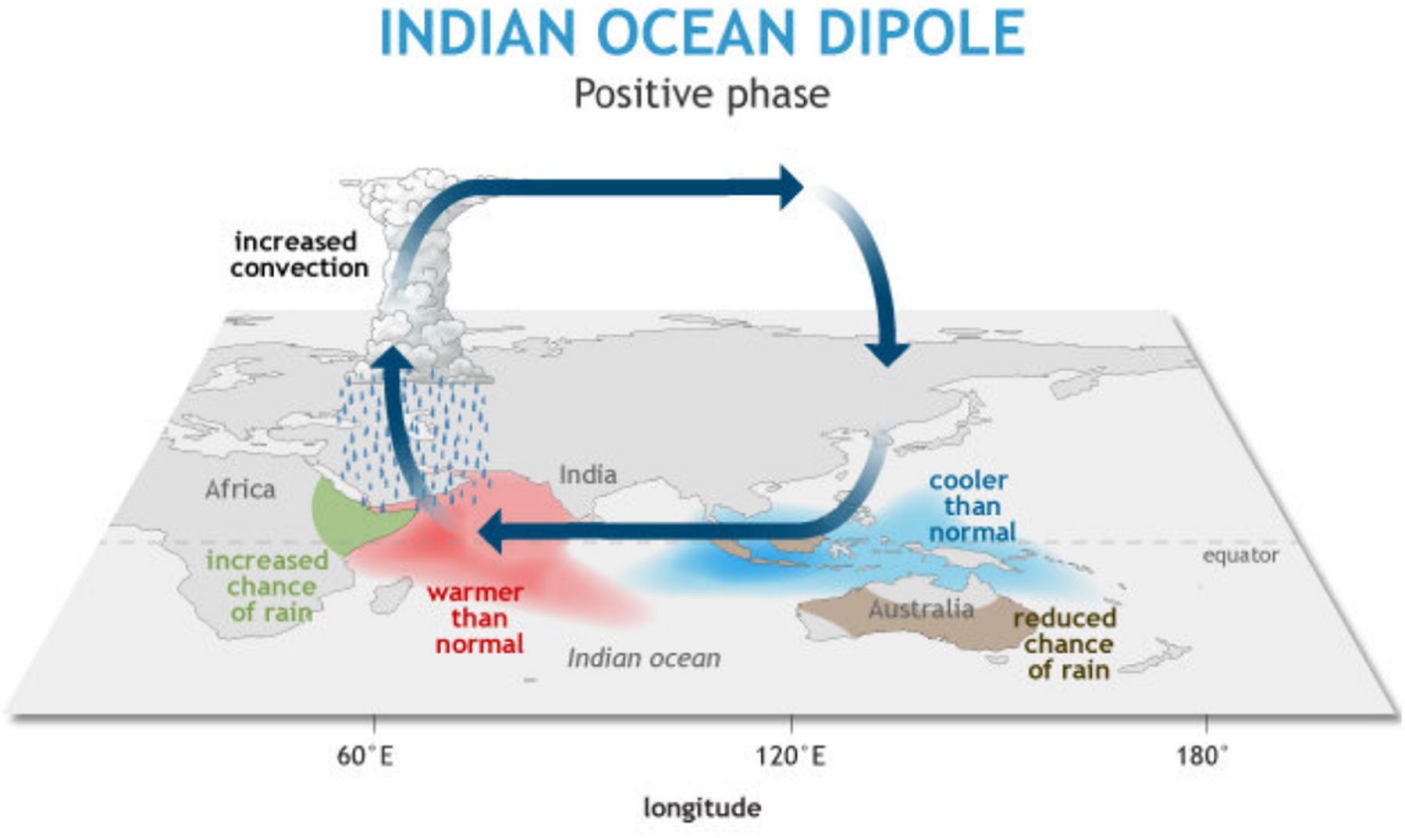
Sea surface temperature anomalies during a positive Indian Ocean Dipole event. *Source:* NOAA (2020).

The IOD is the difference in SST between the western (near East Africa) and the eastern (near Indonesia) sides of the Indian Ocean. A +IOD is characterised by higher SSTs in the western Indian Ocean region, whilst a negative IOD is driven by lower SSTs in the Eastern Indian Ocean (Saji and Yamagata 2003; Ashok et al. 2003) (Figure 4).

A +IOD brings changes to weather patterns, including warmer and more humid conditions, resulting in abundant monsoons and increased risk of flooding across the western Indian subcontinent. Although considered a distinct phenomenon (Ashok et al. 2003), the IOD is affected by ENSO, and the two often occur in temporal proximity (Stuecker et al. 2017).

The effects of ENSO and IOD may materialise as changes in the seasonal sea and land surface temperatures cycle. These effects are fuelled by global warming, leading to more frequent and severe climate disasters compared to the earlier decades of the twentieth century (Saji and Yamagata 2003; Cai et al. 2009; Cai et al. 2021; Yeh et al. 2009). Cai et al. (2009) found a trend of increased frequency, with two or three consecutive +IOD events between 1950 and 1999. This pattern may prolong droughts in regions like South East Asia, where rainfall diminishes during +IOD episodes. In India, the increased frequency of Central Pacific El Niño events after 1990, driven by global warming, could lead to more severe drought conditions (Yeh et al. 2009).

### 2.2 Effects of El Niño and +IOD: Outlining a Theory of Change

**Figure 5:**
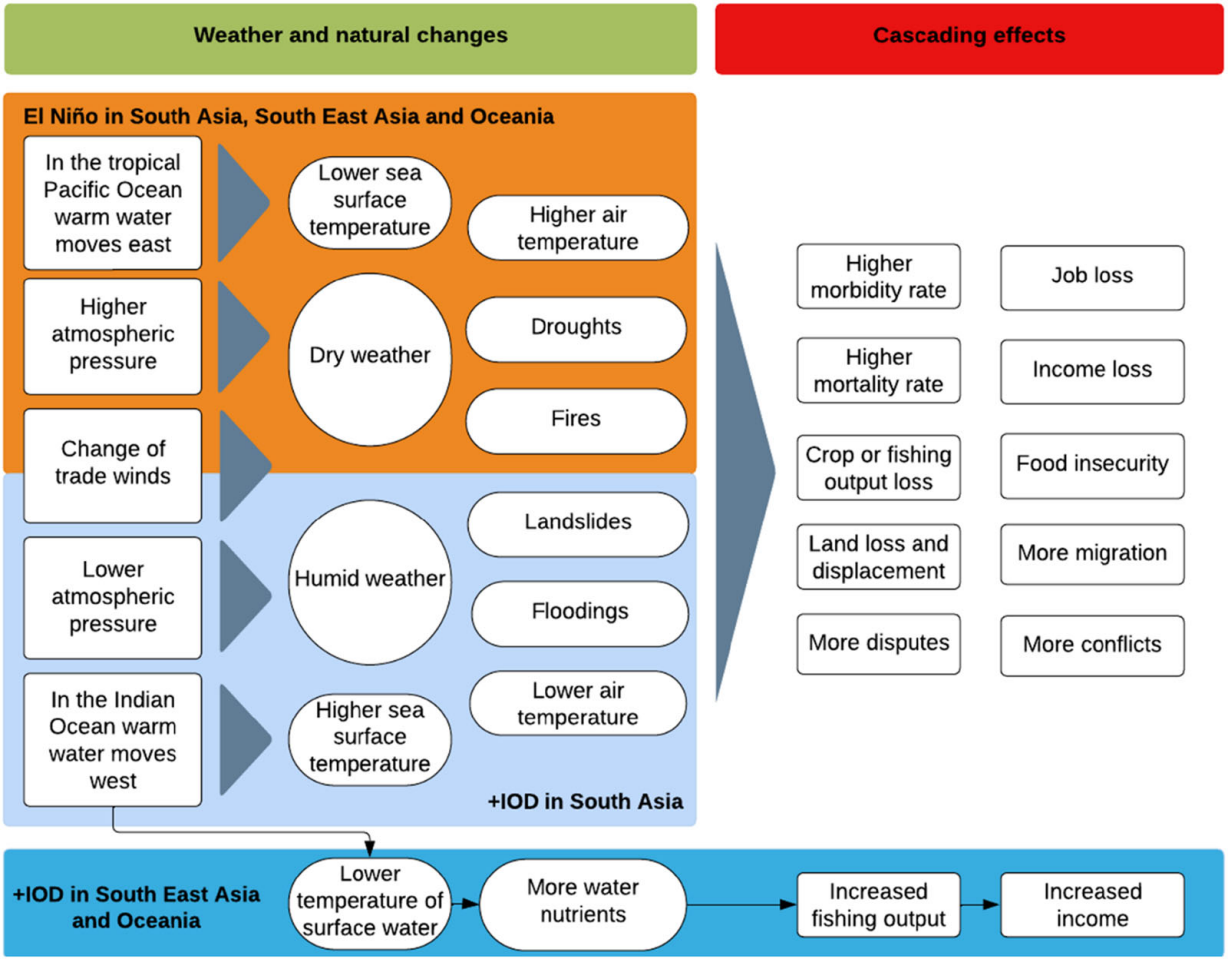
Theory of change for the cascading effects of El Niño and +IOD in South Asia, South East Asia, and Oceania. *Note:* The two climate drivers of interest are colour‐coded: orange for El Niño and blue for +IOD. The diagram is an oversimplified representation, which does not reflect all geographies within the Indo‐Pacific region, the interactions between climate drivers, or other factors. *Source:* 3ie (2025).

These climate drivers could have cascading effects on multiple sectors such as crop loss, food insecurity, infectious diseases, and cholera pandemics due to floods, respiratory and cardiovascular disease from hotter weather, collective and idiosyncratic economic shocks, displacement and land loss, migration, and conflicts (Rosenzweig and Hillel 2008; Alfani et al. 2019; Lal and Singh 2023; Mukherjee et al. 2023; Hendrix et al. 2022). Figure 5 illustrates a simplified theory of change of El Niño and +IOD in South Asia, South East Asia, and Oceania. There is limited evidence for all the cascading effects. Therefore, Figure 5 (and this subsection) illustrates the expected cascading effects based on a scoping exercise of existing literature, rather than on the studies included in this review and the effects detected by our analysis.

During El Niño events, lower SST, combined with higher atmospheric pressure in the Western Pacific and South East Asian countries, leads to drier weather and less cloud cover and rainfall, which in turn can lead to higher temperatures. In these weather conditions, droughts and fires are more likely to occur, with disruptive effects on yields (Mukherjee et al. 2023). Such crop loss undermines income and food security (Rosenzweig and Hillel 2008; Lal and Singh 2023), possibly affecting unemployment and poverty levels (Alfani et al. 2019). The simultaneous warming of the air and the sea surface may also have negative effects on fish catches and aquaculture in the East and South China Seas, with cascading effects on decreased income and increased conflict^2^ (Hendrix et al. 2022).

In this context, adverse effects on health are likely to intensify after El Niño onset. For example, drier and hotter conditions can increase respiratory and cardiovascular disease, especially amongst the most vulnerable areas with alterations in air quality, and individuals with pre‐existing lung conditions (Rony et al. 2024). Similarly, a loss of income and employment may have undesirable effects on nutrition (Soekirman 2001).

A +IOD can lead to lower air pressure and increased SST in the western Indian Ocean. This generates more humid weather, causing more intense Indian monsoon rainfalls (Ashok et al. 2003). Excessive rainfall increases the risks of flooding, which in turn may facilitate the spreading of malaria, cholera pandemics, and other diseases. For instance, Hashizume et al. (2011) found a positive association between the incidence of cholera in Dhaka (Bangladesh) in the first 3 months of +IOD conditions between 1993 and 2007. However, the influence of a +IOD changes with the seasons, and through interaction with ENSO, and may have different effects outside of the Indian subcontinent (e.g., it might exacerbate the effects of El Niño by further decreasing rainfall in South East Asia).

Increased moisture caused by excessive rainfall weakens soil stability, making slopes more susceptible to landslides (Onyutha et al. 2020). The displacement of soil and obstruction of watercourses, coupled with the already intense rainfall, results in a higher risk of flooding and waterborne diseases such as cholera (Levy et al. 2016). The consequences of flooding and landslides go beyond health effects, however, as they can destroy entire neighbourhoods, villages, and towns and lead to the displacement of people. For instance, Zhou et al. (2021) showed that the +IOD of 2019 was amongst the main causes of the 2020 Yangtze flooding in China, which led to the displacement of millions of people in the middle of the COVID‐19 pandemic emergency.

A +IOD is also considered amongst the key factors affecting harvests and yields (particularly of rice), with negative consequences on income and food security (Ghose et al. 2021). Despite causing damage to harvest and yields, a +IOD's resultant lower SST off the eastern Indian Ocean coasts increases its nutrient levels through increased upwelling (Sambah et al. 2023). This attracts more fish, resulting in increased fish capture (Setyohadi et al. 2021) and may lead to improved incomes in the fishery sector.

The interaction between +IOD and El Niño in the Indo‐Pacific depends on the region. An increase in precipitation of Indian monsoon rains associated with +IOD may mitigate the adverse effects of El Niño on droughts in the Indian subcontinent. In southern South East Asia, it may compound El Niño effects due to changes in the SST surrounding the region, resulting in drier weather and drought during the summer boreal season of June to August (Amirudin et al. 2020).

### 2.3 Why It Is Important to Do This Review

[The] El Niño phenomenon has posed a serious challenge to the hard‐fought development gains of developing countries (…), including by increasing the spread of diseases and the number of people displaced, affecting food security and infrastructure and hampering the ability of those countries and peoples to achieve sustainable development.

(United Nations General Assembly UNGA 2017, 2)

The predictability of seasonal‐scale climate drivers such as ENSO and IOD, and their associated weather variations, is increasingly important. The Intergovernmental Panel on Climate Change (Masson‐Delmotte et al. 2021) forecasts that the occurrence of intense ENSO events is expected to rise over the next century. For instance, 2023 has been documented as the warmest year on record globally (NOAA 2023), possibly exacerbated by the concurrent strong El Niño (Li et al. 2023). In this context, more and better evidence of direct and indirect effects at the local, national, and regional levels is required. In our scoping of the literature, we found literature reviews examining the effects of ENSO on health outcomes (Kovats 2000; Kovats et al. 2003; Lam et al. 2019) and one systematic review of ENSO on diarrhoeal disease (Demissie 2017). The latter included 30 studies, but only a handful from Indo‐Pacific countries.

Understanding compounding and cascading socioeconomic effects from weather, seasonal climate variability, and associated hazards holds the potential to inform policy and enable actionable outcomes to minimise or optimise effects.

## 3 Methods

### 3.1 Criteria for Considering Studies for This Review

We included studies from both academic and grey literature sources that employed quantitative and qualitative methods to examine the socioeconomic effects of El Niño and/or +IOD on selected LMICs in the Indo‐Pacific region (Table 1).

To be eligible for inclusion, the studies needed to be published in 1990 or later, and while the search was conducted in English, we did not exclude studies published in other languages. No restriction was applied based on publication status.

**Table 1:** List of inclusion criteria.

Criterion	Included	Excluded
Population	Indian subcontinent and Indian Ocean (Bangladesh, Bhutan, India, Maldives, Nepal, Sri Lanka), South East Asia (Cambodia, Indonesia, Laos, Malaysia, Myanmar, the Philippines, Thailand, Timor Leste, Vietnam) and Oceania (Fiji, Kiribati, Marshall Islands, Micronesia, Nauru, Palau, Papua New Guinea, Samoa, Solomon Islands, Tonga, Tuvalu, Vanuatu)	Countries outside the Indo‐Pacific region High‐income countries in the Indo‐Pacific (New Zealand, Australia, Brunei, Singapore, etc.) Certain LMICs in Indo‐Pacific (China, Pakistan)Broad geographic regions that include countries above (where no separate estimate for included countries is provided)
Climate driver	El Niño, +IOD, El Niño Modoki	La Niña, Arctic Oscillation, Madden‐Julian Oscillation, North Atlantic Oscillation
Comparator	Any	None
Outcomes	Health (direct injuries or fatalities, disruption of health services, morbidity and mortality including mental health and heat stress) Conflict and violence (local, trans‐border, domestic abuse/intimate partner violence, civic unrest and disputes) Economic (income, production,^1^ productivity,^2^ employment, trade, consumption, prices, investments, supply chains, tourism, inequalities, IT, empowerment) Migration (internal, cross‐border, transhumance, economic/labour) Food and nutrition security, malnutrition	Air temperatures, precipitation, biological processes such as chlorophyll‐a, algal proliferation, fish/shellfish poisoning (unless its health effects for humans are examined) and other non‐socioeconomic outcomes
Study design	Quasi‐experimental methods (instrumental variables, fixed effects, statistical matching, synthetic control, panel methods) Regression analysis (time series models, multiple regression, Bayesian) Correlational analysis (Pearson's, partial) Qualitative causal inference methods appropriate for the research question (realist evaluation, general elimination methodology, process tracing or contribution analysis)	Spatial models with no quantitative estimates, and descriptive studies providing estimates of anomalies using methods such as moving averages but not reporting an association estimate between the climate driver and the outcome Qualitative methods that do not aim to infer causation and do not provide a theory of change

^1^ Production outcomes include any measure of the disaggregated economic output such as fish captures, total volume of (agricultural) production or outputs, and (share of) land/area cultivated or harvested.

^2^ Productivity outcomes include measures of agricultural productivity (yields) or business productivity (fish catch per unit effort).

### 3.2 Search Methods for Identification of Studies

#### 3.2.1 Electronic Searches

Systematic search strategies were developed for each database listed in Table 2. Each search incorporated three concepts: (1) El Niño/+IOD; (2) Indo‐Pacific countries/regions; and (3) study design/analysis types. The Indo‐Pacific countries and study design concepts borrowed relevant terms from existing search strategies used by 3ie to search for LMICs and impact evaluations.

**Table 2:** List of electronic databases and sources searched.

Database sources (platform)	
Scopus (Elsevier)	CAB Abstracts (Ovid)
Agricola (Ovid)	Econlit (Ovid)
MEDLINE (Ovid)	Academic Search Complete (EBSCOhost)
EMBASE (Ovid)	ProQuest Dissertations & Theses Global (Web of Science)
Global Health (Ovid)	BIOSIS Citation Index (Web of Science)
Web of Science Core Collection (SSCI, SCI‐EXPANDED, CPCI‐S, CPCI‐SSH and ESCI)	EBSCO Discovery Service (GreenFILE, Science Direct, AGRIS, AGRIS ODS, RePEc, World Bank e‐Library)

The search concepts incorporated free‐text terms and controlled vocabulary, when available. The keywords were enhanced using source‐specific syntax/operators and combined using Boolean operators (AND/OR). The searches were developed and iteratively tested against a set of known articles derived from pearl‐harvesting techniques (Sandieson et al. 2010).

The final database searches were run on January 2024, exported in RIS format, and deduplicated initially on the Covidence software platform. The complete search strategies, search results table, search narrative, and details of the known article testing can be found in Online Appendix S1A.

#### 3.2.2 Searching Other Resources

We identified relevant institutional websites and repositories, based on previous reviews on the topic and suggestions from experts from our advisory group. For those sources that provided the highest number of hits (e.g., Google Scholar), multiple queries were executed. The search process involved one round of both backward and forward citation tracking for included studies and relevant reviews using Web of Science, Scopus, and automated software. For more details, refer to the protocol (Floridi et al. 2024).

### 3.3 Data Collection and Analysis

#### 3.3.1 Selection of Studies

We followed a two‐stage process as detailed in the protocol (Floridi et al. 2024). Trained reviewers screened titles and abstracts against inclusion criteria assisted by a machine‐learning classifier tool. For the included abstracts, two independent reviewers screened the full‐text records and reconciled any disagreements on the decision to include or exclude a study through consensus or with the input of a third reviewer if necessary. The included studies are highlighted with an asterisk in the reference list and their summaries are presented in Online Appendix S1B. Examples of excluded records can be found in Online Appendix S1C.

#### 3.3.2 Data Extraction and Management

We extracted descriptive, methodological, quantitative, and qualitative data from each included study using a standardised data extraction codebook in MS Excel, documented in Floridi et al. (2024). After an initial training round, two reviewers independently double‐coded quantitative data for outcomes analysis and risk‐of‐bias assessments. Any disagreements were resolved through discussion with a third reviewer. Descriptive, methodological, and qualitative data were extracted by one team member, with a second reviewer conducting quality assessments.

For regression studies, except for incidence rate ratios, we calculated effect sizes and converted them to standardised mean differences (SMD) (as described in detail in Online Appendix S1D). For correlation studies, we used the coefficients as reported by the authors since these are unit‐less and comparable measures of the strength of the association between the climate driver and the outcome of interest.

We reversed the sign of the effect estimates for certain outcomes where an increase is considered unfavourable. For example, for disease incidence rates, we reversed the sign such that a positive number would indicate a reduced incidence rate of a disease. Similarly, for price levels, we reversed the sign such that a positive number would indicate lower prices, for example, decreased staple food prices for consumers. This reversal ensured that a positive association indicates something beneficial. It allows for easier interpretation, especially when comparing estimates from different sources such as meta‐analyses and correlation coefficients.

#### 3.3.3 Critical Appraisal

To assess the confidence we have in the findings of included studies, we applied an adapted version of the 3ie risk‐of‐bias tool for quasi‐experimental designs (Waddington et al. 2014) (Online Appendix S1E). The tool assesses three domains: study design, data quality/appropriateness, and selective reporting. Our decision to adapt the tool was based on the fact that the literature in this field rarely uses quasi‐experimental designs and certain aspects of bias assessment in traditional tools, such as attrition (when people drop out of a study or project over time) or performance bias (when there are differences in how participants in different groups are treated or behave during a study), are not applicable to this field. Two reviewers independently assessed the studies and any differences in assessment were reconciled through discussion. We assigned an overall risk‐of‐bias score to each study based on the score for each criterion as outlined in the protocol (Floridi et al. 2024).

#### 3.3.4 Data Synthesis

Considering the variation of included study methods, the analysis followed three steps.

In the first step, we conducted a random‐effects meta‐analysis using robust variance estimation techniques for a limited number of climate drivers and outcome combinations where sufficient regression studies were available (Tanner‐Smith et al. 2016). For the vector‐borne diseases outcome category, we excluded two outlier coefficients from the meta‐analysis, as they use Bayesian modelling methods (Sharmin et al. 2016; Yip et al. 2022) which are not comparable to other included studies. We intended to perform moderator analysis using meta‐regression, but we were able to do so on a limited subset of climate driver/outcome pairs because of scant evidence leading to insufficient degrees of freedom.

In the second step, we explored the variation between countries including evidence from correlational studies. For this, we computed median effects from correlational and regression studies separately to identify patterns in the direction of effects. Specifically, we synthesised results from studies using medians of correlation coefficients and SMDs from regression coefficients to discern whether the prevailing trend suggested a positive or negative association between each climate driver/outcome pair (We report box plots for India and Indonesia, the two countries with the largest amount of evidence, in Online Appendix S1L). The results of the first and second steps of the analysis are jointly presented in Sections 4.3.1 (effects of El Niño) and 4.3.2 (effects of +IOD).

However, given that averaging one effect over several regions or provinces may display null (or no systematic) effects, it is necessary to further disaggregate effects to appreciate the regional variation within each country. The third step thus consisted of country or regional analyses to disentangle the variability of effects within countries. Specifically, we systematically analysed the full text of the included studies and, where possible, extracted qualitative information on factors mitigating or facilitating the cascading effects of the two climate drivers, the within‐country heterogeneity, and other contextual factors. Given the great heterogeneity of included estimates and the fluctuating nature of detected effects within and between countries, we developed focused analyses for countries with the largest evidence base (India, Indonesia, the Philippines, Malaysia, Bangladesh) and two key regions of interest (Lower Mekong River Basin and Oceania). To shed light on the variation across reported cascading effects, we provided insights from the narrative analysis of individual study results.

Moreover, qualitative information was extracted regarding the effects of other drivers such as La Niña. For the analysis, we grouped the studies by climate driver/outcome pair to ensure the comparability of results. This is because El Niño and +IOD have different effects across the considered region.

## 4 Results

### 4.1 Description of Studies

#### 4.1.1 Results of the Search

*We searched 12 academic databases and 14 institutional websites in January 2024, which resulted in 10,113 records*. Of these, 3586 were identified through the electronic searches of academic databases and 6527 were identified through other sources such as searching specialist and institutional websites, consultation of experts, and citation tracking. We found that 101 records met our inclusion criteria, of which 89 represent unique studies and 12 are linked publications.^3^ The diagram in Online Appendix S1F provides a detailed summary of the records excluded at each screening stage and the reasons for their exclusion.

#### 4.1.2 Overview of the Evidence

*All included studies use quantitative methods. We found no qualitative studies that met our inclusion criteria*. Most of the studies were published in the last 10 years (60 of the 89 studies), indicating an increasing volume of empirical literature on the cascading effects of El Niño and +IOD.

**Figure 6:**
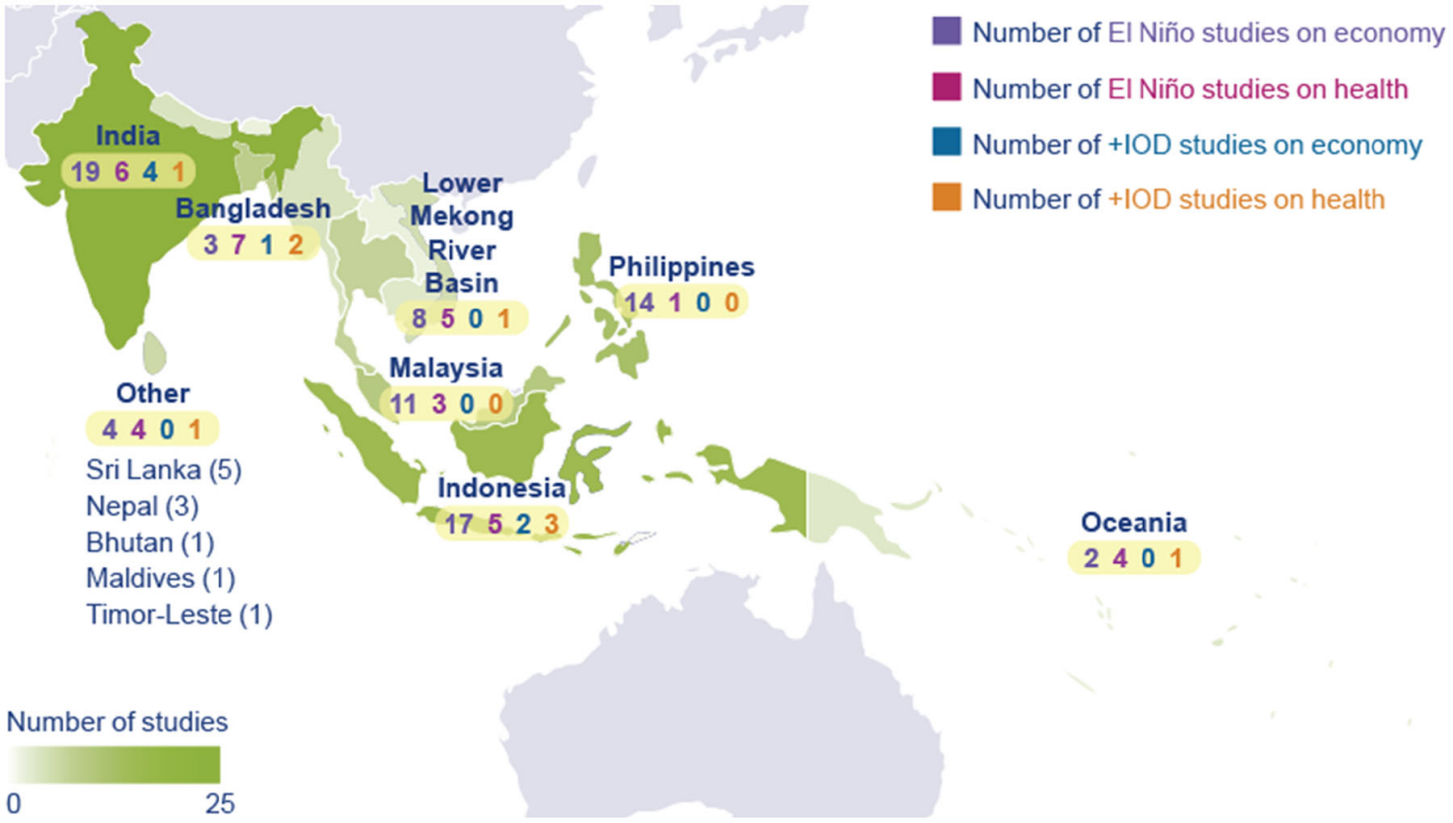
Studies by country. *Note:* The map does not include one multi‐country study from South East Asia which uses data from Cambodia, Indonesia, Philippines, Malaysia, Myanmar, Thailand and Vietnam (Ismail and Chan 2020) and does not report effects separately for each country. *Source:* 3ie (2025).

More than half of the studies were conducted in South East Asia, whilst approximately one out of three studies focus on the effects across the Indian subcontinent. Studies set in Oceania represent 6% of the total evidence base. The evidence is concentrated in five countries: India and Indonesia, followed by the Philippines, Malaysia, and Bangladesh. Less than 20% of included studies evaluate the effects of the two climate drivers of interest in other countries in the region. Figure 6 provides an overview of the distribution of studies by country.

The effects of El Niño in the Indo‐Pacific region were assessed in 88 studies, with wide variation in how it was measured. Sixteen of these studies also looked at the effects of +IOD, and only one additional study exclusively focused on +IOD. None of the studies examining both El Niño and +IOD estimated a combined effect; rather, they reported the effect of each climate driver separately.

Fifty‐six studies provided evidence on the effects on economic outcomes while 33 studies focused on health outcomes. We did not identify studies looking at the effects on social conflict, migration, or food security, and nutrition outcomes. ‘Production’^4^ and ‘productivity’^5^ were the most frequently reported economic outcomes (40 studies), with almost half of the included studies focusing on either one. These outcomes measure agricultural production and yield and can be potentially considered in the context of food security. Other economic outcomes in this literature were consumer and commodity prices (9 studies), aggregated production (4 studies), trade (3 studies), investments (2 studies), and consumption and total income and wealth (1 study).

Amongst health outcomes, the incidence of vector‐borne diseases such as dengue was the most studied cascading effect (20 studies); followed by cholera (5 studies) and other enteric diseases (7 studies).

Table 3 presents the median duration, in years, of the data sets used by studies in each outcome category. We computed this based on the start and end point of data series used by included studies in their analyses. Studies that examined economic outcomes used the longest data sets. Studies that examined aggregated production, prices, production, productivity, investments, and trade measures encompassed between approximately 25–48 years of data. The single study looking at household consumption and income used cross‐sectional data. For studies on health outcomes, those studying enteric infections (other than cholera) used the longest time periods (24 years), followed by studies on vector‐borne diseases (22 years).

**Table 3:** Observation period captured by studies for each outcome.

Outcome	Median (years)	IQR (years)	No. of studies
Economic outcomes			
Production	48.0	17.8	25
Aggregated production	35.0	16.0	4
Prices	35.0	30.0	9
Productivity	31.5	15.3	24
Trade	30.0	17.0	3
Investments	24.8	11.1	3
Consumption and expenditures	0.0	0.0	1
Total income and wealth	0.0	0.0	1
Health outcomes			
Enteric infections and diseases	24.0	6.3	7
Vector‐borne diseases	22.0	16.0	20
Cholera	15.0	8.0	5
Respiratory ailments	10.0	0.0	1
Direct injuries and fatalities	4.5	0.0	1

*Note:* The ‘enteric infections and diseases’ category does not include cholera. The number of studies does not add up to the studies identified in the search and screening process, as some of these reported estimates for more than one outcome.

We identified multiple indices and metrics used by the authors of included studies to capture the occurrence and intensity of the climate drivers in their analyses. The intensity of +IOD is represented by the anomalously positive SST gradient (Dipole Mode Index) between the western equatorial Indian Ocean (50° E–70° E and 10° S–10° N) and the southeastern equatorial Indian Ocean (90° E–110° E and 10° S–0° N).

For El Niño Modoki, the SST anomaly in three regions is averaged by the ENSO Modoki Index^6^ (Ashok et al. 2007). However, for El Niño, there is a diversity of strategies to measure its intensity and occurrence. These range from indices of SST anomalies in specific regions (Niño 1.2, Niño 3, Niño 3.4, Niño 4), a standardised index of the SST anomaly in Niño 3.4 region called Oceanic Niño Index, measures of atmospheric pressure differences between Tahiti and Darwin or the Southern Oscillation Index, and a composite measure that considers both atmospheric and oceanic variables called the Multivariate ENSO Index, amongst others. An exhaustive list of all measures used can be found in Online Appendix S1B (El Niño/+IOD metric column).

Whatever the choice of metric, some studies included the El Niño‐ or +IOD‐related index directly in the analysis (61 studies), while other studies used the index to identify the climate driver occurrences based on thresholds for the metric used and provided results specific to El Niño or +IOD occurrences (25 studies). Some studies employed both approaches (3 studies). We did not find enough studies reporting effects for stronger or weaker El Niño and +IOD events separately to explore whether the effects varied by the intensity of the climate driver.

### 4.2 Risk of Bias in the Included Studies

**Figure 7:**
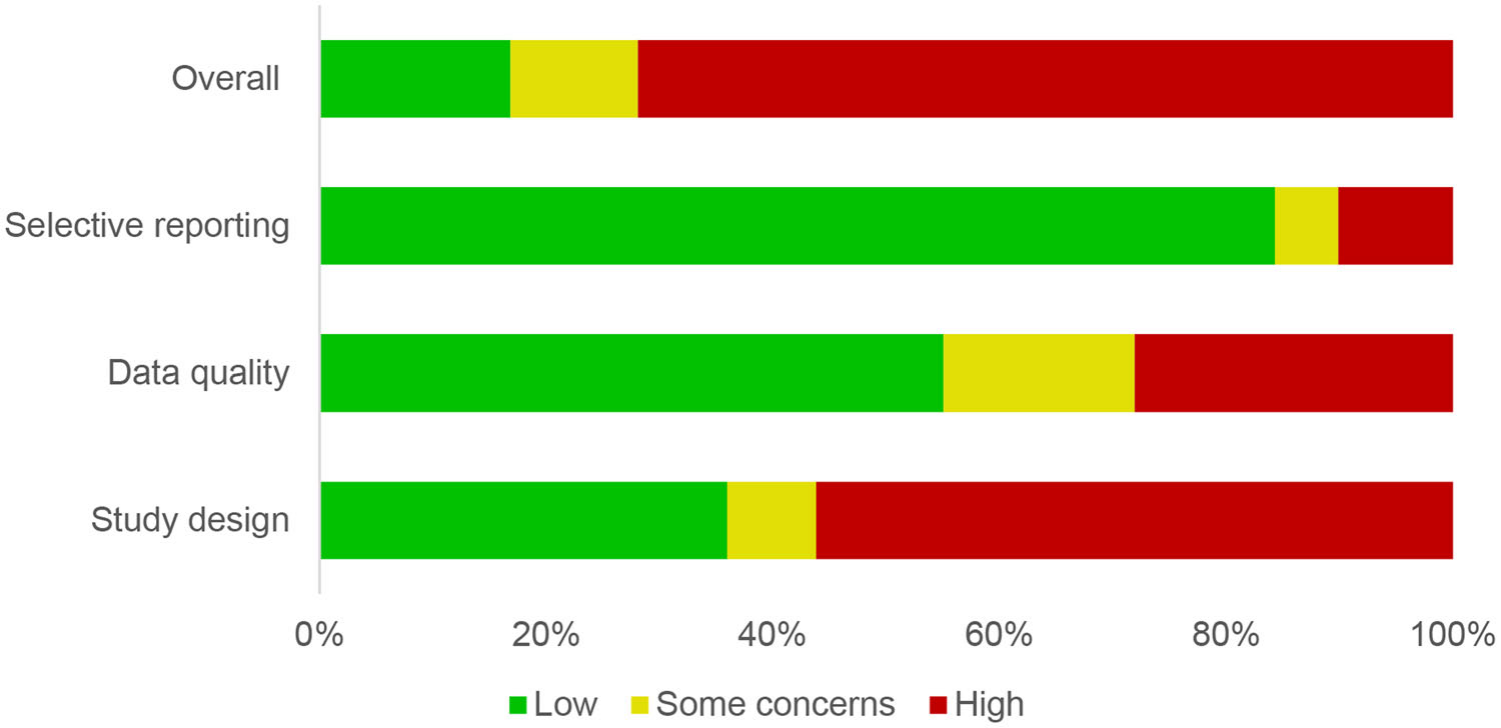
Risk of bias of included studies. *Source:* 3ie (2015).

We found that most of the results from studies meeting our inclusion criteria had a high risk of bias (Figure 7). The main concerns were related to study design. Most studies did not properly account for factors that could have affected the results. For instance, the effects of climate drivers were not isolated from changes in temperature or precipitation, technological innovations, or changes in population immunity. Additionally, it is not common practice to present findings from time series analysis both with and without adjustment for autocorrelation (patterns in data that repeat over time)—despite Kovats et al. (2000; 2003) suggesting this over 20 years ago when they assessed literature on the effects of El Niño on health. Furthermore, many of the studies did not provide evidence supporting their choice of methods or the reliability or robustness of their findings.

Regarding the quality and appropriateness of the data used in these studies, most authors used publicly available data from official sources. However, we marked down studies that used data for only one El Niño or +IOD event or failed to account for the cyclical nature of these climate drivers. For instance, studies that only used El Niño years in the analysis were considered to have a high risk of bias, as climate drivers do not follow calendar years, and there is no official definition of what qualifies as an El Niño event.

Finally, we identified few studies with issues related to selective reporting, where conclusions were not consistent with the unit of analysis, and the reported findings did not correspond to all intended analysis, indicating potential ‘data dredging’ and selective reporting. A summary rating based on a detailed assessment of each included study is reported in Online Appendix S1K.

### 4.3 Effects of Climate Drivers

We present the effects of climate drivers in two parts. First, we provide an overview of the findings across the Indo‐Pacific, which includes meta‐analyses of regression studies and a synthesis of correlational studies. We then delve deeper into specific country‐level analysis from both regression and correlational studies. Findings are presented separately for El Niño and +IOD.

#### 4.3.1 Overall Findings on the Effects of El Niño in the Indo‐Pacific

Overall, El Niño likely decreases production and productivity in the Indo‐Pacific. However, it does not seem to change prices or the incidence of vector‐borne diseases. There are too few studies to conclude on other outcomes such as trade, investment, household income, incidence of cholera, enteric infections, physical injury, and respiratory infections. These findings must be interpreted with caution, given the large variation across studies and the high risk of bias for most studies.

Table 4 provides an overview of the overall evidence base with key findings on the effects of El Niño across the Indo‐Pacific. Detailed explanations of the meta‐analysis findings, including forest plots showing the overall estimate and the weighted contributions of individual study estimates, are provided in Online Appendix S1G. The criteria determining selection of effect estimates for data extraction can be found in Online Appendix S1H. Unfortunately, the analysed body of evidence did not allow us to perform meta‐regression analysis by country; nonetheless, the distribution of correlation coefficients and effect sizes by outcome and region are presented in Online Appendices S1I and S1J.

**Table 4:** Number of studies and effects of El Niño by outcome.

Outcome	Regression studies	Correlation studies	Effects of El Niño	Take‐home message
Economic			56 studies	
Production	13	11	Meta‐analysis (MA) of regression coefficients for all regions pointed to a moderate negative effect on agricultural production: SMD = −0.33 (*p* = 0.004), in line with most correlational studies for both the Indian subcontinent and Indian Ocean (*r* = −0.19) and for South East Asia (*r* = −0.29).	Likely decrease in production
Productivity	13	9	MA for all regions indicated a moderate decrease in productivity: SMD = −0.36 (*p* = 0.02), contrasting most correlational studies for the Indian subcontinent and Indian Ocean (*r* = 0.05) and for South East Asia (*r* = 0.05).	Likely decrease in productivity
Prices	8	1	MA for all regions indicated large but not significant increase in prices (SMD = −0.48, *p* = 0.11). Only one correlational study with five estimates (from as many countries) (*r* = 0.05).	No effects on consumer and commodity prices
Aggregate production	4	1	MA not possible. Most regression findings pointed to a negative effect (*g* = −0.10).	Too few studies for overall conclusions
Investments	3	0	MA not possible. Most regression findings pointed to a negative effect (*g* = −0.06).	Too few studies for overall conclusions
Trade	3	0	MA not possible. Most regression findings pointed to a negative effect (*g* = −0.08).	Too few studies for overall conclusions
Health			31 studies	
Vector‐borne diseases	8	10	MA for all regions suggested a very small but not significant effect on incidence of disease (SMD = 0.02, *p* = 0.93). Most correlation coefficients for the Indian subcontinent and Indian Ocean (*r* = 0.07), South East Asia (*r* = 0.02) and Oceania (*r* = 0.29) pointed to a decrease in incidence.	No effects on vector‐borne diseases
Cholera	1	3	MA not possible. Correlational studies pointed to small increase in cholera in Bangladesh (*r* = −0.23).	Too few studies for overall conclusions
Other enteric infections	3	4	MA not possible. Most regression coefficients pointed to an increase in the number of infections (*g* = −0.31), while correlational studies pointed to fewer infections (*r* = 0.13).	Too few studies for overall conclusions

*Note:* Estimates for all regions are Standardised Mean Differences (SMD) presented alongside their respective *p* values from a meta‐analysis, with robust variance estimation using regression coefficients only. Medians of the correlation coefficients (*r*) or effect sizes from regression coefficients (*g*) are used to describe the distribution of results where MA was not possible (too few studies) or recommended (correlation coefficients). We have reversed the sign of estimates for prices and disease incidence such that a negative effect reflects an undesirable result (inflation or higher disease incidence). Other outcomes identified in the literature are not included in the table because they were reported by single studies (household income and consumption, direct injuries and fatalities, and respiratory ailments) or not reported at all (conflict, migration and food security, and nutrition outcomes).

Abbreviation: MA, meta‐analysis.

##### 4.3.1.1 Economic Outcomes

From the meta‐analysis, we found a *moderate negative effect that suggests El Niño is likely to decrease production* (SMD = −0.33, *p* = 0.004, 95% CI [−0.53, −0.13], 13 studies^7^). The distribution of correlation coefficients on the association between El Niño and production^8^ is consistent with the meta‐analysis (*r* = −0.20, 11 studies^9^). At the regional level, most correlations studies found that El Niño events are associated with decreased production in both the Indian subcontinent (*r* = −0.19, 7 studies) and South East Asia (*r* = −0.29, 3 studies), with the largest median correlation coefficient reported in India (*r* = −0.20, 6 studies).

We also found a *moderate negative effect on productivity* ^10^ (SMD = −0.36, *p* = 0.02, 95% CI [−0.67, −0.06], 12 studies^11^). However, most of the studies using correlational analysis pointed to a positive association between El Niño and productivity in both the Indian subcontinent (*r* = 0.05, 5 studies) and South East Asia (*r* = 0.05, 4 studies). No studies reported results for Oceania.

We did *not identify any effects of El Niño on prices* ^12^ (SMD = −0.48, *p* =0 .11, 95% CI [−1.10, 0.15], 8 studies^13^). We did not have sufficient data for meta‐analysis on aggregate production (4 studies), trade (3 studies), investment (3 studies), total income (1 study), and consumption (1 study). The median effect sizes across studies for each of these outcomes point to negative effects from El Niño.

##### 4.3.1.2 Health Outcomes

From the meta‐analysis, we found *no significant effect of El Niño on incidence of vector‐borne diseases* (SMD = 0.02, *p* = 0.93, 95% CI [−0.40, 0.44], 8 studies^14^). From the correlational studies, there is a suggestion of fewer vector‐borne infections from El Niño events (*r* = 0.14, 10 studies^15^). The direction of the effects found in most correlational studies is consistent with the results from the meta‐analysis, although those findings were not statistically significant.

Due to the limited number of eligible studies, meta‐analysis was not possible for the incidence of cholera (1 study), other enteric infections (3 studies), and direct injuries and fatalities (1 study). Findings from individual studies reporting on enteric infections are inconsistent. More details are available in the country‐level analyses that follow. One study from India (Lam et al. 2019) reported increased physical injuries and fatalities associated with El Niño.

#### 4.3.2 Overall Findings on Effects of +IOD in the Indo‐Pacific

Very few studies assess the effects of +IOD in the Indo‐Pacific, which makes it difficult to draw any conclusions on effects.

Meta‐analysis was not possible for any outcome. One study on production (Suratno 2022) found no statistically significant effects on fish captures in West Sumatra, Indonesia. Two studies on productivity reported mixed effects (median *g* = −0.07): a small negative effect on fish caught per unit effort^16^ in India (Zacharia et al. 2020) and increased rice yields in Indonesia (Kusuma 2019).

Two studies consistently reported a reduction in the incidence of dengue in Bangladesh (Banu et al. 2015) and in the number of dengue cases in Vietnam (Nguyen et al. 2020) (median *g* = −0.62). In Bangladesh, one study reported an increase in cholera infections from +IOD, with more early infections for Dhaka and Matlab, followed by a decrease in infections for Dhaka 4 to 7 months after +IOD conditions (Hashizume et al. 2011).

One study (Kim et al. 2016) assessed the independent effects of +IOD and El Niño on childhood pneumonia in Papua New Guinea. The authors found that while +IOD was associated with reduced incidence of childhood pneumonia (4.3% reduction in risk), El Niño had an opposite effect (0.57% increase in risk). The effects were consistent across most regions of Papua New Guinea, indicating the influential role these drivers play irrespective of local health resources or local climate factors such as rainfall and temperature.

From the correlational studies, we included five studies reporting contrasting findings on the association between +IOD and vector‐borne diseases (*r* = 0.36) and two studies reporting mixed results on production (*r* = 0.01).

We did not identify studies that assessed the combined effects of El Niño and +IOD on any outcome.

##### 4.3.2.1 Temporal Distribution of Effects

One question we wanted to answer is how long the effects of climate drivers last. We did not have sufficient data to run a meta‐regression to answer this question with moderator analysis. Correlational studies evaluating the temporal distribution of the effects of El Niño and +IOD used between one and two lags, on average. Comparing the results from these studies with those from studies that did not consider lags, we observed that the negative effect on production associated with the onset of El Niño (*r* = −0.2, 7 studies estimating a correlation with no lags) seems to diminish with time (*r* = −0.04, 3 studies using any lag).

With regard to production, studies using no lag showed a very small negative association (*r* = −0.04), while studies using any lag had a positive median correlation (*r* = 0.32, 2 studies). For health outcomes, however, the reduced incidence of vector‐borne diseases and enteric infections was observed, both during the occurrence of El Niño (*r* = 0.08, 6 studies looking at vector‐borne diseases; and *r* = 0.37, 3 studies looking at enteric infections other than cholera) and with time (*r* = 0.14, 3 studies; and *r* = 0.13, 2 studies, respectively) at any lag.

### 4.4 Country‐Focus Analysis

Given the variation effects by geography, we further disentangled the sources of variation to understand patterns specific to a country or region. In this section, we present the findings of country and region‐specific analyses. The patterns have been drawn on the qualitative and quantitative descriptive analysis of the studies included in the review. Therefore, the results need to be interpreted with caution, given the great variability in the types and risk of bias for included studies. The primary factors that help to explain the variation in results include type of crop, the region, province or district considered, the time span analysed, and the metric used to measure El Niño or +IOD events.

**Figure 8:**
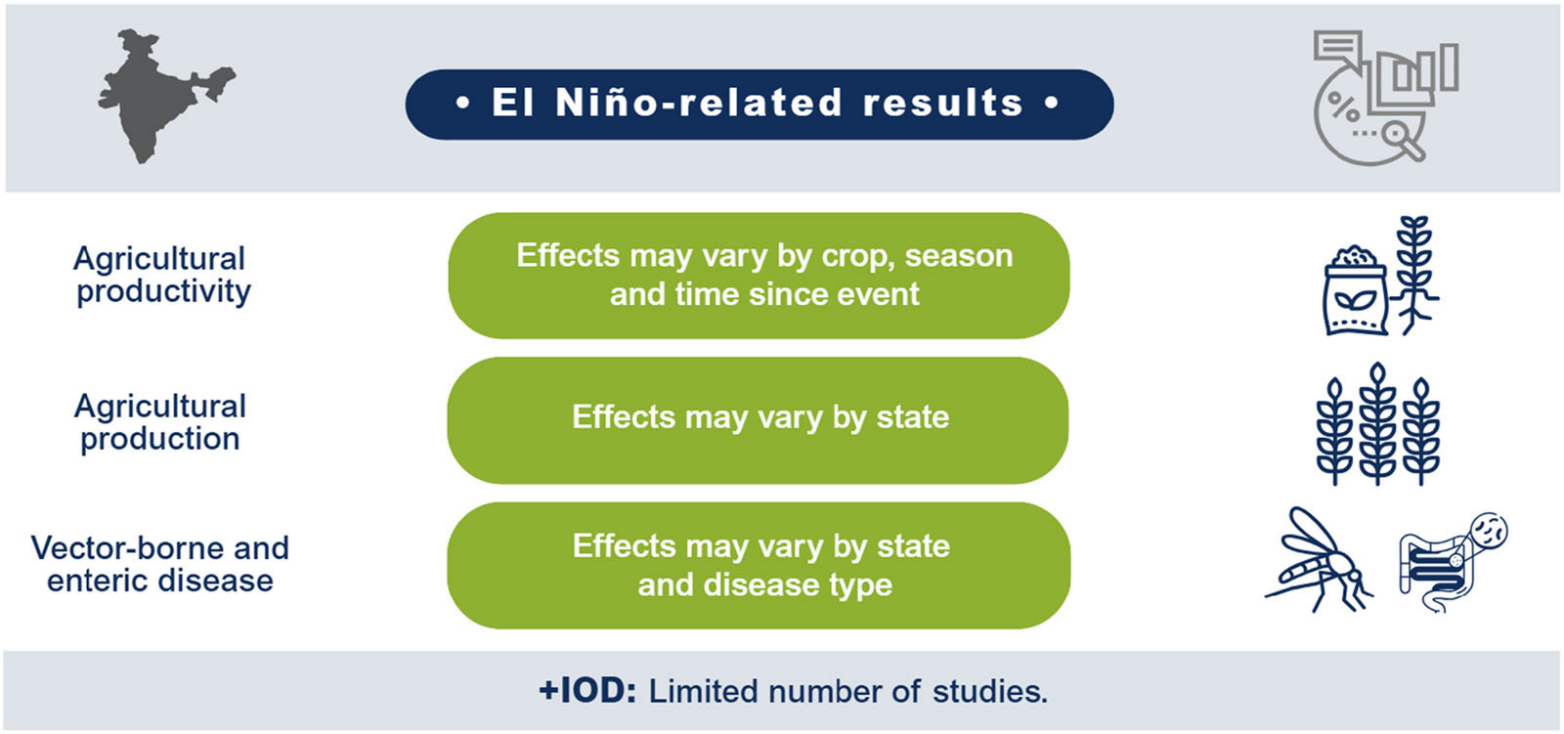
Main findings for India.

#### 4.4.1 India

##### 4.4.1.1 Context

El Niño events in India are often associated with less rainfall and increased likelihood of drought (Saikranthi et al. 2018; Saini and Gulati 2014). A pronounced weakening of the Indian summer monsoon rainfall during the eastern Pacific El Niño has been observed (Qadimi et al. 2021). Although the overall rainfall decreases at the country level, it varies by state. Specifically, the northeastern regions of India receive more rainfall during El Niño (Kiran Kumar and Singh 2021), while the southeastern region experiences weakening of rainfall intensity (Deivanayagam et al. 2024). On the other hand, the influence of +IOD involves an increase in the rainfall over the country due to changes in the wind circulation and anomalous SST (Kripalani and Kumar 2004; Cherchi et al. 2021) (Figure 8).

##### 4.4.1.2 Summary of the Evidence

**Figure 9:**
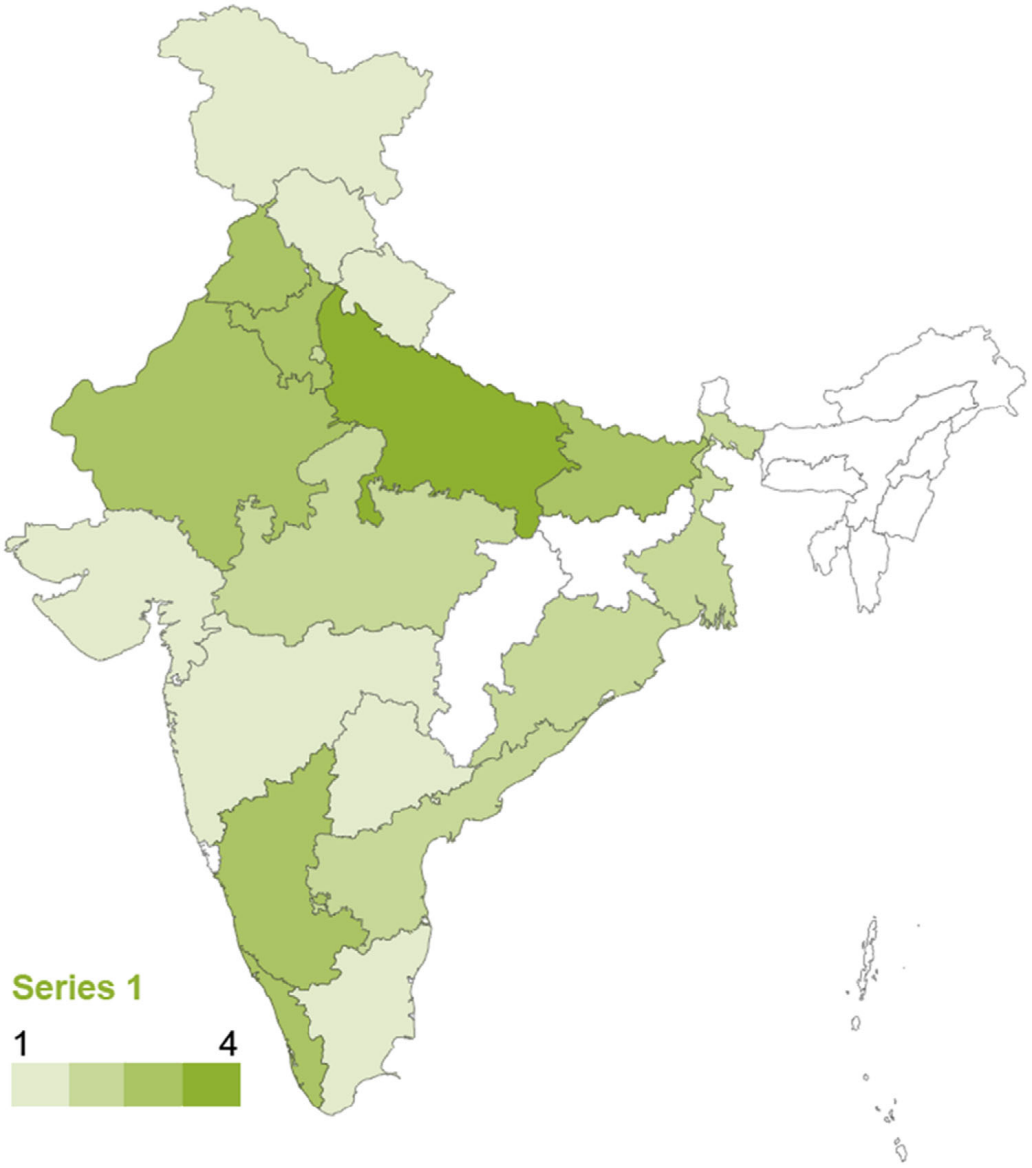
Number of studies in India that report state‐specific effects.

We included 25 studies providing analyses specific to India. These include studies that solely focused on India, as well as multi‐country analyses. Of these 25 studies, only five examined the effects of +IOD (Amat and Ashok 2018; Bhatla et al. 2023; Kakarla et al. 2019; Sahu et al. 2020; Zacharia et al. 2020). The remaining 20 studies examined the effects of El Niño on various outcomes. Productivity and production^17^ were the most reported outcomes in studies that focused on India, with nine studies reporting on productivity and eight on production. For health‐related outcomes, two studies reported on enteric infections and disease outcomes (other than cholera) and three reported on vector‐borne disease outcomes (Figure 9).

Of the 25 studies, 16 did not report state‐specific results, and some presented results on multiple states. The most represented state was Uttar Pradesh (4 studies) (Bhatla et al. 2020; 2023; Nageswararao et al. 2018; Amat and Ashok 2018). This is followed by Rajasthan, Punjab, Haryana, Karnataka, and Bihar (3 studies each). Two studies presented results on Odisha, with one of these providing estimates for three climate drivers: El Niño, El Niño Modoki, and +IOD.

##### 4.4.1.3 Findings on the Effects of El Niño in India

The evidence from India indicated that the effects of El Niño on agricultural productivity varied by crop, season, and time since the event.

The effects of El Niño on agricultural production varied by state. Decreased output was more likely to materialise in western and northwestern states.

El Niño was associated with wide geographical variation in the incidence of some vector‐borne and enteric diseases due to the frequency of monthly precipitation.

We identified little evidence of +IOD's effects on agricultural production, agricultural productivity, and vector‐borne diseases.

We found five studies that examined the effect of El Niño on productivity outcomes in India using correlational analysis, and another five using regression analysis. The median correlation coefficient between El Niño and productivity in India was close to zero (*r* = 0.05, 124 coefficients^18^) (Garnett and Khandekar 1992; Nageswararao et al. 2018; Panda et al. 2019; Raj et al. 2020; Sahu et al. 2020), while the median effect size from studies using regression analysis was large and positive (*g* = 0.31, 4 estimates) (Giuseppe et al. 2022; Ferris 1999; Rao et al. 2012; Zacharia et al. 2020).^19^ This reflects the variability of findings between and within studies. Notably, the type of crop is the main source of variation underpinning the effects on productivity reported in the correlational studies; with negative effects on rice, wheat, and rabi oilseeds. Moreover, the effects of El Niño on agricultural productivity in India vary with time. For example, di Giuseppe et al. (2022) reported a reduction in wheat crop yields in El Niño years, while Ferris (1999) found an increase in yields the year after El Niño took place.

Using monthly data grouped in quarters, Garnett and Khandekar (1992) found varying effects of El Niño by season. Their results suggest that El Niño was associated with an increase in rice yields (although not statistically significant) from December to February, and a reduction in the period between March and August—implying that the effects are observed during periods of drought or less rainfall.

Nageswararao et al. (2018) reported heterogeneous effects depending on the considered crop. Specifically, the authors found that wheat, rapeseed‐mustard, and rabi oilseed are more resistant to El Niño anomalies, whilst gram and rabi food grain yields are more sensitive and negatively affected by El Niño events. Another source of variation in the results was the metric used to measure the driver.

For instance, Sahu et al. (2020) reported correlation coefficients between rice productivity in Bihar and various indices measuring El Niño,^20^ with results varying in both direction and magnitude. This might be because metrics differ in their definitions, focus areas, and purposes. The Trans‐Niño Index, for example, is quite narrow, and its purpose is to understand the transition and gradient of SST anomalies in the Pacific Ocean.

Agricultural production output varies by state. We found six studies that examined the effect of El Niño on production outcomes in India using correlational analysis (Amat and Ashok 2018; Bhatla et al. 2020; 2023; Krishna Kumar et al. 2004; Panda et al. 2019; Selvaraju 2003). The median correlation coefficient (*r* = −0.2, 133 coefficients) suggested that most studies found that El Niño events can reduce production in India.

Selvaraju (2003) used national‐level data between 1950 and 1999 to assess the effects of El Niño on a wide array of crops (including foodgrains, cereals, and pulses), and found negative effects across them all.

However, this finding was not consistent with other studies. Bhatla et al. (2020) explored the association between El Niño and agricultural production in the lower, middle, upper, and Trans Indo‐Gangetic Plain regions separately. The authors found that results varied by crop, with consistent negative effects for rice but varying effects by region for wheat, maize, pulses, and sugarcane. Similarly, Bhatla et al. (2023) explored the effects of El Niño on maize, rice, pulses, and sugarcane production, using data between 1966 and 2011, and found that effects were consistently negative for rice and pulses but varied by region for maize and sugarcane.

Amat and Ashok (2018) analysed the effects of El Niño on kharif crop production and found that El Niño conditions were associated with lower production in most states, with the exception of Kerala and West Bengal, where an increase in kharif crop production was observed. Panda et al. (2019) found that rice and maize production increased during El Niño events in Odisha. This pattern was consistent across different El Niño indices and seasons.

We also identified two studies using regression analysis to explore the effects of El Niño on agricultural production (Abdolrahimi 2016) and aquaculture (Bertrand et al. 2020), neither of which found evidence of significant El Niño effects in India.

We found two studies that examined the association between El Niño and aggregated production in India (Cashin et al. 2015; Laosuthi and Selover 2007), both finding no effect during the year of the event. However, Laosuthi and Selover (2007) found that economic growth declined 1 year after El Niño conditions, and the effect attenuated a year later.

We included two studies looking at the effects of El Niño Modoki on economic outcomes in India. Sahu et al. (2020) found that the ENSO Modoki Index is expected to reduce rice productivity, and the effect is larger between June and September (during the Indian summer monsoon rainfall). Similarly, Amat and Ashok (2018) found a significant correlation between kharif crop production and the summer monsoon rainfall, and a reduction associated with the ENSO Modoki Index for most major kharif crop‐producing states in India—similar to the results reported for El Niño.

With regard to health outcomes, three studies examined the relationship between El Niño events and dengue and malaria cases (Azad and Lio 2014; Kakarla et al. 2019; Pramanik et al. 2020), all using correlational analysis. We found 18 coefficients within these studies with a median of 0.178, suggesting that most analyses found that El Niño conditions may be associated with a reduced incidence of vector‐borne diseases, though findings from individual studies are not consistent.

Pramanik et al. (2020) used state‐wide data between 2014 and 2017, which suggested mixed effects of El Niño on dengue incidence in two cities with frequent dengue outbreaks. While the results showed a decreased incidence in Vishakhapatnam in the first 3 months following El Niño events, the authors found an increased incidence in Delhi in the first 2 months after the event. In both cities, dengue cases increased with rainfall.

Azad and Lio (2014) used country‐wide data and found that El Niño may be associated with increased malaria incidence and decreased dengue incidence between 1985 and 2009. The different effects are explained by the spread of the malaria epidemic being deeply rooted in poor communities, especially those living in remote forest areas. A study by Kakarla et al. (2019) assessed the effects of El Niño at the national level between 2010 and 2017 and reported a small, not statistically significant increase in the incidence of dengue.

Evidence on the effects of El Niño on enteric disease (other than cholera) was reported by Iyer et al. (2021) and Oluwole (2015). The former study employed regression analysis to assess the effects on the number of enteric fever cases between 1995 and 2017 and reported an increase in the incidence rate for Ahmedabad and a reduction for Surat associated with strong El Niño events. The authors suggested that one possible explanation was the relatively higher frequency of monthly extreme precipitation events in Ahmedabad during a strong El Niño compared to Surat (which are located inland and on the coast, respectively).

Oluwole (2015) explored the effect of El Niño on lathyrism epidemic cases and found a strong association between El Niño conditions and outbreaks in central provinces where people rely exclusively on the *Lathyrus sativus* legume for food. The study concluded that to prevent such outbreaks, food programmes should be implemented for vulnerable populations during El Niño‐induced droughts.

##### 4.4.1.4 Findings on the Effects of +IOD in India

Few studies measured the association between +IOD and economic and health outcomes in India. Sahu et al. (2020) explored the seasonal association between +IOD and rice productivity in Bihar and found the highest association for the quarter from April to June (*r* = 0.76). Two studies (Amat and Ashok 2018; Bhatla et al. 2023) estimated the correlation between +IOD and production outcomes. While Amat and Ashok (2018) found mostly positive associations between +IOD and kharif crop production in states other than Kerala and Karnataka, Bhatla et al. (2023) found that +IOD is associated with a reduction in crop production in many states (particularly sugarcane in Madhya Pradesh, Rajasthan, and Uttar Pradesh).

One possible explanation for the reduction in agricultural production reported by Bhatla et al. (2023) is that most of the studied +IOD years co‐occur with El Niño years, and El Niño‐rainfall relation is often dominant over the +IOD–rainfall relation. In other words, the rainfall reduction driven by El Niño prevails, leading to a contraction of agricultural production (Bhatla et al. 2023). As the only study examining health outcomes, Kakarla et al. (2019) reported an increase in dengue cases associated with +IOD, though the coefficient was not statistically significant.

##### 4.4.1.5 Findings for India at the State Level

To disentangle the heterogeneity underpinning the effects of El Niño and +IOD, we explored variations in findings by state. However, the results should be interpreted with caution, as the analysis was based on a limited number of studies. We found that El Niño was correlated with higher production in the southern and eastern coastal areas (with the highest correlation reported in Kerala), whereas it negatively affected production in western, northern, and inland areas (with the lowest levels reported in Karnataka).

**Figure 10:**
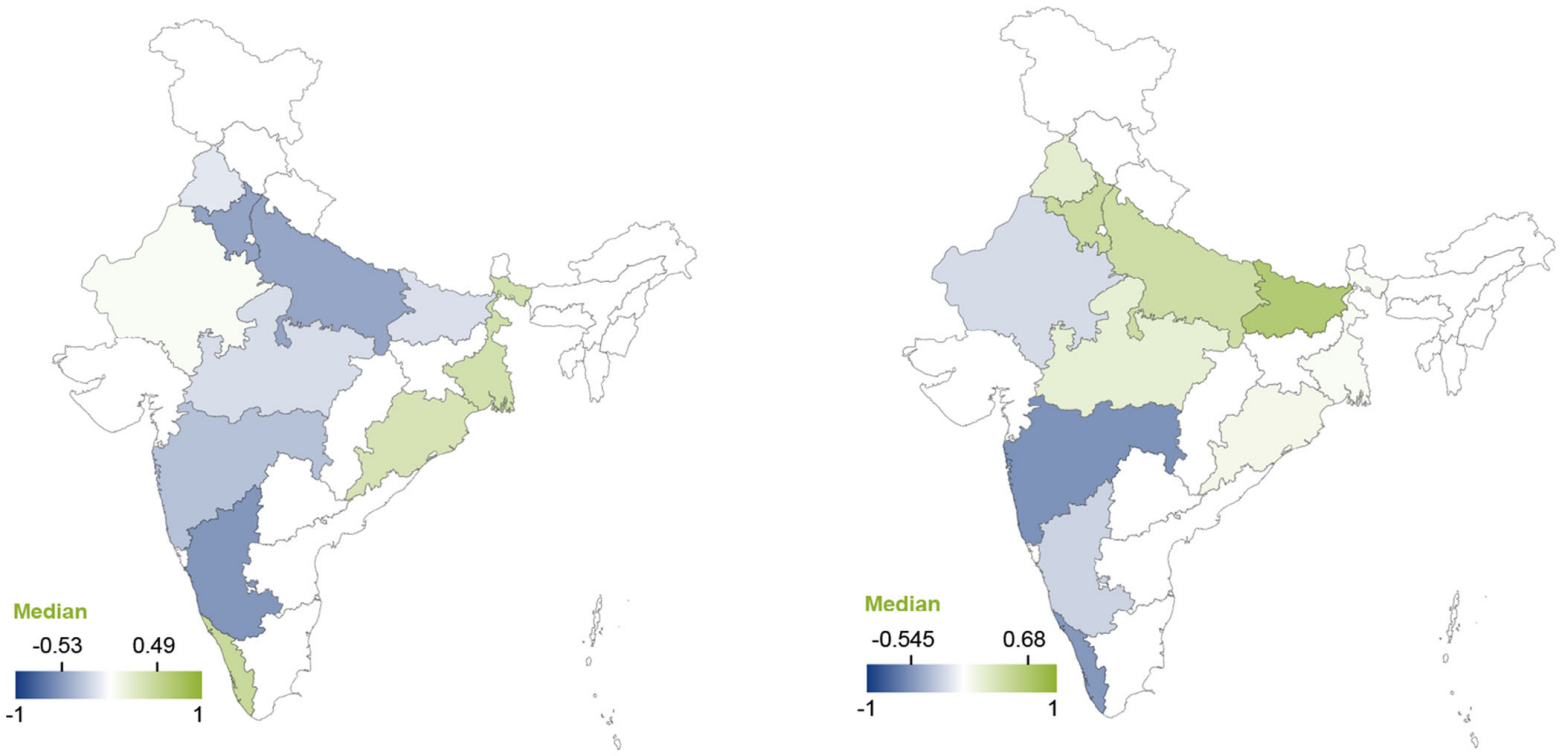
Effects of El Niño and +IOD on production by Indian state. *Note:* The map displays median values of correlation coefficients. Positive values indicate increased production. Negative values indicate decreased production. The maps contain coefficients from specific states (nationwide coefficients are omitted). For example, out of the 133 coefficients measuring the association between El Niño and production, the map for production shows only 38 that are specific to one of the states in the list. *Source:* 3ie (2025).

Conversely, single study results suggested that +IOD may be associated with lower production and harvest in the coastal states (with the lowest levels recorded in Maharashtra), whilst it may be correlated with higher production in the Great Indian Plains, just south of the Himalayas (with the highest levels reported in Bihar). The effects of El Niño and +IOD on production are displayed in Figure 10.

**Figure 11:**
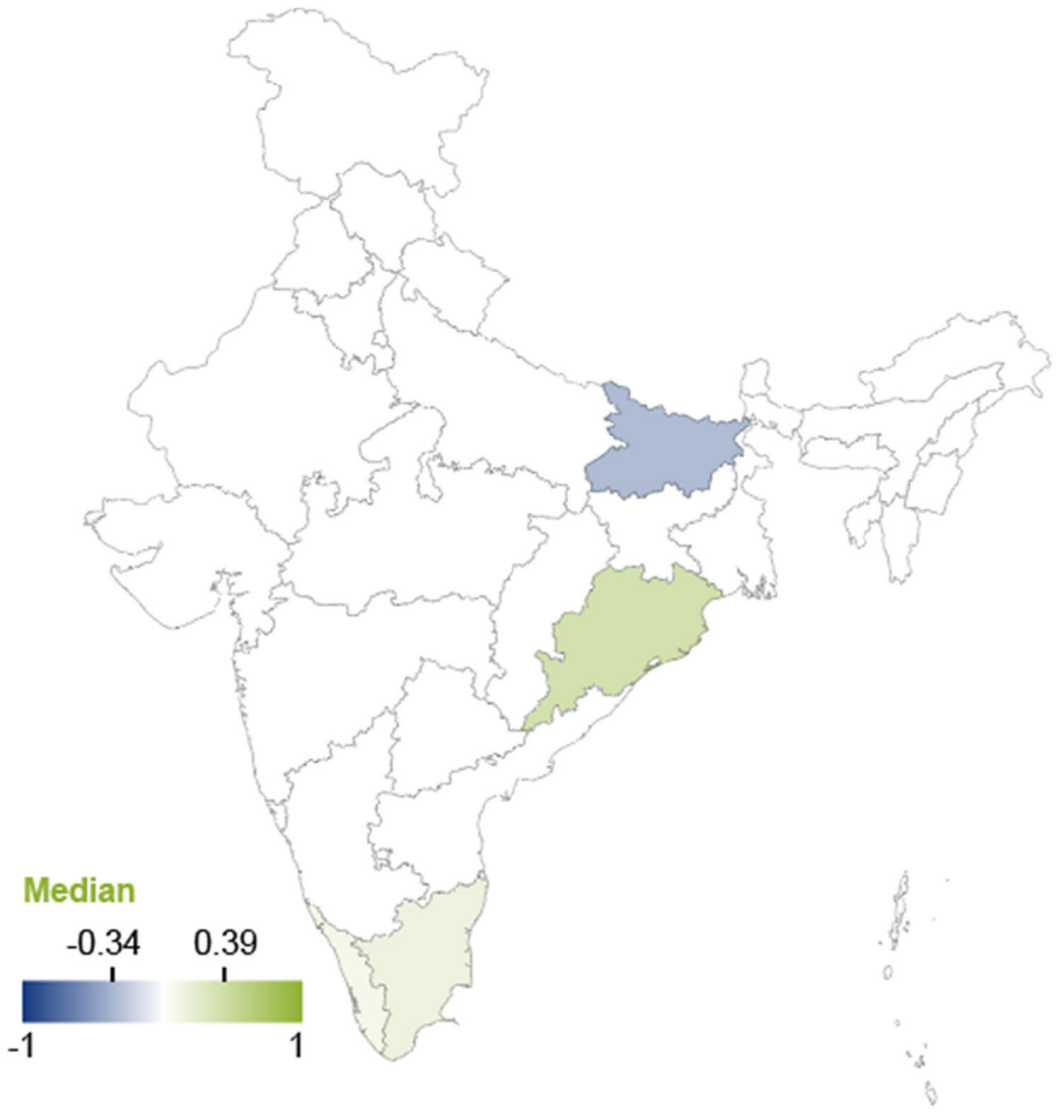
Effects of El Niño on productivity by Indian state. *Note:* The map displays median values of correlation coefficients. Positive values indicate increased productivity. Negative values indicate decreased productivity. *Source:* 3ie (2025).

Similar patterns (though with even less data than that available for production) were found for effects on productivity, with coastal areas positively affected and inland states adversely affected by El Niño events. The effects of El Niño on productivity by state are displayed in Figure 11.

**Figure 12:**
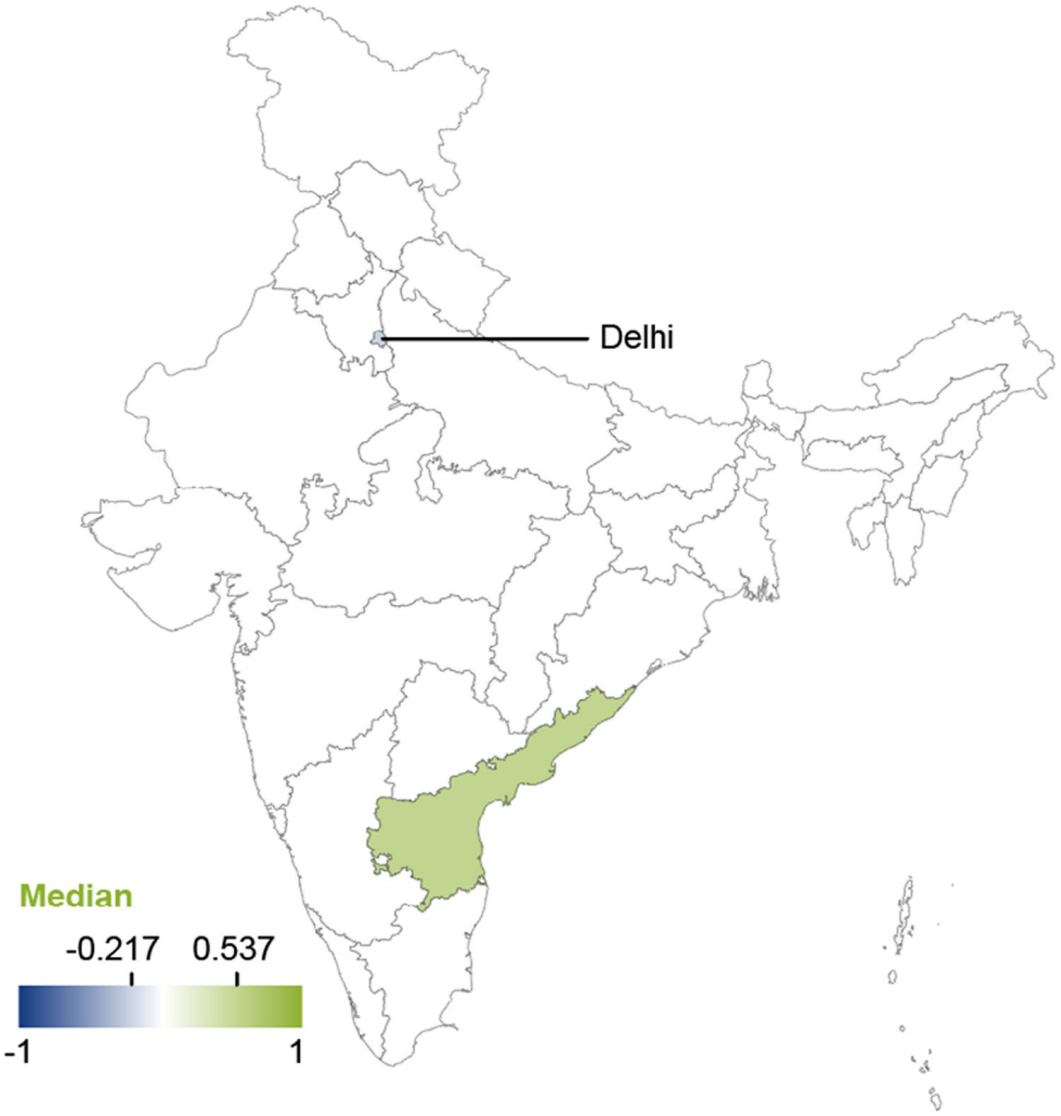
Effects of El Niño and vector‐borne diseases by Indian State. *Note:* The map displays median values of correlation coefficients. Positive values indicate decreased incidence of vector‐borne diseases. Negative values indicate increased incidence of vector‐borne diseases. *Source:* 3ie (2025).

The geographical stratification of the effects of El Niño on vector‐borne diseases indicates positive effects in the coastal state of Andra Pradesh (Pramanik et al. 2020) (implying a reduction of disease incidence) and negative median effects in Delhi (Ibid) (implying an increase in disease incidence), though the adverse effects in Delhi may vary with time. The effects of El Niño on vector‐borne diseases are displayed in Figure 12. It is important to note that the reported findings should be interpreted with caution, as they are derived from a single study.

**Figure 13:**
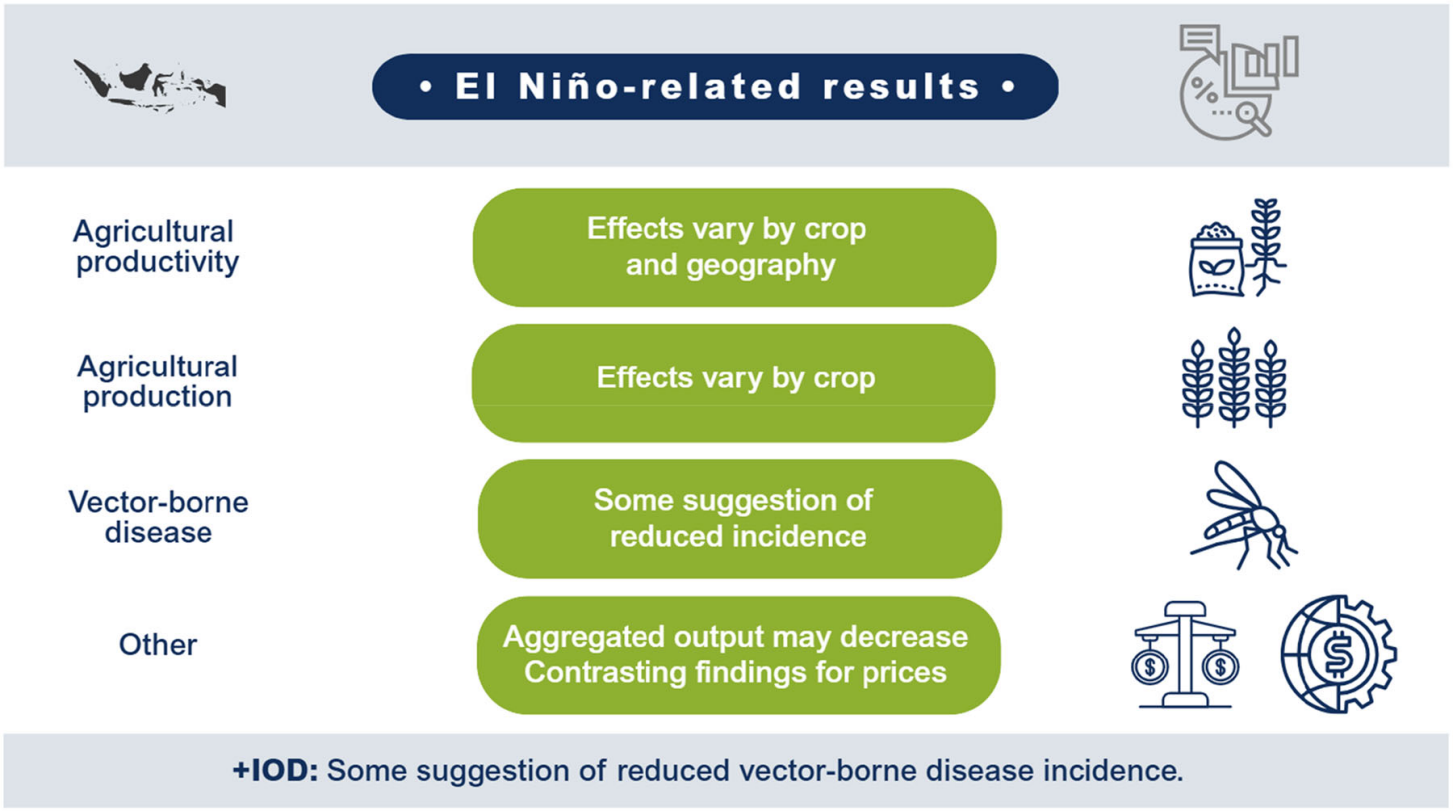
Main findings for Indonesia.

#### 4.4.2 Indonesia

##### 4.4.2.1 Context

In Indonesia, El Niño events reduce rainfall, especially in the period between September and December (Iskandar et al. 2019). Meteorological droughts associated with El Niño increase the risks of wildfires (Novitasari et al. 2019). As for +IOD, the effects vary within the country, as rainfall generally decreases in the islands of Sumatra and Borneo whilst increasing in the islands of Java, Sulawesi, and Irian Jaya (Nur'utami and Hidayat 2016) (Figure 13).

##### 4.4.2.2 Summary of the Evidence

Twenty‐four of the included studies provided analyses of Indonesia, of which seven (Abdolrahimi 2016; Azad and Lio 2014; Bertrand et al. 2020; Bekkering 2017; Cashin et al. 2015; Ismail and Chan 2020; Laosuthi and Selover 2007) presented multi‐country analyses. Of these 24 studies, 19 examined the effects of El Niño alone, while 3 examined the effects of both +IOD and El Niño (Harapan et al. 2020; Kusuma 2019; Prasetyowati et al. 2021) and 2 studies focused on effects specific to +IOD (Dhewantara et al. 2021; Suratno 2022).

Vector‐borne diseases, production, and prices are the most reported outcomes in studies that focused on Indonesia (six studies each). This is followed by productivity outcomes (four studies) and aggregated production (three studies). Upon disaggregating this information by climate driver, we found that the +IOD‐specific studies presented results on production outcomes (Suratno 2022) and vector‐borne disease incidence (Dhewantara et al. 2021). Of the three studies that examined the effects of both teleconnection patterns separately, two reported the effects on vector‐borne disease outcomes (Harapan et al. 2020; Prasetyowati et al. 2021) and only one study (Kusuma 2019) reported on productivity‐related outcomes.

Nine of the 24 included studies reporting results for Indonesia used correlational analysis methods to study the association of El Niño or +IOD with health and economic outcomes. The remaining studies used inferential analysis (mostly regression). There were too few regional analyses to present findings by province. We provide more details on the distribution of the median correlation coefficients at the country level in Online Appendix S1L.

The evidence from Indonesia indicated inconsistent effects of El Niño on agricultural production and productivity, with effects varying by types of crops for both outcomes (and for productivity also by geography). There was some evidence of association with decreased rice production but conflicting findings for rice yields and maize output and yields.

Most studies indicated that both El Niño and +IOD conditions were associated with a reduction in the incidence of vector‐borne diseases such as dengue and chikungunya.

While six studies found contrasting findings for prices, there is some suggestion of El Niño being associated with decreased aggregate measures of economic output. We did not find enough evidence on the effects of +IOD on other outcomes.

##### 4.4.2.3 Findings on the Effects of El Niño in Indonesia

Overall, we found varied associations between El Niño and health and economic outcomes in Indonesia. Vector‐borne diseases are the most reported outcome in studies using correlational analysis, with four studies reporting five coefficients. Based on findings from these studies, we found that El Niño conditions could reduce the prevalence of vector‐borne diseases.

Azad and Lio (2014) reported a negative correlation between SST anomalies caused by El Niño and annual cases of dengue and malaria at the national level (implying a reduction in the incidence of these diseases). Harapan et al. (2020) reported a decline in chikungunya incidence rates associated to El Niño. Prasetyowati et al. (2021) also explored effects on dengue and found a negative association (albeit very small). The expected decrease in vector‐borne diseases during El Niño events was further corroborated by Arcari and Tapper (2017), who used linear regression and found a significant reduction in dengue haemorrhagic fever incidence in Jakarta during the 1992–1995 and 1997–1998 El Niño events.

Only one study reported that El Niño is associated with an increased risk of dengue epidemic (Gagnon et al. 2001). However, the correlation coefficient was very small, and the authors used calendar years classified as ‘El Niño’ without further describing how this categorisation was done. As mentioned in Section 4.2, this practice undermines the credibility of the results, as the cyclicity of El Niño does not follow a calendar year. We found no studies exploring the effects of El Niño on other health outcomes in Indonesia.

Four studies explored the associations between El Niño and agricultural productivity. Nugroho and Nuraini (2016) used correlational analyses to examine its association with corn, paddy, and soybean crop yields, with results disaggregated by town/municipalities in the Banyumas District in Central Java. Nugroho (2013) performed a similar analysis, using Pearson's correlation to study the association between dryland paddy, corn, cassava, and soybean yields. This study focused on the Gunungkidul District in the highlands of South Central Java, and their results were also disaggregated by town/municipality under the district.

The median correlation coefficient from these two studies was positive (*r* = 0.057) and the range of coefficients went from −0.53 to 0.54, suggesting that results largely varied by district. Kusuma (2019) used geographically weighted regression analysis and Falcon et al. (2004) used time series regression to explore the effects of El Niño on paddy yields across time. Both studies found negative effects.

Five studies provided evidence on the effect of El Niño on production. Nugroho (2015) used correlational analysis and found that under El Niño conditions, maize production decreases (*r* = −0.5). The rest of studies used either analysis of variance (Bertrand et al. 2020) or regression analysis (Falcon et al. 2004; Utami et al. 2011; Abdolrahimi 2016). Results from regression analysis broadly suggest a negative association with a median effect size of *g* = −0.25. This was likely driven by Falcon et al. (2004), who found a large reduction in both area and production in tonnes of rice.

Abdolrahimi (2016) also found a reduction in rice production, but the magnitude was small, and Utami et al. (2011) found no effect. However, contrary to findings by Nugroho (2015), Utami et al. (2011) reported an increase in corn production associated with El Niño.

As for other economic outcomes, three studies using regression analysis (Cashin et al. 2015; Laosuthi and Selover 2007; Siregar et al. 2020) reported a negative effect of El Niño on aggregate production, with a median effect size of *g* = −0.20. Laosuthi and Selover (2007) also found that El Niño is negatively correlated with gross domestic product (GDP) growth (*r* = −0.02) and further reported a positive correlation with consumer prices (*r* = 0.063).

Five studies using regression analysis (Ahmad et al. 2019; Cashin et al. 2015; Fajri et al. 2019; Ismaya and Anugrah 2018; Khoiruddin et al. 2021) also reported the effect on prices, with varied results and a median of *g* = −0.01. Finally, one study (Bekkering 2017) reported the effects of El Niño on percentage stock return per month using price indices and trading volume, estimating effects for every quarter up to a year after the occurrence of El Niño.

##### 4.4.2.4 Findings on the Effects of +IOD in Indonesia

No correlational study was found for the association between +IOD and economic outcomes in Indonesia, while all three studies focusing on the association between +IOD and health outcomes reported on vector‐borne diseases (Dhewantara et al. 2021; Harapan et al. 2020; Prasetyowati et al. 2021). Findings suggested that +IOD conditions are associated with a reduction in the prevalence of vector‐borne diseases (*r* = 0.40).

Harapan et al. (2020) examined the effect on the number of chikungunya cases and found a decrease in its incidence. Similarly, Prasetyowati et al. (2021) found a moderate negative correlation with the Dipole Mode Index that measures +IOD. Dhewantara et al. (2021) focused on examining the association between +IOD and dengue incidence rates, and their results indicated negative, albeit weak correlation between the two, implying a decrease in dengue incidence associated with +IOD conditions.

**Figure 14:**
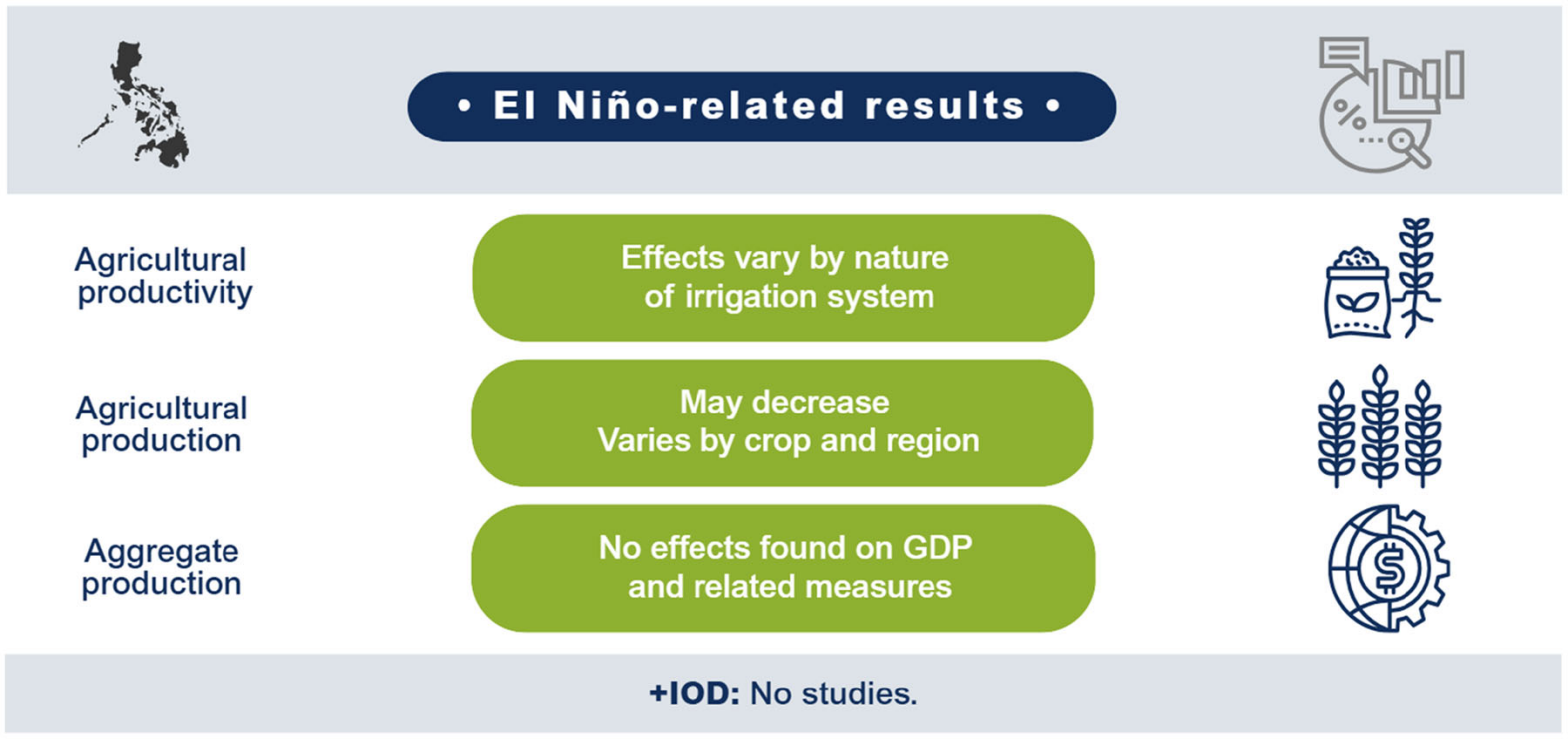
Main findings for the Philippines.

#### 4.4.3 The Philippines

##### 4.4.3.1 Context

El Niño events typically are associated with drier conditions and less abundant rainfall (Villafuerte et al. 2014), which increase the risk of drought (Hilario et al. 2009). The effects of an El Niño event on monthly rainfall depend on factors such as the specific region and the El Niño's intensity: strong El Niño events have short but intense effects with a stark decrease in rainfall, which last for a few months, while moderate events have milder effects on rainfall, which last longer (de los Reyes and David 2006) (Figure 14).

##### 4.4.3.2 Summary of the Evidence

We included 15 studies investigating the effects of El Niño in the Philippines for a total of 66 coefficients. We found no studies on +IOD in the Philippines that met our review's inclusion criteria.

##### 4.4.3.3 Findings on the Effects of El Niño in the Philippines

Twelve of the 15 studies employed inferential analysis techniques such as regression (Abdolrahimi 2016; Arcenas 2018; Bekkering 2017; Bertrand et al. 2020; Cashin et al. 2015; Cruz and Canlas 2004; Dait 2022; Datt and Hoogeveen 2003; Ismail and Chan 2020; Reyes et al. 2010; Roberts et al. 2009; Soria and Preciados 2018), two studies used correlational analysis (Carvajal et al. 2018; David 2023), and one study performed both correlational and regression analyses (Laosuthi and Selover 2007).

Almost all studies (14 out of 15) focused on economic outcomes such as production, productivity, investments, and prices. One study looked at the effects of El Niño on vector‐borne diseases (Carvajal et al. 2018).

Seven studies reported the effects of El Niño on production outcomes (Abdolrahimi 2016; Cruz and Canlas 2004; Ismail and Chan 2020; Reyes et al. 2010; Roberts et al. 2009; Soria and Preciados 2018; Stuecker et al. 2018). Most studies found a negative effect (*g* = −0.22) but results varied by type of irrigation, region, and crop.

Abdolrahimi (2016) reported negative, albeit very small and not significant effects on rice and maize production between 1970 and 2005. Bertrand et al. (2020) used the analysis of variance method to compare mean aquaculture production anomalies and found no systematic differences in production between different ENSO categories or El Niño event types, although La Niña and extreme El Niño events often produced shocks in the aquaculture sector.

The effects on production may vary significantly by region, as shown by Reyes et al. (2010) in a study assessing the effects on rice production across 12 regions. In a similar vein, Cruz and Canlas (2004) explored how effects on production change by crop and found that while rice, corn, and sugarcane production may increase, coconut production does not systematically change with El Niño events.

Roberts et al. (2009) conducted a study in the province of Luzon and found El Niño was associated with decreased rice production; however, rainfed rice was more sensitive compared to irrigated rice, as the latter is less reliant on rainfall. Inconsistent with the latter study, Stuecker et al.(2018) found no difference in the effects on production between rainfed and irrigated rice, as they are both negatively correlated with Niño events.

Three studies reported the effects of El Niño on agricultural productivity. Stuecker et al. (2018) reported a decrease in rice productivity and found a difference in the effects between rainfed and irrigated rice, with larger effects on the former and mild effects on the latter.

The other two studies assessing the effects on productivity reported contrasting effects on rice yields. A correlational study (David 2023) from the region of Pampanga found a positive association with El Niño between 2009 and 2018, implying that rice yields in the region were larger under El Niño conditions. On the other hand, for Luzon, Roberts et al. (2009) reported a decline in rice productivity during El Niño events between 1970 and 2005 but results were statistically significant only for rainfed ecosystems.

We included three studies on the effects of El Niño on aggregated production in the Philippines, none of which found significant effects. Laosuthi and Selover (2007) reported non‐significant changes in GDP growth during El Niño conditions. Dait (2022) looked at effects on the value added of the agricultural output^21^ between 1982 and 2010, while Cashin et al. (2015) employed a general vector auto‐regressive model to explore the association between El Niño and the country's real GDP. Although the direction of the effects in both studies was inconsistent, none of the associations were statistically significant, suggesting that aggregated production does not vary with El Niño.

Three studies provided evidence on the effects of El Niño on prices. Laosuthi and Selover (2007) analysed data from 1950 to 2000 and reported a decrease in the Consumer Prices Index due to El Niño events, but the effect was not statistically significant. The other two studies found a positive association; however, only one found statistically significant results. Using data from 1979 to 2013, Cashin et al. (2015) found no significant association between El Niño and inflation. Conversely, Arcenas (2018) used a random effects regression model and found that El Niño was associated with higher inflation rates between 1996 and 2014.

As for the other economic outcomes, the evidence suggested potential adverse effects of El Niño on consumption (Datt and Hoogeveen 2003) and varying effects on investments over time (Bekkering 2017).

We included one study assessing the effects on vector‐borne diseases. Carvajal et al. (2018) found that El Niño was correlated with a small reduction in dengue incidence between 2009 and 2013.

**Figure 15:**
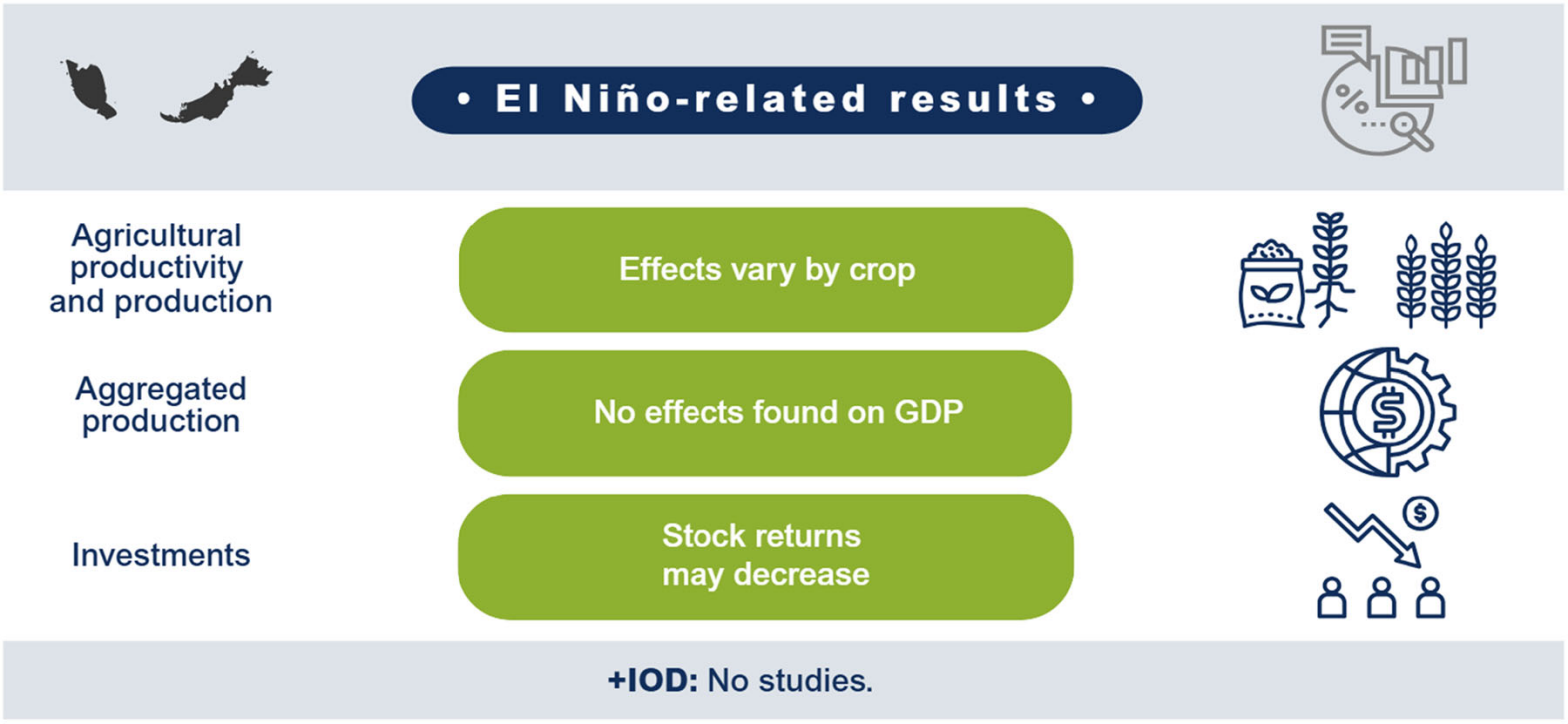
Main findings for Malaysia.

#### 4.4.4 Malaysia

##### 4.4.4.1 Context

Malaysia usually experiences a reduction in rainfall during El Niño events (Tangang et al. 2017). Effects on rainfall are particularly accentuated in the west coastal region of the Malaysian Peninsula (Wong et al. 2009) (Figure 15).

##### 4.4.4.2 Summary of the Evidence

We included 14 studies from Malaysia for a total of 43 estimates. All studies reported on the effects of El Niño, with 11 studies focusing on economic outcomes (Abdolrahimi 2016; Bekkering 2017; Cashin et al. 2015; Khor et al. 2021; Laosuthi and Selover 2007; Rahman et al. 2013; Tawang et al. 2002; Tawang and Tengku 2019; Wen and Sidik 2011; Yang 2017) and three studies providing evidence on health (Che Him 2015; Impoinvil et al. 2013; Yip et al. 2022). We did not identify eligible studies focusing on the effects of +IOD in Malaysia.

Evidence from Malaysia indicated that the effects of El Niño on agricultural production and productivity may vary by type of crop. Specifically, studies suggested that palm oil production and yields are particularly damaged by rainfall reduction and droughts induced by El Niño events, as oil palm trees are a rainfall‐sensitive crop.

The evidence also pointed to decreased investments due to El Niño and no systematic effect on Malaysia's GDP.

Regarding health outcomes, the effects of El Niño on vector‐borne diseases in general may vary by region and disease type. Two studies pointed to an increase in dengue incidence rates in the central region and the Malaysian Peninsula, while another found a decrease in Japanese encephalitis in Sarawak.

We did not find any studies reporting on the effects of +IOD on any of the considered outcomes.

##### 4.4.4.3 Findings on the Effects of El Niño in Malaysia

Three studies provided evidence on the effects of El Niño on the production of various crops and on fishing. Wen and Sidik (2011) reported an increase in fish landing associated with El Niño conditions. The authors used data from 2005 to 2009 to explore the correlation between palm oil production and El Niño conditions and reported a negative association, implying a reduction in production. Similarly, Rahman et al. (2013) used multiple regression analysis to analyse data from 1990 to 2012 and found statistically significant and negative effects, suggesting a decrease in crude palm oil production. The other crop assessed was rice, for which Abdolrahimi (2016) reported no effects on production using data from 1962 to 2009.

We identified three studies on the effects of El Niño on agricultural productivity in Malaysia. Khor et al. (2021) consistently reported negative effects on palm oil yields across different El Niño events that took place between 1987 and 2019. Similarly, Tawang et al. (2002) found negative effects of El Niño conditions on palm oil yield using regression analysis on countrywide data. They also found a negative effect on rubber yields but positive effects on rice yields.

Tawang and Tengku (2019) used linear regression analysis to explore the effects on various crop yields in the Kedah‐Perlis region. The authors did not find evidence of an effect on palm oil yields at this regional level but found negative effects on rubber and rice yields and positive effects on tobacco yields. These contrasting results suggest that the effects of El Niño on agricultural productivity in Malaysia may vary by crop and region.

Three studies providing evidence on the effect of El Niño on prices were included in the review. Rahman et al. (2013) reported evidence of an increase in crude palm oil prices. Cashin et al. (2015) and Laosuthi and Selover (2007) investigated the effects on general inflation and did not find evidence of a change in consumer prices.

Two of the included studies from Malaysia provided evidence on aggregated production and reported no effects of El Niño on Malaysia's GDP (Cashin et al. 2015; Laosuthi and Selover 2007).

Three studies provided evidence on the effects of El Niño on investments. Mahmudul et al. used data between 2003 and 2016 to assess the effects on returns on assets and stocks. The study indicated large negative effects on firms' financial performance. A study by Bekkering (2017) explored whether the effects on stock returns vary with time, and found that investments have a fluctuating pattern over the first year after an El Niño event, with a significant decrease at 3 months but no effects afterwards. Finally, Yang (2017) reported a negative effect of El Niño on stock returns in the Malaysian markets, though they found a positive effect during winter months.

Three studies reported on vector‐borne disease, of which two provided evidence on dengue incidence and one reported on Japanese encephalitis. A study by Yip et al. (2022) reported a positive association between El Niño conditions and dengue incidence rates in the central region, implying an increase in dengue incidence between 2013 and 2019. However, Che Him (2015) employed regression analysis to analyse country‐wide data between 2001 and 2016 and found that El Niño conditions had no effect on dengue incidence. Lastly, a study by Impoinvil et al. (2013) investigated the effects on Japanese encephalitis between 1997 and 2006 and found a decreased risk of the disease associated with El Niño conditions (delayed by 6 months).

**Figure 16:**
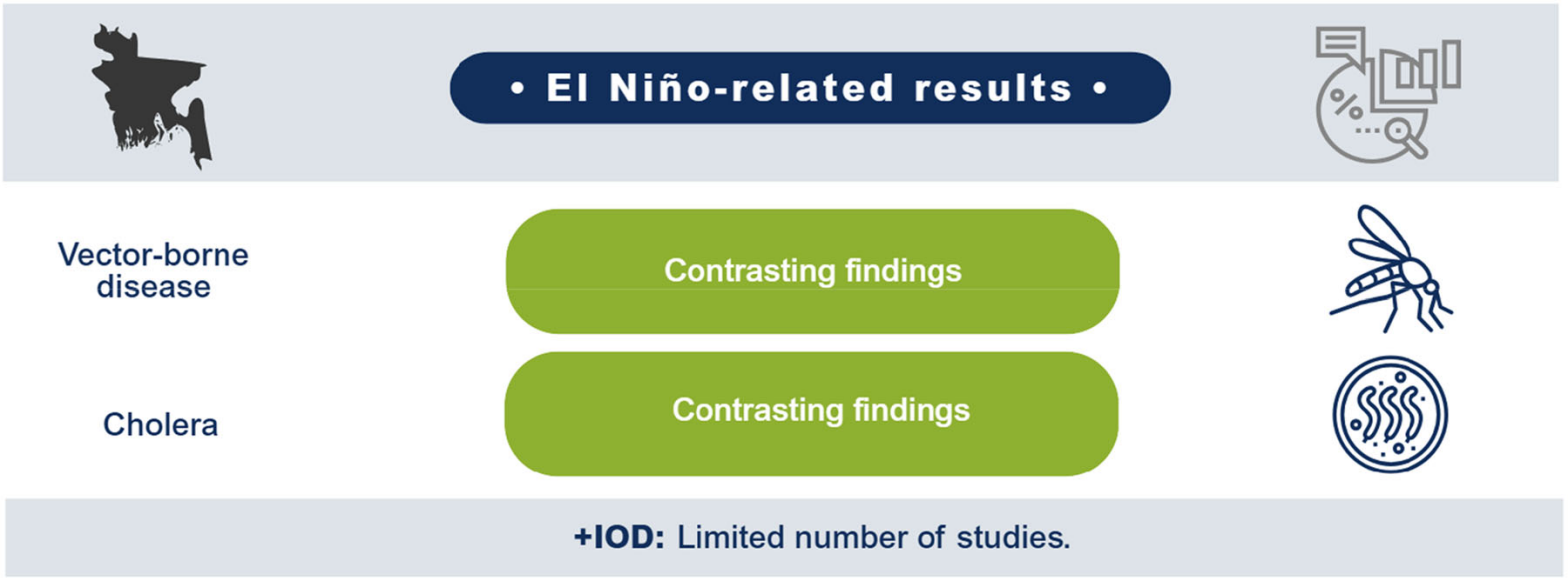
Main findings for Bangladesh.

#### 4.4.5 Bangladesh

##### 4.4.5.1 Context

During El Niño events, Bangladesh typically receives less rainfall (Islam and Parvez 2020). The negative anomaly of rainfall during El Niño events is more prominent in the western (including north and southwestern) part of the country (Wahiduzzaman and Luo 2021). Conversely, during +IOD events, rainfall increases, particularly in the western region and its two divisions (Ahmed et al. 2017) (Figure 16).

##### 4.4.5.2 Summary of the Evidence

We included 11 studies reporting evidence of the effects of El Niño or +IOD on health and economic outcomes in Bangladesh.

Evidence from Bangladesh suggested that the effects of El Niño on agricultural production might be negative; however, the number of studies was too small to corroborate this conclusion, and new studies may produce a different result.

El Niño and +IOD seem to be associated with an increase in cholera incidence in Dhaka and Matlab, which contrasts with the reduced incidence observed at a national level.

Two studies found that El Niño is associated with increased vector‐borne disease incidence, which contrasted with another study that used a more robust research design and found no effect.

##### 4.4.5.3 Findings on the Effects of El Niño in Bangladesh

Ten studies provided evidence on the effects of El Niño. Seven of these reported on health outcomes (Azad and Lio 2014; Banu et al. 2015; Cash et al. 2014; Daisy et al. 2020; Perez‐Saez et al. 2016; Rodó et al. 2002; Sharmin et al. 2016), while three studies reported on economic outcomes (Abdolrahimi 2016; Bertrand et al. 2020; Ghose et al. 2021).

Three studies explored the effect of El Niño on cholera (Daisy et al. 2020; Perez‐Saez et al. 2016; Rodó et al. 2002), while one study provided information on both cholera and shigellosis (Cash et al. 2014). While two studies reported negative effects, implying an increase in the cholera incidence, one study (Rodó et al. 2002, which explored the association between El Niño and cholera incidence between 1980 and 2001 at the country level) reported a reduction in cholera incidence during El Niño conditions.

Cash et al. (2014) used correlational analysis to explore whether the effects vary by region and found an increase in shigellosis and cholera incidence in both Dhaka and Matlab. These findings are corroborated by Perez‐Saez et al. (2016), who reported an increase in cholera incidence in Dhaka associated with El Niño between 1995 and 2008. Daisy et al. (2020) also reported a higher incidence of cholera in Dhaka associated with El Niño.

Three studies reported on the effects of El Niño on vector‐borne diseases. Banu et al. (2015), a study we rated as having a low risk of bias, used regression analysis to explore national‐level data for 2000–2012 and found an increase in the dengue incidence associated with El Niño conditions. Azad and Lio (2014) used data between 1985 and 2009 and reported no effect of El Niño on dengue fever cases, but an increase in malaria fever cases. Sharmin et al. (2016) explored potential time variations of the observed effects but also found no influence on the incidence of dengue during El Niño events, nor 1 month after the event.

Three studies reported on the effects of El Niño on economic outcomes. Two of these provided evidence of effects on production, and one provided information on productivity. Abdolrahimi (2016) provided regression evidence on the association between El Niño and rice and wheat production. They found a decrease in rice and no effect for wheat. Bertrand et al. (2020) found no large variation in aquacultural production between 1950 and 2016, although certain ENSO conditions (La Niña and extreme El Niño) were more often associated with larger variations in production. Ghose et al. (2021) used data between 1980 and 2017 to assess the association between El Niño and rice productivity in Rangpur, Dinajpur, Dhaka, Tangail, Mymensingh, Barisal, and Rajshahi, but found no major changes.

##### 4.4.5.4 Findings on the Effects of +IOD in Bangladesh

Three studies provided evidence on the effects of +IOD. Of these, two studies reported on health outcomes and one study report on productivity.

The study by Banu et al. (2015) assessed the effects on dengue between 2000 and 2012. The authors found an increase in dengue incidence associated with +IOD events. Hashizume et al. (2011) explored the effects on cholera incidence between 1993 and 2007 and reported that impacts vary by district. The authors found increased cholera incidence in Dhaka and Matlab during the first 3 months of +IOD conditions, but a decrease in incidence in Dhaka 4 to 7 months after +IOD conditions. The authors called for further research to understand the reasons for the detected geographical variation.

Ghose et al. (2021) analysed the impact of +IOD on productivity of Boro rice in Khulna, Jashore, and Faridpur. The study reported no effects on yields from 1980 to 2017.

**Figure 17:**
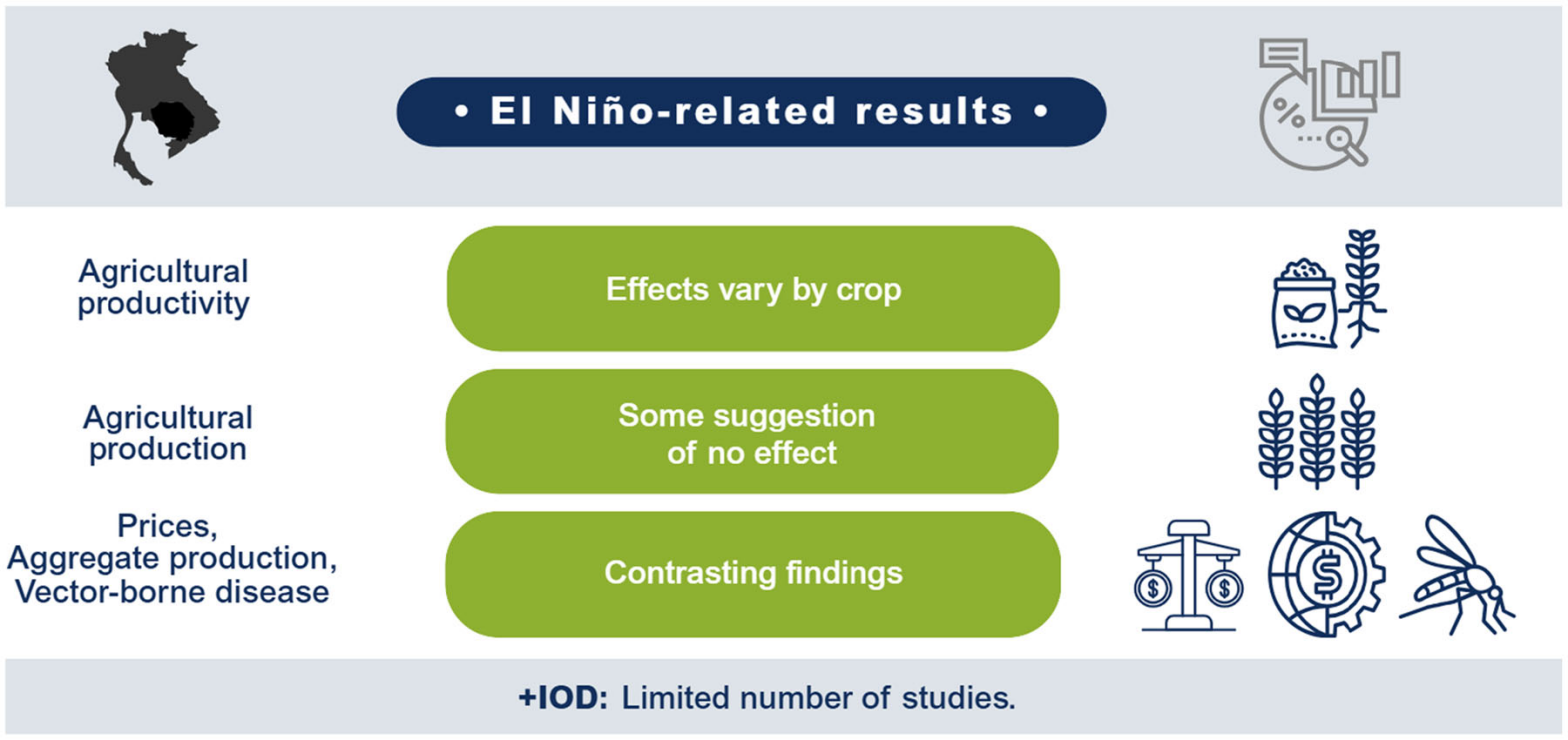
Main findings for the Lower Mekong River Basin region.

#### 4.4.6 Lower Mekong River Basin Region

##### 4.4.6.1 Context

El Niño is typically associated with less abundant rainfall across the four countries in this region (Kirtphaiboon et al. 2014). In Vietnam, the effects are stronger in the southern region and weaker in the northern regions (Duc et al. 2018). The effects of El Niño in Thailand can induce droughts (Power et al. 2020), although effects are more pronounced in the southern regions and weaker in the northern regions (Supriyasilp and Pongput 2021). Likewise, El Niño events are associated with lower rainfall and associated risk of drought in Cambodia (Chhinh and Millington 2015) and Laos (Kim et al. 2020) (Figure 17).

##### 4.4.6.2 Summary of the Evidence

We included 13 studies reporting the effects of El Niño and +IOD in the Lower Mekong River Basin region. Ten studies provided evidence on effects in Thailand (Abdolrahimi 2016; Azad and Lio 2014; Bekkering 2017; Cashin et al. 2015; Laosuthi and Selover 2007; Limsakul 2019; Pipitpukdee et al. 2020a and 2020b; Tiensuwan and O'Brien 2013; Tipayamongkholgul et al. 2009), four studies provided evidence on Vietnam (Abdolrahimi 2016; Bertrand et al. 2020; Kien et al. 2010; Nguyen et al. 2020); two studies examined Cambodia (Abdolrahimi 2016; Bertrand et al. 2020); one examined Laos (Abdolrahimi 2016), and one provided aggregated effects from multiple countries (Ismail and Chan 2020). Twelve of these studies explored the effects of El Niño, while one also provided information on the impact of +IOD (Nguyen et al. 2020).

The included studies provided evidence on various economic and health outcomes: production, productivity, aggregated production, investments, prices, vector‐borne diseases, and enteric diseases (other than cholera).

Most studies in the lower Mekong River Basin region were conducted in Thailand.

El Niño may decrease agricultural productivity, with one study finding reduced cassava yields in Thailand, and other studies from the same country indicating reduced rice yields and no effects on sugarcane yields.

We found no consistent evidence of El Niño negatively affecting agricultural production. One study suggested that rice production in Thailand might decrease due to droughts and less rainfall during El Niño, but this was contradicted by another study. Additionally, three other studies found no effects on aquaculture, sugarcane, maize, or cassava production.

The evidence highlighted heterogeneous findings on aggregate production, prices, and vector‐borne diseases.

We did not find enough evidence on the effects of +IOD in the region.

##### 4.4.6.3 Findings on the Effects of El Niño in the Lower Mekong River Basin Region

Five studies explored the effects of El Niño on production outcomes in at least one of the four countries in the Lower Mekong River Basin region (Abdolrahimi 2016; Bertrand et al. 2020; Limsakul 2019; Pipitpukdee et al. 2020a, 2020b), with one study finding a large negative effect on rice production in Thailand (*g* = −0.86; Limsakul 2019). This contrasts with Abdolrahimi (2016), who found no effect. The rest of the results suggested no effect of El Niño on production for this region (outcomes assessed were aquaculture, sugarcane, maize, and cassava production).

All evidence on productivity, aggregated production, investments, and prices came from Thailand. Limsakul (2019) found that El Niño events in Thailand are associated with decreased rice crop production but had no effect on yields between 1961 and 2016; while Pipitpukdee et al. (2020a, 2020b) reported negative effects on cassava yields and no effects on sugarcane yields in Thailand between 1989 and 2016.

Two studies reported the effects of El Niño on Thailand's aggregated production. Specifically, Cashin et al. (2015) found a statistically significant increase in GDP between 1979 and 2013, whilst Laosuthi and Selover (2007) found no effect on GDP between 1950 and 2000.

Two studies assessed the effects of El Niño on inflation in Thailand. Cashin et al. (2015) found no effect, while Laosuthi and Selover (2007) suggested some effect on inflation in the short term.

One study investigated the effects of El Niño on investments in Thailand. Bekkering (2017) reported negative effects on stock returns but positive effects on trading volumes for the period 1997–2017. The author explored the temporal distribution of effects and found that the largest occurred 3 months after the onset of El Niño.

Five studies reported the effects of El Niño on health outcomes (Azad and Lio 2014; Kien et al. 2010; Nguyen et al. 2020; Tiensuwan and O'Brien 2013; Tipayamongkholgul et al. 2009). Of these five studies, four provided evidence on vector‐borne diseases in Thailand and Vietnam (Nguyen et al. 2020; Tiensuwan and O'Brien 2013; Tipayamongkholgul et al. 2009), but the effects varied widely across studies.

Three studies assessed the effects in Thailand and reported contrasting findings, which might be explained by the different time lags employed in the analysis. Tiensuwan and O'Brien (2013) used a 0‐time lag to evaluate the effects of El Niño between 2004 and 2009 and found a decrease in dengue incidence. Conversely, Tipayamongkholgul et al. (2009) measured the effects on 13 provinces between 1996 and 2004 and found an increase in dengue cases 6 months after the occurrence of the El Niño event.

However, Azad and Lio (2014) found an increased incidence of dengue fever in Thailand associated with El Niño conditions using data from 1985 to 2009. For Vietnam, Nguyen et al. (2020) reported negative effects of El Niño on vector‐borne diseases. Using panel data covering the two provinces of Da Nang and Khanh Hoa from 2014 to 2018, the authors found that El Niño increased the incidence of dengue in both provinces.

One study focused on enteric diseases in South Vietnam using data between 1979 and 2003 (Kien et al. 2010). The authors reported an increase in the rate of diarrhoeal diseases and dysentery associated with El Niño events.

##### 4.4.6.4 Findings on the Effects of +IOD in the Lower Mekong River Basin Region

One study explored the effects of +IOD in Vietnam. Using panel data covering the two provinces of Da Nang and Khanh Hoa between 2014 and 2018, Nguyen et al. (2020) found that +IOD increased the incidence of dengue in both provinces.

**Figure 18:**
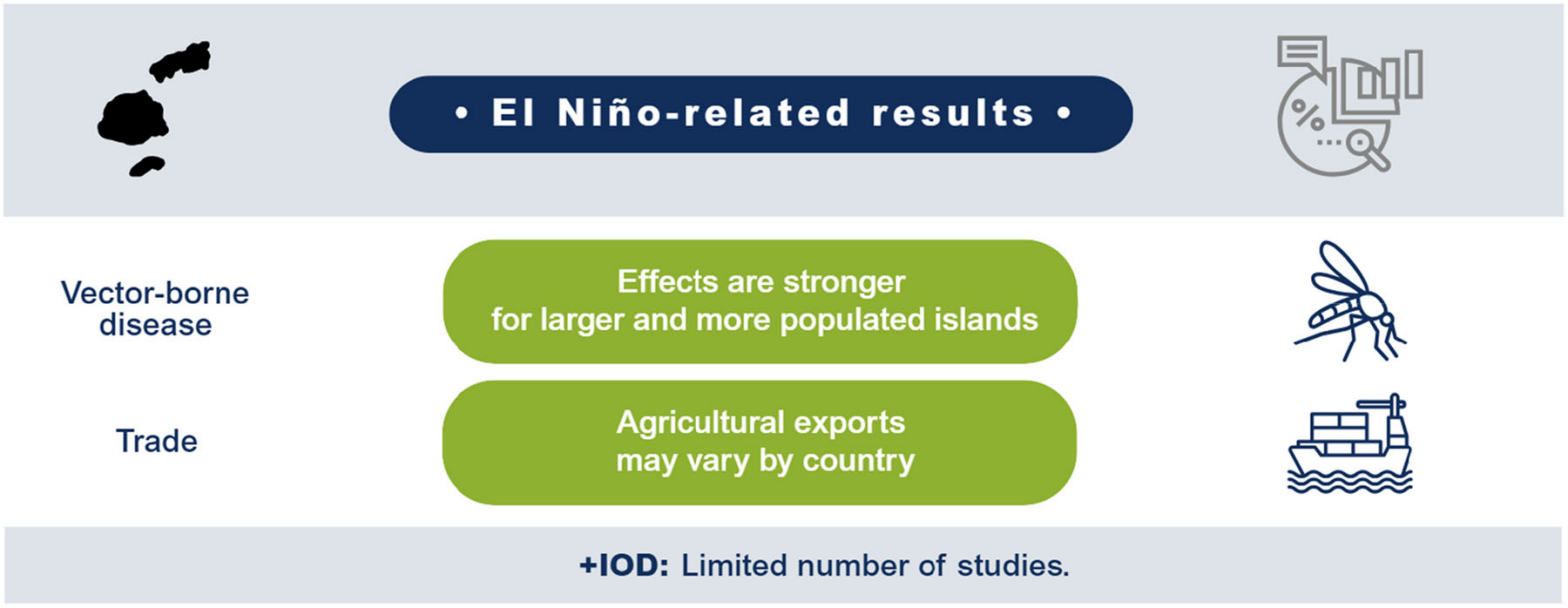
Main findings for Oceania.

#### 4.4.7 Oceania

##### 4.4.7.1 Context

The effects of El Niño across the Pacific islands are heterogeneous. Overall, El Niño events are associated with a reduction in rainfall, which can lead to severe droughts (Annamalai et al. 2015; Kuleshov et al. 2014) (Figure 18).

##### 4.4.7.2 Summary of the Evidence

We included six studies focusing on Oceanian countries (Andhikaputra et al. 2023; Duncan 2008; Hales et al. 1999; Hughes et al. 2010; Kim et al. 2016; Llewellyn 2010). Two of these assessed the effects of El Niño in Papua New Guinea (Duncan 2008; Kim et al. 2016), one reported on Samoa (Hughes et al. 2010), and another examined the Solomon Islands (Andhikaputra et al. 2023). The two remaining studies report on impacts on multiple Oceanian countries. We identified one study on the effect of +IOD in Oceanian countries.

Evidence from Oceania suggested that El Niño may increase dengue incidence in larger and more populated islands where the disease is endemic. The effects of El Niño varied depending on rainfall intensity and weather temperature in each country.

Two studies examined the relationship between El Niño and trade. In Papua New Guinea, effects on exports varied by type of crop. In Samoa, overall agricultural exports did not change but agricultural imports increased.

One study reported on the effects of both El Niño and +IOD on pneumonia cases for children under 5 in Papua New Guinea between 1997 and 2006. The authors found reduced incidence associated with +IOD and increased incidence associated with El Niño.

We did not find enough evidence on the effects of El Niño and +IOD on other economic and health outcomes in the Pacific Islands.

##### 4.4.7.3 Findings on the Effects of El Niño in Oceania

Four of the six studies reported on health outcomes. Specifically, two focused on dengue‐like disease incidence. Andhikaputra et al. (2023) used data on the Solomon Islands between 2015 and 2018 and found that dengue incidence decreased with El Niño in the two analysed provinces of Guadalcanal and Western Solomon. The authors argued that weather temperature and rainfall intensity accounted for the observed change and for the variation across provinces.

However, Hales et al. (1999) used panel data of Oceanian islands between 1973 and 1994 and reported that El Niño increased dengue cases in five countries (with highest effects recorded in Tokelau, followed by Western Samoa, Fiji, American Samoa, and Tonga). No association between El Niño and reported dengue fever cases was observed in the other countries and territories (Nauru, Vanuatu, Wallis, New Caledonia, Kiribati, Niue, Tuvalu, and the Cook Islands). The authors concluded that El Niño may trigger an increase in dengue fever transmission in larger, more populated islands where the disease is endemic. There was also evidence of propagation of infection from larger islands to smaller neighbours—although the latter is independent of interannual climate variations and appears to be driven by population density and travel.

The other two studies assessed the effects of El Niño on enteric and respiratory diseases. Kim et al. (2016) reported on the effects of both El Niño and +IOD on pneumonia cases for children under 5 years in Papua New Guinea between 1997 and 2006. The authors found that El Niño was associated with an increase in pneumonia cases over the five considered areas (Port Moresby and Central Province, Daru, Eastern Highland Province, Madang Province, and East Sepik Province), representing the southern coastal, highland, and northern coastal regions. The effects might be explained by heightened susceptibility to pneumonia related to food shortage during dry seasons (Kim et al. 2016).

Llewellyn (2010) used a panel data set including Fiji, Western Samoa, Vanuatu, and Kiribati, amongst other countries outside the scope of this review, to assess the effects of El Niño on reported cases of ciguatera poisoning (from eating fish) between 1973 and 1996. The author reported that El Niño may have both positive and negative impacts on ciguatera‐reported cases. Several ENSO indices exhibited robust relationships with the incidence rate, with El Niño 4 SST being the strongest, likely due to geographical proximity.

We identified two studies reporting effects on economic outcomes. Duncan (2008) found that El Niño in Papua New Guinea between 1976 and 2006 is associated with decreased cocoa, copra, and palm oil exports and with higher trade volumes of copra oil and coffee. Hughes et al. (2010) used data from the Food and Agriculture Organisation Corporate Statistical Database to assess the effects of El Niño on trade and agricultural production and yields in Samoa. The findings indicated an increase in agricultural imports, negative effects on taro production and coconut yields, and no effect on banana production and yields, taro yields, and coconut production.

##### 4.4.7.4 Findings on the Effects of +IOD in Oceania

Kim et al. (2016) reported an overall reduction in pneumonia cases for children under 5 years over the southern coastal, highland, and northern coastal regions during +IOD events.

## 5 Discussion

We conducted a systematic review of the effects of El Niño and +IOD on health, economics, food security, conflicts, and migration. The review includes evidence from 89 studies looking at the association of either El Niño or +IOD and economic and health outcomes. We did not find studies assessing the impact on migration and social conflicts in LMICs in the Indo‐Pacific region.

We separated evidence using correlational analysis from studies that used inferential analysis, including regression methods, to explore the relationship between El Niño or +IOD and the outcomes of interest (such as multivariable regression techniques, time series methods, or quasi‐experimental designs). Where we found enough evidence, we used the latter to estimate the average effect of these climate drivers in the region using meta‐analysis.

The findings from the meta‐analyses indicate that El Niño is associated with an average reduction in agricultural production and productivity in Indo‐Pacific countries. The decrease in agricultural production and crop yields is due to warmer and drier climatic conditions which characterise El Niño events in this region. These findings are in line with evidence from other regions and countries not included in this review, such as North and South America (Anderson et al. 2017; Barrios‐Perez et al. 2021; Berlato and Fontana 2001), Africa (Rembold et al. 2016), and China (Tao et al. 2004), and with existing cross‐country analyses (Iizumi et al. 2014; Ubilava and Abdolrahimi 2019).

The meta‐analyses further suggest no consistent evidence of an association between El Niño and prices or incidence of vector‐borne diseases in the Indo‐Pacific. As for the other outcomes (e.g., aggregated production, investments, cholera, and other enteric infections), we did not find enough evidence to meta‐analyse the effects of El Niño for the Indo‐Pacific region. There is also insufficient evidence to assess the overall or average effects of +IOD on the outcomes of interest and to draw firm conclusions for the Indo‐Pacific.

The larger number of included correlational studies allowed us to further explore the cascading effects of +IOD and El Niño. We used correlational coefficients to look at the direction of the associations found in the evidence and explore factors that can explain differences in results from included studies reporting on the same outcome. Overall, the findings from the correlational analysis corroborate the results of the meta analysis.

Specifically, we found that El Niño was associated with decreased production. Furthermore, the analysis of the correlational coefficients shed some light on the effects of +IOD. The median correlation values presented in the analysis suggest +IOD is associated with increased production and a reduction in the incidence of vector‐borne diseases. These findings are in line with some of the broader literature from countries and geographical areas not covered by this review (Atique et al. 2016; Funk and Brown 2009; Xu et al. 2021) and corroborate our theory of change.

Drawing on the narrative analysis, we could identify potential mitigating factors, facilitators, within‐country variation, cascading effects of La Niña, and implications for the United Kingdom. Increased awareness and efforts to combat vector‐borne diseases like malaria and dengue, such as artesunate combination therapy, insecticide‐treated mosquito nets, indoor residual spraying, and long‐lasting insecticide‐treated nets, could reduce the impact of climate drivers on these diseases.

Water management within irrigation schemes for water‐intensive crops like rice, and measures to improve control and management systems, are also in place to mitigate effects on production and productivity. The qualitative analysis indicates that the effects of La Niña are generally opposite to those reported for El Niño, although we did not quantitatively assess the effects of La Niña in this review.

### 5.1 Overall Applicability and Confidence in Individual Study Findings

We assessed author's decisions related to study design, conduct, and reporting and how they affect the confidence we place in the relevant findings of the included studies. We identified risks of bias, which is the possibility that the reported results differ from the actual effects of the climate driver due to study design or reporting limitations. To attribute observed changes to El Niño and +IOD, studies must account for other factors that may affect the outcomes. For example, seasonality, technological improvements, political and economic changes, geographical differences, and climate change also influence economic, health, and social outcomes.

We assessed studies that did not account for these factors as having a high risk of bias. Ignoring potential sources of variation undermines the validity of some of the studies and limits the applicability of their results. A strong study design should account for potential confounding factors while also considering the cyclicity and temporality of climate drivers and outcomes. Additionally, it is essential to assess the non‐linearity of effects and verify key study design assumptions to ensure robust and comparable findings.

We have categorised the evidence by risk of bias (high, low, some concerns) in Online Appendix S1K. Unfortunately, for most outcomes, we were unable to test whether there were differences in the results based on the risk of bias because of insufficient data. The only outcome with sufficient data to test the risk of bias score as a moderator of the average effect was the incidence of vector‐borne diseases. No statistically significant difference was observed in the effects of El Niño based on the risk of bias assessment of studies.

We only included evidence that presented disaggregated results for LMICs from the Indo‐Pacific region; therefore, our results cannot be generalised to other regions. Expanding the geographical scope to effects on the eastern and western coasts of both the Pacific and Indian Oceans could help to shed more light on the impacts of El Niño and +IOD. However, this would be challenging in terms of combining teleconnection patterns that bring opposite effects to different coasts of both oceans.

Our aim was to identify the systematic effects of El Niño and +IOD at the regional level for Indo‐Pacific countries. Yet, we found that effects vary largely by climate driver and geographical region. Whenever meta‐analysis results are strong, as the negative effect of El Niño observed (i.e., reduced agricultural production and productivity), we were able to conclude that the overall region should be prepared to prevent or mitigate negative impacts. However, outcomes such as prices and the incidence of vector‐borne diseases lack consistent evidence across the region, so readers may prefer to consult that narrative synthesis of studies at the country level.

For more information about the review's strengths and limitations, refer to Online Appendix S1N. Potential biases in the review process are described in Online Appendix S1M.

### 5.2 Agreements or Disagreements With Other Reviews

In this section, we contrast the main findings of our review on health and economic outcomes with the findings from some reviews identified during our scoping and search periods. The results of this systematic review are fairly aligned with existing systematic reviews on the effects of El Niño on health outcomes. Like Kovats et al. 2003) and Ahmed (2023), we found heterogeneous effects that vary across and within countries.

While the former reviews synthesised evidence with narrative analysis, we were able to perform a meta‐analysis and provide a combination of quantitative and qualitative synthesis. Our meta‐analysis found an overall non‐systematic (statistically non‐significant) small reduction in the incidence of vector‐borne diseases associated with El Niño, consistent with the weak evidence identified by Kovats et al. (2003) in the association between the ENSO cycle and mosquito‐borne disease incidence.

Furthermore, the negative impact found on production and productivity aligns with findings reported by Hirons and Klingaman (2016). Based on scientific literature retrieved from academic databases and grey literature sources, the authors reported high‐level‐confidence evidence indicating a decrease in crop production in Indonesia, as well as reduced crop productivity in the South East Asian Peninsula and Southern Asia during the development phase of El Niño.

Hirons and Klingaman (2016) also explored other sectors for potential socioeconomic impacts of ENSO and found a possible pathway to impact (based on low‐confidence evidence) on migration in Vietnam, Pakistan, and Bangladesh. We could not identify any literature meeting our inclusion criteria to corroborate these findings.

We did not identify any syntheses on the effects of +IOD on socioeconomic outcomes.

## 6 Authors' Conclusions

We attempted to address research questions 1 and 2 regarding the magnitude and direction of effects using meta‐analysis but we did not have enough evidence to do so for each El Niño/+IOD and outcome pair. Where meta‐analysis was not possible, we described the median distribution of estimates and used narrative synthesis. Results are presented in Section 4.3.

We were not able to address research question 3 about differences in results by magnitude and type of climate driver, as the evidence is limited in this regard. However, we summarise the findings of each study and present them, along with the characteristics of the climate driver being evaluated, in Online Appendix S1B.

We addressed research question 4 on factors accounting for the heterogeneity of findings using narrative synthesis. We present results separately for each country and region in Section 4.4 and highlight factors identified in the literature throughout the analysis.

We assessed the confidence we place in the findings of each study by conducting a risk‐of‐bias assessment to address research question 5. The assessment is summarised in Section 4.2.

Our search and screening processes, described in Section 3, helped us to identify the literature gaps highlighted in Section 4. In this section, we provide implications from these findings for future research in response to research question 6.

### 6.1 Implications for Practice and Policy

Given the variability in frequency and magnitude of El Niño events, where no two events are exactly alike, affected countries could prioritise proactive measures to address socioeconomic challenges exacerbated by these phenomena. General strategies for adapting to climate variability can also help to mitigate the unpredictable effects of El Niño and +IOD and enhance countries' resilience to the effects of these climate drivers.

At the same time, the heterogeneous nature of the effects observed underscores the necessity for nuanced and context‐specific policy interventions. By acknowledging the diverse effects of El Niño and +IOD events within and between countries, the review emphasises the importance of flexible and adaptive policy responses. Policy responses should be context‐specific and tailored to the impact of El Niño and +IOD on specific regions and crops and consider the contextual socio‐economic and cultural peculiarities. There is a need for comprehensive tailored plans aimed at supporting coping strategies through both infrastructure improvements and capacity building.

For the agricultural sector, in particular, negative effects on production and productivity highlight the urgent need for coping strategies. These strategies could focus on providing essential infrastructure such as water supplies and irrigation systems. Additionally, capacity building amongst farmers is crucial; this involves training on farm practices such as moisture management, soil, and water conservation and the efficient use of inputs to mitigate crop losses. Implementation of crop insurance programmes alongside these strategies can help to mitigate unavoidable risks. Mitigation plans are particularly needed for rainfed crops, given the association between El Niño and +IOD and precipitation variability.

Health systems should be prepared to mitigate any possible increase in the incidence of infectious disease. In general, prevention and coping strategies should prioritise strengthening health infrastructure and enhancing capacity building amongst healthcare providers. Equipping healthcare professionals with the necessary tools to mitigate impacts, and bolstering health system resilience against risks, such as infectious diseases and heat‐related illnesses, is essential particularly for vulnerable populations.

Ensure that effective coordination and communication strategies are in place to swiftly disseminate advisories and guidelines. These measures facilitate prompt adjustments to interventions and enhance the overall effectiveness of preparedness measures at both national and regional levels.

Community engagement strategies and education could be used to help adapt to and mitigate the effects of climate drivers. Identifying and leveraging local solutions, as well as raising public awareness about these event cycles can significantly strengthen community resilience.

Finally, prediction systems of teleconnection patterns could be improved to provide accurate forecasting of weather anomalies for governments, health workers, farmers, vulnerable populations, and the public. Farmers, for instance, can mitigate adverse impacts by adopting strategies such as planting drought‐resistant crops during El Niño events and increasing their use of seeds, organic fertilisers, pest control, and irrigation in anticipation of drier seasons. Similarly, communication campaigns aimed at promoting public behaviours can help to prevent the spread of vector‐borne diseases and other health‐related risks.

### 6.2 Implications for Research

The following infographic presents the implications for future research based on our findings in terms of evidence gaps and the quality assessment of included studies.

**Table:** 

RESEARCH IMPLICATIONS			
Finding	Evidence gaps	No evidence on migration, conflict, food security, and nutrition Limited and high‐risk‐of‐bias evidence on economic, health, and food security outcomes (agriculture) No studies examining the combined effect of El Niño and +IOD Limited evidence on differential effects by magnitude of the climate driver No qualitative effectiveness studies found	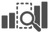
Implication	More research	More research is needed on the socioeconomic effects in Indo‐Pacific LMICs particularly: Migration and social conflict, food security, and nutrition +IOD cascading effects and combined effects with El Niño El Niño Modoki	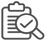
Finding	High‐risk‐of‐bias evidence	Identified studies are mostly observational Study designs do not meet the minimum criteria for obtaining unbiased estimates Studies explore the effect of El Niño and +IOD without considering the functional form of effects No sensitivity analysis or robustness checks	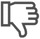
Implication	Minimum criteria	Identify and control for potential confounding factors (contextual and geographic aspects, technological improvement) Consider the cyclicity and temporality of climate drivers and outcomes Consider non‐linearity of the effects Verify study design assumptions	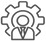
Finding	Other unexplored factors	The role of preventive and mitigation strategies, infrastructure, and response capacity Differences in effects based on crop irrigation type Standardised indices and metrics for capturing El Niño and +IOD intensity How does global warming affect the analysed outcomes through changes in the intensity and length of El Niño and +IOD events Future studies may consider a Bayesian approach, which would also offer opportunities for future synthesis of such models	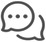
Implication	Research opportunities	What is the combined effect of El Niño and +IOD on socioeconomic outcomes? What are the effects on vulnerable populations? How do effects vary with respect to stronger or weaker events? Develop and use consistent, standardised, and well‐defined climate indices.	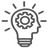

## Author Contributions

A.F., M.D.A.‐L., T.K., M.B., S.S., A.T., P.B., L.T., J.S., and Z.P. are the core team for this review. A.F. is an evaluation specialist with extensive synthesis experience. M.D.A.‐L. is a research associate with experience in development economics. T.K. is a senior research associate with experience in rapid synthesis methods. M.B. is a research assistant. S.S. is a systematic review and quantitative methods expert, with over a decade of experience in designing, managing, and analysing quantitative research, including meta‐analyses. A.T. is a deputy director providing technical leadership of the project. P.B. is a biometeorologist and the principal scientist at the Natural Resources Institute of the University of Greenwich. L.T. is a senior lecturer at the School of Accounting, Finance, and Economics at the University of Greenwich. J.S. is a professor of international politics and climate change at the University of Leeds. Z.P. is an information specialist. Below is a detailed breakdown of their contributions to this report.

- Content: A.F., M.D.A.‐L., T.K. and M.B. developed the content of the review, with inputs and quality assurance from AT.
- Information retrieval: Z.P. developed the initial search strings with input from T.K. and quality assurance from A.T.
- Screening: T.K., M.D.A.‐L., A.F., M.B., and A.T. conducted screenings of academic and grey literature and snowballing. T.K. and Z.P. conducted backwards and forwards citation tracking.
- Methods: T.K., M.D.A.‐L., A.,F and Z.P. drafted the review methods, with input and support from A.T. and S.S.
- Statistical analysis: S.S. oversaw the statistical analysis by M.D.A.‐L., A.F., and T.K., who independently extracted effects data in duplicate, with quality assurance from S.S.
- Risk of bias appraisal: M.D.A.‐L., T.K., A.F., M.B., and A.T. independently assessed the risk of bias of included studies in duplicate, with quality assurance from S.S.
- Narrative synthesis: A.F., M.D.A.‐L., M.B., and T.K., with quality assurance from A.T., S.S., P.B., L.T., and J.S.
- Review of background and theory of change: A.F., M.D.A.‐L., T.K., M.B., and A.T. with quality assurance from P.B., L.T., and J.S.

## Conflicts of Interest

The authors declare no conflicts of interest.

## Preliminary Timeframe

The search was developed and executed in January 2024. The protocol was developed from October 2023 until it was published in April 2024. The final report is due to be completed and published in October 2024.

## Plans for Updating This Review

We plan to update the review as new evidence becomes available and funding permitting.

## Differences Between Protocol and Review

Several deviations are related to the analysis, risk of bias, and data extraction procedures.

We did not test the presence of publication bias even in outcomes with 10 or more studies because our sample included dependent effect size estimates which has been proved to inflate the likelihood of detecting selective reporting (Rodgers and Pustejovsky 2021). Further, we were constrained in the resources that would have been needed to adopt an exploratory approach and report multiple tests along with sensitivity analysis to derive confident conclusions about publication bias.

While the protocol presents two separate risk‐of‐bias tools—one to appraise observational designs and another for quasi‐experimental methods—we ended up only using the former to avoid penalising the two studies that used panel methods and instrumental variable estimation.

With regard to data extraction, the protocol specified that two team members would independently extract the data from each study. Due to time limitations, this was the case for quantitative data for effects sizes for meta‐analysis and median analysis of correlation coefficients, as well as for risk‐of‐bias assessments. However, data used for descriptive analysis were extracted by one team member, with a second reviewer spot‐checking and providing guidance if necessary. Also, the following changes were made to the data extraction form:

- The morbidity and mortality outcome category and its definition were revised by removing morbidity, as this overlapped with disease‐related categories. Also, we clarified that enteric disease outcomes would not include cholera.
- The following variables were removed from the quantitative extraction form as they were not relevant for the type of studies that were included: evaluation period (in months), slope coefficient, data points before and data points after.
- The following variables were added to capture the nuances of included study designs: teleconnection lag and El Niño/+IOD specific results (to distinguish between direct measures such as El Niño years and indices).
- The following variables were added to collect qualitative information from the included studies: mitigating factors, facilitators, other relevant information (La Niña/UK), and policy implications and specific region analysis if not reported in quantitative analysis (this was particularly important, as no qualitative methods that met the criteria have been identified).
- The risk of bias tool was adapted so that it can incorporate both quasi‐experimental, regression, and correlation analysis designs.

## Supporting information

Supporting information.

## Data Availability

The data that support the findings of this study are available from the corresponding author upon reasonable request.

## Endnotes

1 Canonical El Niño refers to traditional El Niño events, characterised by warm SST anomalies in the eastern equatorial Pacific (Amaya and Foltz 2014). Conversely, El Niño Modoki is characterised by higher SST in the central tropical Pacific Ocean, and lower SST and negative rainfall anomalies in both the eastern and western Pacific Ocean coasts (Ashok et al. 2007).

2 Evidence suggests that conflicts may be more frequent in the years of El Niño. For instance, Hendrix et al. (2022) reported that fishery disputes in the South China Sea increased during El Niño years due to a reduction in fishing yields. In a similar vein, Hsiang et al. (2011) found that between 1950 and 2004, climate changes induced by El Niño increased the likelihood of civil conflicts globally (even when limiting the sample to exclude African countries).

3 When multiple publications examined the exact same relationship, a primary study was utilised to extract data, while any associated studies were used as a source for additional information (linked publications). When identifying the main study, journal articles were given preference, and if multiple reports or working papers existed, the most recent was chosen.

4 Production outcomes include any measure of the disaggregated economic output such as fish captures, total volume of (agricultural) production or outputs, and (share of) land/area cultivated or harvested.

5 Productivity outcomes include measures of agricultural productivity (yields) and for one study only: fish catch per unit effort.

6 Regions A (165°E–140°W, 10°S–10°N), B (110°W–70°W, 15°S–5°N), and C (125°E–145°E, 10°S−20°N).

7 Two studies that used analysis of variance to estimate the effect of El Niño on production outcomes were not included in the meta‐analysis, as they did not report all the information needed to estimate the SMD (Suratno 2022; Bertrand et al. 2020).

8 Production includes any measure of the disaggregated economic output. This would typically include total volume of production or outputs, and (share of) land/area cultivated or harvested.

9 A negative ‘*r*’ indicates decreased production or productivity during El Niño or +IOD, and a positive ‘*r*’ indicates the opposite.

10 Productivity includes measures of agricultural productivity (yields) or business productivity (fish catch per unit effort).

11 Three studies using regression analysis to estimate the effect of El Niño on productivity outcomes were not included in the meta‐analysis, as they did not report all the information needed to estimate the SMDs (Ghose et al. 2021; Nugroho 2013; Rao et al. 2012).

12 Prices include changes in prices of goods and services, the measures of which are included but not limited to indices of prices, market surveys, and self‐reported prices.

13 One study using regression analysis to estimate the effect of El Niño on prices was not included in the meta‐analysis, as it did not report all the information needed to estimate the SMDs (Selvaraju 2003).

14 Three studies using negative binomial models to estimate the effect of El Niño on vector‐borne disease incidence were not included in the meta‐analysis, as they did not report all the information needed to estimate the SMDs (Andhikaputra et al. 2023; Sharmin et al. 2016; Yip et al. 2022).

15 A positive ‘*r*’ indicates fewer infections associated with El Niño.

16 The study does not clearly indicate whether the association is statistically significant. The text suggests an association, but the results table does not confirm it.

17 Production includes any measure of the disaggregated economic output. This would typically include fish captures, total volume of production or outputs, and (share of) land/area cultivated or harvested. Productivity includes measures of business productivity and agricultural productivity (yields).

18 A negative *r* indicates a reduction in productivity.

19 Rao et al. (2012) did not report enough evidence to compute effect sizes and was not considered in the median effect of El Niño on productivity for India. Using linear regression, the authors reported a reduction in rice yields associated with El Niño conditions.

20 Oceanic Niño Index, Niño 3 SST Anomaly Index, Southern Oscillation Index, El Niño Modoki Index, Dipole mode index, and Trans Niño Index.

21 The study employs the Cobb‐Douglas production function to estimate households' agricultural productivity based on labour, physical capital, investments, human capital and resource endowment.
