# Supplementary material for: Effects of El Niño and the Positive Indian Ocean Dipole (+IOD) on Health, Food Security, Economics, and Conflict in Low‐ and Middle‐Income Countries in the Indo‐Pacific: A Systematic Review
Source: Campbell Syst Rev. 2025 Apr 17;21(2):e70038. doi: 10.1002/cl2.70038 (PMC12004400; doi:10.1002/cl2.70038)
Supplement: Supplementary file 1 — Supporting information. [file CL2-21-e70038-s001.docx]

**Acronyms**

| **ENSO** | El Niño Southern Oscillation |
| --- | --- |
| **+IOD** | Positive Indian Ocean Dipole |
| **L&MICs** | Low- and middle-income countries |
| **SMD**  **SST** | Standardised mean difference  Sea surface temperature |

# Online appendixes

## Appendix A Search strategy: development, summary table, full list of searches

The primary searches were first developed in Scopus and Academic Search Complete. A set of 21 known papers were manually analyzed to harvest free-text terms (found in the title, abstract, or author-supplied keywords fields), and Index terms (applied in Academic Search Complete). For more details including the list of papers used refer to Floridi et al. (2024).

The initial search concepts consisted of free-text terms collated from the known paper harvesting, additional terms known to the review team, terms found in scope notes of various databases (Medline, Embase, Academic Search Complete), and terms found in Wikipedia pages (on the various countries and El Niño). Index terms were gathered from looking up how each known paper was indexed in each database, along with browsing the thesaurus in each source. The searches were iteratively refined by testing known paper retrieval and adding new terms to improve comprehensiveness. Five of the known papers were only found in the grey literature, and so were not used to test database search sensitivity.

The final searches retrieved all known articles available in that source, except in two cases where a given source did not have an abstract for that article. However, in both those cases, the article would be retrieved by a different database search. Therefore, all of the 16 known scholarly articles were retrieved by at least one database. A decision was made to limit to specific Web of Science categories for the Web of Science Core Collection search. This decision was made after thorough testing, including screening a random set of 50 results that would be excluded from application of the limit, which would reduce the yield by 40%.

Due to time restrictions, the searches did not undergo formal peer review by a second information specialist, however it was reviewed by other members of the research team. The results of both the academic and grey literature searches are presented in Appendix Table 1 below. The search strategies for all electronic databases are presented in Appendix Table 2 - Table 13 below.

**Appendix Table 1. Summary of sources searched, known article retrieval, and duplicate removal from the search results.**

| **Database/ Source [keywords]** | **Platform/**  **website** | **Known articles retrieved** | | **Date searched** | **Search Results** | | |
| --- | --- | --- | --- | --- | --- | --- | --- |
|  |  |  |  |  | **Records  found** | **Duplicates removed** | **Unique records** |
| SCOPUS | Elsevier | 15 of 15 | | 2024-01-28 | 3407 | 10 | 3397 |
| Web of Science (WoS) Core Collection | WoS | 13 of 13 | | 2024-01-28 | 2567 | 1336 | 1231 |
| Academic Search Complete | EBSCOhost | 7 of 8 | | 2024-01-28 | 1690 | 1375 | 315 |
| Medline | Ovid | 8 of 8 | | 2024-01-28 | 245 | 202 | 43 |
| CAB Abstracts | Ovid | 6 of 6 | | 2024-01-28 | 1383 | 832 | 551 |
| AGRICOLA | Ovid | 3 of 4 | | 2024-01-28 | 582 | 544 | 38 |
| Global Health | Ovid | 6 of 6 | | 2024-01-28 | 130 | 124 | 6 |
| Biosis Citation Index | WoS | 9 of 9 | | 2024-01-28 | 728 | 608 | 120 |
| Embase | Ovid | 7 of 7 | | 2024-01-28 | 296 | 257 | 39 |
| ProQuest Dissertations | WoS | 0 | | 2024-01-28 | 164 | 1 | 163 |
| Econlit | Ovid | 0 | | 2024-01-28 | 27 | 12 | 15 |
| EBSCO Discovery Service | EBSCOhost | 3 of 3 | | 2024-01-28 | 852 | 594 | 258 |
| **Total for academic databases above** | | | | | **12071** | **5895** | **6176** |
| [IPCC](https://archive.ipcc.ch/search/searchreports.shtml#gsc.tab=0) [Go to "Publications and Data" and screen Reports, Technical papers and Supporting material] | Grey literature | - | | 2024-02-02 | 78 | - | - |
| [Oxfam Library](https://oxfamilibrary.openrepository.com/) [Use same keywords as Google Scholar] | Grey literature | - | | 2024-02-02 | 180 | - | - |
| [Food and Agriculture Organization (FAO)](https://www.fao.org/common-pages/search/en/) ["El Niño effect"  "ENSO effect"  "Indian ocean dipole effect"] | Grey literature | - | | 2024-02-05 | 269 | - | - |
| [International Initiative for Impact Evaluation](https://developmentevidence.3ieimpact.org/) (3ie)  [El Niño] | Grey literature | - | | 2024-01-29 | 6 | - | - |
| [National Bureau of Economic Research (NBER) – Working Papers](https://www.nber.org/) [Home / Research / Working Papers "El Niño"  "Indian Ocean Dipole"] | Grey literature | - | | 2024-01-29 | 58 | - | - |
| [International Fund for Agricultural Development (IFAD)](https://www.ifad.org/en/web/knowledge/research) [Choose Theme = Climate and environment] | Grey literature | - | | 2024-01-29 | 15 | - | - |
| [International Food Policy Research Institute (IFPRI)](https://www.ifpri.org/collections/related/publications/13) [Screen all records related to Climate change] | Grey literature | - | | 2024-01-29 | 37 | - | - |
| [Social Science Research Network](https://www.ssrn.com/index.cfm/en/)[Advanced search: ENSO] | Grey literature | - | | 2024-01-30 | 116 | - | - |
| [USAID Development Experience Clearinghouse (DEC)](https://dec.usaid.gov/dec/content/search.aspx)[Type in the ENSO keyword in a small search box at the top right. Use the Country region filter to only screen studies for populations included in the review.] | Grey literature | - | | 2024-01-29 | 112 | - | - |
| [AgEcon Search](https://ageconsearch.umn.edu/?ln=en) [Use Advanced search, select the option for "Exact" under category "contains" for all search terms: "El Niño" OR "Indian ocean dipole" or ENSO or "southern oscilliation"] | Grey literature | - | | 2024-02-01 | 63 | - | - |
| [Research for Development: FCDO](https://www.gov.uk/research-for-development-outputs)[Search 1: "el nino", search 2: enso] | Grey literature | - | | 2024-01-30 | 23 | - | - |
| [Google Scholar](https://scholar.google.com/) [el nino effect economic] | Grey literature | - | | 2024-01-28 | 310 | - | - |
| [Google Scholar](https://scholar.google.com/) [Indian ocean dipole effect economic] | Grey literature | - | | 2024-02-02 | 50 | - | - |
| [Google Scholar](https://scholar.google.com/) [el nino effect production] | Grey literature | - | | 2024-01-29 | 132 | - | - |
| [Google Scholar](https://scholar.google.com/) [Indian ocean dipole effect production] | Grey literature | - | | 2024-01-29 | 31 | - | - |
| [Google Scholar](https://scholar.google.com/) [el nino effect food] | Grey literature | - | | 2024-02-01 | 140 | - | - |
| [Google Scholar](https://scholar.google.com/) [Indian ocean dipole effect food] | Grey literature | - | | 2024-02-02 | 100 | - | - |
| [Google Scholar](https://scholar.google.com/) ["el nino" effect health] | Grey literature | - | | 2024-01-30 | 210 | - | - |
| [Google Scholar](https://scholar.google.com/) [Indian ocean dipole effect health] | Grey literature | - | | 2024-02-01 | 180 | - | - |
| [Google Scholar](https://scholar.google.com/) [el nino effect social] | Grey literature | - | | 2024-02-01 | 60 | - | - |
| [Google Scholar](https://scholar.google.com/) [Indian ocean dipole effect social] | Grey literature | - | | 2024-02-01 | 30 | - | - |
| [Google Scholar](https://scholar.google.com/) [el nino conflict] | Grey literature | - | | 2024-02-10 | 30 | - | - |
| [Google Scholar](https://scholar.google.com/) [Indian ocean dipole conflict] | Grey literature | - | | 2024-02-10 | 30 | - | - |
| [Google Scholar](https://scholar.google.com/) [el nino migration] | Grey literature | - | | 2024-02-10 | 30 | - | - |
| [Google Scholar](https://scholar.google.com/) [Indian Ocean Dipole migration] | Grey literature | - | | 2024-02-10 | 30 | - | - |
| Prevention Web | Grey literature | - | | 2024-02-02 | 200 | - | - |
| ReliefWeb | Grey literature | - | | 2024-02-01 | 240 | - | - |
| **Total for grey literature above** | | | | | **2760** | **-** | **-** |
|  |  | |  |  |  |  |  |

**Appendix Table 2. Scopus search strategy**

| # | Search string | Results | Notes |
| --- | --- | --- | --- |
| 1 | TITLE-ABS-KEY ( ( "El Niño*" OR "El Niño*" OR "El Ni˜no*" OR "Oceanic Niño Index" OR "Oceanic Nino Index" OR "southern oscillation index" OR "La Niña*" OR "La Nina*" OR ( ( "sea surface temperature" OR "SST*" ) W/3 ( "anomal*" ) ) OR "SOI index" OR "IOD" OR "IOD?" OR "+IOD" OR "IOD+" OR "pIOD" OR "pIOD?" OR "ENSO" OR "ENSO?" OR "MENSOI" OR ( "Indian Ocean" W/5 "Dipole" ) OR ( ( "nino*" OR "Niño*" ) W/3 ( "pacific" OR "3.4" OR "modoki" OR "canonical" OR "conventional" OR "cold tongue" OR "warm pool" OR "dateline" ) ) OR ( ( "dipole mode index" OR "DMI" OR "walker cell" OR "walker circulation" OR "sea surface temperature gradient" ) AND ( "indian ocean" ) ) OR "Delayed Oscillator" OR "Recharge Oscillator" OR "Western Pacific Oscillator" OR "Advective-Reflective Oscillator" OR "Unified Oscillator" ) ) | 73,968 | Concept 1: El Niño and positive Indian Ocean Dipole |
| 2 | ( TITLE-ABS-KEY ( ( "bangladesh" OR "bhutan" OR "borneo" OR "burma" OR "cambodia" OR "kampuchea" OR "khmer republic" OR "cook islands" OR "fiji" OR "guam" OR "india" OR "indonesia" OR "kiribati" OR "laos" OR "marshal island*" OR "malaysia" OR "malay* federation" OR "maldives" OR "micronesia" OR "myanmar" OR "nauru" OR "nepal" OR "Pleasant Island" OR "northern mariana islands" OR "new guinea" OR "Oceania" OR "palau" OR "philippines" OR "philipines" OR "phillipines" OR "phillippines" OR "pilipinas" OR "pacific islands" OR "polynesia" OR "samoa" OR "samoan islands" OR "navigator island" OR "navigator islands" OR "melanesia" OR "solomon island*" OR "norfolk island*" OR "santa cruz islands" OR "sri lanka" OR "ceylon" OR "tahiti" OR "thailand" OR "siam" OR "timor" OR "tonga" OR "tuvalu" OR "ellice islands" OR "vanuatu" OR "vietnam" OR "viet nam" OR "west indies" OR "bangladeshi*" OR "bangalees" OR "bajan*" OR "bhutanese" OR "bornean*" OR "burmese" OR "cambodian*" OR "cook islander*" OR "fijian*" OR "guamanian*" OR "indonesian*" OR "kirabatian*" OR "lao" OR "laotian*" OR "malaysian*" OR "maldivian*" OR "marshallese" OR "melanesian" OR "micronesian*" OR "myanma" OR "nepali*" OR "nepalese" OR "mariana islander*" OR "mariana*" OR "chamorros" OR "nauruan*" OR "norfolk islander*" OR "oceanian" OR "palauan*" OR "papua new guinean*" OR "philippine*" OR "philipine*" OR "phillipine*" OR "phillippine*" OR "filipino*" OR "filipina*" OR "pacific islander*" OR "polynesian*" OR "samoan*" OR "solomon islander*" OR "sri lankan*" OR "ceylonese" OR "tahitian*" OR "thai" OR "timorese*" OR "tongan*" OR "tuvaluan*" OR "vanuatuan*" OR "vietnamese" ) ) OR TITLE-ABS-KEY ( ( "north* pacific ocean*" OR "tropical pacific ocean*" OR "equatorial pacific ocean*" OR "mekong delta*" OR "ganges delta*" OR "ayeyarwady delta*" OR "bay of bengal" OR "arabian sea" OR "andaman sea" OR "south* pacific ocean*" OR "north indian ocean" OR "southwestern indian ocean" OR "south china sea*" OR "indian subcontinent*" OR ( "countries" AND ( "the world" OR "worldwide" OR "global*" ) ) OR ( "southeastern" W/1 "asia" ) OR ( "south eastern" W/1 "asia" ) OR "southeast asia" OR "south east asia" OR "south asia" OR ( ( "indian ocean" OR "pacific ocean" OR "tropical pacific" OR "equatorial pacific" OR "north* pacific" OR "south* pacific" OR "indochina" OR "indochinese" OR "indo pacific" OR "indo-pacific" OR "indopacific*" ) W/3 ( "adjacent" OR "border*" OR "country" OR "countries" OR "region" OR "regions" OR "island" OR "islands" OR "nation" OR "nations" OR "economies" ) ) )  ) ) | 1,931,461 | Concept 2 – Countries/ regions |
| 3 | TITLE-ABS-KEY ( ( ( "match*" W/2 ( "propensity" OR "coarsened" OR "covariate" OR "co-variate" OR "neighbor" OR "neighbour" ) ) OR "propensity score" OR "difference* in difference*" OR "difference-in-difference*" OR "differences-in-difference*" OR "double difference*" OR "quasi-experiment*" OR "quasi experiment*" OR ( "estimat*" AND "evaluat*" ) OR "instrumental variable*" OR ( "IV" W/2 ( "estimation" OR "approach*" ) ) OR ( "Heckman" W/3 ( "model*" OR "approach*" ) ) OR ( ( "two-stage" OR "two stage" ) W/3 ( "control*" OR "function*" OR "least squares" ) ) OR "regression discontinuity" OR "time series" OR "counterfactual" OR "segment* regression" OR "coefficient of variation" OR ( "non" W/2 "participant*" ) OR ( ( "control" OR "comparison" ) W/2 ( "group*" OR "condition*" OR "area*" OR "village*" OR "household*" OR "intervention" ) ) OR ( "panel*" W/2 ( "data" OR "household*" OR "model*" ) ) OR ( ( "exploit*" OR "tak* advantage" ) W/3 ( "variation*" OR "variety" OR "exogen*" OR "heterogen*" ) ) OR ( "econometric" W/2 ( "model*" OR "adjust*" ) ) OR ( "select*" W/2 ( "bias*" OR "self" ) ) OR ( "experiment*" W/2 ( "design" OR "study" OR "research" OR "evaluation" OR "evidence" OR "vary" OR "varies" OR "variation" ) ) OR ( ( "random" OR "randomised" OR "randomized" OR "randomly" ) W/2 ( "trial" OR "assign*" OR "treatment" OR "control*" OR "allocat*" OR "experiment*" OR "vary" OR "varies" OR "variation" OR "choose" OR "chose*" OR model* ) ) OR ( ( "impact*" OR "effect*" ) W/5 ( "evaluat*" OR "assess" OR "assessing" OR "assessment*" OR "analyze*" OR "analyse*" OR "analyzing" OR "analysing" OR "analysis" OR "analyses" OR "analytical" OR "estimate*" OR "estimating" OR "estimation*" OR "examin*" OR "quantif*" OR "investigat*" OR "cause" OR "causes" OR "causal" OR "causation" OR "causatively" OR "association" OR "associations" OR "associate*" OR "hypothesi*" OR "produce*" OR "production*" OR "food" OR "crop" OR "crops" OR "disease" OR "infection*" OR "health" OR "economic" OR "price" OR "prices" OR "markets" OR "socioeconomic" OR "migration" ) ) OR ( ( "association *" ) W/5 ( "evaluat*" OR "assess" OR "assessing" OR "assessment" OR "analyze*" OR "analyse*" OR "analyzing" OR "analysing" OR "analysis" OR "analyses" OR "analytical" OR "estimate*" OR "estimating" OR "estimation*" OR "examin*" OR "quantif*" OR "investigat*" OR "cause" OR "causes" OR "causal" OR "causation" OR "causatively" OR "hypothesi*" ) ) OR "spatial correlation" OR "spatial temporal" OR "inciden* rate*" OR "inciden* ratio*" OR "rate ratio*" OR ( ( "quant*" OR "effect*" OR "pattern*" ) W/5 ( "association" OR "associated" ) ) OR ( ( "composite" OR "component*" OR "spatial*" OR "variabilit*" OR "function" OR "wavelet" OR "correlation*" OR "statistical*" OR "economi*" OR "macroeconomi*" OR "empirical*" ) W/5 ( "analys*" OR "analyz*" ) ) ) ) | 13,034,080 | Concept 3 – Study designs/ Analysis |
| 4 | ( TITLE-ABS-KEY ( ( ( "match*" W/2 ( "propensity" OR "coarsened" OR "covariate" OR "co-variate" OR "neighbor" OR "neighbour" ) ) OR "propensity score" OR "difference* in difference*" OR "difference-in-difference*" OR "differences-in-difference*" OR "double difference*" OR "quasi-experiment*" OR "quasi experiment*" OR ( "estimat*" AND "evaluat*" ) OR "instrumental variable*" OR ( "IV" W/2 ( "estimation" OR "approach*" ) ) OR ( "Heckman" W/3 ( "model*" OR "approach*" ) ) OR ( ( "two-stage" OR "two stage" ) W/3 ( "control*" OR "function*" OR "least squares" ) ) OR "regression discontinuity" OR "time series" OR "counterfactual" OR "segment* regression" OR "coefficient of variation" OR ( "non" W/2 "participant*" ) OR ( ( "control" OR "comparison" ) W/2 ( "group*" OR "condition*" OR "area*" OR "village*" OR "household*" OR "intervention" ) ) OR ( "panel*" W/2 ( "data" OR "household*" OR "model*" ) ) OR ( ( "exploit*" OR "tak* advantage" ) W/3 ( "variation*" OR "variety" OR "exogen*" OR "heterogen*" ) ) OR ( "econometric" W/2 ( "model*" OR "adjust*" ) ) OR ( "select*" W/2 ( "bias*" OR "self" ) ) OR ( "experiment*" W/2 ( "design" OR "study" OR "research" OR "evaluation" OR "evidence" OR "vary" OR "varies" OR "variation" ) ) OR ( ( "random" OR "randomised" OR "randomized" OR "randomly" ) W/2 ( "trial" OR "assign*" OR "treatment" OR "control*" OR "allocat*" OR "experiment*" OR "vary" OR "varies" OR "variation" OR "choose" OR "chose*" OR model* ) ) OR ( ( "impact*" OR "effect*" ) W/5 ( "evaluat*" OR "assess" OR "assessing" OR "assessment*" OR "analyze*" OR "analyse*" OR "analyzing" OR "analysing" OR "analysis" OR "analyses" OR "analytical" OR "estimate*" OR "estimating" OR "estimation*" OR "examin*" OR "quantif*" OR "investigat*" OR "cause" OR "causes" OR "causal" OR "causation" OR "causatively" OR "association" OR "associations" OR "associate*" OR "hypothesi*" OR "produce*" OR "production*" OR "food" OR "crop" OR "crops" OR "disease" OR "infection*" OR "health" OR "economic" OR "price" OR "prices" OR "markets" OR "socioeconomic" OR "migration" ) ) OR ( ( "association *" ) W/5 ( "evaluat*" OR "assess" OR "assessing" OR "assessment" OR "analyze*" OR "analyse*" OR "analyzing" OR "analysing" OR "analysis" OR "analyses" OR "analytical" OR "estimate*" OR "estimating" OR "estimation*" OR "examin*" OR "quantif*" OR "investigat*" OR "cause" OR "causes" OR "causal" OR "causation" OR "causatively" OR "hypothesi*" ) ) OR "spatial correlation" OR "spatial temporal" OR "inciden* rate*" OR "inciden* ratio*" OR "rate ratio*" OR ( ( "quant*" OR "effect*" OR "pattern*" ) W/5 ( "association" OR "associated" ) ) OR ( ( "composite" OR "component*" OR "spatial*" OR "variabilit*" OR "function" OR "wavelet" OR "correlation*" OR "statistical*" OR "economi*" OR "macroeconomi*" OR "empirical*" ) W/5 ( "analys*" OR "analyz*" ) ) ) ) ) AND ( ( TITLE-ABS-KEY ( ( "bangladesh" OR "bhutan" OR "borneo" OR "burma" OR "cambodia" OR "kampuchea" OR "khmer republic" OR "cook islands" OR "fiji" OR "guam" OR "india" OR "indonesia" OR "kiribati" OR "laos" OR "marshal island*" OR "malaysia" OR "malay* federation" OR "maldives" OR "micronesia" OR "myanmar" OR "nauru" OR "nepal" OR "Pleasant Island" OR "northern mariana islands" OR "new guinea" OR "Oceania" OR "palau" OR "philippines" OR "philipines" OR "phillipines" OR "phillippines" OR "pilipinas" OR "pacific islands" OR "polynesia" OR "samoa" OR "samoan islands" OR "navigator island" OR "navigator islands" OR "melanesia" OR "solomon island*" OR "norfolk island*" OR "santa cruz islands" OR "sri lanka" OR "ceylon" OR "tahiti" OR "thailand" OR "siam" OR "timor" OR "tonga" OR "tuvalu" OR "ellice islands" OR "vanuatu" OR "vietnam" OR "viet nam" OR "west indies" OR "bangladeshi*" OR "bangalees" OR "bajan*" OR "bhutanese" OR "bornean*" OR "burmese" OR "cambodian*" OR "cook islander*" OR "fijian*" OR "guamanian*" OR "indonesian*" OR "kirabatian*" OR "lao" OR "laotian*" OR "malaysian*" OR "maldivian*" OR "marshallese" OR "melanesian" OR "micronesian*" OR "myanma" OR "nepali*" OR "nepalese" OR "mariana islander*" OR "mariana*" OR "chamorros" OR "nauruan*" OR "norfolk islander*" OR "oceanian" OR "palauan*" OR "papua new guinean*" OR "philippine*" OR "philipine*" OR "phillipine*" OR "phillippine*" OR "filipino*" OR "filipina*" OR "pacific islander*" OR "polynesian*" OR "samoan*" OR "solomon islander*" OR "sri lankan*" OR "ceylonese" OR "tahitian*" OR "thai" OR "timorese*" OR "tongan*" OR "tuvaluan*" OR "vanuatuan*" OR "vietnamese" ) ) OR TITLE-ABS-KEY ( ( "north* pacific ocean*" OR "tropical pacific ocean*" OR "equatorial pacific ocean*" OR "mekong delta*" OR "ganges delta*" OR "ayeyarwady delta*" OR "bay of bengal" OR "arabian sea" OR "andaman sea" OR "south* pacific ocean*" OR "north indian ocean" OR "southwestern indian ocean" OR "south china sea*" OR "indian subcontinent*" OR ( "countries" AND ( "the world" OR "worldwide" OR "global*" ) ) OR ( "southeastern" W/1 "asia" ) OR ( "south eastern" W/1 "asia" ) OR "southeast asia" OR "south east asia" OR "south asia" OR ( ( "indian ocean" OR "pacific ocean" OR "tropical pacific" OR "equatorial pacific" OR "north* pacific" OR "south* pacific" OR "indochina" OR "indochinese" OR "indo pacific" OR "indo-pacific" OR "indopacific*" ) W/3 ( "adjacent" OR "border*" OR "country" OR "countries" OR "region" OR "regions" OR "island" OR "islands" OR "nation" OR "nations" OR "economies" ) ) ) ) ) ) AND ( TITLE-ABS-KEY ( ( "El Niño*" OR "El Niño*" OR "El Ni˜no*" OR "Oceanic Niño Index" OR "Oceanic Nino Index" OR "southern oscillation index" OR "La Niña*" OR "La Nina*" OR ( ( "sea surface temperature" OR "SST*" ) W/3 ( "anomal*" ) ) OR "SOI index" OR "IOD" OR "IOD?" OR "+IOD" OR "IOD+" OR "pIOD" OR "pIOD?" OR "ENSO" OR "ENSO?" OR "MENSOI" OR ( "Indian Ocean" W/5 "Dipole" ) OR ( ( "nino*" OR "Niño*" ) W/3 ( "pacific" OR "3.4" OR "modoki" OR "canonical" OR "conventional" OR "cold tongue" OR "warm pool" OR "dateline" ) ) OR ( ( "dipole mode index" OR "DMI" OR "walker cell" OR "walker circulation" OR "sea surface temperature gradient" ) AND ( "indian ocean" ) ) OR "Delayed Oscillator" OR "Recharge Oscillator" OR "Western Pacific Oscillator" OR "Advective-Reflective Oscillator" OR "Unified Oscillator" ) ) ) | 3,456 | All concepts combined using AND |
|  | AND PUBYEAR > 1989 AND PUBYEAR < 2025 | 3.407 | Publication date limit |

**Appendix Table 3. Web of Science Core Collection search strategy. (Indexes included: A&HCI, ESCI (2015 – present), CPCI-SSH, CPCI-S, SCI-EXPANDED, SSCI)**

| # | Search Query | Results | Notes |
| --- | --- | --- | --- |
| 1 | TS=("El Niño*" or "El Niño*" or "El Ni˜no*" or "Oceanic Niño Index" or "Oceanic Nino Index" or "southern oscillation index" OR (("sea surface temperature" or "SST*") NEAR/3 ("anomal*" )) OR "SOI index" or "IOD$" or "+IOD" or "IOD+" or "pIOD$" or "ENSO$" or "SSTA$" or "MENSOI" OR ("Indian Ocean" NEAR/5 "Dipole") OR (("nino*" or "Niño*") NEAR/3 ("pacific" or "3.4" or "modoki" or "canonical" or "conventional" or "cold tongue" or "warm pool" or "dateline")) OR ( ("dipole mode index" or "DMI" or "walker cell" or "walker circulation" or "sea surface temperature gradient*") AND ("indian ocean") ) OR "Delayed Oscillator" or "Recharge Oscillator" or "Western Pacific Oscillator" or "Advective-Reflective Oscillator" or "Unified Oscillator" OR "La Niña*" or "La Nina*" ) | 59,499 | Concept 1 – El Niño and positive Indian Ocean Dipole |
| 2 | TS=("bangladesh" or "bhutan" or "borneo" or "burma" or "cambodia" or "kampuchea" or "khmer republic" or "cook islands" or "fiji" or "guam" or "india" or "indonesia" or "kiribati" or "laos" or "marshal island$" or "malaysia" or "malay$ federation" or "maldives" or "micronesia" or "myanmar" or "nauru" or "nepal" or "Pleasant Island" or "northern mariana islands" or "new guinea" or "Oceania" or "palau" or "philippines" or "philipines" or "phillipines" or "phillippines" or "pilipinas" or "pacific islands" or "polynesia" or "samoa" or "samoan islands" or "navigator island" or "navigator islands" or "melanesia" or "solomon island$" or "norfolk island$" or "santa cruz islands" or "sri lanka" or "ceylon" or "tahiti" or "thailand" or "siam" or "timor" or "tonga" or "tuvalu" or "ellice islands" or "vanuatu" or "vietnam" or "viet nam" or "west indies" or "bangladeshi$" or "bangalees" or "bajan$" or "bhutanese" or "bornean$" or "burmese" or "cambodian$" or "cook islander$" or "fijian$" or "guamanian$" or "indonesian$" or "kirabatian$" or "lao" or "laotian$" or "malaysian$" or "maldivian$" or "marshallese" or "melanesian" or "micronesian$" or "myanma" or "nepali$" or "nepalese" or "mariana islander$" or "mariana$" or "chamorros" or "nauruan$" or "norfolk islander$" or "oceanian" or "palauan$" or "papua new guinean$" or "philippine$" or "philipine$" or "phillipine$" or "phillippine$" or "filipino$" or "filipina$" or "pacific islander$" or "polynesian$" or "samoan$" or "solomon islander$" or "sri lankan$" or "ceylonese" or "tahitian$" or "thai" or "timorese$" or "tongan$" or "tuvaluan$" or "vanuatuan$" OR "vietnamese") OR CU=("bangladesh" or "bhutan" or "borneo" or "burma" or "cambodia" or "kampuchea" or "khmer republic" or "cook islands" or "fiji" or "guam" or "india" or "indonesia" or "kiribati" or "laos" or "marshal island$" or "malaysia" or "malay$ federation" or "maldives" or "micronesia" or "myanmar" or "nauru" or "nepal" or "Pleasant Island" or "northern mariana islands" or "new guinea" or "Oceania" or "palau" or "philippines" or "philipines" or "phillipines" or "phillippines" or "pilipinas" or "pacific islands" or "polynesia" or "samoa" or "samoan islands" or "navigator island" or "navigator islands" or "melanesia" or "solomon island$" or "norfolk island$" or "santa cruz islands" or "sri lanka" or "ceylon" or "tahiti" or "thailand" or "siam" or "timor" or "tonga" or "tuvalu" or "ellice islands" or "vanuatu" or "vietnam" or "viet nam" or "west indies" or "bangladeshi$" or "bangalees" or "bajan$" or "bhutanese" or "bornean$" or "burmese" or "cambodian$" or "cook islander$" or "fijian$" or "guamanian$" or "indonesian$" or "kirabatian$" or "lao" or "laotian$" or "malaysian$" or "maldivian$" or "marshallese" or "melanesian" or "micronesian$" or "myanma" or "nepali$" or "nepalese" or "mariana islander$" or "mariana$" or "chamorros" or "nauruan$" or "norfolk islander$" or "oceanian" or "palauan$" or "papua new guinean$" or "philippine$" or "philipine$" or "phillipine$" or "phillippine$" or "filipino$" or "filipina$" or "pacific islander$" or "polynesian$" or "samoan$" or "solomon islander$" or "sri lankan$" or "ceylonese" or "tahitian$" or "thai" or "timorese$" or "tongan$" or "tuvaluan$" or "vanuatuan$" or "vietnamese") | 3,946,249 | Concept 2 – Countries/ regions |
| 3 | TS=("north* pacific ocean*" or "tropical pacific ocean*" or "equatorial pacific ocean*" or "south* pacific ocean*" or "mekong delta$" or "ganges delta$" or "ayeyarwady delta$" or "bay of bengal" or "arabian sea" or "andaman sea" or "north indian ocean" or "southwestern indian ocean" or "south china sea$" or "indian subcontinent*" or ( "countries" AND ( "the world" or "worldwide" or "global*" ) ) or ("southeastern" NEAR/1 "asia") or ("south eastern" NEAR/1 "asia") or "southeast asia" or "south east asia" or "south asia") OR TI=("global") OR TS=(("indian ocean" or "pacific ocean" or "indo pacific" or "indo-pacific" or "indopacific*" or "north pacific" or "tropical pacific" or "equatorial pacific" or "south pacific" or "indochina" or "indochinese" ) NEAR/3 ("adjacent" or "border*" or "country" or "countries" or "region" or "regions" or "island" or "islands" or "nation" or "nations" or "economies")) | 657,946 |  |
| 4 | #2 OR #3 | 4,486,371 |  |
| 5 | TS=( ("match*" NEAR/2 ("propensity" or "coarsened" or "covariate" or "co-variate" or "neighbo$r")) or "propensity score" or "difference* in difference*" or "difference-in-difference*" or "differences-in-difference*" or "double difference*" or "quasi-experiment*" or "quasi experiment*" or ("estimat*" and "evaluat*") or "instrumental variable*" or ("IV" NEAR/2 ("estimation" or "approach*")) or ("Heckman" NEAR/3 ("model*" or "approach*")) or (("two-stage" or "two stage") NEAR/3 ("control*" or "function*" or "least squares")) or "regression discontinuity" or "time series" or "counterfactual" or "segment* regression" or "coefficient of variation" or ("non" NEAR/2 "participant*") or (("control" or "comparison") NEAR/2 ("group*" or "condition*" or "area*" or "village*" or "household*" or "intervention")) or ("panel*" NEAR/2 ("data" or "household*" or "model*")) or (("exploit*" or "tak* advantage") NEAR/3 ("variation*" or "variety" or "exogen*" or "heterogen*")) or ("econometric" NEAR/2 ("model*" or "adjust*")) or ("select*" NEAR/2 ("bias*" or "self")) OR ("experiment*" NEAR/2 ("design" or "study" or "research" or "evaluation" or "evidence" or "vary" or "varies" or "variation")) or (("random" or "randomi$ed" or "randomly") NEAR/2 ("trial" or "assign*" or "treatment" or "control*" or "allocat*" or "experiment*" or "vary" or "varies" or "variation" or "choose" or "chose*" or model*)) or (("impact$" or "effect*") NEAR/5 ("evaluat*" or "assess" or "assessing" or "assessment*" or "analyze*" or "analyse*" or "analyzing" or "analysing" or "analysis" or "analyses" or "analytical" or "estimate*" or "estimating" or "estimation*" or "examin*" or "quantif*" or "investigat*" or "cause" or "causes" or "causal" or "causation" or "causatively" or "association$" or "associate*" or "hypothesi*" or "produce*" or "production*" or "food" or "crop" or "crops" or "disease" or "infection*" or "health" or "economic" or "price" or "prices" or "markets" or "socioeconomic" or "migration"**))** or (("association") NEAR/5 ("evaluat*" or "assess" or "assessing" or "assessment" or "analyze*" or "analyse*" or "analyzing" or "analysing" or "analysis" or "analyses" or "analytical" or "estimate*" or "estimating" or "estimation*" or "examin*" or "quantif*" or "investigat*" or "cause" or "causes" or "causal" or "causation" or "causatively" or "hypothesi*")) or "spatial correlation" or "spatial temporal" or "inciden* rate$" or "inciden* ratio$" or "rate ratio$" or (("quant*" OR "effect$" OR "pattern$") NEAR/5 ("association" OR "associated")) OR (("composite" or "component*" or "spatial*" or "variabilit*" or "function" or "correlation*" or "wavelet" or "statistical*" or "economi*" or "macroeconomi*" or "empirical*") NEAR/5 ("analys*" or "analyz*")) ) | 8,702,453 | Concept 3 – Study designs/ Analysis |
| 6 | #1 AND #4 AND #5 | 4,263 | All concepts |
| 7 | #5 AND #4 AND #1 and 2024 or 2023 or 2022 or 2021 or 2020 or 2019 or 2018 or 2017 or 2016 or 2015 or 2014 or 2013 or 2012 or 2011 or 2010 or 2009 or 2008 or 2007 or 2006 or 2005 or 2004 or 2003 or 2002 or 2001 or 2000 or 1999 or 1998 or 1997 or 1996 or 1995 or 1994 or 1993 or 1992 or 1991 or 1990 (Publication Years) and Geosciences Multidisciplinary or Environmental Sciences or Oceanography or Water Resources or Multidisciplinary Sciences or Remote Sensing or Geography Physical or Marine Freshwater Biology or Ecology or Imaging Science Photographic Technology or Environmental Studies or Engineering Electrical Electronic or Public Environmental Occupational Health or Biodiversity Conservation or Forestry or Fisheries or Limnology or Engineering Environmental or Engineering Ocean or Infectious Diseases or Agronomy or Green Sustainable Science Technology or Agriculture Multidisciplinary or Tropical Medicine or Computer Science Information Systems or Optics or Telecommunications or Geography or Parasitology or Astronomy Astrophysics or Engineering Marine or Mathematics Interdisciplinary Applications or Physics Multidisciplinary or Economics or Physics Applied or Plant Sciences or Chemistry Multidisciplinary or Chemistry Physical or Statistics Probability or Toxicology or Biology or Computer Science Interdisciplinary Applications or Engineering Multidisciplinary or Geology or Computer Science Artificial Intelligence or Materials Science Multidisciplinary or Mathematics Applied or Computer Science Theory Methods or Evolutionary Biology or Physics Fluids Plasmas or Dentistry Oral Surgery Medicine or Engineering Aerospace or Engineering Geological or Food Science Technology or Medicine General Internal or Microbiology or Biochemistry Molecular Biology or Crystallography or Energy Fuels or International Relations or Physics Mathematical or Biophysics or Chemistry Inorganic Nuclear or Computer Science Hardware Architecture or Computer Science Software Engineering or Agricultural Economics Policy or Automation Control Systems or Chemistry Analytical or Genetics Heredity or Instruments Instrumentation or Physics Atomic Molecular Chemical or Physiology or Zoology or Agriculture Dairy Animal Science or Anthropology or Archaeology or Chemistry Medicinal or Development Studies or Education Scientific Disciplines or Entomology or Health Care Sciences Services or Management or Nanoscience Nanotechnology or Nuclear Science Technology or Operations Research Management Science or Social Sciences Interdisciplinary or Soil Science or Spectroscopy or Agricultural Engineering or Biochemical Research Methods or Biotechnology Applied Microbiology or Endocrinology Metabolism or Engineering Biomedical or Engineering Mechanical or Hospitality Leisure Sport Tourism or Mathematical Computational Biology or Mechanics or Ornithology or Pharmacology Pharmacy or Transportation Science Technology or Veterinary Sciences or Acoustics or Anesthesiology or Area Studies or Business Finance or Chemistry Applied or Computer Science Cybernetics or Construction Building Technology or Engineering Industrial or Engineering Manufacturing or Gastroenterology Hepatology or Geriatrics Gerontology or History or History Philosophy Of Science or Horticulture or Immunology or Integrative Complementary Medicine or Materials Science Biomaterials or Neurosciences or Oncology or Physics Condensed Matter or Physics Nuclear or Political Science or Psychiatry or Public Administration or Robotics or Sociology or Sport Sciences or Thermodynamics or Transportation or Virology (Web o  Science Categories) | 2,567 | Date limit and Web of Science Categories limits due to feasibility. 1990 – 2024 + Web of Science categories (include all except - Geochemistry Geophysics, Meteorology and atmospheric sciences, civil engineering, paleontology) |

**Appendix Table 4. Academic Search Complete (EBSCOhost) Search strategy**

| # | Query | Results | Notes |
| --- | --- | --- | --- |
| S1 | DE "SOUTHERN oscillation" OR DE "El Niño" | 9,242 | Concept 1 - El Niño and positive Indian Ocean Dipole |
| S2 | TI ( ("El Niño*" or "El Niño*" or "El Ni˜no*" or "Oceanic Niño Index" or "Oceanic Nino Index" or "southern oscillation index" or (("sea surface temperature" or "SST*") N3 ("anomal*")) OR "SOI index" or "IOD#" or "+IOD" or "IOD+" or "pIOD#" or "ENSO#" or "MENSOI" OR ("Indian Ocean" N5 "Dipole") OR (("nino*" or "Niño*") N3 ("pacific" or "3.4" or "modoki" or "canonical" or "conventional" or "cold tongue" or "warm pool" or "dateline")) OR ( ("dipole mode index" or "DMI" or "walker cell" or "walker circulation" or "sea surface temperature gradient") AND ("indian ocean") ) OR "Delayed Oscillator" or "Recharge Oscillator" or "Western Pacific Oscillator" or "Advective-Reflective Oscillator" or "Unified Oscillator" OR "La Niña*" or "La Nina*") ) OR AB ( ("El Niño*" or "El Niño*" or "El Ni˜no*" or "Oceanic Niño Index" or "Oceanic Nino Index" or "southern oscillation index" or (("sea surface temperature" or "SST*") N3 ("anomal*")) OR "SOI index" or "IOD#" or "+IOD" or "IOD+" or "pIOD#" or "ENSO#" or "MENSOI" OR ("Indian Ocean" N5 "Dipole") OR (("nino*" or "Niño*") N3 ("pacific" or "3.4" or "modoki" or "canonical" or "conventional" or "cold tongue" or "warm pool" or "dateline")) OR ( ("dipole mode index" or "DMI" or "walker cell" or "walker circulation" or "sea surface temperature gradient") AND ("indian ocean") ) OR "Delayed Oscillator" or "Recharge Oscillator" or "Western Pacific Oscillator" or "Advective-Reflective Oscillator" or "Unified Oscillator" OR "La Niña*" or "La Nina*") ) OR KW ( ("El Niño*" or "El Niño*" or "El Ni˜no*" or "Oceanic Niño Index" or "Oceanic Nino Index" or "southern oscillation index" or (("sea surface temperature" or "SST*") N3 ("anomal*")) OR "SOI index" or "IOD#" or "+IOD" or "IOD+" or "pIOD#" or "ENSO#" or "MENSOI" OR ("Indian Ocean" N5 "Dipole") OR (("nino*" or "Niño*") N3 ("pacific" or "3.4" or "modoki" or "canonical" or "conventional" or "cold tongue" or "warm pool" or "dateline")) OR ( ("dipole mode index" or "DMI" or "walker cell" or "walker circulation" or "sea surface temperature gradient") AND ("indian ocean") ) OR "Delayed Oscillator" or "Recharge Oscillator" or "Western Pacific Oscillator" or "Advective-Reflective Oscillator" or "Unified Oscillator" OR "La Niña*" or "La Nina*") ) OR SU ( ("El Niño*" or "El Niño*" or "El Ni˜no*" or "Oceanic Niño Index" or "Oceanic Nino Index" or "southern oscillation index" or (("sea surface temperature" or "SST*") N3 ("anomal*")) OR "SOI index" or "IOD#" or "+IOD" or "IOD+" or "pIOD#" or "ENSO#" or "MENSOI" OR ("Indian Ocean" N5 "Dipole") OR (("nino*" or "Niño*") N3 ("pacific" or "3.4" or "modoki" or "canonical" or "conventional" or "cold tongue" or "warm pool" or "dateline")) OR ( ("dipole mode index" or "DMI" or "walker cell" or "walker circulation" or "sea surface temperature gradient") AND ("indian ocean") ) OR "Delayed Oscillator" or "Recharge Oscillator" or "Western Pacific Oscillator" or "Advective-Reflective Oscillator" or "Unified Oscillator" OR "La Niña*" or "La Nina*") ) | 29,004 |  |
| S3 | S1 OR S2 | 29,148 |  |
| S4 | TI ( ( ("bangladesh" or "bhutan" or "borneo" or "burma" or "cambodia" or "kampuchea" or "khmer republic" or "cook islands" or "fiji" or "guam" or "india" or "indonesia" or "kiribati" or "laos" or "marshal island#" or "malaysia" or "malay# federation" or "maldives" or "melanesia" or "micronesia" or "myanmar" or "nauru" or "nepal" or "Pleasant Island" or "northern mariana islands" or "new guinea" or "Oceania" or "palau" or "philippines" or "philipines" or "phillipines" or "phillippines" or "pilipinas" or "pacific islands" or "polynesia" or "samoa" or "samoan islands" or "navigator island" or "navigator islands" or "solomon island#" or "norfolk island#" or "santa cruz island#" or "sri lanka" or "ceylon" or "thailand" or "siam" or "timor" or "tonga" or "tahiti" or "tuvalu" or "ellice islands" or "vanuatu" or "vietnam" or "viet nam" or "west indies" or "bangladeshi#" or "bangalees" or "bajan#" or "bhutanese" or "bornean#" or "burmese" or "cambodian#" or "cook islander#" or "fijian#" or "guamanian#" or "indonesian#" or "kirabatian#" or "lao" or "laotian#" or "malaysian#" or "maldivian#" or "marshallese" or "melanesian" or "micronesian#" or "myanma" or "nepali#" or "nepalese" or "northern mariana islander#" or "mariana#" or "chamorros" or "nauruan#" or "norfolk islander#" or "oceanian" or "palauan#" or "papua new guinean#" or "philippine#" or "philipine#" or "phillipine#" or "phillippine#" or "filipino#" or "filipina#" or "pacific islander#" or "polynesian#" or "samoan#" or "solomon islander#" or "sri lankan#" or "ceylonese" or "tahitian#" or "thai" or "timorese#" or "tongan#" or "tuvaluan#" or "vanuatuan#" or "vietnamese") ) ) OR AB ( ( ("bangladesh" or "bhutan" or "borneo" or "burma" or "cambodia" or "kampuchea" or "khmer republic" or "cook islands" or "fiji" or "guam" or "india" or "indonesia" or "kiribati" or "laos" or "marshal island#" or "malaysia" or "malay# federation" or "maldives" or "melanesia" or "micronesia" or "myanmar" or "nauru" or "nepal" or "Pleasant Island" or "northern mariana islands" or "new guinea" or "Oceania" or "palau" or "philippines" or "philipines" or "phillipines" or "phillippines" or "pilipinas" or "pacific islands" or "polynesia" or "samoa" or "samoan islands" or "navigator island" or "navigator islands" or "solomon island#" or "norfolk island#" or "santa cruz island#" or "sri lanka" or "ceylon" or "thailand" or "siam" or "timor" or "tonga" or "tahiti" or "tuvalu" or "ellice islands" or "vanuatu" or "vietnam" or "viet nam" or "west indies" or "bangladeshi#" or "bangalees" or "bajan#" or "bhutanese" or "bornean#" or "burmese" or "cambodian#" or "cook islander#" or "fijian#" or "guamanian#" or "indonesian#" or "kirabatian#" or "lao" or "laotian#" or "malaysian#" or "maldivian#" or "marshallese" or "melanesian" or "micronesian#" or "myanma" or "nepali#" or "nepalese" or "northern mariana islander#" or "mariana#" or "chamorros" or "nauruan#" or "norfolk islander#" or "oceanian" or "palauan#" or "papua new guinean#" or "philippine#" or "philipine#" or "phillipine#" or "phillippine#" or "filipino#" or "filipina#" or "pacific islander#" or "polynesian#" or "samoan#" or "solomon islander#" or "sri lankan#" or "ceylonese" or "tahitian#" or "thai" or "timorese#" or "tongan#" or "tuvaluan#" or "vanuatuan#" or "vietnamese") ) ) OR KW ( ( ("bangladesh" or "bhutan" or "borneo" or "burma" or "cambodia" or "kampuchea" or "khmer republic" or "cook islands" or "fiji" or "guam" or "india" or "indonesia" or "kiribati" or "laos" or "marshal island#" or "malaysia" or "malay# federation" or "maldives" or "melanesia" or "micronesia" or "myanmar" or "nauru" or "nepal" or "Pleasant Island" or "northern mariana islands" or "new guinea" or "Oceania" or "palau" or "philippines" or "philipines" or "phillipines" or "phillippines" or "pilipinas" or "pacific islands" or "polynesia" or "samoa" or "samoan islands" or "navigator island" or "navigator islands" or "solomon island#" or "norfolk island#" or "santa cruz island#" or "sri lanka" or "ceylon" or "thailand" or "siam" or "timor" or "tonga" or "tahiti" or "tuvalu" or "ellice islands" or "vanuatu" or "vietnam" or "viet nam" or "west indies" or "bangladeshi#" or "bangalees" or "bajan#" or "bhutanese" or "bornean#" or "burmese" or "cambodian#" or "cook islander#" or "fijian#" or "guamanian#" or "indonesian#" or "kirabatian#" or "lao" or "laotian#" or "malaysian#" or "maldivian#" or "marshallese" or "melanesian" or "micronesian#" or "myanma" or "nepali#" or "nepalese" or "northern mariana islander#" or "mariana#" or "chamorros" or "nauruan#" or "norfolk islander#" or "oceanian" or "palauan#" or "papua new guinean#" or "philippine#" or "philipine#" or "phillipine#" or "phillippine#" or "filipino#" or "filipina#" or "pacific islander#" or "polynesian#" or "samoan#" or "solomon islander#" or "sri lankan#" or "ceylonese" or "tahitian#" or "thai" or "timorese#" or "tongan#" or "tuvaluan#" or "vanuatuan#" or "vietnamese") ) ) OR GE ( ( ("bangladesh" or "bhutan" or "borneo" or "burma" or "cambodia" or "kampuchea" or "khmer republic" or "cook islands" or "fiji" or "guam" or "india" or "indonesia" or "kiribati" or "laos" or "marshal island#" or "malaysia" or "malay# federation" or "maldives" or "melanesia" or "micronesia" or "myanmar" or "nauru" or "nepal" or "Pleasant Island" or "northern mariana islands" or "new guinea" or "Oceania" or "palau" or "philippines" or "philipines" or "phillipines" or "phillippines" or "pilipinas" or "pacific islands" or "polynesia" or "samoa" or "samoan islands" or "navigator island" or "navigator islands" or "solomon island#" or "norfolk island#" or "santa cruz island#" or "sri lanka" or "ceylon" or "thailand" or "siam" or "timor" or "tonga" or "tahiti" or "tuvalu" or "ellice islands" or "vanuatu" or "vietnam" or "viet nam" or "west indies" or "bangladeshi#" or "bangalees" or "bajan#" or "bhutanese" or "bornean#" or "burmese" or "cambodian#" or "cook islander#" or "fijian#" or "guamanian#" or "indonesian#" or "kirabatian#" or "lao" or "laotian#" or "malaysian#" or "maldivian#" or "marshallese" or "melanesian" or "micronesian#" or "myanma" or "nepali#" or "nepalese" or "northern mariana islander#" or "mariana#" or "chamorros" or "nauruan#" or "norfolk islander#" or "oceanian" or "palauan#" or "papua new guinean#" or "philippine#" or "philipine#" or "phillipine#" or "phillippine#" or "filipino#" or "filipina#" or "pacific islander#" or "polynesian#" or "samoan#" or "solomon islander#" or "sri lankan#" or "ceylonese" or "tahitian#" or "thai" or "timorese#" or "tongan#" or "tuvaluan#" or "vanuatuan#" or "vietnamese") ) ) OR SU ( ( ("bangladesh" or "bhutan" or "borneo" or "burma" or "cambodia" or "kampuchea" or "khmer republic" or "cook islands" or "fiji" or "guam" or "india" or "indonesia" or "kiribati" or "laos" or "marshal island#" or "malaysia" or "malay# federation" or "maldives" or "melanesia" or "micronesia" or "myanmar" or "nauru" or "nepal" or "Pleasant Island" or "northern mariana islands" or "new guinea" or "Oceania" or "palau" or "philippines" or "philipines" or "phillipines" or "phillippines" or "pilipinas" or "pacific islands" or "polynesia" or "samoa" or "samoan islands" or "navigator island" or "navigator islands" or "solomon island#" or "norfolk island#" or "santa cruz island#" or "sri lanka" or "ceylon" or "thailand" or "siam" or "timor" or "tonga" or "tahiti" or "tuvalu" or "ellice islands" or "vanuatu" or "vietnam" or "viet nam" or "west indies" or "bangladeshi#" or "bangalees" or "bajan#" or "bhutanese" or "bornean#" or "burmese" or "cambodian#" or "cook islander#" or "fijian#" or "guamanian#" or "indonesian#" or "kirabatian#" or "lao" or "laotian#" or "malaysian#" or "maldivian#" or "marshallese" or "melanesian" or "micronesian#" or "myanma" or "nepali#" or "nepalese" or "northern mariana islander#" or "mariana#" or "chamorros" or "nauruan#" or "norfolk islander#" or "oceanian" or "palauan#" or "papua new guinean#" or "philippine#" or "philipine#" or "phillipine#" or "phillippine#" or "filipino#" or "filipina#" or "pacific islander#" or "polynesian#" or "samoan#" or "solomon islander#" or "sri lankan#" or "ceylonese" or "tahitian#" or "thai" or "timorese#" or "tongan#" or "tuvaluan#" or "vanuatuan#" or "vietnamese") ) ) | 658,898 | Concept 2 - Countries/ regions |
| S5 | TI ( ( ("north* pacific ocean*" or "tropical pacific ocean*" or "equatorial pacific ocean*" or "south* pacific ocean*" or "mekong delta#" or "ganges delta#" or "ayeyarwady delta#" or "arabian sea" or "andaman sea" or "bay of Bengal" or "north indian ocean" or "southwestern indian ocean" or "south china sea#" or "indian subcontinent*" or ("countries" AND ("the world" or "worldwide" or "global*")) or ("southeastern" N1 "asia") or ("south eastern" N1 "asia") or "southeast asia" or "south east asia" or "south asia" or (("indian ocean" or "pacific ocean" or "indo pacific" or "indo-pacific" or "indopacific*" or "north pacific" or "tropical pacific" or "equatorial pacific" or "south pacific" or "indochina" or "indochinese") N3 ("adjacent" or "border*" or "country" or "countries" or "region" or "regions" or "island" or "islands" or "nation" or "nations" or "economies")) ) ) ) OR AB ( ( ("north* pacific ocean*" or "tropical pacific ocean*" or "equatorial pacific ocean*" or "south* pacific ocean*" or "mekong delta#" or "ganges delta#" or "ayeyarwady delta#" or "arabian sea" or "andaman sea" or "bay of Bengal" or "north indian ocean" or "southwestern indian ocean" or "south china sea#" or "indian subcontinent*" or ("countries" AND ("the world" or "worldwide" or "global*")) or ("southeastern" N1 "asia") or ("south eastern" N1 "asia") or "southeast asia" or "south east asia" or "south asia" or (("indian ocean" or "pacific ocean" or "indo pacific" or "indo-pacific" or "indopacific*" or "north pacific" or "tropical pacific" or "equatorial pacific" or "south pacific" or "indochina" or "indochinese") N3 ("adjacent" or "border*" or "country" or "countries" or "region" or "regions" or "island" or "islands" or "nation" or "nations" or "economies")) ) ) ) OR KW ( ( ("north* pacific ocean*" or "tropical pacific ocean*" or "equatorial pacific ocean*" or "south* pacific ocean*" or "mekong delta#" or "ganges delta#" or "ayeyarwady delta#" or "arabian sea" or "andaman sea" or "bay of Bengal" or "north indian ocean" or "southwestern indian ocean" or "south china sea#" or "indian subcontinent*" or ("countries" AND ("the world" or "worldwide" or "global*")) or ("southeastern" N1 "asia") or ("south eastern" N1 "asia") or "southeast asia" or "south east asia" or "south asia" or (("indian ocean" or "pacific ocean" or "indo pacific" or "indo-pacific" or "indopacific*" or "north pacific" or "tropical pacific" or "equatorial pacific" or "south pacific" or "indochina" or "indochinese") N3 ("adjacent" or "border*" or "country" or "countries" or "region" or "regions" or "island" or "islands" or "nation" or "nations" or "economies")) ) ) ) OR GE ( ( ("north* pacific ocean*" or "tropical pacific ocean*" or "equatorial pacific ocean*" or "south* pacific ocean*" or "mekong delta#" or "ganges delta#" or "ayeyarwady delta#" or "arabian sea" or "andaman sea" or "bay of Bengal" or "north indian ocean" or "southwestern indian ocean" or "south china sea#" or "indian subcontinent*" or ("countries" AND ("the world" or "worldwide" or "global*")) or ("southeastern" N1 "asia") or ("south eastern" N1 "asia") or "southeast asia" or "south east asia" or "south asia" or (("indian ocean" or "pacific ocean" or "indo pacific" or "indo-pacific" or "indopacific*" or "north pacific" or "tropical pacific" or "equatorial pacific" or "south pacific" or "indochina" or "indochinese") N3 ("adjacent" or "border*" or "country" or "countries" or "region" or "regions" or "island" or "islands" or "nation" or "nations" or "economies")) ) ) ) OR SU ( ( ("north* pacific ocean*" or "tropical pacific ocean*" or "equatorial pacific ocean*" or "south* pacific ocean*" or "mekong delta#" or "ganges delta#" or "ayeyarwady delta#" or "arabian sea" or "andaman sea" or "bay of Bengal" or "north indian ocean" or "southwestern indian ocean" or "south china sea#" or "indian subcontinent*" or ("countries" AND ("the world" or "worldwide" or "global*")) or ("southeastern" N1 "asia") or ("south eastern" N1 "asia") or "southeast asia" or "south east asia" or "south asia" or (("indian ocean" or "pacific ocean" or "indo pacific" or "indo-pacific" or "indopacific*" or "north pacific" or "tropical pacific" or "equatorial pacific" or "south pacific" or "indochina" or "indochinese") N3 ("adjacent" or "border*" or "country" or "countries" or "region" or "regions" or "island" or "islands" or "nation" or "nations" or "economies")) ) ) ) | 245,506 |  |
| S6 | S4 OR S5 | 856,739 |  |
| S7 | TI ( ( ("match*" N2 ("propensity" or "coarsened" or "covariate" or "co-variate" or "neighbo#r")) or "propensity score" or "difference* in difference*" or "difference-in-difference*" or "differences-in-difference*" or "double difference*" or "quasi-experiment*" or "quasi experiment*" or ("estimat*" and "evaluat*") or "instrumental variable*" or ("IV" N2 ("estimation" or "approach*")) or ("Heckman" N3 ("model*" or "approach*")) or (("two-stage" or "two stage") N3 ("control*" or "function*" or "least squares")) or "regression discontinuity" or "time series" or "counterfactual" or "segment* regression" or "coefficient of variation" or ("non" N2 "participant*") or (("control" or "comparison") N2 ("group*" or "condition*" or "area*" or "village*" or "household*" or "intervention")) or ("panel*" N2 ("data" or "household*" or "model*")) or (("exploit*" or "tak* advantage") N3 ("variation*" or "variety" or "exogen*" or "heterogen*")) or ("econometric" N2 ("model*" or "adjust*")) or ("select*" N2 ("bias*" or "self")) or ("experiment*" N2 ("design" or "study" or "research" or "evaluation" or "evidence" or "vary" or "varies" or "variation")) or (("random" or "randomi#ed" or "randomly") N2 ("trial" or "assign*" or "treatment" or "control*" or "allocat*" or "experiment*" or "vary" or "varies" or "variation" or "choose" or "chose*" or model*)) or (("impact#" or "effect*") N5 ("evaluat*" or "assess" or "assessing" or "assessment*" or "analyze*" or "analyse*" or "analyzing" or "analysing" or "analysis" or "analyses" or "analytical" or "estimate*" or "estimating" or "estimation*" or "examin*" or "quantif*" or "investigat*" or "cause" or "causes" or "causal" or "causation" or "causatively" or "association#" or "associate*" or "hypothesi*" or "produce*" or "production*" or "food" or "crop" or "crops" or "disease" or "infection*" or "health" or "economic" or "price" or "prices" or "markets" or "socioeconomic" or "migration")) OR (("association*") N5 ("evaluat*" or "assess" or "assessing" or "assessment*" or "analyze*" or "analyse*" or "analyzing" or "analysing" or "analysis" or "analyses" or "analytical" or "estimate*" or "estimating" or "estimation*" or "examin*" or "quantif*" or "investigat*" or "cause" or "causal" or "causation" or "causatively" or "hypothesi*")) or "spatial correlation" or "spatial temporal" or "inciden* rate#" or "inciden* ratio#" or "rate ratio#" or (("quant*" OR "effect#" OR "pattern#") N5 ("association" OR "associated")) OR (("composite" or "component*" or "spatial*" or "variabilit*" or "function" or "wavelet" or "correlation*" or "statistical*" or "economi*" or "macroeconomi*" or "empirical*") N5 ("analys*" or "analyz*")) ) ) OR AB ( ( ("match*" N2 ("propensity" or "coarsened" or "covariate" or "co-variate" or "neighbo#r")) or "propensity score" or "difference* in difference*" or "difference-in-difference*" or "differences-in-difference*" or "double difference*" or "quasi-experiment*" or "quasi experiment*" or ("estimat*" and "evaluat*") or "instrumental variable*" or ("IV" N2 ("estimation" or "approach*")) or ("Heckman" N3 ("model*" or "approach*")) or (("two-stage" or "two stage") N3 ("control*" or "function*" or "least squares")) or "regression discontinuity" or "time series" or "counterfactual" or "segment* regression" or "coefficient of variation" or ("non" N2 "participant*") or (("control" or "comparison") N2 ("group*" or "condition*" or "area*" or "village*" or "household*" or "intervention")) or ("panel*" N2 ("data" or "household*" or "model*")) or (("exploit*" or "tak* advantage") N3 ("variation*" or "variety" or "exogen*" or "heterogen*")) or ("econometric" N2 ("model*" or "adjust*")) or ("select*" N2 ("bias*" or "self")) or ("experiment*" N2 ("design" or "study" or "research" or "evaluation" or "evidence" or "vary" or "varies" or "variation")) or (("random" or "randomi#ed" or "randomly") N2 ("trial" or "assign*" or "treatment" or "control*" or "allocat*" or "experiment*" or "vary" or "varies" or "variation" or "choose" or "chose*" or model*)) or (("impact#" or "effect*") N5 ("evaluat*" or "assess" or "assessing" or "assessment*" or "analyze*" or "analyse*" or "analyzing" or "analysing" or "analysis" or "analyses" or "analytical" or "estimate*" or "estimating" or "estimation*" or "examin*" or "quantif*" or "investigat*" or "cause" or "causes" or "causal" or "causation" or "causatively" or "association#" or "associate*" or "hypothesi*" or "produce*" or "production*" or "food" or "crop" or "crops" or "disease" or "infection*" or "health" or "economic" or "price" or "prices" or "markets" or "socioeconomic" or "migration")) OR (("association*") N5 ("evaluat*" or "assess" or "assessing" or "assessment*" or "analyze*" or "analyse*" or "analyzing" or "analysing" or "analysis" or "analyses" or "analytical" or "estimate*" or "estimating" or "estimation*" or "examin*" or "quantif*" or "investigat*" or "cause" or "causal" or "causation" or "causatively" or "hypothesi*")) or "spatial correlation" or "spatial temporal" or "inciden* rate#" or "inciden* ratio#" or "rate ratio#" or (("quant*" OR "effect#" OR "pattern#") N5 ("association" OR "associated")) OR (("composite" or "component*" or "spatial*" or "variabilit*" or "function" or "wavelet" or "correlation*" or "statistical*" or "economi*" or "macroeconomi*" or "empirical*") N5 ("analys*" or "analyz*")) ) ) OR KW ( ( ("match*" N2 ("propensity" or "coarsened" or "covariate" or "co-variate" or "neighbo#r")) or "propensity score" or "difference* in difference*" or "difference-in-difference*" or "differences-in-difference*" or "double difference*" or "quasi-experiment*" or "quasi experiment*" or ("estimat*" and "evaluat*") or "instrumental variable*" or ("IV" N2 ("estimation" or "approach*")) or ("Heckman" N3 ("model*" or "approach*")) or (("two-stage" or "two stage") N3 ("control*" or "function*" or "least squares")) or "regression discontinuity" or "time series" or "counterfactual" or "segment* regression" or "coefficient of variation" or ("non" N2 "participant*") or (("control" or "comparison") N2 ("group*" or "condition*" or "area*" or "village*" or "household*" or "intervention")) or ("panel*" N2 ("data" or "household*" or "model*")) or (("exploit*" or "tak* advantage") N3 ("variation*" or "variety" or "exogen*" or "heterogen*")) or ("econometric" N2 ("model*" or "adjust*")) or ("select*" N2 ("bias*" or "self")) or ("experiment*" N2 ("design" or "study" or "research" or "evaluation" or "evidence" or "vary" or "varies" or "variation")) or (("random" or "randomi#ed" or "randomly") N2 ("trial" or "assign*" or "treatment" or "control*" or "allocat*" or "experiment*" or "vary" or "varies" or "variation" or "choose" or "chose*" or model*)) or (("impact#" or "effect*") N5 ("evaluat*" or "assess" or "assessing" or "assessment*" or "analyze*" or "analyse*" or "analyzing" or "analysing" or "analysis" or "analyses" or "analytical" or "estimate*" or "estimating" or "estimation*" or "examin*" or "quantif*" or "investigat*" or "cause" or "causes" or "causal" or "causation" or "causatively" or "association#" or "associate*" or "hypothesi*" or "produce*" or "production*" or "food" or "crop" or "crops" or "disease" or "infection*" or "health" or "economic" or "price" or "prices" or "markets" or "socioeconomic" or "migration")) OR (("association*") N5 ("evaluat*" or "assess" or "assessing" or "assessment*" or "analyze*" or "analyse*" or "analyzing" or "analysing" or "analysis" or "analyses" or "analytical" or "estimate*" or "estimating" or "estimation*" or "examin*" or "quantif*" or "investigat*" or "cause" or "causal" or "causation" or "causatively" or "hypothesi*")) or "spatial correlation" or "spatial temporal" or "inciden* rate#" or "inciden* ratio#" or "rate ratio#" or (("quant*" OR "effect#" OR "pattern#") N5 ("association" OR "associated")) OR (("composite" or "component*" or "spatial*" or "variabilit*" or "function" or "wavelet" or "correlation*" or "statistical*" or "economi*" or "macroeconomi*" or "empirical*") N5 ("analys*" or "analyz*")) ) ) OR SU ( ( ("match*" N2 ("propensity" or "coarsened" or "covariate" or "co-variate" or "neighbo#r")) or "propensity score" or "difference* in difference*" or "difference-in-difference*" or "differences-in-difference*" or "double difference*" or "quasi-experiment*" or "quasi experiment*" or ("estimat*" and "evaluat*") or "instrumental variable*" or ("IV" N2 ("estimation" or "approach*")) or ("Heckman" N3 ("model*" or "approach*")) or (("two-stage" or "two stage") N3 ("control*" or "function*" or "least squares")) or "regression discontinuity" or "time series" or "counterfactual" or "segment* regression" or "coefficient of variation" or ("non" N2 "participant*") or (("control" or "comparison") N2 ("group*" or "condition*" or "area*" or "village*" or "household*" or "intervention")) or ("panel*" N2 ("data" or "household*" or "model*")) or (("exploit*" or "tak* advantage") N3 ("variation*" or "variety" or "exogen*" or "heterogen*")) or ("econometric" N2 ("model*" or "adjust*")) or ("select*" N2 ("bias*" or "self")) or ("experiment*" N2 ("design" or "study" or "research" or "evaluation" or "evidence" or "vary" or "varies" or "variation")) or (("random" or "randomi#ed" or "randomly") N2 ("trial" or "assign*" or "treatment" or "control*" or "allocat*" or "experiment*" or "vary" or "varies" or "variation" or "choose" or "chose*" or model*)) or (("impact#" or "effect*") N5 ("evaluat*" or "assess" or "assessing" or "assessment*" or "analyze*" or "analyse*" or "analyzing" or "analysing" or "analysis" or "analyses" or "analytical" or "estimate*" or "estimating" or "estimation*" or "examin*" or "quantif*" or "investigat*" or "cause" or "causes" or "causal" or "causation" or "causatively" or "association#" or "associate*" or "hypothesi*" or "produce*" or "production*" or "food" or "crop" or "crops" or "disease" or "infection*" or "health" or "economic" or "price" or "prices" or "markets" or "socioeconomic" or "migration")) OR (("association*") N5 ("evaluat*" or "assess" or "assessing" or "assessment*" or "analyze*" or "analyse*" or "analyzing" or "analysing" or "analysis" or "analyses" or "analytical" or "estimate*" or "estimating" or "estimation*" or "examin*" or "quantif*" or "investigat*" or "cause" or "causal" or "causation" or "causatively" or "hypothesi*")) or "spatial correlation" or "spatial temporal" or "inciden* rate#" or "inciden* ratio#" or "rate ratio#" or (("quant*" OR "effect#" OR "pattern#") N5 ("association" OR "associated")) OR (("composite" or "component*" or "spatial*" or "variabilit*" or "function" or "wavelet" or "correlation*" or "statistical*" or "economi*" or "macroeconomi*" or "empirical*") N5 ("analys*" or "analyz*")) ) ) | 4,428,352 | Concept 3 – Study designs/ Analysis |
| S8 | S3 AND S6 AND S7 | 1,692 | All Concepts |
| S9 | S3 AND S6 AND S7  Limiters - Publication Date: 19900101-20241231 | 1,690 | Date limit |

**Appendix Table 5. Biosis Citation Index (Web of Science) Search Strategy**

| # | Search Query | Results | Notes |
| --- | --- | --- | --- |
| 1 | TS=("El Niño*" or "El Niño*" or "El Ni˜no*" or "Oceanic Niño Index" or "Oceanic Nino Index" or "southern oscillation index" OR (("sea surface temperature" or "SST*") NEAR/3 ("anomal*" )) OR "SOI index" or "IOD$" or "+IOD" or "IOD+" or "pIOD$" or "ENSO$" or "SSTA$" or "MENSOI" OR ("Indian Ocean" NEAR/5 "Dipole") OR (("nino*" or "Niño*") NEAR/3 ("pacific" or "3.4" or "modoki" or "canonical" or "conventional" or "cold tongue" or "warm pool" or "dateline")) OR ( ("dipole mode index" or "DMI" or "walker cell" or "walker circulation" or "sea surface temperature gradient*") AND ("indian ocean") ) OR "Delayed Oscillator" or "Recharge Oscillator" or "Western Pacific Oscillator" or "Advective-Reflective Oscillator" or "Unified Oscillator" OR "La Niña*" or "La Nina*" ) | 30,399 | Concept 1 - +ENSO and +IOD |
| 2 | TS=("bangladesh" or "bhutan" or "borneo" or "burma" or "cambodia" or "kampuchea" or "khmer republic" or "cook islands" or "fiji" or "guam" or "india" or "indonesia" or "kiribati" or "laos" or "marshal island$" or "malaysia" or "malay$ federation" or "maldives" or "micronesia" or "myanmar" or "nauru" or "nepal" or "Pleasant Island" or "northern mariana islands" or "new guinea" or "Oceania" or "palau" or "philippines" or "philipines" or "phillipines" or "phillippines" or "pilipinas" or "pacific islands" or "polynesia" or "samoa" or "samoan islands" or "navigator island" or "navigator islands" or "melanesia" or "solomon island$" or "norfolk island$" or "santa cruz islands" or "sri lanka" or "ceylon" or "tahiti" or "thailand" or "siam" or "timor" or "tonga" or "tuvalu" or "ellice islands" or "vanuatu" or "vietnam" or "viet nam" or "west indies" or "bangladeshi$" or "bangalees" or "bajan$" or "bhutanese" or "bornean$" or "burmese" or "cambodian$" or "cook islander$" or "fijian$" or "guamanian$" or "indonesian$" or "kirabatian$" or "lao" or "laotian$" or "malaysian$" or "maldivian$" or "marshallese" or "melanesian" or "micronesian$" or "myanma" or "nepali$" or "nepalese" or "mariana islander$" or "mariana$" or "chamorros" or "nauruan$" or "norfolk islander$" or "oceanian" or "palauan$" or "papua new guinean$" or "philippine$" or "philipine$" or "phillipine$" or "phillippine$" or "filipino$" or "filipina$" or "pacific islander$" or "polynesian$" or "samoan$" or "solomon islander$" or "sri lankan$" or "ceylonese" or "tahitian$" or "thai" or "timorese$" or "tongan$" or "tuvaluan$" or "vanuatuan$" OR "vietnamese") OR CU=("bangladesh" or "bhutan" or "borneo" or "burma" or "cambodia" or "kampuchea" or "khmer republic" or "cook islands" or "fiji" or "guam" or "india" or "indonesia" or "kiribati" or "laos" or "marshal island$" or "malaysia" or "malay$ federation" or "maldives" or "micronesia" or "myanmar" or "nauru" or "nepal" or "Pleasant Island" or "northern mariana islands" or "new guinea" or "Oceania" or "palau" or "philippines" or "philipines" or "phillipines" or "phillippines" or "pilipinas" or "pacific islands" or "polynesia" or "samoa" or "samoan islands" or "navigator island" or "navigator islands" or "melanesia" or "solomon island$" or "norfolk island$" or "santa cruz islands" or "sri lanka" or "ceylon" or "tahiti" or "thailand" or "siam" or "timor" or "tonga" or "tuvalu" or "ellice islands" or "vanuatu" or "vietnam" or "viet nam" or "west indies" or "bangladeshi$" or "bangalees" or "bajan$" or "bhutanese" or "bornean$" or "burmese" or "cambodian$" or "cook islander$" or "fijian$" or "guamanian$" or "indonesian$" or "kirabatian$" or "lao" or "laotian$" or "malaysian$" or "maldivian$" or "marshallese" or "melanesian" or "micronesian$" or "myanma" or "nepali$" or "nepalese" or "mariana islander$" or "mariana$" or "chamorros" or "nauruan$" or "norfolk islander$" or "oceanian" or "palauan$" or "papua new guinean$" or "philippine$" or "philipine$" or "phillipine$" or "phillippine$" or "filipino$" or "filipina$" or "pacific islander$" or "polynesian$" or "samoan$" or "solomon islander$" or "sri lankan$" or "ceylonese" or "tahitian$" or "thai" or "timorese$" or "tongan$" or "tuvaluan$" or "vanuatuan$" or "vietnamese") | 1,147,025 | Concept 2 -Countries/ regions |
| 3 | TS=(("indian ocean" or "pacific ocean" or "indo pacific" or "indo-pacific" or "indopacific*" or "north pacific" or "tropical pacific" or "equatorial pacific" or "south pacific" or "indochina" or "indochinese" ) NEAR/3 ("adjacent" or "border*" or "country" or "countries" or "region" or "regions" or "island" or "islands" or "nation" or "nations" or "economies")) | 196,415 |  |
| 4 | #2 OR #3 | 1,298,311 |  |
| 5 | TS=( ("match*" NEAR/2 ("propensity" or "coarsened" or "covariate" or "co-variate" or "neighbo$r")) or "propensity score" or "difference* in difference*" or "difference-in-difference*" or "differences-in-difference*" or "double difference*" or "quasi-experiment*" or "quasi experiment*" or ("estimat*" and "evaluat*") or "instrumental variable*" or ("IV" NEAR/2 ("estimation" or "approach*")) or ("Heckman" NEAR/3 ("model*" or "approach*")) or (("two-stage" or "two stage") NEAR/3 ("control*" or "function*" or "least squares")) or "regression discontinuity" or "time series" or "counterfactual" or "segment* regression" or "coefficient of variation" or ("non" NEAR/2 "participant*") or (("control" or "comparison") NEAR/2 ("group*" or "condition*" or "area*" or "village*" or "household*" or "intervention")) or ("panel*" NEAR/2 ("data" or "household*" or "model*")) or (("exploit*" or "tak* advantage") NEAR/3 ("variation*" or "variety" or "exogen*" or "heterogen*")) or ("econometric" NEAR/2 ("model*" or "adjust*")) or ("select*" NEAR/2 ("bias*" or "self")) OR ("experiment*" NEAR/2 ("design" or "study" or "research" or "evaluation" or "evidence" or "vary" or "varies" or "variation")) or (("random" or "randomi$ed" or "randomly") NEAR/2 ("trial" or "assign*" or "treatment" or "control*" or "allocat*" or "experiment*" or "vary" or "varies" or "variation" or "choose" or "chose*" or model*)) or (("impact$" or "effect*") NEAR/5 ("evaluat*" or "assess" or "assessing" or "assessment*" or "analyze*" or "analyse*" or "analyzing" or "analysing" or "analysis" or "analyses" or "analytical" or "estimate*" or "estimating" or "estimation*" or "examin*" or "quantif*" or "investigat*" or "cause" or "causes" or "causal" or "causation" or "causatively" or "association$" or "associate*" or "hypothesi*" or "produce*" or "production*" or "food" or "crop" or "crops" or "disease" or "infection*" or "health" or "economic" or "price" or "prices" or "markets" or "socioeconomic" or "migration")) or (("association") NEAR/5 ("evaluat*" or "assess" or "assessing" or "assessment" or "analyze*" or "analyse*" or "analyzing" or "analysing" or "analysis" or "analyses" or "analytical" or "estimate*" or "estimating" or "estimation*" or "examin*" or "quantif*" or "investigat*" or "cause" or "causes" or "causal" or "causation" or "causatively" or "hypothesi*")) or "spatial correlation" or "spatial temporal" or "inciden* rate$" or "inciden* ratio$" or "rate ratio$" or (("quant*" OR "effect$" OR "pattern$") NEAR/5 ("association" OR "associated")) OR (("composite" or "component*" or "spatial*" or "variabilit*" or "function" or "correlation*" or "wavelet" or "statistical*" or "economi*" or "macroeconomi*" or "empirical*") NEAR/5 ("analys*" or "analyz*")) ) | 3,692,607 | Concept 3 – Study designs/ Analysis |
| 6 | #1 AND #4 AND #5 | 738 | All concepts |
| 7 | #5 AND #4 AND #1 and 2023 or 2022 or 2021 or 2020 or 2019 or 2018 or 2017 or 2016 or 2015 or 2014 or 2013 or 2012 or 2011 or 2010 or 2009 or 2008 or 2007 or 2006 or 2005 or 2004 or 2003 or 2002 or 2001 or 2000 or 1999 or 1998 or 1997 or 1996 or 1995 or 1994 or 1993 or 1992 or 1991 or 1990 (Publication Years) | 728 | Date limit |

**Appendix Table 6. Medline All (Ovid) Search Strategy**

| **#** | **Query** | **Results** | **Notes** |
| --- | --- | --- | --- |
| 1 | El Niño-Southern Oscillation/ | 577 | Concept 1 – +ENSO and +IOD |
| 2 | ("El Niño*" or "El Niño*" or "El Ni˜no*" or "Oceanic Niño Index" or "Oceanic Nino Index" or "southern oscillation index" or (("sea surface temperature" or "SST*") adj3 "anomal*") or "SOI index" or "IOD?" or "+IOD" or "IOD+" or "pIOD?" or "ENSO?" or "SSTA?" or "MENSOI" or ("Indian Ocean" adj5 "Dipole") or (("nino*" or "Niño*") adj3 (pacific or "3 4" or "modoki" or "canonical" or "conventional" or "cold tongue" or "warm pool" or "dateline")) or (("dipole mode index" or "DMI" or "walker cell" or "walker circulation" or "surface sea temperature gradient") and "indian ocean") or "Delayed Oscillator" or "Recharge Oscillator" or "Western Pacific Oscillator" or "Advective-Reflective Oscillator" or "Unified Oscillator" or "La Niña*" or "La Nina*").ti,ab,ot,kf,hw. | 14,476 |  |
| 3 | 1 or 2 | 14,476 |  |
| 4 | borneo/ or cambodia/ or indonesia/ or laos/ or malaysia/ or myanmar/ or philippines/ or thailand/ or timor-leste/ or vietnam/ or bangladesh/ or bhutan/ or india/ or maldives/ or nepal/ or sri lanka/ or indian ocean islands/ or melanesia/ or fiji/ or papua new guinea/ or vanuatu/ or micronesia/ or guam/ or palau/ or polynesia/ or exp samoa/ or tonga/ or pacific islands/ or indian ocean/ or pacific ocean/ | 266,216 | Concept 2 – Countries/ regions |
| 5 | ("bangladesh" or "bhutan" or "borneo" or "burma" or "cambodia" or "kampuchea" or "khmer republic" or "cook islands" or "fiji" or "guam" or "india" or "indonesia" or "kiribati" or "laos" or "marshal island?" or "malaysia" or "malay? federation" or "maldives" or "melanesia" or "micronesia" or "myanmar" or "nauru" or "nepal" or "Pleasant Island" or "northern mariana islands" or "new guinea" or "Oceania" or "palau" or "philippines" or "philipines" or "phillipines" or "phillippines" or "pilipinas" or "pacific islands" or "polynesia" or "samoa" or "samoan islands" or "navigator island" or "navigator islands" or "solomon island?" or "norfolk island?" or "santa cruz island?" or "sri lanka" or "ceylon" or "thailand" or "siam" or "timor" or "tonga" or "tahiti" or "tuvalu" or "ellice islands" or "vanuatu" or "vietnam" or "viet nam" or "west indies" or "bangladeshi?" or "bangalees" or "bajan?" or "bhutanese" or "bornean?" or "burmese" or "cambodian?" or "cook islander?" or "fijian?" or "guamanian?" or "indonesian?" or "kirabatian?" or "lao" or "laotian?" or "malaysian?" or "maldivian?" or "marshallese" or "melanesian" or "micronesian?" or "myanma" or "nepali?" or "nepalese" or "northern mariana islander?" or "mariana?" or "chamorros" or "nauruan?" or "norfolk islander?" or "oceanian" or "palauan?" or "papua new guinean?" or "philippine?" or "philipine?" or "phillipine?" or "phillippine?" or "filipino?" or "filipina?" or "pacific islander?" or "polynesian?" or "samoan?" or "solomon islander?" or "sri lankan?" or "ceylonese" or "tahitian?" or "thai" or "timorese?" or "tongan?" or "tuvaluan?" or "vanuatuan?" or "vietnamese").ti,ab,ot,kf,hw. | 429,957 |  |
| 6 | ("north* pacific ocean*" or "tropical pacific ocean*" or "equatorial pacific ocean*" or "south* pacific ocean*" or "mekong delta?" or "ganges delta?" or "ayeyarwady delta?" or "arabian sea" or "andaman sea" or "bay of Bengal" or "north indian ocean" or "southwestern indian ocean" or "south china sea?" or "indian subcontinent*" or ("countries" and ("the world" or "worldwide" or "global*")) or ("southeastern" adj2 "asia") or ("south eastern" adj2 "asia") or "southeast asia" or "south east asia" or "south asia" or (("indian ocean" or "pacific ocean" or "indo pacific" or "indo-pacific" or "indopacific*" or "north pacific" or "tropical pacific" or "equatorial pacific" or "south pacific" or "indochina" or "indochinese") adj4 ("adjacent" or "border*" or "country" or "countries" or "region" or "regions" or "island" or "islands" or "nation" or "nations" or "economies"))).ti,ab,ot,kf,hw. | 197,134 |  |
| 7 | 4 or 5 or 6 | 596,713 |  |
| 8 | (("match*" adj2 ("propensity" or "coarsened" or "covariate" or "co-variate" or "neighbo?r")) or "propensity score" or "difference* in difference*" or "difference-in-difference*" or "differences-in-difference*" or "double difference*" or "quasi-experiment*" or "quasi experiment*" or ("estimat*" and "evaluat*") or "instrumental variable*" or ("IV" adj3 ("estimation" or "approach*")) or ("Heckman" adj4 ("model*" or "approach*")) or (("two-stage" or "two stage") adj4 ("control*" or "function*" or "least squares")) or "regression discontinuity" or "time series" or "counterfactual" or "segment* regression" or "coefficient of variation" or ("non" adj3 "participant*") or (("control" or "comparison") adj3 ("group*" or "condition*" or "area*" or "village*" or "household*" or "intervention")) or ("panel*" adj3 ("data" or "household*" or "model*")) or (("exploit*" or "tak* advantage") adj4 ("variation*" or "variety" or "exogen*" or "heterogen*")) or ("econometric" adj3 ("model*" or "adjust*")) or ("select*" adj3 ("bias*" or "self")) or ("experiment*" adj3 ("design" or "study" or "research" or "evaluation" or "evidence" or "vary" or "varies" or "variation")) or (("random" or "randomi?ed" or "randomly") adj3 ("trial" or "assign*" or "treatment" or "control*" or "allocat*" or "experiment*" or "vary" or "varies" or "variation" or "choose" or "chose*" or model*))).ti,ab,ot,kf,hw. | 2,527,996 | Concept 3 – Study designs/ Analysis |
| 9 | ((("impact?" or "effect*") adj6 ("evaluat*" or "assess" or "assessing" or "assessment*" or "analyze*" or "analyse*" or "analyzing" or "analysing" or "analysis" or "analyses" or "analytical" or "estimate*" or "estimating" or "estimation*" or "examin*" or "quantif*" or "investigat*" or "cause" or "causes" or "causal" or "causation" or "causatively" or "association?" or "associate*" or "hypothesi*" or "produce*" or "production*" or "food" or "crop" or "crops" or "disease" or "infection*" or "health" or "economic" or "price" or "prices" or "markets" or "socioeconomic" or "migration")) or ("association*" adj6 ("evaluat*" or "assess" or "assessing" or "assessment*" or "analyze*" or "analyse*" or "analyzing" or "analysing" or "analysis" or "analyses" or "analytical" or "estimate*" or "estimating" or "estimation*" or "examin*" or "quantif*" or "investigat*" or "cause" or "causal" or "causation" or "causatively" or "hypothesi*")) or "spatial correlation" or "spatial temporal" or "inciden* rate?" or "inciden* ratio?" or "rate ratio?" or (("quant*" or "effect?" or "pattern?") adj6 ("association" or "associated")) or (("composite" or "component*" or "spatial*" or "variabilit*" or "function" or "wavelet" or "correlation*" or "statistical*" or "economi*" or "macroeconomi*" or "empirical*") adj6 ("analys*" or "analyz*"))).ti,ab,ot,kf,hw. | 3,913,440 |  |
| 10 | 8 or 9 | 5,698,143 |  |
| 11 | 3 and 7 and 10 | 250 | All concepts |
| 12 | limit 11 to yr="1990 - 2024" | 245 | Date limit |

**Appendix Table 7. CAB Abstracts (Ovid) Search Strategy**

| **#** | **Query** | **Results** | **Notes** |
| --- | --- | --- | --- |
| 1 | "El Niño-Southern Oscillation".sh. | 5,413 | Concept 1 - +ENSO and +IOD |
| 2 | ("El Niño*" or "El Niño*" or "El Ni˜no*" or "Oceanic Niño Index" or "Oceanic Nino Index" or "southern oscillation index" or (("sea surface temperature" or "SST*") adj3 "anomal*") or "SOI index" or "IOD?" or "+IOD" or "IOD+" or "pIOD?" or "ENSO?" or "SSTA?" or "MENSOI" or ("Indian Ocean" adj5 "Dipole") or (("nino*" or "Niño*") adj3 (pacific or "3 4" or "modoki" or "canonical" or "conventional" or "cold tongue" or "warm pool" or "dateline")) or (("dipole mode index" or "DMI" or "walker cell" or "walker circulation" or "surface sea temperature gradient") and "indian ocean") or "Delayed Oscillator" or "Recharge Oscillator" or "Western Pacific Oscillator" or "Advective-Reflective Oscillator" or "Unified Oscillator" or "La Niña*" or "La Nina*").ti,ab,ot,hw,gl. | 11,948 |  |
| 3 | 1 or 2 | 11,948 |  |
| 4 | ("bangladesh" or "bhutan" or "borneo" or "burma" or "cambodia" or "kampuchea" or "khmer republic" or "cook islands" or "fiji" or "guam" or "india" or "indonesia" or "kiribati" or "laos" or "marshal island?" or "malaysia" or "malay? federation" or "maldives" or "melanesia" or "micronesia" or "myanmar" or "nauru" or "nepal" or "Pleasant Island" or "northern mariana islands" or "new guinea" or "Oceania" or "palau" or "philippines" or "philipines" or "phillipines" or "phillippines" or "pilipinas" or "pacific islands" or "polynesia" or "samoa" or "samoan islands" or "navigator island" or "navigator islands" or "solomon island?" or "norfolk island?" or "santa cruz island?" or "sri lanka" or "ceylon" or "thailand" or "siam" or "timor" or "tonga" or "tahiti" or "tuvalu" or "ellice islands" or "vanuatu" or "vietnam" or "viet nam" or "west indies" or "bangladeshi?" or "bangalees" or "bajan?" or "bhutanese" or "bornean?" or "burmese" or "cambodian?" or "cook islander?" or "fijian?" or "guamanian?" or "indonesian?" or "kirabatian?" or "lao" or "laotian?" or "malaysian?" or "maldivian?" or "marshallese" or "melanesian" or "micronesian?" or "myanma" or "nepali?" or "nepalese" or "northern mariana islander?" or "mariana?" or "chamorros" or "nauruan?" or "norfolk islander?" or "oceanian" or "palauan?" or "papua new guinean?" or "philippine?" or "philipine?" or "phillipine?" or "phillippine?" or "filipino?" or "filipina?" or "pacific islander?" or "polynesian?" or "samoan?" or "solomon islander?" or "sri lankan?" or "ceylonese" or "tahitian?" or "thai" or "timorese?" or "tongan?" or "tuvaluan?" or "vanuatuan?" or "vietnamese").ti,ab,ot,hw,gl. | 865,114 | Concept 2 – Countries/ regions |
| 5 | ("north* pacific ocean*" or "tropical pacific ocean*" or "equatorial pacific ocean*" or "south* pacific ocean*" or "mekong delta?" or "ganges delta?" or "ayeyarwady delta?" or "arabian sea" or "andaman sea" or "bay of Bengal" or "north indian ocean" or "southwestern indian ocean" or "south china sea?" or "indian subcontinent*" or ("countries" adj10 ("the world" or "worldwide" or "global*")) or ("southeastern" adj2 "asia") or ("south eastern" adj2 "asia") or "southeast asia" or "south east asia" or "south asia" or (("indian ocean" or "pacific ocean" or "indo pacific" or "indo-pacific" or "indopacific*" or "north pacific" or "tropical pacific" or "equatorial pacific" or "south pacific" or "indochina" or "indochinese") adj4 ("adjacent" or "border*" or "country" or "countries" or "region" or "regions" or "island" or "islands" or "nation" or "nations" or "economies"))).ti,ab,ot,hw,gl. | 796,581 |  |
| 6 | 4 or 5 | 1,021,147 |  |
| 7 | (("match*" adj2 ("propensity" or "coarsened" or "covariate" or "co-variate" or "neighbo?r")) or "propensity score" or "difference* in difference*" or "difference-in-difference*" or "differences-in-difference*" or "double difference*" or "quasi-experiment*" or "quasi experiment*" or ("estimat*" and "evaluat*") or "instrumental variable*" or ("IV" adj3 ("estimation" or "approach*")) or ("Heckman" adj4 ("model*" or "approach*")) or (("two-stage" or "two stage") adj4 ("control*" or "function*" or "least squares")) or "regression discontinuity" or "time series" or "counterfactual" or "segment* regression" or "coefficient of variation" or ("non" adj3 "participant*") or (("control" or "comparison") adj3 ("group*" or "condition*" or "area*" or "village*" or "household*" or "intervention")) or ("panel*" adj3 ("data" or "household*" or "model*")) or (("exploit*" or "tak* advantage") adj4 ("variation*" or "variety" or "exogen*" or "heterogen*")) or ("econometric" adj3 ("model*" or "adjust*")) or ("select*" adj3 ("bias*" or "self")) or ("experiment*" adj3 ("design" or "study" or "research" or "evaluation" or "evidence" or "vary" or "varies" or "variation")) or (("random" or "randomi?ed" or "randomly") adj3 ("trial" or "assign*" or "treatment" or "control*" or "allocat*" or "experiment*" or "vary" or "varies" or "variation" or "choose" or "chose*" or model*))).ti,ab,ot,hw,gl. | 680,196 | Concept 3 – Study designs/ Analysis |
| 8 | ((("impact?" or "effect*") adj6 ("evaluat*" or "assess" or "assessing" or "assessment*" or "analyze*" or "analyse*" or "analyzing" or "analysing" or "analysis" or "analyses" or "analytical" or "estimate*" or "estimating" or "estimation*" or "examin*" or "quantif*" or "investigat*" or "cause" or "causes" or "causal" or "causation" or "causatively" or "association?" or "associate*" or "hypothesi*" or "produce*" or "production*" or "food" or "crop" or "crops" or "disease" or "infection*" or "health" or "economic" or "price" or "prices" or "markets" or "socioeconomic" or "migration")) or ("association*" adj6 ("evaluat*" or "assess" or "assessing" or "assessment*" or "analyze*" or "analyse*" or "analyzing" or "analysing" or "analysis" or "analyses" or "analytical" or "estimate*" or "estimating" or "estimation*" or "examin*" or "quantif*" or "investigat*" or "cause" or "causal" or "causation" or "causatively" or "hypothesi*")) or "spatial correlation" or "spatial temporal" or "inciden* rate?" or "inciden* ratio?" or "rate ratio?" or (("quant*" or "effect?" or "pattern?") adj6 ("association" or "associated")) or (("composite" or "component*" or "spatial*" or "variabilit*" or "function" or "wavelet" or "correlation*" or "statistical*" or "economi*" or "macroeconomi*" or "empirical*") adj6 ("analys*" or "analyz*"))).ti,ab,ot,hw,gl. | 1,733,197 |  |
| 9 | 7 or 8 | 2,161,134 |  |
| 10 | 3 and 6 and 9 | 1,383 | All concepts |
| 11 | limit 10 to yr="1990 - 2024" | 1,383 | Date limit |

**Appendix Table 8. AGRICOLA (Ovid) Search Strategy**

| **#** | **Query** | **Results** | **Notes** |
| --- | --- | --- | --- |
| 1 | "El Niño"/ | 4,255 | Concept 1 - +ENSO and +IOD |
| 2 | ("El Niño*" or "El Niño*" or "El Ni˜no*" or "Oceanic Niño Index" or "Oceanic Nino Index" or "southern oscillation index" or (("sea surface temperature" or "SST*") adj3 "anomal*") or "SOI index" or "IOD?" or "+IOD" or "IOD+" or "pIOD?" or "ENSO?" or "SSTA?" or "MENSOI" or ("Indian Ocean" adj5 "Dipole") or (("nino*" or "Niño*") adj3 (pacific or "3 4" or "modoki" or "canonical" or "conventional" or "cold tongue" or "warm pool" or "dateline")) or (("dipole mode index" or "DMI" or "walker cell" or "walker circulation" or "surface sea temperature gradient") and "indian ocean") or "Delayed Oscillator" or "Recharge Oscillator" or "Western Pacific Oscillator" or "Advective-Reflective Oscillator" or "Unified Oscillator" or "La Niña*" or "La Nina*").ti,ab,ot,hw,de. | 8,290 |  |
| 3 | 1 or 2 | 8,290 |  |
| 4 | ("bangladesh" or "bhutan" or "borneo" or "burma" or "cambodia" or "kampuchea" or "khmer republic" or "cook islands" or "fiji" or "guam" or "india" or "indonesia" or "kiribati" or "laos" or "marshal island?" or "malaysia" or "malay? federation" or "maldives" or "melanesia" or "micronesia" or "myanmar" or "nauru" or "nepal" or "Pleasant Island" or "northern mariana islands" or "new guinea" or "Oceania" or "palau" or "philippines" or "philipines" or "phillipines" or "phillippines" or "pilipinas" or "pacific islands" or "polynesia" or "samoa" or "samoan islands" or "navigator island" or "navigator islands" or "solomon island?" or "norfolk island?" or "santa cruz island?" or "sri lanka" or "ceylon" or "thailand" or "siam" or "timor" or "tonga" or "tahiti" or "tuvalu" or "ellice islands" or "vanuatu" or "vietnam" or "viet nam" or "west indies" or "bangladeshi?" or "bangalees" or "bajan?" or "bhutanese" or "bornean?" or "burmese" or "cambodian?" or "cook islander?" or "fijian?" or "guamanian?" or "indonesian?" or "kirabatian?" or "lao" or "laotian?" or "malaysian?" or "maldivian?" or "marshallese" or "melanesian" or "micronesian?" or "myanma" or "nepali?" or "nepalese" or "northern mariana islander?" or "mariana?" or "chamorros" or "nauruan?" or "norfolk islander?" or "oceanian" or "palauan?" or "papua new guinean?" or "philippine?" or "philipine?" or "phillipine?" or "phillippine?" or "filipino?" or "filipina?" or "pacific islander?" or "polynesian?" or "samoan?" or "solomon islander?" or "sri lankan?" or "ceylonese" or "tahitian?" or "thai" or "timorese?" or "tongan?" or "tuvaluan?" or "vanuatuan?" or "vietnamese").ti,ab,ot,hw,de. | 169,223 | Concept 2 – Countries/ regions |
| 5 | ("north* pacific ocean*" or "tropical pacific ocean*" or "equatorial pacific ocean*" or "south* pacific ocean*" or "mekong delta?" or "ganges delta?" or "ayeyarwady delta?" or "arabian sea" or "andaman sea" or "bay of Bengal" or "north indian ocean" or "southwestern indian ocean" or "south china sea?" or "indian subcontinent*" or ("countries" adj10 ("the world" or "worldwide" or "global*")) or ("southeastern" adj2 "asia") or ("south eastern" adj2 "asia") or "southeast asia" or "south east asia" or "south asia" or (("indian ocean" or "pacific ocean" or "indo pacific" or "indo-pacific" or "indopacific*" or "north pacific" or "tropical pacific" or "equatorial pacific" or "south pacific" or "indochina" or "indochinese") adj4 ("adjacent" or "border*" or "country" or "countries" or "region" or "regions" or "island" or "islands" or "nation" or "nations" or "economies"))).ti,ab,ot,hw,de. | 34,662 |  |
| 6 | 4 or 5 | 191,657 |  |
| 7 | (("match*" adj2 ("propensity" or "coarsened" or "covariate" or "co-variate" or "neighbo?r")) or "propensity score" or "difference* in difference*" or "difference-in-difference*" or "differences-in-difference*" or "double difference*" or "quasi-experiment*" or "quasi experiment*" or ("estimat*" and "evaluat*") or "instrumental variable*" or ("IV" adj3 ("estimation" or "approach*")) or ("Heckman" adj4 ("model*" or "approach*")) or (("two-stage" or "two stage") adj4 ("control*" or "function*" or "least squares")) or "regression discontinuity" or "time series" or "counterfactual" or "segment* regression" or "coefficient of variation" or ("non" adj3 "participant*") or (("control" or "comparison") adj3 ("group*" or "condition*" or "area*" or "village*" or "household*" or "intervention")) or ("panel*" adj3 ("data" or "household*" or "model*")) or (("exploit*" or "tak* advantage") adj4 ("variation*" or "variety" or "exogen*" or "heterogen*")) or ("econometric" adj3 ("model*" or "adjust*")) or ("select*" adj3 ("bias*" or "self")) or ("experiment*" adj3 ("design" or "study" or "research" or "evaluation" or "evidence" or "vary" or "varies" or "variation")) or (("random" or "randomi?ed" or "randomly") adj3 ("trial" or "assign*" or "treatment" or "control*" or "allocat*" or "experiment*" or "vary" or "varies" or "variation" or "choose" or "chose*" or model*))).ti,ab,ot,hw,de. | 306,164 | Concept 3 – Study designs/ Analysis |
| 8 | ((("impact?" or "effect*") adj6 ("evaluat*" or "assess" or "assessing" or "assessment*" or "analyze*" or "analyse*" or "analyzing" or "analysing" or "analysis" or "analyses" or "analytical" or "estimate*" or "estimating" or "estimation*" or "examin*" or "quantif*" or "investigat*" or "cause" or "causes" or "causal" or "causation" or "causatively" or "association?" or "associate*" or "hypothesi*" or "produce*" or "production*" or "food" or "crop" or "crops" or "disease" or "infection*" or "health" or "economic" or "price" or "prices" or "markets" or "socioeconomic" or "migration")) or ("association*" adj6 ("evaluat*" or "assess" or "assessing" or "assessment*" or "analyze*" or "analyse*" or "analyzing" or "analysing" or "analysis" or "analyses" or "analytical" or "estimate*" or "estimating" or "estimation*" or "examin*" or "quantif*" or "investigat*" or "cause" or "causal" or "causation" or "causatively" or "hypothesi*")) or "spatial correlation" or "spatial temporal" or "inciden* rate?" or "inciden* ratio?" or "rate ratio?" or (("quant*" or "effect?" or "pattern?") adj6 ("association" or "associated")) or (("composite" or "component*" or "spatial*" or "variabilit*" or "function" or "wavelet" or "correlation*" or "statistical*" or "economi*" or "macroeconomi*" or "empirical*") adj6 ("analys*" or "analyz*"))).ti,ab,ot,hw,de. | 827,501 |  |
| 9 | 7 or 8 | 1,027,876 |  |
| 10 | 3 and 6 and 9 | 582 | All concepts |
| 11 | limit 10 to yr="1990 - 2024" | 582 | Date limit |

**Appendix Table 9. Global Health (Ovid) Search Strategy**

| **#** | **Query** | **Results** | **Notes** |
| --- | --- | --- | --- |
| 1 | "El Niño-Southern Oscillation".sh. | 186 | Concept 1 - +ENSO and +IOD |
| 2 | ("El Niño*" or "El Niño*" or "El Ni˜no*" or "Oceanic Niño Index" or "Oceanic Nino Index" or "southern oscillation index" or (("sea surface temperature" or "SST*") adj3 "anomal*") or "SOI index" or "IOD?" or "+IOD" or "IOD+" or "pIOD?" or "ENSO?" or "SSTA?" or "MENSOI" or ("Indian Ocean" adj5 "Dipole") or (("nino*" or "Niño*") adj3 (pacific or "3 4" or "modoki" or "canonical" or "conventional" or "cold tongue" or "warm pool" or "dateline")) or (("dipole mode index" or "DMI" or "walker cell" or "walker circulation" or "surface sea temperature gradient") and "indian ocean") or "Delayed Oscillator" or "Recharge Oscillator" or "Western Pacific Oscillator" or "Advective-Reflective Oscillator" or "Unified Oscillator" or "La Niña*" or "La Nina*").ti,ab,ot,hw. | 1,205 |  |
| 3 | 1 or 2 | 1,205 |  |
| 4 | ("bangladesh" or "bhutan" or "borneo" or "burma" or "cambodia" or "kampuchea" or "khmer republic" or "cook islands" or "fiji" or "guam" or "india" or "indonesia" or "kiribati" or "laos" or "marshal island?" or "malaysia" or "malay? federation" or "maldives" or "melanesia" or "micronesia" or "myanmar" or "nauru" or "nepal" or "Pleasant Island" or "northern mariana islands" or "new guinea" or "Oceania" or "palau" or "philippines" or "philipines" or "phillipines" or "phillippines" or "pilipinas" or "pacific islands" or "polynesia" or "samoa" or "samoan islands" or "navigator island" or "navigator islands" or "solomon island?" or "norfolk island?" or "santa cruz island?" or "sri lanka" or "ceylon" or "thailand" or "siam" or "timor" or "tonga" or "tahiti" or "tuvalu" or "ellice islands" or "vanuatu" or "vietnam" or "viet nam" or "west indies" or "bangladeshi?" or "bangalees" or "bajan?" or "bhutanese" or "bornean?" or "burmese" or "cambodian?" or "cook islander?" or "fijian?" or "guamanian?" or "indonesian?" or "kirabatian?" or "lao" or "laotian?" or "malaysian?" or "maldivian?" or "marshallese" or "melanesian" or "micronesian?" or "myanma" or "nepali?" or "nepalese" or "northern mariana islander?" or "mariana?" or "chamorros" or "nauruan?" or "norfolk islander?" or "oceanian" or "palauan?" or "papua new guinean?" or "philippine?" or "philipine?" or "phillipine?" or "phillippine?" or "filipino?" or "filipina?" or "pacific islander?" or "polynesian?" or "samoan?" or "solomon islander?" or "sri lankan?" or "ceylonese" or "tahitian?" or "thai" or "timorese?" or "tongan?" or "tuvaluan?" or "vanuatuan?" or "vietnamese").ti,ab,ot,hw,gl. | 356,442 | Concept 2 – Countries/ regions |
| 5 | ("north* pacific ocean*" or "tropical pacific ocean*" or "equatorial pacific ocean*" or "south* pacific ocean*" or "mekong delta?" or "ganges delta?" or "ayeyarwady delta?" or "arabian sea" or "andaman sea" or "bay of Bengal" or "north indian ocean" or "southwestern indian ocean" or "south china sea?" or "indian subcontinent*" or ("countries" adj10 ("the world" or "worldwide" or "global*")) or ("southeastern" adj2 "asia") or ("south eastern" adj2 "asia") or "southeast asia" or "south east asia" or "south asia" or (("indian ocean" or "pacific ocean" or "indo pacific" or "indo-pacific" or "indopacific*" or "north pacific" or "tropical pacific" or "equatorial pacific" or "south pacific" or "indochina" or "indochinese") adj4 ("adjacent" or "border*" or "country" or "countries" or "region" or "regions" or "island" or "islands" or "nation" or "nations" or "economies"))).ti,ab,ot,hw,gl. | 349,352 |  |
| 6 | 4 or 5 | 452,911 |  |
| 7 | (("match*" adj2 ("propensity" or "coarsened" or "covariate" or "co-variate" or "neighbo?r")) or "propensity score" or "difference* in difference*" or "difference-in-difference*" or "differences-in-difference*" or "double difference*" or "quasi-experiment*" or "quasi experiment*" or ("estimat*" and "evaluat*") or "instrumental variable*" or ("IV" adj3 ("estimation" or "approach*")) or ("Heckman" adj4 ("model*" or "approach*")) or (("two-stage" or "two stage") adj4 ("control*" or "function*" or "least squares")) or "regression discontinuity" or "time series" or "counterfactual" or "segment* regression" or "coefficient of variation" or ("non" adj3 "participant*") or (("control" or "comparison") adj3 ("group*" or "condition*" or "area*" or "village*" or "household*" or "intervention")) or ("panel*" adj3 ("data" or "household*" or "model*")) or (("exploit*" or "tak* advantage") adj4 ("variation*" or "variety" or "exogen*" or "heterogen*")) or ("econometric" adj3 ("model*" or "adjust*")) or ("select*" adj3 ("bias*" or "self")) or ("experiment*" adj3 ("design" or "study" or "research" or "evaluation" or "evidence" or "vary" or "varies" or "variation")) or (("random" or "randomi?ed" or "randomly") adj3 ("trial" or "assign*" or "treatment" or "control*" or "allocat*" or "experiment*" or "vary" or "varies" or "variation" or "choose" or "chose*" or model*))).ti,ab,ot,hw. | 420,490 | Concept 3 – Study designs/ Analysis |
| 8 | ((("impact?" or "effect*") adj6 ("evaluat*" or "assess" or "assessing" or "assessment*" or "analyze*" or "analyse*" or "analyzing" or "analysing" or "analysis" or "analyses" or "analytical" or "estimate*" or "estimating" or "estimation*" or "examin*" or "quantif*" or "investigat*" or "cause" or "causes" or "causal" or "causation" or "causatively" or "association?" or "associate*" or "hypothesi*" or "produce*" or "production*" or "food" or "crop" or "crops" or "disease" or "infection*" or "health" or "economic" or "price" or "prices" or "markets" or "socioeconomic" or "migration")) or ("association*" adj6 ("evaluat*" or "assess" or "assessing" or "assessment*" or "analyze*" or "analyse*" or "analyzing" or "analysing" or "analysis" or "analyses" or "analytical" or "estimate*" or "estimating" or "estimation*" or "examin*" or "quantif*" or "investigat*" or "cause" or "causal" or "causation" or "causatively" or "hypothesi*")) or "spatial correlation" or "spatial temporal" or "inciden* rate?" or "inciden* ratio?" or "rate ratio?" or (("quant*" or "effect?" or "pattern?") adj6 ("association" or "associated")) or (("composite" or "component*" or "spatial*" or "variabilit*" or "function" or "wavelet" or "correlation*" or "statistical*" or "economi*" or "macroeconomi*" or "empirical*") adj6 ("analys*" or "analyz*"))).ti,ab,ot,hw. | 955,951 |  |
| 9 | 7 or 8 | 1,200,089 |  |
| 10 | 3 and 6 and 9 | 130 | All concepts |
| 11 | limit 10 to yr="1990 - 2024" | 130 | Date limit |

**Appendix Table 10. EconLit (Ovid) Search Strategy**

| **#** | **Query** | **Results** | **Notes** |
| --- | --- | --- | --- |
| 1 | ("El Niño*" or "El Niño*" or "El Ni˜no*" or "Oceanic Niño Index" or "Oceanic Nino Index" or "southern oscillation index" or (("sea surface temperature" or "SST*") adj3 "anomal*") or "SOI index" or "IOD?" or "+IOD" or "IOD+" or "pIOD?" or "ENSO?" or "SSTA?" or "MENSOI" or ("Indian Ocean" adj5 "Dipole") or (("nino*" or "Niño*") adj3 (pacific or "3 4" or "modoki" or "canonical" or "conventional" or "cold tongue" or "warm pool" or "dateline")) or (("dipole mode index" or "DMI" or "walker cell" or "walker circulation" or "surface sea temperature gradient") and "indian ocean") or "Delayed Oscillator" or "Recharge Oscillator" or "Western Pacific Oscillator" or "Advective-Reflective Oscillator" or "Unified Oscillator" or "La Niña*" or "La Nina*").ti,ab,hw. | 170 | Concept 1 - +ENSO and +IOD |
| 2 | ("bangladesh" or "bhutan" or "borneo" or "burma" or "cambodia" or "kampuchea" or "khmer republic" or "cook islands" or "fiji" or "guam" or "india" or "indonesia" or "kiribati" or "laos" or "marshal island?" or "malaysia" or "malay? federation" or "maldives" or "melanesia" or "micronesia" or "myanmar" or "nauru" or "nepal" or "Pleasant Island" or "northern mariana islands" or "new guinea" or "Oceania" or "palau" or "philippines" or "philipines" or "phillipines" or "phillippines" or "pilipinas" or "pacific islands" or "polynesia" or "samoa" or "samoan islands" or "navigator island" or "navigator islands" or "solomon island?" or "norfolk island?" or "santa cruz island?" or "sri lanka" or "ceylon" or "thailand" or "siam" or "timor" or "tonga" or "tahiti" or "tuvalu" or "ellice islands" or "vanuatu" or "vietnam" or "viet nam" or "west indies" or "bangladeshi?" or "bangalees" or "bajan?" or "bhutanese" or "bornean?" or "burmese" or "cambodian?" or "cook islander?" or "fijian?" or "guamanian?" or "indonesian?" or "kirabatian?" or "lao" or "laotian?" or "malaysian?" or "maldivian?" or "marshallese" or "melanesian" or "micronesian?" or "myanma" or "nepali?" or "nepalese" or "northern mariana islander?" or "mariana?" or "chamorros" or "nauruan?" or "norfolk islander?" or "oceanian" or "palauan?" or "papua new guinean?" or "philippine?" or "philipine?" or "phillipine?" or "phillippine?" or "filipino?" or "filipina?" or "pacific islander?" or "polynesian?" or "samoan?" or "solomon islander?" or "sri lankan?" or "ceylonese" or "tahitian?" or "thai" or "timorese?" or "tongan?" or "tuvaluan?" or "vanuatuan?" or "vietnamese").ti,ab,ct,hw. | 88,512 | Concept 2 – Countries/ regions |
| 3 | ("north* pacific ocean*" or "tropical pacific ocean*" or "equatorial pacific ocean*" or "south* pacific ocean*" or "mekong delta?" or "ganges delta?" or "ayeyarwady delta?" or "arabian sea" or "andaman sea" or "bay of Bengal" or "north indian ocean" or "southwestern indian ocean" or "south china sea?" or "indian subcontinent*" or ("countries" adj10 ("the world" or "worldwide" or "global*")) or ("southeastern" adj2 "asia") or ("south eastern" adj2 "asia") or "southeast asia" or "south east asia" or "south asia" or (("indian ocean" or "pacific ocean" or "indo pacific" or "indo-pacific" or "indopacific*" or "north pacific" or "tropical pacific" or "equatorial pacific" or "south pacific" or "indochina" or "indochinese") adj4 ("adjacent" or "border*" or "country" or "countries" or "region" or "regions" or "island" or "islands" or "nation" or "nations" or "economies"))).ti,ab,ct,hw. | 20,180 |  |
| 4 | 2 or 3 | 105,339 |  |
| 5 | 1 and 4 | 27 | All concepts |
| 6 | limit 5 to yr="1990 - 2024" | 27 | Date limit |

**Appendix Table 11. Embase (Ovid) Search Strategy**

| **#** | **Query** | **Results** | **Notes** |
| --- | --- | --- | --- |
| 1 | El Niño/ | 1,473 | Concept 1 - +ENSO and +IOD |
| 2 | ("El Niño*" or "El Niño*" or "El Ni˜no*" or "Oceanic Niño Index" or "Oceanic Nino Index" or "southern oscillation index" or (("sea surface temperature" or "SST*") adj3 "anomal*") or "SOI index" or "IOD?" or "+IOD" or "IOD+" or "pIOD?" or "ENSO?" or "SSTA?" or "MENSOI" or ("Indian Ocean" adj5 "Dipole") or (("nino*" or "Niño*") adj3 (pacific or "3 4" or "modoki" or "canonical" or "conventional" or "cold tongue" or "warm pool" or "dateline")) or (("dipole mode index" or "DMI" or "walker cell" or "walker circulation" or "surface sea temperature gradient") and "indian ocean") or "Delayed Oscillator" or "Recharge Oscillator" or "Western Pacific Oscillator" or "Advective-Reflective Oscillator" or "Unified Oscillator" or "La Niña*" or "La Nina*").ti,ab,ot,kf,hw,ox,dq. | 22,727 |  |
| 3 | 1 or 2 | 22,727 |  |
| 4 | pacific islands/ or cook islands/ or fiji/ or french polynesia/ or guam/ or indonesia/ or kiribati/ or marshall islands/ or melanesia/ or nauru/ or norfolk island/ or northern mariana islands/ or palau/ or papua new guinea/ or philippines/ or polynesia/ or exp samoan islands/ or solomon islands/ or timor-leste/ or tonga/ or tuvalu/ or vanuatu/ or south asia/ or bangladesh/ or bhutan/ or india/ or maldives/ or nepal/ or sri lanka/ or indian ocean/ or andaman sea/ or arabian sea/ or "bay of bengal"/ or southeast asia/ or borneo/ or cambodia/ or exp indonesia/ or laos/ or malaysia/ or myanmar/ or thailand/ or viet nam/ | 379,656 | Concept 2 – Countries/ regions |
| 5 | ("bangladesh" or "bhutan" or "borneo" or "burma" or "cambodia" or "kampuchea" or "khmer republic" or "cook islands" or "fiji" or "guam" or "india" or "indonesia" or "kiribati" or "laos" or "marshal island?" or "malaysia" or "malay? federation" or "maldives" or "melanesia" or "micronesia" or "myanmar" or "nauru" or "nepal" or "Pleasant Island" or "northern mariana islands" or "new guinea" or "Oceania" or "palau" or "philippines" or "philipines" or "phillipines" or "phillippines" or "pilipinas" or "pacific islands" or "polynesia" or "samoa" or "samoan islands" or "navigator island" or "navigator islands" or "solomon island?" or "norfolk island?" or "santa cruz island?" or "sri lanka" or "ceylon" or "thailand" or "siam" or "timor" or "tonga" or "tahiti" or "tuvalu" or "ellice islands" or "vanuatu" or "vietnam" or "viet nam" or "west indies" or "bangladeshi?" or "bangalees" or "bajan?" or "bhutanese" or "bornean?" or "burmese" or "cambodian?" or "cook islander?" or "fijian?" or "guamanian?" or "indonesian?" or "kirabatian?" or "lao" or "laotian?" or "malaysian?" or "maldivian?" or "marshallese" or "melanesian" or "micronesian?" or "myanma" or "nepali?" or "nepalese" or "northern mariana islander?" or "mariana?" or "chamorros" or "nauruan?" or "norfolk islander?" or "oceanian" or "palauan?" or "papua new guinean?" or "philippine?" or "philipine?" or "phillipine?" or "phillippine?" or "filipino?" or "filipina?" or "pacific islander?" or "polynesian?" or "samoan?" or "solomon islander?" or "sri lankan?" or "ceylonese" or "tahitian?" or "thai" or "timorese?" or "tongan?" or "tuvaluan?" or "vanuatuan?" or "vietnamese").ti,ab,ot,kf,hw,ox,dq. | 533,598 |  |
| 6 | ("north* pacific ocean*" or "tropical pacific ocean*" or "equatorial pacific ocean*" or "south* pacific ocean*" or "mekong delta?" or "ganges delta?" or "ayeyarwady delta?" or "arabian sea" or "andaman sea" or "bay of Bengal" or "north indian ocean" or "southwestern indian ocean" or "south china sea?" or "indian subcontinent*" or ("countries" and ("the world" or "worldwide" or "global*")) or ("southeastern" adj2 "asia") or ("south eastern" adj2 "asia") or "southeast asia" or "south east asia" or "south asia" or (("indian ocean" or "pacific ocean" or "indo pacific" or "indo-pacific" or "indopacific*" or "north pacific" or "tropical pacific" or "equatorial pacific" or "south pacific" or "indochina" or "indochinese") adj4 ("adjacent" or "border*" or "country" or "countries" or "region" or "regions" or "island" or "islands" or "nation" or "nations" or "economies"))).ti,ab,ot,kf,hw,ox,dq. | 230,272 |  |
| 7 | 4 or 5 or 6 | 723,223 |  |
| 8 | (("match*" adj2 ("propensity" or "coarsened" or "covariate" or "co-variate" or "neighbo?r")) or "propensity score" or "difference* in difference*" or "difference-in-difference*" or "differences-in-difference*" or "double difference*" or "quasi-experiment*" or "quasi experiment*" or ("estimat*" and "evaluat*") or "instrumental variable*" or ("IV" adj3 ("estimation" or "approach*")) or ("Heckman" adj4 ("model*" or "approach*")) or (("two-stage" or "two stage") adj4 ("control*" or "function*" or "least squares")) or "regression discontinuity" or "time series" or "counterfactual" or "segment* regression" or "coefficient of variation" or ("non" adj3 "participant*") or (("control" or "comparison") adj3 ("group*" or "condition*" or "area*" or "village*" or "household*" or "intervention")) or ("panel*" adj3 ("data" or "household*" or "model*")) or (("exploit*" or "tak* advantage") adj4 ("variation*" or "variety" or "exogen*" or "heterogen*")) or ("econometric" adj3 ("model*" or "adjust*")) or ("select*" adj3 ("bias*" or "self")) or ("experiment*" adj3 ("design" or "study" or "research" or "evaluation" or "evidence" or "vary" or "varies" or "variation")) or (("random" or "randomi?ed" or "randomly") adj3 ("trial" or "assign*" or "treatment" or "control*" or "allocat*" or "experiment*" or "vary" or "varies" or "variation" or "choose" or "chose*" or model*))).ti,ab,ot,kf,hw,ox,dq. | 3,258,256 | Concept 3 – Study designs/ Analysis |
| 9 | ((("impact?" or "effect*") adj6 ("evaluat*" or "assess" or "assessing" or "assessment*" or "analyze*" or "analyse*" or "analyzing" or "analysing" or "analysis" or "analyses" or "analytical" or "estimate*" or "estimating" or "estimation*" or "examin*" or "quantif*" or "investigat*" or "cause" or "causes" or "causal" or "causation" or "causatively" or "association?" or "associate*" or "hypothesi*" or "produce*" or "production*" or "food" or "crop" or "crops" or "disease" or "infection*" or "health" or "economic" or "price" or "prices" or "markets" or "socioeconomic" or "migration")) or ("association*" adj6 ("evaluat*" or "assess" or "assessing" or "assessment*" or "analyze*" or "analyse*" or "analyzing" or "analysing" or "analysis" or "analyses" or "analytical" or "estimate*" or "estimating" or "estimation*" or "examin*" or "quantif*" or "investigat*" or "cause" or "causal" or "causation" or "causatively" or "hypothesi*")) or "spatial correlation" or "spatial temporal" or "inciden* rate?" or "inciden* ratio?" or "rate ratio?" or (("quant*" or "effect?" or "pattern?") adj6 ("association" or "associated")) or (("composite" or "component*" or "spatial*" or "variabilit*" or "function" or "wavelet" or "correlation*" or "statistical*" or "economi*" or "macroeconomi*" or "empirical*") adj6 ("analys*" or "analyz*"))).ti,ab,ot,kf,hw,ox,dq. | 5,863,594 |  |
| 10 | 8 or 9 | 8,028,929 |  |
| 11 | 3 and 7 and 10 | 297 | All concepts |
| 12 | limit 11 to yr="1990 - 2025" | 296 | Date limit |

**Appendix Table 12. ProQuest Thesis & Dissertations Global (Web of Science) Search Strategy**

| # | Search Query | Results | Notes |
| --- | --- | --- | --- |
| 1 | TS=("El Niño*" or "El Niño*" or "El Ni˜no*" or "Oceanic Niño Index" or "Oceanic Nino Index" or "southern oscillation index" OR (("sea surface temperature" or "SST*") NEAR/3 ("anomal*" )) OR "SOI index" or "IOD$" or "+IOD" or "IOD+" or "pIOD$" or "ENSO$" or "SSTA$" or "MENSOI" OR ("Indian Ocean" NEAR/5 "Dipole") OR (("nino*" or "Niño*") NEAR/3 ("pacific" or "3.4" or "modoki" or "canonical" or "conventional" or "cold tongue" or "warm pool" or "dateline")) OR ( ("dipole mode index" or "DMI" or "walker cell" or "walker circulation" or "sea surface temperature gradient*") AND ("indian ocean") ) OR "Delayed Oscillator" or "Recharge Oscillator" or "Western Pacific Oscillator" or "Advective-Reflective Oscillator" or "Unified Oscillator" OR "La Niña*" or "La Nina*" ) | 4,333 | Concept 1 – +ENSO and +IOD |
| 2 | TS=("bangladesh" or "bhutan" or "borneo" or "burma" or "cambodia" or "kampuchea" or "khmer republic" or "cook islands" or "fiji" or "guam" or "india" or "indonesia" or "kiribati" or "laos" or "marshal island$" or "malaysia" or "malay$ federation" or "maldives" or "micronesia" or "myanmar" or "nauru" or "nepal" or "Pleasant Island" or "northern mariana islands" or "new guinea" or "Oceania" or "palau" or "philippines" or "philipines" or "phillipines" or "phillippines" or "pilipinas" or "pacific islands" or "polynesia" or "samoa" or "samoan islands" or "navigator island" or "navigator islands" or "melanesia" or "solomon island$" or "norfolk island$" or "santa cruz islands" or "sri lanka" or "ceylon" or "tahiti" or "thailand" or "siam" or "timor" or "tonga" or "tuvalu" or "ellice islands" or "vanuatu" or "vietnam" or "viet nam" or "west indies" or "bangladeshi$" or "bangalees" or "bajan$" or "bhutanese" or "bornean$" or "burmese" or "cambodian$" or "cook islander$" or "fijian$" or "guamanian$" or "indonesian$" or "kirabatian$" or "lao" or "laotian$" or "malaysian$" or "maldivian$" or "marshallese" or "melanesian" or "micronesian$" or "myanma" or "nepali$" or "nepalese" or "mariana islander$" or "mariana$" or "chamorros" or "nauruan$" or "norfolk islander$" or "oceanian" or "palauan$" or "papua new guinean$" or "philippine$" or "philipine$" or "phillipine$" or "phillippine$" or "filipino$" or "filipina$" or "pacific islander$" or "polynesian$" or "samoan$" or "solomon islander$" or "sri lankan$" or "ceylonese" or "tahitian$" or "thai" or "timorese$" or "tongan$" or "tuvaluan$" or "vanuatuan$" OR "vietnamese") OR CU=("bangladesh" or "bhutan" or "borneo" or "burma" or "cambodia" or "kampuchea" or "khmer republic" or "cook islands" or "fiji" or "guam" or "india" or "indonesia" or "kiribati" or "laos" or "marshal island$" or "malaysia" or "malay$ federation" or "maldives" or "micronesia" or "myanmar" or "nauru" or "nepal" or "Pleasant Island" or "northern mariana islands" or "new guinea" or "Oceania" or "palau" or "philippines" or "philipines" or "phillipines" or "phillippines" or "pilipinas" or "pacific islands" or "polynesia" or "samoa" or "samoan islands" or "navigator island" or "navigator islands" or "melanesia" or "solomon island$" or "norfolk island$" or "santa cruz islands" or "sri lanka" or "ceylon" or "tahiti" or "thailand" or "siam" or "timor" or "tonga" or "tuvalu" or "ellice islands" or "vanuatu" or "vietnam" or "viet nam" or "west indies" or "bangladeshi$" or "bangalees" or "bajan$" or "bhutanese" or "bornean$" or "burmese" or "cambodian$" or "cook islander$" or "fijian$" or "guamanian$" or "indonesian$" or "kirabatian$" or "lao" or "laotian$" or "malaysian$" or "maldivian$" or "marshallese" or "melanesian" or "micronesian$" or "myanma" or "nepali$" or "nepalese" or "mariana islander$" or "mariana$" or "chamorros" or "nauruan$" or "norfolk islander$" or "oceanian" or "palauan$" or "papua new guinean$" or "philippine$" or "philipine$" or "phillipine$" or "phillippine$" or "filipino$" or "filipina$" or "pacific islander$" or "polynesian$" or "samoan$" or "solomon islander$" or "sri lankan$" or "ceylonese" or "tahitian$" or "thai" or "timorese$" or "tongan$" or "tuvaluan$" or "vanuatuan$" or "vietnamese") | 139,400 | Concept 2 – Countries/ regions |
| 3 | TS=("north* pacific ocean*" or "tropical pacific ocean*" or "equatorial pacific ocean*" or "south* pacific ocean*" or "mekong delta$" or "ganges delta$" or "ayeyarwady delta$" or "bay of bengal" or "arabian sea" or "andaman sea" or "north indian ocean" or "southwestern indian ocean" or "south china sea$" or "indian subcontinent*" or ( "countries" AND ( "the world" or "worldwide" or "global*" ) ) or ("southeastern" NEAR/1 "asia") or ("south eastern" NEAR/1 "asia") or "southeast asia" or "south east asia" or "south asia") OR TI=("global") OR TS=(("indian ocean" or "pacific ocean" or "indo pacific" or "indo-pacific" or "indopacific*" or "north pacific" or "tropical pacific" or "equatorial pacific" or "south pacific" or "indochina" or "indochinese" ) NEAR/3 ("adjacent" or "border*" or "country" or "countries" or "region" or "regions" or "island" or "islands" or "nation" or "nations" or "economies")) | 66,688 |  |
| 4 | #2 OR #3 | 197,140 |  |
| 5 | TS=( ("match*" NEAR/2 ("propensity" or "coarsened" or "covariate" or "co-variate" or "neighbo$r")) or "propensity score" or "difference* in difference*" or "difference-in-difference*" or "differences-in-difference*" or "double difference*" or "quasi-experiment*" or "quasi experiment*" or ("estimat*" and "evaluat*") or "instrumental variable*" or ("IV" NEAR/2 ("estimation" or "approach*")) or ("Heckman" NEAR/3 ("model*" or "approach*")) or (("two-stage" or "two stage") NEAR/3 ("control*" or "function*" or "least squares")) or "regression discontinuity" or "time series" or "counterfactual" or "segment* regression" or "coefficient of variation" or ("non" NEAR/2 "participant*") or (("control" or "comparison") NEAR/2 ("group*" or "condition*" or "area*" or "village*" or "household*" or "intervention")) or ("panel*" NEAR/2 ("data" or "household*" or "model*")) or (("exploit*" or "tak* advantage") NEAR/3 ("variation*" or "variety" or "exogen*" or "heterogen*")) or ("econometric" NEAR/2 ("model*" or "adjust*")) or ("select*" NEAR/2 ("bias*" or "self")) OR ("experiment*" NEAR/2 ("design" or "study" or "research" or "evaluation" or "evidence" or "vary" or "varies" or "variation")) or (("random" or "randomi$ed" or "randomly") NEAR/2 ("trial" or "assign*" or "treatment" or "control*" or "allocat*" or "experiment*" or "vary" or "varies" or "variation" or "choose" or "chose*" or model*)) or (("impact$" or "effect*") NEAR/5 ("evaluat*" or "assess" or "assessing" or "assessment*" or "analyze*" or "analyse*" or "analyzing" or "analysing" or "analysis" or "analyses" or "analytical" or "estimate*" or "estimating" or "estimation*" or "examin*" or "quantif*" or "investigat*" or "cause" or "causes" or "causal" or "causation" or "causatively" or "association$" or "associate*" or "hypothesi*" or "produce*" or "production*" or "food" or "crop" or "crops" or "disease" or "infection*" or "health" or "economic" or "price" or "prices" or "markets" or "socioeconomic" or "migration")) or (("association") NEAR/5 ("evaluat*" or "assess" or "assessing" or "assessment" or "analyze*" or "analyse*" or "analyzing" or "analysing" or "analysis" or "analyses" or "analytical" or "estimate*" or "estimating" or "estimation*" or "examin*" or "quantif*" or "investigat*" or "cause" or "causes" or "causal" or "causation" or "causatively" or "hypothesi*")) or "spatial correlation" or "spatial temporal" or "inciden* rate$" or "inciden* ratio$" or "rate ratio$" or (("quant*" OR "effect$" OR "pattern$") NEAR/5 ("association" OR "associated")) OR (("composite" or "component*" or "spatial*" or "variabilit*" or "function" or "correlation*" or "wavelet" or "statistical*" or "economi*" or "macroeconomi*" or "empirical*") NEAR/5 ("analys*" or "analyz*")) ) | 1,019,083 | Concept 3 – Study designs/ Analysis |
| 6 | #1 AND #4 AND #5 | 255 | All concepts |
| 7 | #5 AND #4 AND #1 and Oceanography or Environmental Sciences Ecology or Physical Geography or Engineering or Remote Sensing or Mathematics or Geology or Area Studies or Marine Freshwater Biology or Forestry or Public Environmental Occupational Health or Chemistry or Agriculture or Computer Science or Microbiology or Pathology or Physics or Business Economics or Education Educational Research or Entomology or Health Care Sciences Services or Mechanics or Thermodynamics or Zoology or Anthropology or Archaeology or Arts Humanities Other Topics or Asian Studies or Astronomy Astrophysics or Biodiversity Conservation or Communication or Ethnic Studies or General Internal Medicine or Life Sciences Biomedicine Other Topics or Materials Science or Mathematical Computational Biology or Ophthalmology or Pharmacology Pharmacy or Physiology or Radiology Nuclear Medicine Medical Imaging or Science Technology Other Topics or Surgery or Water Resources (Research Areas) and 2023 or 2022 or 2021 or 2020 or 2019 or 2018 or 2017 or 2016 or 2015 or 2014 or 2013 or 2012 or 2011 or 2010 or 2009 or 2008 or 2007 or 2006 or 2005 or 2004 or 2003 or 2002 or 2001 or 2000 or 1999 or 1998 or 1996 or 1995 or 1994 or 1993 or 1992 (Publication Years) | 164 | 1990 – 2024 + Web of Science categories (include all except - Geochemistry Geophysics, Meteorology and atmospheric sciences, paleontology) |

**Appendix Table 13. EBSCO Discovery Service Search Strategy (limited to: GreenFILE, ScienceDirect, RePec, AGRIS, AGRIS ODS, World Bank eLibrary)**

| ID# | Search Terms | Results | Notes |
| --- | --- | --- | --- |
| S1 | TI ( ("El Niño*" or "El Niño*" or "El Ni˜no*" or "Oceanic Niño Index" or "Oceanic Nino Index" or "southern oscillation index" or (("sea surface temperature" or "SST*") N3 ("anomal*")) OR "SOI index" or "IOD#" or "+IOD" or "IOD+" or "pIOD#" or "ENSO#" or "MENSOI" OR ("Indian Ocean" N5 "Dipole") OR (("nino*" or "Niño*") N3 ("pacific" or "3.4" or "modoki" or "canonical" or "conventional" or "cold tongue" or "warm pool" or "dateline")) OR ( ("dipole mode index" or "DMI" or "walker cell" or "walker circulation" or "sea surface temperature gradient") AND ("indian ocean") ) OR "Delayed Oscillator" or "Recharge Oscillator" or "Western Pacific Oscillator" or "Advective-Reflective Oscillator" or "Unified Oscillator" OR "La Niña*" or "La Nina*") ) OR AB ( ("El Niño*" or "El Niño*" or "El Ni˜no*" or "Oceanic Niño Index" or "Oceanic Nino Index" or "southern oscillation index" or (("sea surface temperature" or "SST*") N3 ("anomal*")) OR "SOI index" or "IOD#" or "+IOD" or "IOD+" or "pIOD#" or "ENSO#" or "MENSOI" OR ("Indian Ocean" N5 "Dipole") OR (("nino*" or "Niño*") N3 ("pacific" or "3.4" or "modoki" or "canonical" or "conventional" or "cold tongue" or "warm pool" or "dateline")) OR ( ("dipole mode index" or "DMI" or "walker cell" or "walker circulation" or "sea surface temperature gradient") AND ("indian ocean") ) OR "Delayed Oscillator" or "Recharge Oscillator" or "Western Pacific Oscillator" or "Advective-Reflective Oscillator" or "Unified Oscillator" OR "La Niña*" or "La Nina*") ) OR SU ( ("El Niño*" or "El Niño*" or "El Ni˜no*" or "Oceanic Niño Index" or "Oceanic Nino Index" or "southern oscillation index" or (("sea surface temperature" or "SST*") N3 ("anomal*")) OR "SOI index" or "IOD#" or "+IOD" or "IOD+" or "pIOD#" or "ENSO#" or "MENSOI" OR ("Indian Ocean" N5 "Dipole") OR (("nino*" or "Niño*") N3 ("pacific" or "3.4" or "modoki" or "canonical" or "conventional" or "cold tongue" or "warm pool" or "dateline")) OR ( ("dipole mode index" or "DMI" or "walker cell" or "walker circulation" or "sea surface temperature gradient") AND ("indian ocean") ) OR "Delayed Oscillator" or "Recharge Oscillator" or "Western Pacific Oscillator" or "Advective-Reflective Oscillator" or "Unified Oscillator" OR "La Niña*" or "La Nina*") ) | 760,004 | Concept 1 - +ENSO and +IOD |
| S2 | TI ( ("bangladesh" or "bhutan" or "borneo" or "burma" or "cambodia" or "kampuchea" or "khmer republic" or "cook islands" or "fiji" or "guam" or "india" or "indonesia" or "kiribati" or "laos" or "marshal island#" or "malaysia" or "malay# federation" or "maldives" or "melanesia" or "micronesia" or "myanmar" or "nauru" or "nepal" or "Pleasant Island" or "northern mariana islands" or "new guinea" or "Oceania" or "palau" or "philippines" or "philipines" or "phillipines" or "phillippines" or "pilipinas" or "pacific islands" or "polynesia" or "samoa" or "samoan islands" or "navigator island" or "navigator islands" or "solomon island#" or "norfolk island#" or "santa cruz island#" or "sri lanka" or "ceylon" or "thailand" or "siam" or "timor" or "tonga" or "tahiti" or "tuvalu" or "ellice islands" or "vanuatu" or "vietnam" or "viet nam" or "west indies" or "bangladeshi#" or "bangalees" or "bajan#" or "bhutanese" or "bornean#" or "burmese" or "cambodian#" or "cook islander#" or "fijian#" or "guamanian#" or "indonesian#" or "kirabatian#" or "lao" or "laotian#" or "malaysian#" or "maldivian#" or "marshallese" or "melanesian" or "micronesian#" or "myanma" or "nepali#" or "nepalese" or "northern mariana islander#" or "mariana#" or "chamorros" or "nauruan#" or "norfolk islander#" or "oceanian" or "palauan#" or "papua new guinean#" or "philippine#" or "philipine#" or "phillipine#" or "phillippine#" or "filipino#" or "filipina#" or "pacific islander#" or "polynesian#" or "samoan#" or "solomon islander#" or "sri lankan#" or "ceylonese" or "tahitian#" or "thai" or "timorese#" or "tongan#" or "tuvaluan#" or "vanuatuan#" or "vietnamese") ) OR AB ( ("bangladesh" or "bhutan" or "borneo" or "burma" or "cambodia" or "kampuchea" or "khmer republic" or "cook islands" or "fiji" or "guam" or "india" or "indonesia" or "kiribati" or "laos" or "marshal island#" or "malaysia" or "malay# federation" or "maldives" or "melanesia" or "micronesia" or "myanmar" or "nauru" or "nepal" or "Pleasant Island" or "northern mariana islands" or "new guinea" or "Oceania" or "palau" or "philippines" or "philipines" or "phillipines" or "phillippines" or "pilipinas" or "pacific islands" or "polynesia" or "samoa" or "samoan islands" or "navigator island" or "navigator islands" or "solomon island#" or "norfolk island#" or "santa cruz island#" or "sri lanka" or "ceylon" or "thailand" or "siam" or "timor" or "tonga" or "tahiti" or "tuvalu" or "ellice islands" or "vanuatu" or "vietnam" or "viet nam" or "west indies" or "bangladeshi#" or "bangalees" or "bajan#" or "bhutanese" or "bornean#" or "burmese" or "cambodian#" or "cook islander#" or "fijian#" or "guamanian#" or "indonesian#" or "kirabatian#" or "lao" or "laotian#" or "malaysian#" or "maldivian#" or "marshallese" or "melanesian" or "micronesian#" or "myanma" or "nepali#" or "nepalese" or "northern mariana islander#" or "mariana#" or "chamorros" or "nauruan#" or "norfolk islander#" or "oceanian" or "palauan#" or "papua new guinean#" or "philippine#" or "philipine#" or "phillipine#" or "phillippine#" or "filipino#" or "filipina#" or "pacific islander#" or "polynesian#" or "samoan#" or "solomon islander#" or "sri lankan#" or "ceylonese" or "tahitian#" or "thai" or "timorese#" or "tongan#" or "tuvaluan#" or "vanuatuan#" or "vietnamese") ) OR SU ( ("bangladesh" or "bhutan" or "borneo" or "burma" or "cambodia" or "kampuchea" or "khmer republic" or "cook islands" or "fiji" or "guam" or "india" or "indonesia" or "kiribati" or "laos" or "marshal island#" or "malaysia" or "malay# federation" or "maldives" or "melanesia" or "micronesia" or "myanmar" or "nauru" or "nepal" or "Pleasant Island" or "northern mariana islands" or "new guinea" or "Oceania" or "palau" or "philippines" or "philipines" or "phillipines" or "phillippines" or "pilipinas" or "pacific islands" or "polynesia" or "samoa" or "samoan islands" or "navigator island" or "navigator islands" or "solomon island#" or "norfolk island#" or "santa cruz island#" or "sri lanka" or "ceylon" or "thailand" or "siam" or "timor" or "tonga" or "tahiti" or "tuvalu" or "ellice islands" or "vanuatu" or "vietnam" or "viet nam" or "west indies" or "bangladeshi#" or "bangalees" or "bajan#" or "bhutanese" or "bornean#" or "burmese" or "cambodian#" or "cook islander#" or "fijian#" or "guamanian#" or "indonesian#" or "kirabatian#" or "lao" or "laotian#" or "malaysian#" or "maldivian#" or "marshallese" or "melanesian" or "micronesian#" or "myanma" or "nepali#" or "nepalese" or "northern mariana islander#" or "mariana#" or "chamorros" or "nauruan#" or "norfolk islander#" or "oceanian" or "palauan#" or "papua new guinean#" or "philippine#" or "philipine#" or "phillipine#" or "phillippine#" or "filipino#" or "filipina#" or "pacific islander#" or "polynesian#" or "samoan#" or "solomon islander#" or "sri lankan#" or "ceylonese" or "tahitian#" or "thai" or "timorese#" or "tongan#" or "tuvaluan#" or "vanuatuan#" or "vietnamese") ) OR TI ( ("north* pacific ocean*" or "tropical pacific ocean*" or "equatorial pacific ocean*" or "south* pacific ocean*" or "mekong delta#" or "ganges delta#" or "ayeyarwady delta#" or "arabian sea" or "andaman sea" or "bay of Bengal" or "north indian ocean" or "southwestern indian ocean" or "south china sea#" or "indian subcontinent*" or ("countries" AND ("the world" or "worldwide" or "global*")) or ("southeastern" N1 "asia") or ("south eastern" N1 "asia") or "southeast asia" or "south east asia" or "south asia" or (("indian ocean" or "pacific ocean" or "indo pacific" or "indo-pacific" or "indopacific*" or "north pacific" or "tropical pacific" or "equatorial pacific" or "south pacific" or "indochina" or "indochinese") N3 ("adjacent" or "border*" or "country" or "countries" or "region" or "regions" or "island" or "islands" or "nation" or "nations" or "economies")) ) ) OR AB ( ("north* pacific ocean*" or "tropical pacific ocean*" or "equatorial pacific ocean*" or "south* pacific ocean*" or "mekong delta#" or "ganges delta#" or "ayeyarwady delta#" or "arabian sea" or "andaman sea" or "bay of Bengal" or "north indian ocean" or "southwestern indian ocean" or "south china sea#" or "indian subcontinent*" or ("countries" AND ("the world" or "worldwide" or "global*")) or ("southeastern" N1 "asia") or ("south eastern" N1 "asia") or "southeast asia" or "south east asia" or "south asia" or (("indian ocean" or "pacific ocean" or "indo pacific" or "indo-pacific" or "indopacific*" or "north pacific" or "tropical pacific" or "equatorial pacific" or "south pacific" or "indochina" or "indochinese") N3 ("adjacent" or "border*" or "country" or "countries" or "region" or "regions" or "island" or "islands" or "nation" or "nations" or "economies")) ) ) OR SU ( ("north* pacific ocean*" or "tropical pacific ocean*" or "equatorial pacific ocean*" or "south* pacific ocean*" or "mekong delta#" or "ganges delta#" or "ayeyarwady delta#" or "arabian sea" or "andaman sea" or "bay of Bengal" or "north indian ocean" or "southwestern indian ocean" or "south china sea#" or "indian subcontinent*" or ("countries" AND ("the world" or "worldwide" or "global*")) or ("southeastern" N1 "asia") or ("south eastern" N1 "asia") or "southeast asia" or "south east asia" or "south asia" or (("indian ocean" or "pacific ocean" or "indo pacific" or "indo-pacific" or "indopacific*" or "north pacific" or "tropical pacific" or "equatorial pacific" or "south pacific" or "indochina" or "indochinese") N3 ("adjacent" or "border*" or "country" or "countries" or "region" or "regions" or "island" or "islands" or "nation" or "nations" or "economies")) ) ) OR TI (global) | 105,085,027 | Concept 2 – Countries/ regions |
| S3 | TI ( ( ("match*" N2 ("propensity" or "coarsened" or "covariate" or "co-variate" or "neighbo#r")) or "propensity score" or "difference* in difference*" or "difference-in-difference*" or "differences-in-difference*" or "double difference*" or "quasi-experiment*" or "quasi experiment*" or ("estimat*" and "evaluat*") or "instrumental variable*" or ("IV" N2 ("estimation" or "approach*")) or ("Heckman" N3 ("model*" or "approach*")) or (("two-stage" or "two stage") N3 ("control*" or "function*" or "least squares")) or "regression discontinuity" or "time series" or "counterfactual" or "segment* regression" or "coefficient of variation" or ("non" N2 "participant*") or (("control" or "comparison") N2 ("group*" or "condition*" or "area*" or "village*" or "household*" or "intervention")) or ("panel*" N2 ("data" or "household*" or "model*")) or (("exploit*" or "tak* advantage") N3 ("variation*" or "variety" or "exogen*" or "heterogen*")) or ("econometric" N2 ("model*" or "adjust*")) or ("select*" N2 ("bias*" or "self")) or ("experiment*" N2 ("design" or "study" or "research" or "evaluation" or "evidence" or "vary" or "varies" or "variation")) or (("random" or "randomi#ed" or "randomly") N2 ("trial" or "assign*" or "treatment" or "control*" or "allocat*" or "experiment*" or "vary" or "varies" or "variation" or "choose" or "chose*" or model*)) or (("impact#" or "effect*") N5 ("evaluat*" or "assess" or "assessing" or "assessment*" or "analyze*" or "analyse*" or "analyzing" or "analysing" or "analysis" or "analyses" or "analytical" or "estimate*" or "estimating" or "estimation*" or "examin*" or "quantif*" or "investigat*" or "cause" or "causes" or "causal" or "causation" or "causatively" or "association#" or "associate*" or "hypothesi*" or "produce*" or "production*" or "food" or "crop" or "crops" or "disease" or "infection*" or "health" or "economic" or "price" or "prices" or "markets" or "socioeconomic" or "migration")) OR (("association*") N5 ("evaluat*" or "assess" or "assessing" or "assessment*" or "analyze*" or "analyse*" or "analyzing" or "analysing" or "analysis" or "analyses" or "analytical" or "estimate*" or "estimating" or "estimation*" or "examin*" or "quantif*" or "investigat*" or "cause" or "causal" or "causation" or "causatively" or "hypothesi*")) or "spatial correlation" or "spatial temporal" or "inciden* rate#" or "inciden* ratio#" or "rate ratio#" or (("quant*" OR "effect#" OR "pattern#") N5 ("association" OR "associated")) OR (("composite" or "component*" or "spatial*" or "variabilit*" or "function" or "wavelet" or "correlation*" or "statistical*" or "economi*" or "macroeconomi*" or "empirical*") N5 ("analys*" or "analyz*")) ) ) OR AB ( ( ("match*" N2 ("propensity" or "coarsened" or "covariate" or "co-variate" or "neighbo#r")) or "propensity score" or "difference* in difference*" or "difference-in-difference*" or "differences-in-difference*" or "double difference*" or "quasi-experiment*" or "quasi experiment*" or ("estimat*" and "evaluat*") or "instrumental variable*" or ("IV" N2 ("estimation" or "approach*")) or ("Heckman" N3 ("model*" or "approach*")) or (("two-stage" or "two stage") N3 ("control*" or "function*" or "least squares")) or "regression discontinuity" or "time series" or "counterfactual" or "segment* regression" or "coefficient of variation" or ("non" N2 "participant*") or (("control" or "comparison") N2 ("group*" or "condition*" or "area*" or "village*" or "household*" or "intervention")) or ("panel*" N2 ("data" or "household*" or "model*")) or (("exploit*" or "tak* advantage") N3 ("variation*" or "variety" or "exogen*" or "heterogen*")) or ("econometric" N2 ("model*" or "adjust*")) or ("select*" N2 ("bias*" or "self")) or ("experiment*" N2 ("design" or "study" or "research" or "evaluation" or "evidence" or "vary" or "varies" or "variation")) or (("random" or "randomi#ed" or "randomly") N2 ("trial" or "assign*" or "treatment" or "control*" or "allocat*" or "experiment*" or "vary" or "varies" or "variation" or "choose" or "chose*" or model*)) or (("impact#" or "effect*") N5 ("evaluat*" or "assess" or "assessing" or "assessment*" or "analyze*" or "analyse*" or "analyzing" or "analysing" or "analysis" or "analyses" or "analytical" or "estimate*" or "estimating" or "estimation*" or "examin*" or "quantif*" or "investigat*" or "cause" or "causes" or "causal" or "causation" or "causatively" or "association#" or "associate*" or "hypothesi*" or "produce*" or "production*" or "food" or "crop" or "crops" or "disease" or "infection*" or "health" or "economic" or "price" or "prices" or "markets" or "socioeconomic" or "migration")) OR (("association*") N5 ("evaluat*" or "assess" or "assessing" or "assessment*" or "analyze*" or "analyse*" or "analyzing" or "analysing" or "analysis" or "analyses" or "analytical" or "estimate*" or "estimating" or "estimation*" or "examin*" or "quantif*" or "investigat*" or "cause" or "causal" or "causation" or "causatively" or "hypothesi*")) or "spatial correlation" or "spatial temporal" or "inciden* rate#" or "inciden* ratio#" or "rate ratio#" or (("quant*" OR "effect#" OR "pattern#") N5 ("association" OR "associated")) OR (("composite" or "component*" or "spatial*" or "variabilit*" or "function" or "wavelet" or "correlation*" or "statistical*" or "economi*" or "macroeconomi*" or "empirical*") N5 ("analys*" or "analyz*")) ) ) OR SU ( ( ("match*" N2 ("propensity" or "coarsened" or "covariate" or "co-variate" or "neighbo#r")) or "propensity score" or "difference* in difference*" or "difference-in-difference*" or "differences-in-difference*" or "double difference*" or "quasi-experiment*" or "quasi experiment*" or ("estimat*" and "evaluat*") or "instrumental variable*" or ("IV" N2 ("estimation" or "approach*")) or ("Heckman" N3 ("model*" or "approach*")) or (("two-stage" or "two stage") N3 ("control*" or "function*" or "least squares")) or "regression discontinuity" or "time series" or "counterfactual" or "segment* regression" or "coefficient of variation" or ("non" N2 "participant*") or (("control" or "comparison") N2 ("group*" or "condition*" or "area*" or "village*" or "household*" or "intervention")) or ("panel*" N2 ("data" or "household*" or "model*")) or (("exploit*" or "tak* advantage") N3 ("variation*" or "variety" or "exogen*" or "heterogen*")) or ("econometric" N2 ("model*" or "adjust*")) or ("select*" N2 ("bias*" or "self")) or ("experiment*" N2 ("design" or "study" or "research" or "evaluation" or "evidence" or "vary" or "varies" or "variation")) or (("random" or "randomi#ed" or "randomly") N2 ("trial" or "assign*" or "treatment" or "control*" or "allocat*" or "experiment*" or "vary" or "varies" or "variation" or "choose" or "chose*" or model*)) or (("impact#" or "effect*") N5 ("evaluat*" or "assess" or "assessing" or "assessment*" or "analyze*" or "analyse*" or "analyzing" or "analysing" or "analysis" or "analyses" or "analytical" or "estimate*" or "estimating" or "estimation*" or "examin*" or "quantif*" or "investigat*" or "cause" or "causes" or "causal" or "causation" or "causatively" or "association#" or "associate*" or "hypothesi*" or "produce*" or "production*" or "food" or "crop" or "crops" or "disease" or "infection*" or "health" or "economic" or "price" or "prices" or "markets" or "socioeconomic" or "migration")) OR (("association*") N5 ("evaluat*" or "assess" or "assessing" or "assessment*" or "analyze*" or "analyse*" or "analyzing" or "analysing" or "analysis" or "analyses" or "analytical" or "estimate*" or "estimating" or "estimation*" or "examin*" or "quantif*" or "investigat*" or "cause" or "causal" or "causation" or "causatively" or "hypothesi*")) or "spatial correlation" or "spatial temporal" or "inciden* rate#" or "inciden* ratio#" or "rate ratio#" or (("quant*" OR "effect#" OR "pattern#") N5 ("association" OR "associated")) OR (("composite" or "component*" or "spatial*" or "variabilit*" or "function" or "wavelet" or "correlation*" or "statistical*" or "economi*" or "macroeconomi*" or "empirical*") N5 ("analys*" or "analyz*")) ) ) | 32,078,839 | Concept 3 – Study designs/ Analysis |
| S4 | S1 AND S2 AND S3 | 14,932 | All concepts |
| S5 | S1 AND S2 AND S3  Limit to collections (GreenFILE, ScienceDirect, RePec, AGRIS, AGRIS ODS, World Bank eLibrary) | 852 | Limited to specific sources |

## Appendix B Characteristics of included papers

**Appendix Table 14. Characteristics of included studies with a summary of findings for economic outcomes, categorised by country**

*Order: Multi-country studies first. Then alphabetic based on the country. For each country, alphabetic based on lead author’s name.*

| Study | Country | ENSO/IOD metric | Outcome | Method (only those relevant for our review) | Risk of bias | Findings as presented by authors (unless findings are different based on our inclusion criteria) |
| --- | --- | --- | --- | --- | --- | --- |
| Abdolrahimi (2016) | Bangladesh, India, Nepal, Sri Lanka, Indonesia, Malaysia, Philippines, Vietnam, Cambodia, Thailand, Myanmar, Laos and out-of-scope countries | Niño 3.4 SST anomaly index | Production | Threshold regression model | High | El Niño shocks are likely to cause on average a reduction in global production of rice and maize. La Niña episodes, on the other hand, are associated with increased global rice and decreased global wheat and maize production. Results are also reported separately by country. For Indo-Pacific countries the effect sizes are not significant except for Bangladesh (rice), Indonesia (rice) and Philippines (maize). |
| Bertrand et al. (2023) | Bangladesh, India, Indonesia, Vietnam, Philippines, Myanmar, Cambodia and out-of-scope countries | ONI: Oceanic Nino Index | Production | Analysis of Variance comparing mean anomalies estimates using locally weighted scatterplot smoothing | High | Results show that there is no obvious large-scale differences in production between different ENSO categories or El Niño event types. However, certain ENSO conditions (La Niña and Extreme El Niño) are more often associated with larger variations in production anomalies, indicating that they have more scope to produce shocks in the aquaculture sector. ENSO event category or El Niño event type have no statistical effect on inland fisheries production in any of the subregions, but percentage anomalies associated with the different ENSO conditions and El Niño event types vary considerably (between −18.8 percent and +34.9 percent). La Niña is associated with more extreme production anomalies than neutral or El Niño events. Extreme El Niño saw particularly large numbers of inland fisheries subregions with negative production anomalies. Considerable (−14.4 percent to +8.3 percent) production anomalies at the country level are associated with the different El Niño event types. |
| Ismail and Chan (2020) | Cambodia, Indonesia, Philippines, Malaysia, Myanmar, Thailand and Vietnam | MEI (including El Niño intensity) / ONI / Niño 3.4 SST anomaly index / SOI | Production | Pooled Mean Group estimation method | Low | The findings reveal that the ENSO effect is exclusively captured by Multivariate ENSO Index (MEI) among four climate change variables. Furthermore, ENSO (EL Niño) arrival during the paddy planting season caused a paddy reduction in the long run. |
| Bekkering (2017) | India, Indonesia, Malaysia, Philippines and Thailand and out-of-scope countries | SOI: southern oscillation index | Investments | Simple univariate regression | Unclear | Strong El Niño events influence the stock returns of eight markets. First, a decline in returns is visible in most markets, after which an increase is observed [Indonesia is the only country where the overall effect is significantly negative]. Trading volume tends to decrease as a consequence of strong El Niño shocks. The strongest effects are visible on the Indian and the Filipino market. |
| Ghose et al. (2021) | Bangladesh | SOI: southern oscillation index / Niño 3.4 / 4 SST anomaly index / Multivariate ENSO Index / DMI: dipole mode index | Productivity | Linear regression | High | Among the four sub-zones, south-central and northern zones have the most notable associations between the climate-induced yield index and key climatic variables/large-scale atmospheric circulation index, while the potential evapotranspiration (PET) in March and multivariate ENSO indices (MEI) in January are identified as the best yield prediction indicator. Wavelet coherence analysis indicates significant in-phase and out-phase coherences between climate-induced yield index and key climatic variable fluctuations at different time-frequency bands in these sub-zones. Regression analysis does not report a significant relationship with productivity for the studied climate drivers. |
| Amat and Ashok (2018) | India | Niño 3 / 3.4 SST anomaly index / Multivariate ENSO Index / IODMI: Indian Ocean Dipole Mode Index | Production | Partial correlation analysis | High | El Niño was associated with lower production in most states, with the exemption of Kerala and West Bengal. +IOD was associated mostly with increased Kharif crop production with the exception of Kerala and Karnataka. |
| Bhatla et al. (2020) | India | Niño 3.4 SST anomaly index | Production | Correlation analysis | High | During El Niño years, the production of rice and sugarcane is affected in the middle and upper regions of Indian Gangetic Plains (IGP). The production of wheat decreases during La Nina events in the middle regions of IGP. The rice production is severely affected by El Niño events over middle and upper IGP regions whereas wheat production increases. The decrease in maize production is observed in the upper and trans regions of IGP during El Niño. Pulse production decreases mainly in the middle, upper, and trans IGP during both events. The sugarcane production is highly affected during La Nina events overall sub-regions of IGP. |
| Bhatla et al. (2023) | India | Niño 3.4 SST anomaly index / ONI: Oceanic Nino Index / DMI: dipole mode index | Production | Correlation analysis | High | The results show that rice productions which require hot and humid conditions have been largely affected during drought years associated with El Niño which results in poor rainfall over all the zones. The production of pulses which does not require excess humid conditions shows marginal improvement during the neutral years or non-El Niño/non-La Nina years. Maize production seems to be better in La Nina years and worst in El Niño years. El Niño years provide a minor impact on sugarcane productions in different zones. Positive IOD years are associated with poor crop productions as compared to negative IOD years mostly in all zones as most of the positive IOD years happen to be El Niño years. El Niño-rainfall relation being dominant than positive IOD-rainfall relation is, therefore, responsible for negative rainfall anomalies over the selected zones. |
| Cashin et al. (2015) | India | SOI: southern oscillation index | Aggregated production / Prices | Global Vector Auto Regressive modelling | Low | The results show that there are considerable heterogeneities in the responses of different countries to El Niño shocks. While India, faces a short-lived fall in economic activity in response to an El Niño shock, for other countries (including the United States and European region), an El Niño occurrence has a growth-enhancing effect. Furthermore, most countries in our sample experience short-run inflationary pressures as both energy and non-fuel commodity prices increase. Given these findings, macroeconomic policy formulation should take into consideration the likelihood and effects of El Niño weather episodes. Results are also reported separately by country. For Indo-Pacific countries the effects are not significant except for Indonesia and Thailand: a negative relationship between El Niño and GDP for the former, and positive for the latter. |
| Ferris (1999) | India | Niño 3.4 SST anomaly index | Productivity | Autoregressive Moving Average time series linear regression | High | India’s wheat yields are significantly and positively affected by lagged El Niño and La Niña and negatively affected by the current La Niña. The most significant impact on coarse grain yields is a positive effect of lagged El Niño. |
| Garnett and Khandekar (1992) | India and out-of-scope countries | Niño 3 SST anomaly index / SOI: southern oscillation index | Productivity | Linear correlation with detrended series | High | It is found that an ENSO event (El Niño) is generally associated with a drought in the Indian monsoon followed by low grain yield over south Asia and Australia and high grain yields over the North American prairies. For India, while effects on productivity are not statistically significant, they may vary by season. |
| Giuseppe et al. (2022) | India and out-of-scope countries | ONI: Oceanic Nino Index | Productivity | Robust Analysis of Variance one-way regression | High | Percentage effects of El Niño on winter yields indicate a 4.74% decrease of yield with respect to the neutral phase. |
| Krishna et al. (2004) | India | Niño 3 SST anomaly index / Niño 4 SST anomaly index / Darwin, Australia, sea-level pressure (SLP) anomaly | Production | Correlation analysis | High | The influence of NINO3 SST anomalies on kharif food-grain production is strongest in the western and central peninsula (Gujarat, Rajasthan, Uttar Pradesh, Punjab, Andhra Pradesh), and to a lesser extent in Karnataka and Tamil Nadu. When examining the correlations for the recent two decades between different crop indices and NINO3, authors found that the magnitude of correlations shows some reduction but is still statistically significant, unlike the recent weakening of correlation between all-India monsoon rainfall and NINO3. |
| Laosuthi and Selover (2007) | India, Indonesia, Malaysia, Philippines and Thailand | SOI: southern oscillation index | Aggregated production / Prices | Correlational analysis | High | El Niño has relatively little detectable effect on the business cycles of most of the countries that we study. El Niño appears to significantly affect mainly South Africa, Australia, India, and perhaps Malaysia. |
| Nageswararao et al. (2018) | India | SOI: southern oscillation index | Productivity | Partial correlation analysis | High | The productivity of different Rabi crops in most of the places of NWI is most likely influenced by variability in local temperatures. Moreover, Nino3.4 region SST (SOI) positively (negatively) affects the productivity of gram, rapeseed–mustard, and total Rabi oilseeds in most of the states. |
| Panda et al. (2019) | India | ONI: Oceanic Nino Index / Niño 3 SST anomaly index / 3.4 SST anomaly index | Productivity | Correlation analysis | High | The amount of monsoon rainfall is found to have a significant impact on crop productivity, compared to temperature, in the study area, and as a result the Monsoon Index has a determining impact on crop yield among various indices. Climate indices like ONI and NINO 3 are shown to have a moderate impact on the crop yield of the study area; this may be due to the time lag between sowing season (May–June) of the crops and peak occurrence (November–December) of the climatic conditions in Paciﬁc Ocean. |
| Raj et al. (2020) | India | Niño 1.2 / 3 / 3.4 / 4 SST anomaly index index | Productivity | Correlational analysis | High | Authors report a positive correlation between Nino anomalies and tea productivity. The effects vary by region (with negative estimates for Vandiperiyar). |
| Rao et al. (2012) | India | MEI: multivariate ENSO index | Productivity | Linear regression and correlation analysis | High | The regression equation that was developed for the All-India rice yield maintained a high, statistically significant correlation. The regression portrays the significance of not only the regional climate variables but also the index of atmospheric climate drivers (MEI) on rice yield by modulating the regional weather systems. |
| Sahu et al. (2020) | India | ONI / SOI / El Niño Modoki index / TNI: Trans Niño Index / Niño 3 SST anomaly index / DMI: dipole mode index | Productivity | Pearson’s product moment correlation | High | Pearson’s Product Moment Correlation provides an overview of the signiﬁcant correlation between climate indices and rice productivity. Niño 3, Ocean Niño Index (ONI) and Southern Oscillation Index are found highly associated with years having severer than −10% decline in rice productivity. Findings from the correlational analysis used for this systematic review indicate that the direction and magnitude of effects on rice productivity in Bihar varied with the choice of El Niño metric but possibly increased during +IOD in April-May-June. |
| Selvaraju (2003) | India | Niño 1.2 / 3 / 3.4 / 4 SST anomaly index | Production | Correlational analysis | High | The authors found that El Niño is negatively associated with foodgrain production, and that the effects are largest on rice crops. |
| Zacharia et al. (2020) | India | SOI: southern oscillation index / Indian Ocean Dipole (IOD) [unclear which index] | Productivity | Path analysis (Shipley, 2016) followed by multiple regression analysis | High | El Niño is found to be negatively associated with catch per unit effort. A weak negative correlation is also reported for +IOD but it is not clear whether the association is statistically significant (The text suggests there is an association, but the results table does not confirm it). |
| Ahmad et al. (2019) | Indonesia | Niño 3.4 SST anomaly index / Temperature anomaly of the province in Indonesia | Prices | Spatial lag model or spatial autoregressive | High | The anomaly of provincial temperature has a positive effect on inflation in the southern Indonesian provinces that are affected by El Niño, but no effect in northern Indonesia. No effects detected for Niño 3.4 SST anomaly index. |
| Cahyaningtyas et al. (2022) | Indonesia | Niño 3.4 SST anomaly index | Trade | Vector Error Correction Model regression | Unclear | The Vector Error Correction Model demonstrated the negative effect of ENSO (El Niño) on Indonesia’s natural rubber export. |
| Fajri et al. (2019) | Indonesia | El Niño years (dummy variable) | Prices | Fixed effects model | Unclear | The result shows that El Niño has a small inflationary yet statistically significant association with rice and soy bean prices yet a deflationary association with maize price. |
| Falcon et al. (2004) | Indonesia | August SSTA fluctuations | Production / Productivity | Time series regression using time and time-squared terms and level variables instead of first difference | High | Each degree Celsius change in the August SSTA is associated with a 1,318,000 metric ton reduction in output. Of the inter-annual production changes due to SSTA variation, 90% occur within 12 provinces, notably Java and South Sulawesi. |
| Ismaya and Anugrah (2018) | Indonesia | ONI: Oceanic Nino Index | Prices | Multivariate regression and Generalized Method of Moments | High | Authors find that backward-looking and forward-looking expectations, rice production, credit to the agricultural sector, climate (La Niña-El Niño), rice imports, demand level (M1/consumption), rice field productivity, infrastructure (irrigation), and seasonal events (Ramadhan-Eid Mubarak) have a statistically significant correlation with rice price level. El Niño is associated with higher prices. |
| Khoiruddin (2021) | Indonesia | ONI: Oceanic Nino Index | Prices | Vector Error Correction Model | Low | The results showed that the price volatility of CPO in Indonesia is low and will be persistent in the long term. ENSO affects the volatility of CPO prices in the long run, but there is no effect in the short run. |
| Kusuma (2019) | Indonesia | Niño 3.4 SST anomaly index / DMI: dipole mode index | Productivity | Geographically weighted regression | High | Paddy yields are correlated positively with the Indian El Niño in most of Kalimantan and all parts of Sumatra, Java-Bali and Sulawesi while there is no evidence that climate impact exists at districts further away in northern Kalimantan, all Maluku and all of Papua. For the Pacific El Niño (ENSO), the phenomenon’s impact on paddy yield is found in the southern part of Sumatra and in only a small part of northern Java while a moderate effect is experienced in the northern part of Sumatra and much of Java. Negative effects found on average and for the bottom quartile and median of the El Niño 3.4 Anomaly index. |
| Nugroho (2013) | Indonesia | SOI: southern oscillation index | Productivity | Linear regression | High | For some districts, the first principal component of the SSTs was negatively correlated to crop yield residuals of com and soybean while SOl was positively correlated to that of com and dryland paddy. Using these relationships, linear regressions were successfully constructed to predict crop yields. El Niño phase is negatively correlated with paddy yields in Panggang/Patuk and corn yields in Wonosari; not significant for other districts and crops. |
| Nugroho (2015) | Indonesia | SOI: southern oscillation index | Productivity | Correlation analysis | High | Soybean and dryland paddy yields were highly correlated with the amount of rainfall in January. The average SOI during June-September was highly positively correlated with maize yield during January-April which points to a negative relationship with EL Niño. |
| Nugroho and Nuraini (2016) | Indonesia | SOI: southern oscillation index | Productivity | Linear correlation analyses | High | The result shows in macro scale, mostly sub-district has significant correlations between global climate indices and crop yields.  The correlation between SOI and crop yields shows mostly sub districts in Banyumas have a positive correlation between El Niño and crop yields. However, the relationship is only statistically significant for paddy in Kalibagor, Patikraja and paddy/corn in Sumbang. |
| Siregar et al. (2020) | Indonesia | Niño 3.4 SST anomaly index | Aggregated production | Pooled least squares | High | The findings indicate that El Niño negatively affects the small and medium industries production index |
| Suratno (2022) | Indonesia | DMI: dipole mode index | Production | Analysis of Variance | High | The findings indicate no statistically significant relationship between IOD and fish catches |
| Utami et al. (2011) | Indonesia | SOI: southern oscillation index | Production | Ordinary least squares regression (log model) | Unclear | The findings indicate that El Niño events had no statistically significant relationship with rice paddy supply yet a positive relationship with maize production. |
| Khor et al.(2021) | Malaysia | ONI: Oceanic Nino Index | Productivity | Mann Kendall's test and Sen's slope test | High | With the Fresh Fruit Bunch Index model, Malaysian oil palm yields are better correlated with ONI and have higher predictive ability. In general, oil palm yields started to decrease after zero to six months of an El Niño event. |
| Mahmudul et al. (2020) | Malaysia | El Niño years (dummy variable) | Investments | Generalized Method of Moments | Low | Both El Niño and flood have significant negative impact on the firms’ financial performance as measured by ROA and ROE. |
| Rahman et al. (2013) | Malaysia | El Niño years (dummy variable) | Prices / Production | Autoregressive integrated moving average, Multiple regression analysis | Low | Both La Niña and El Niño events are associated with increases in crude palm oil prices and decreases in palm oil production. |
| Tawang et al. (2002) | Malaysia | El Niño years (dummy variable) | Productivity | Linear regression for estimating yield functions | High | The analyses carried out provided strong evidence of the negative effects of El Niño on Malaysian agriculture. Total losses to agriculture as a result of poorer performance of oil palm, rubber and rice due to El Niño were estimated to be more than RM 3.3 billion for the 1980 to 1999 period. Authors report negative effects for productivity of palm and rubber yet positive for rice. |
| Tawang et al. (2003) | Malaysia | El Niño years (dummy variable) | Productivity | Linear regression for estimating yield functions | High | In general, this study concludes that the only crop or commodity that was negatively affected by El Niño phenomena in the northwest region of Peninsular Malaysia was paddy. A positive effect was observed for tobacco yields. |
| Wen and Sidik (2011) | Malaysia | SOI: southern oscillation index | Production | Correlation analysis | High | Fish landings along the West Coast of Sabah are enhanced during El Niño periods while production of palm oil FFB is favoured during La Niña periods although floods are not desirable. |
| Yang (2017) | Malaysia | Niño 3 SST anomaly index | Productivity | Ordinary least squares regression | Low | El Niño anomaly conditional on winter season can predict stock returns in Malaysia and in other 12 considered countries. The effects of El Niño unconditional of winter are negative, whereas the effects of El Niño conditional to winter seasons are positive. |
| Duncan (2008) | Papua New Guinea | El Niño years (dummy variable) | Trade | Nerlove supply equations | Unclear | Findings show that there is little supporting evidence for a link between agricultural output in export cash crops and PNG elections. What does appear to be true is that recent ENSO events in Papua New Guinea have occurred around election years. It is argued that these ENSO events and the droughts and floods that follow that have impacted on Papua New Guinea's agricultural productivity. El Niño is associated with decreased cocoa, copra, palm oil export but with higher trade volumes of copra oil and coffee. |
| Arcenas (2018) | Philippines | El Niño years (dummy variable) | Prices | Random effects | High | Both weather shocks have significant inflationary effects on the general price level in the Philippines, along with interest rate, foreign exchange, and unemployment rate. |
| Cruz and Canlas (2004) | Philippines | SOI: southern oscillation index / SST Anomalies | Production | Multiple regression with Hodrick-Prescott Filter | Unclear | Results from the statistical extraction show that cyclical fluctuations do manifest in the agricultural crop production in the Philippines. The turning points of the resulting cycle coincide with the ENSO years. The time series also shows marked deviations from the trend, which coincide with the turning points of the cycle as well as with the ENSO years. Effects vary by crop type. While rice, corn and sugarcane output may increase, coconut production does not systematically change with El Niño events. |
| Dait (2022) | Philippines | El Niño years (dummy variable) | Aggregated production | Autoregressive time series regression analysis | High | Results show that only three variables indicated considerable significance on agricultural production in the Philippines based on their respective t-ratios: Agricultural Employment, temperature, and La Nina. The incidence of El Niño is associated with decreased agricultural output. The relationship is not statistically significant. |
| Datt and Hoogeveen (2003) | Philippines | El Niño Shock in 1998 (self-reported being affected by drought or El Niño in the last six months) | Consumption and expenditures | Instrumental variable estimation | Unclear | The largest share of the overall impact on poverty appears attributable to El Niño shock as opposed to shocks mediated through the labor market. Both household and community characteristics mattered to the differential impact of the crisis. There is some evidence of consumption smoothing by the crisis-affected households, though the poor amongst them were more constrained in their ability to protect their consumption. |
| David (2023) | Philippines | SOI: southern oscillation index | Productivity | Correlation analysis | High | There were weak negative correlations between rice crop yields and variables like wind speed, pressure at station, pressure at -sea, SOI, and Tropical Depressions. These correlations suggest potential associations between these factors and rice crop yield, albeit with weak linear relationships. |
| Reyes et al. (2009) | Philippines | El Niño years (dummy variable) | Production | Multiple linear regressions (robust) | Low | This study demonstrated an approach that systematically linked seasonal climate forecast to rice importation decisions. In particular, it showed that SCF can be incorporated in rice production models to predict the total supply of rice. Multiple linear regressions showed that the association between El Niño and rice production varies greatly by region. |
| Roberts et al. (2009) | Philippines | Niño 3.4 SST anomaly index | Production / Productivity | Ordinary least squares regression | High | Both irrigated and rainfed ecosystems are impacted by El Niño. Production declines for rainfed ecosystems are relatively larger than for irrigated ecosystems: a 18C increase in average July–September Niño-3.4 SSTA is associated with a 3.7% decrease in irrigated dry-season production but with a 13.7% decline in rainfed dry-season production. |
| Soria and Preciados (2018) | Philippines | ONI: Oceanic Nino Index | Production | Ordinary least squares regression | Unclear | El Niño is associated with decreased cassava production although the estimates are not statistically significant. |
| Stuecker et al. (2018) | Philippines | Niño 3.4 SST anomaly index | Production / Productivity | Correlation analysis | High | Authors report that rice production is negatively correlated with Nino events. This is due to the detrimental impact of Nino on soil moisture, which in turn undermines crop production. While the correlation between El Niño and production does not differ by region, the negative effects on productivity are larger on rainfed rice fields as irrigated paddy rice is less reliant on rainfalls although it is ultimately dependent on water availability. |
| Zubair (2002) | Sri Lanka | Niño 3.4 SST anomaly index | Productivity | Correlational analysis | High | El Niño conditions are likely correlated with an increase in the average Maha season rice production and a decline in that of Yala season. |
| Limsakul (2019) | Thailand | MEI: multivariate ENSO index | Production / Productivity | Spearman’s rank order correlation for MEI positive values and multiple least-squares linear regression | High | Analysis showed that year-to-year weather-related variations in Thailand’s rice production, area harvested and yield which accounted for about one third of total interannual variance tended to vary in response to the phase reversals of ENSO events, with large decreases occurred during El Niño events. |
| Pipitpukdee et al. (2020a) | Thailand | El Niño years (dummy variable) | Production / Productivity | Instrumental variable and Spatial Regression with Price and Wage Variables | High | General climate variables, both mean and variability, statistically determined the yield and harvested area of sugarcane [for La Niña but not for EL Niño]. Increased population density reduced the harvested area for non-agricultural use. |
| Pipitpukdee et al. (2020b) | Thailand | El Niño years (dummy variable) | Production / Productivity | Instrumental variable with generalized method of moments | High | The findings indicate negative effects of El Niño on cassava yields and harvested area (though not statistically significant for the latter). Furthermore, La Nina years are positively correlated with both harvested area and yields. |
| Hughes et al. (2010) | Samoa | SOI: southern oscillation index / TNI: Trans Niño Index / ONI: Oceanic Nino Index / MEI: multivariate ENSO index | Productivity/ Production / Trade | Autoregressive Moving Average time series analysis | High | A macroeconomic model of the interactions between climate and the economy suggests that the present value of the damage to the Samoan economy through 2050 due to climate change— and without additional adaptation—may be $104–$212 million; this is equivalent to 0.6–1.3 percent of the present value of GDP over the same period. The findings indicate a positive relationship of El Niño with agricultural import but negative for Taro production and coconut yields. |

**Appendix Table 15. Characteristics of included studies with a summary of findings for health outcomes, categorised by country**

*Order: Multi-country studies first. Then alphabetic based on the country. For each country, alphabetic based on lead author’s name.*

| Short Title | Country | ENSO/IOD metric | Outcome | Method (only those relevant for our review) | Risk of bias | Findings as presented by authors (unless findings are different based on our inclusion criteria) |
| --- | --- | --- | --- | --- | --- | --- |
| Azad and Lio (2014) | Bangladesh, Bhutan, India, Indonesia, Maldives, Nepal, Sri Lanka, Myanmar, Sri Lanka, Thailand, Timor Leste and out-of-scope countries | SST Anomalies | Vector-borne diseases | Pearson’s correlation coefficient | High | There is not much evidence of a periodic association between malaria and ENSO in the region; however, dengue fever and ENSO show significant cross-coherence in the two–four year wavelet band and the results are statistically significant in the last decade. |
| Lam et al. (2019) | India, Philippines and out-of-scope countries | ONI: Oceanic Nino Index | Direct injuries and fatalities | Poisson Generalized Additive Models | High | Flood events were the disaster type most strongly associated with El Niño regionally: in South Asia, flood-related people affected by disasters (PAD) increased with each boundary point increase in ONI. India was found to be the country with the largest increase in flood-related PAD rates following an El Niño event, with the Philippines experiencing the largest increase following a La Niña event. |
| Llewellyn (2010) | Fiji, Samoa, Vanuatu, Kiribati and out-of-scope countries | SOI: southern oscillation index / Niño 3.4 / 4 SST anomaly index / TNI / MEI / Bivariate ENSO | Enteric infections and diseases (other than cholera) | Correlation analysis | High | Statistically significant, positive and negative cross-correlations were obtained between time series of annual ciguatera case rates and the Pacific Warm Pool Index and several ENSO related indices which had been lagged for up to two years before the ciguatera time series. |
| Gagnon et al. (2001) | Indonesia and out-of-scope countries | El Niño years (dummy variable) | Vector-borne diseases | Correlation using contingency tables with Fisher’s exact test | Unclear | There is a statistically significant correlation between El Niño and dengue epidemics in Indonesia and countries from northern South America which experience statistically significant warmer temperatures and less rainfall during El Niño years. The evidence suggests that droughts lead to dengue haemorrhagic fever epidemics in Indonesia at the onset of the following rainy season. |
| Banu et al. (2015) | Bangladesh | Niño 3.4 SST anomaly index / DMI: dipole mode index | Vector-borne diseases | Partial correlation analysis and Poisson time series model combined with the distributed lag nonlinear model | Low | A distributed lag nonlinear model revealed that the association between dengue incidence and ENSO or IOD were comparatively stronger after adjustment for local climate variables, seasonality and trend. The estimated effects were nonlinear for both ENSO and IOD with higher relative risks at higher ENSO and IOD. The weak association between ENSO, IOD and dengue incidence might be driven by the stronger effects of local climate variables such as temperature and rainfall. |
| Cash et al. (2014) | Bangladesh | Niño 3.4 SST anomaly index | Cholera | Spearman correlation | High | Similar climate associations are found for Cholera and Shigellosis in Dhaka and Matlab. Namely, increased cases follow increased monsoon flooding and increased sea surface temperatures in the preceding winter corresponding to an El Niño event. |
| Daisy et al. (2020) | Bangladesh | Niño 3.4 SST anomaly index / SOI: southern oscillation index | Cholera | Cross-correlation analysis (Pearson’s correlation coefficient) | High | A low relationship was found between cholera incidence and relative humidity, ENSO and SOI. For Dhaka, the correlation of cholera with temperature and rainfall individually showed better results than other climatic variables, i.e. relative humidity, ENSO and SOI. |
| Hashizume et al. (2011) | Bangladesh | Niño 3 SST anomaly index / DMI: dipole mode index | Cholera | Negative binomial generalized linear models | Low | The findings suggest that hospital visits for cholera in both Dhaka and Matlab increased in association with a positive IOD (positive DMI) during the previous zero–three months and with an increase in the ENSO (high Niño 3 SST anomaly index) during the previous 8–11 months. |
| Perez-Saez et al. (2016) | Bangladesh | Niño 3.4 SST anomaly index | Cholera | Autoregressive Integrated Moving Average time series analysis | High | Findings suggest a higher sensitivity to ENSO in the highly populated urban center than in the more rural periphery. More significantly, the results show that cholera risk is largely transmitted from the climate-sensitive core to the periphery of the city, with implications for the planning of control efforts. |
| Rodó et al. (2002) | Bangladesh | SOI: southern oscillation index | Cholera | Correlational analysis | High | Findings indicate strong effects of ENSO over the recent events (1980 –2001), while the effects are weaker and eventually uncorrelated during the first parts of the last century. |
| Sharmin et al. (2016) | Bangladesh | Niño 3.4 SST anomaly index | Vector-borne diseases | Negative binomial Bayesian generalised linear model | Low | The model estimated that only a small portion of all cases in the capital Dhaka were reported through passive case reporting. The optimal mean monthly temperature for dengue transmission is 29° C and average monthly rainfall above 15 mm decreases transmission. A bimodal relationship was observed between SSTA and dengue incidence, reflecting the influence of both El Niño and La Niña events on monsoon rainfall in Bangladesh. However, the influence was not significant in the current month and at a lag of one month. |
| Iyer et al. (2021) | India | ONI: Oceanic Nino Index | Enteric infections and diseases (other than cholera) | Negative binomial generalized estimating equations | Unclear | Phases of ENSO had opposite effects on enteric fever across the cities of Ahmedabad and Surat. In Ahmedabad, strong El Niño months were associated with an increase in enteric fever risk while strong La Niña months with a reduction in risk. In Surat, strong El Niño was associated with a reduction in risk while moderate La Niña with an increase in risk. |
| Kakarla et al. (2019) | India | Niño 3.4 SST anomaly index / DMI: dipole mode index | Vector-borne diseases | Pearson's correlation | High | A weak synchronous correlation was observed between Nino3.4, DMI and dengue cases from 2010 to 2017. However, the largest El Niño events and the largest positive phases of the IOD coincide with the largest number of dengue cases reported in India in 2015 and 2016. El Niño and +IOD were associated with increased dengue cases (though the relationship was not statistically significant). |
| Oluwole (2015) | India | Paleoclimate ENSO multiproxy / MEI: multivariate ENSO index | Vector-borne diseases | Contingency table and odds ratio calculation | High | Findings show a strong association of lathyrism Epidemics in India and El Niño phase of the ENSO from 1833 to 1902. Visual inspection of historical lathyrism epidemics and ENSO shows that lathyrism epidemics occur following El Niño. |
| Pramanik et al. (2020) | India | ONI: Oceanic Nino Index | Vector-borne diseases | Pearson's correlation test | High | A high correlation between positive ONI and dengue incidence was found in India. The generated map showing the spatial correlation between El Niño and dengue suggests a positive correlation in the central part, while negative correlation in some coastal, northern, and north-eastern part of India. |
| Arcari and Tapper (2017) | Indonesia | SOI: southern oscillation index | Vector-borne diseases | Multiple regression analyses | High | During El Niño events, the amount of variance in dengue/DHF incidence explained by climate variables such as rainfall and temperature increases. A negative association with SOI is observed for both ENSO events during the research period (1992-5 and 1997-8). |
| Dhewantara et al. (2021) | Indonesia | East Central Tropical Pacific SST (Nino 3.4) / DMI: dipole mode index | Vector-borne diseases | Correlation analysis | High | The annual trend of dengue incidence rate seems to be influenced by DMI but there is no clear evidence that DMI is the main reason for dengue reduction in 2017. |
| Harapan et al. (2020) | Indonesia | Niño 3.4 SST anomaly index / DMI: dipole mode index | Vector-borne diseases | Spearman's rank correlation | High | There was no significant correlation between monthly El Niño and chikungunya incidence. However, there was a significant negative correlation between monthly DMI and chikungunya incidence, suggesting that DMI could be a potential driver of chikunguya. |
| Prasetyowati et al. (2021) | Indonesia | Niño 3.4 SST anomaly index / DMI: dipole mode index | Vector-borne diseases | Spearman correlation | High | The findings indicate not significant correlation between El Niño events and Dengue Fever incidence in Jakarta but a significant association between the later and +IOD, which along with significant correlations with precipitation, humidity and temperature, confirms that Dengue Fever in Jakarta is driven by climatic components. |
| Che-Him (2018) | Malaysia | Niño 4 SST anomaly index | Vector-borne diseases | Negative binomial generalized additive model l | Low | Climate information alone does not account for a large proportion of the overall variation in dengue incidence rate of Malaysia, however, spatio-temporal climate information does significantly account for some of this variability. For ENSO, dengue predictive models need to consider up to six months of lagged values. Still, the results were not significantly different from zero. |
| Impoinvil et al. (2013) | Malaysia | SOI: southern oscillation index | Vector-borne diseases | Poisson log-linear regression model | Low | There was an estimated reduction in Japanese Encephalitis (JE) risk after the introduction of vaccination which is over-estimated when the inter-annual variability in climate is not considered. The Poisson model indicated that rainfall (lag 1-month), minimum temperature (lag six-months) and SOI (lag six6-months) were positively associated with JE cases. |
| Yip et al. (2022) | Malaysia | Niño 4 SST anomaly index | Vector-borne diseases | Correlation analysis | High | The authors report a positive correlation between dengue infection rate and El Niño 4 SST anomaly index, suggesting an increased incidence of the disease during El Niño events. |
| Adams et al. (2022) | Nepal | ONI: Oceanic Nino Index | Enteric infections and diseases (other than cholera) | Negative binomial Generalized Estimating Equations | High | El Niño was associated with a reduction in under-five diarrheal disease risk in the plains, hilly and mountainous regions of Nepal while La Niña was associated with an increase in the risk. |
| Kim et al. (2016) | Papua New Guinea | SOI: southern oscillation index / DMI: dipole mode index | Respiratory ailments | Parametric multivariate analysis using a generalized linear model | Low | Southern oscillation index and dipole mode index showed an overall negative effect on childhood pneumonia incidence, and the risk of pneumonia was higher in the dry season than in the rainy season. |
| Carvajal et al. (2018) | Philippines | SOI: southern oscillation index | Vector-borne diseases | Pearson’s correlation coefficient | High | The study exhibited that there are different predictive outcomes generated from different statistical modelling techniques such that the Random Forest model with delayed meteorological effects was the best in predicting the temporal pattern of Dengue incidence in Metropolitan Manila. The study found a small negative correlation between El Niño and the incidence rate of dengue. |
| Ehelepola et al. (2021) | Sri Lanka | SOI: southern oscillation index / Niño 3.4 / 4 SST anomaly index / MEI/ EMI / DMI: dipole mode index | Vector-borne diseases | De-trended cross correlation analysis | High | Results suggest that ENSO and IOD modulate Leptospirosis incidence (LI) in Kandy by modulating local rainfall and probably other weather parameters. Wavelet analysis displayed indices of ENSO, IOD, and ENSO Modoki were correlated with the LI with 1.9–11.5-month lags. |
| Liyanage et al. (2016) | Sri Lanka | ONI: Oceanic Nino Index | Vector-borne diseases | Meta-regression: pooled exposure response curves at different lag periods estimated using Poisson time series for each location | High | The strongest association with dengue risk centred around six to 10 weeks following rainfalls of more than 300 mm per week. With increasing temperature, the overall relative risk of dengue increased steadily starting from a lag of four weeks. Results suggest a strong link between the ONI and weather patterns in the district in Sri Lanka and to dengue at a longer latency time confirming these relationships. |
| Tiensuwan and O'Brien (2013) | Thailand | MEI: multivariate ENSO index | Vector-borne diseases | Multivariable regression with backward selections of covariates | High | The results show that for dengue fever (DF) and dengue haemorrhagic fever (DHF) the factors that affect infection rates are time, seasonal factors, and monthly multivariate ENSO index while the factors for dengue shock syndrome (DSS) are population in each month, seasonal factors, and monthly multivariate ENSO index. Dengue virus infection decreases for each severity of dengue disease with monthly multivariate ENSO index increases. |
| Tipayamongkholgul et al. (2009) | Thailand | MEI: multivariate ENSO index | Vector-borne diseases | Poisson autoregressive model | Low | El Niño has a positive and significant correlation with Dengue outbreaks, but a substantial geographical heterogeneity exists. |
| Kien et al. (2010) | Vietnam | ONI: Oceanic Nino Index | Enteric infections and diseases | Linear regression | High | The results suggest that climate factors (temperature and rainfall) and climate variability (El Niño) are associated with infectious disease burden in Vietnam. The correlation is higher in the specific geographical areas where climate parameters and anomalies are linked with the ENSO cycle. The associations are obvious with malaria and some waterborne diseases such as diarrheal diseases and dysentery. For influenza, cholera, and typhoid diseases, the correlation is not that obvious, but there is clear linkage between climate factors and the changing pattern of disease over the last 25 years. |
| Nguyen et al. (2020) | Vietnam | MEI: multivariate ENSO index/ DMI: dipole mode index | Vector-borne diseases | Generalized Additive Model | Low | The results suggest a positive impact of IOD and multivariate El Niño-Southern Oscillation index on vector index in dengue transmission in Khanh Hoa and Da Nang provinces. Regional climatic variables and mosquito population may drive dengue transmission in central Vietnam. |
| Hales et al. (1999) | Oceania | SOI: southern oscillation index | Vector-borne diseases | Correlations analysis | High | The study found that there were positive correlations between SOI and dengue in 10 countries. In five of these (including all of the larger islands) there were also positive corelations between SOI and estimates of local temperature and/or rainfall. |
| Andhikaputra et al. (2023) | Solomon Islands | ONI: Oceanic Nino Index | Vector-borne diseases | GLM - Negative binomial generalized estimating equations | High | La Niña was associated with increased dengue-like-illness risks in Guadalcanal Province, whereas El Niño was associated with risk reduction in both Guadalcanal and Western provinces. |

## Appendix C Examples of excluded studies

Based on our population-related inclusion criteria, we excluded studies that reported effects for broader geographic regions encompassing L&MIC and HIC populations, where effects for the former were not disaggregated (Hales, Weinstein, and Woodward 1996).

We found that studies measuring the relationship between EL Niño/+IOD on socio-economic outcomes use a variety of mostly quantitative methods and descriptive analyses. We excluded studies where the independent variable was a measure of an adverse event not explicitly attributed to EL Niño/+IOD; for instance, if the household reported losses due to a natural disaster even if the outcome was relevant such as the decision to migrate (Uyen et al. 2022). Of the very few studies that used a qualitative method, we excluded studies that did not use realist evaluation, general elimination methodology, process tracing or contribution analysis; or if they used other methods without outlining a theory of change between the climate driver and a socio-economic outcome (Savage et al. 2021). Some quantitative studies used a relevant climate driver proxy and data were available to provide a measure of the association of the relationship, but authors did not quantify it even when they used moving averages to describe differences between observed and expected values for outcomes (Irawan, 2002). Such studies were excluded using the EXCLUDE not a quantitative effectiveness study code.

In addition to a large number of regression and correlational studies that met our inclusion criteria, some used spatial models such as wavelet coherence analysis or multiple-regime panel smooth transition regression (Ubilava and Abdolrahimi 2019). Those were only included if they reported a quantitative estimate of association that could be extracted. Another frequently encountered group of designs were simulation studies. We included these only if authors reported results from the ex-post analysis (Reyes et al. 2009) but excluded them if only estimates derived from the predictive model were available (Aprilina et al. 2021). Finally, as mentioned in our limitations section, there were multiple cases where we found insufficient data but due to time limitations, we were not able to reach out to authors.

The table below reports how many records ended up being excluded due to near miss methods or missing data.

**Appendix Table 16. Number of excluded records by reason**

| Exclusion reason on full text | Records excluded |
| --- | --- |
| Visual/spatial analysis with no quant estimate | 18 |
| Simulation with no ex-post analysis | 18 |
| Missing data | 25 |

**List of excluded studies on near-miss methods or missing data**

Anderson, Weston Buckley. 2018. “Climate Variability Poses a Correlated Risk to Global Food Production.”

Aprilina, K., A. Susandi, A. Sopaheluwakan, and H. Harsa. 2021. “Study of the Effect of Climate Variation on Irrigated and Rainfed Rice Productivity Based on Aquacrop Crop Modelling Simulation (Case Study of Java Island).” In . Vol. 880. <https://doi.org/10.1088/1755-1315/880/1/012027>.

Apriyana, Yayan, E. Aldrian, and Y. Koesmaryono. 2019. “The Dynamics of Rice Cropping Calendar and Its Relation with the ENSO (El Nino-Southern Oscillation) and IOD (Indian Ocean Dipole) in Monsoon and Equatorial Regions of Indonesia.” In . Vol. 363. <https://doi.org/10.1088/1755-1315/363/1/012013>.

Asbjørn, Aaheim, and Linda Sygna. 2000. “Economic Impacts of Climate Change on Tuna Fisheries in Fiji Islands and Kiribati.” *NA* NA (NA): NA-NA.

Bahri, Muhamad Khairul. 2017. “Integrating Statistical and System Dynamics Modelling to Analyse the Impacts of Climate Change on Rice Production in West Nusa Tenggara, Indonesia.” openaccess.wgtn.ac.nz. [https://openaccess.wgtn.ac.nz/articles/thesis/Integrating_Statistical_and_System_Dynamics_Modelling_to_Analyse_the_Impacts_of_Climate_Change_on_Rice_Production_in_West_Nusa_Tenggara_Indonesia/17060483 https://openaccess.wgtn.ac.nz/articles/thesis/Integrating_Statistical_and_System_Dynamics_Modelling_to_Analyse_the_Impacts_of_Climate_Change_on_Rice_Production_in_West_Nusa_Tenggara_Indonesia/17060483/1/files/31548269.pdf](https://openaccess.wgtn.ac.nz/articles/thesis/Integrating_Statistical_and_System_Dynamics_Modelling_to_Analyse_the_Impacts_of_Climate_Change_on_Rice_Production_in_West_Nusa_Tenggara_Indonesia/17060483%20https:/openaccess.wgtn.ac.nz/articles/thesis/Integrating_Statistical_and_System_Dynamics_Modelling_to_Analyse_the_Impacts_of_Climate_Change_on_Rice_Production_in_West_Nusa_Tenggara_Indonesia/17060483/1/files/31548269.pdf).

Bouma, Menno J., and H. J. van der Kaay. 1996. “The El Niño Southern Oscillation and the Historic Malaria Epidemics on the Indian Subcontinent and Sri Lanka: An Early Warning System for Future Epidemics?” *Tropical Medicine & International Health* 1 (1): 86–96. <https://doi.org/10.1046/j.1365-3156.1996.d01-7.x>.

Busnita, Silvia Sari, R. Oktaviani, and T. Novianti. 2017. “How Far Climate Change Affects the Indonesian Paddy Production and Rice Price Volatility?” *International Journal of …*. [http://ijasc.pasca.unand.ac.id/index.php/ijac/article/view/29 http://ijasc.pasca.unand.ac.id/index.php/ijac/article/download/29/22](http://ijasc.pasca.unand.ac.id/index.php/ijac/article/view/29%20http:/ijasc.pasca.unand.ac.id/index.php/ijac/article/download/29/22).

Callahan, Christopher W., and Justin S. Mankin. 2023. “Persistent Effect of El Niño on Global Economic Growth.” *Science* 380 (6649): 1064–69. <https://doi.org/10.1126/science.adf2983>.

Cao, Juan, Z. Zhang, F. Tao, Y. Chen, X. Luo, and J. Xie. 2023. “Forecasting Global Crop Yields Based on El Nino Southern Oscillation Early Signals.” *Agricultural Systems* 205. <https://doi.org/10.1016/j.agsy.2022.103564>.

Cash, Benjamin A., X. Rodó, and J. L. Kinter III. 2008. “Links between Tropical Pacific SST and Cholera Incidence in Bangladesh: Role of the Eastern and Central Tropical Pacific.” *Journal of Climate* 21 (18): 4647–63. <https://doi.org/10.1175/2007JCLI2001.1>.

Cashin, Paul, Kamiar Mohaddes, and Mehdi Raissi. 2014. “Fair Weather or Foul? The Macroeconomic Effects of El Nino.”

Cazelles, Bernard, M. Chavez, A. J. McMichael, and S. Hales. 2005. “Nonstationary Influence of El Niño on the Synchronous Dengue Epidemics in Thailand.” *PLoS Medicine* 2:0313–18. <https://doi.org/10.1371/journal.pmed.0020106>.

Chapman, Ross, J. Cock, M. Samson, N. Janetski, K. Janetski, D. Gusyana, S. Dutta, and T. Oberthür. 2021. “Crop Response to El Niño-Southern Oscillation Related Weather Variation to Help Farmers Manage Their Crops.” *Scientific Reports* 11 (1). <https://doi.org/10.1038/s41598-021-87520-4>.

Chaturvedi, Shweta, and Suneet Dwivedi. 2023. “Impact of El Niño–Southern Oscillation and Indian Ocean Dipole on Malaria Transmission over India in Changing Climate.” *International Journal of Environmental Science and Technology* 21 (1): 91–100. <https://doi.org/10.1007/s13762-023-04836-6>.

Chau, Nguyen Hai, and Le Thi Ngoc Anh. 2016. “Using Local Weather and Geographical Information to Predict Cholera Outbreaks in Hanoi, Vietnam.” In *Advanced Computational Methods for Knowledge Engineering*, 212. <https://doi.org/10.1007/978-3-319-38884-7_15>.

Chen, Chi-Chung, Bruce A. McCarl, and Ching-Cheng Chang. 2008. “Strong El Niño–Southern Oscillation Events and the Economics of the International Rice Market.” *Climate Research* 36 (2): 113–22. <https://doi.org/10.3354/cr00738>.

Cherian, Shilpa, S. Sridhara, K. N. Manoj, P. Gopakkali, N. Ramesh, A. A. Alrajhi, A. Z. Dewidar, and M. A. Mattar. 2021. “Impact of El Niño Southern Oscillation on Rainfall and Rice Production: A Micro-Level Analysis.” *Agronomy* 11 (6). <https://doi.org/10.3390/agronomy11061021>.

Cobon, David H., M. Ewai, K. Inape, and R. M. Bourke. 2016. “Food Shortages Are Associated with Droughts, Floods, Frosts and ENSO in Papua New Guinea.” *Agricultural Systems* 145:150–64. <https://doi.org/10.1016/j.agsy.2016.02.012>.

Dawe, David, Piedad F. Moya, and Shiela Valencia. 2008. “Institutional, Policy and Farmer Responses to Drought: El Niño Events and Rice in the Philippines.” *Disasters* 33 (2): 291–307. <https://doi.org/10.1111/j.1467-7717.2008.01075.x>.

Dhiman, Ramesh C., and Soma Sarkar. 2017. “El Niño Southern Oscillation as an Early Warning Tool for Malaria Outbreaks in India.” *Malaria Journal* 16 (1). <https://doi.org/10.1186/s12936-017-1779-y>.

Ehelepola, N. D. B., K. Ariyaratne, and D. S. Dissanayake. 2021. “The Interrelationship between Meteorological Parameters and Leptospirosis Incidence in Hambantota District, Sri Lanka 2008-2017 and Practical Implications.” *PLoS ONE* 16 (1 January). <https://doi.org/10.1371/journal.pone.0245366>.

Generoso, Rémi, C. Couharde, O. Damette, and K. Mohaddes. 2020. “The Growth Effects of El Niño and La Niña: Local Weather Conditions Matter.” *Annals of Economics and Statistics*, no. 140, 83–126. <https://doi.org/10.15609/ANNAECONSTAT2009.140.0083>.

Hadi, Prayogo U., and Istiqlal Amien. 2010. “Effect of El Nino on Food Security in Indonesia.” *Palawija News* 27 (2): 1–5.

Hashizume, Masahiro, L. F. Chaves, A. S. G. Faruque, Md. Yunus, K. Streatfield, and K. Moji. 2013. “A Differential Effect of Indian Ocean Dipole and El Niño on Cholera Dynamics in Bangladesh.” *PLoS ONE* 8 (3). <https://doi.org/10.1371/journal.pone.0060001>.

Hasudungan, P., I. Irham, and A. W. Utami. 2021. “The Impact of El Nino Southern Oscillation and Covid-19 on the Rice Price Dynamics in Indonesia: The Vector Error Correction Model Approach.” In . Vol. 883. <https://doi.org/10.1088/1755-1315/883/1/012061>.

Hay, S. I., M. F. Myers, D. S. Burke, D. W. Vaughn, T. Endy, N. Ananda, G. D. Shanks, R. W. Snow, and D. J. Rogers. 2000. “Etiology of Interepidemic Periods of Mosquito-Borne Disease.” *Proceedings of the …*. <https://doi.org/10.1073/pnas.97.16.9335>.

Heino, Matias, Joseph H. A. Guillaume, Christoph Müller, Toshichika Iizumi, and Matti Kummu. 2020. “A Multi-Model Analysis of Teleconnected Crop Yield Variability in a Range of Cropping Systems.” *Earth System Dynamics* 11 (1): 113–28. <https://doi.org/10.5194/esd-11-113-2020>.

Herho, Sandy H. S., Brahmana Ferio, Katarina E. P. Herho, and Dasapta. E. Irawan. 2021. “Does ENSO Significantly Affect Rice Production In Indonesia? A Preliminary Study Using Computational Time-Series Approach.” *International Journal of Data Science* 2 (2): 69–76. <https://doi.org/10.18517/ijods.2.2.69-76.2021>.

Herho, SHS, F Brahmana, and KEP Herho. 2021. “Does ENSO Significantly Affect Rice Production in Indonesia? A Preliminary Study Using Computational Time-Series Approach.” *International Journal of …*. [http://www.ijods.org/index.php/ds/article/view/28 http://www.ijods.org/index.php/ds/article/download/28/23](http://www.ijods.org/index.php/ds/article/view/28%20http:/www.ijods.org/index.php/ds/article/download/28/23).

Iizumi, Toshichika, J. J. Luo, A. J. Challinor, G. Sakurai, M. Yokozawa, H. Sakuma, M. E. Brown, and T. Yamagata. 2014. “Impacts of El Niño Southern Oscillation on the Global Yields of Major Crops.” *Nature Communications* 5. <https://doi.org/10.1038/ncomms4712>.

Imai, Chisato, H. K. Cheong, H. Kim, Y. Honda, J. H. Eum, C. T. Kim, J. S. Kim, et al. 2016. “Associations between Malaria and Local and Global Climate Variability in Five Regions in Papua New Guinea.” *Tropical Medicine and Health* 44 (1). <https://doi.org/10.1186/s41182-016-0021-x>.

Keil, Alwin, N. Teufel, D. Gunawan, and C. Leemhuis. 2009. “Vulnerability of Smallholder Farmers to ENSO-Related Drought in Indonesia.” *Climate Research* 38 (2): 155–69. <https://doi.org/10.3354/cr00778>.

Koelle Katia. 2009. “The Impact of Climate on the Disease Dynamics of Cholera.” *Clinical Microbiology and Infection : The Official Publication of the European Society of Clinical Microbiology and Infectious Diseases* 15 (NA): 29–31. <https://doi.org/10.1111/j.1469-0691.2008.02686.x>.

Koelle, Katia, X. Rodó, M. Pascual, Md. Yunus, and G. Mostafa. 2005. “Refractory Periods and Climate Forcing in Cholera Dynamics.” *Nature* 436 (7051): 696–700. <https://doi.org/10.1038/nature03820>.

Kurnianingsih, Anindya Wirasatriya, Lutfan Lazuardi, Naoyuki Kubota, and Nawi Ng. 2020a. “CcS - IOD and ENSO-Related Time Series Variability and Forecasting of Dengue and Malaria Incidence in Indonesia.” *2020 International Symposium on Community-Centric Systems (CcS)* NA (NA): 1–8. <https://doi.org/10.1109/ccs49175.2020.9231358>.

———. 2020b. “IOD and ENSO-Related Time Series Variability and Forecasting of Dengue and Malaria Incidence in Indonesia.” In . <https://doi.org/10.1109/CcS49175.2020.9231358>.

Liyanage, Prasad, Y. Tozan, H. J. Overgaard, H. A. Tissera, and J. Rocklöv. 2022. “Effect of El Niño–Southern Oscillation and Local Weather on Aedes Dvector Activity from 2010 to 2018 in Kalutara District, Sri Lanka: A Two-Stage Hierarchical Analysis.” *The Lancet Planetary Health* 6 (7): e577–85. <https://doi.org/10.1016/S2542-5196(22)00143-7>.

Liyantono, T. Kato, K. Yoshida, and H. Kuroda. 2012. “The Influence of El Nino Southern Oscillation on Agricultural Production Sustainability in a Tropical Monsoon Region: Case Study in Nganjuk District, East Java, Indonesia.” *Journal of Developments in Sustainable Agriculture* 7 (1): 65–74.

Lobell, David B., Wolfram Schlenker, and Justin Costa-Roberts. 2011. “Climate Trends and Global Crop Production Since 1980.” *Science (New York, N.Y.)* 333 (6042): 616–20. <https://doi.org/10.1126/science.1204531>.

McIver, Lachlan, M. Hashizume, H. Kim, Y. Honda, M. Pretrick, S. Iddings, and B. Pavlin. 2015. “Assessment of Climate-Sensitive Infectious Diseases in the Federated States of Micronesia.” *Tropical Medicine and Health* 43 (1): 29–40. <https://doi.org/10.2149/tmh.2014-17>.

Naylor, Rosamond L., David S. Battisti, Daniel J. Vimont, and Walter P. Falcon. 2009. “Final Report: Agricultural Decision-Making in Indonesia with ENSO Variability: Integrating Climate Science, Risk Assessment, and Policy Analysis.” *NA* NA (NA): NA-NA.

Nizamuddin, Mohammad. 2010. “Hybrid Application of AVHRR Based Satellite Remote Sensing and ENSO Signals for Early Warning and Monitoring of Malaria in Asia and South America.”

Pascual, Mercedes, X. Rodo, S. P. Ellner, R. Colwell, and M. J. Bouma. 2000. “Cholera Dynamics and El Nino-Southern Oscillation.” *Science* 289 (5485): 1766–69. <https://doi.org/10.1126/science.289.5485.1766>.

Phung, Vera Ling Hui, K. Oka, Y. Hijioka, K. Ueda, M. Sahani, and W. R. W. Mahiyuddin. 2022. “Environmental Variable Importance for Under-Five Mortality in Malaysia: A Random Forest Approach.” *SCIENCE OF THE TOTAL ENVIRONMENT* 845. <https://doi.org/10.1016/j.scitotenv.2022.157312>.

Qian, Yonglan, J. Zhao, S. Zheng, Y. Cao, and L. Xue. 2020. “Risk Assessment of the Global Crop Loss in ENSO Events.” *Physics and Chemistry of the Earth* 116. <https://doi.org/10.1016/j.pce.2020.102845>.

Rees, Eleanor. M., Martin Lotto Batista, Mike Kama, Adam J. Kucharski, Colleen. L Lau, and Rachel Lowe. 2023. “Quantifying the Relationship between Climatic Indicators and Leptospirosis Incidence in Fiji: A Modelling Study.” *PLOS Global Public Health* 3 (10): e0002400. <https://doi.org/10.1371/journal.pgph.0002400>.

Shackleton, Debbie, F. A. Memon, A. Chen, S. Dutta, S. Kanungo, and A. Deb. 2023. “The Changing Relationship between Cholera and Interannual Climate Variables in Kolkata over the Past Century.” *Gut Pathogens* 15 (1). <https://doi.org/10.1186/s13099-023-00565-w>.

Sutton, William R., J. P. Srivastava, J. Koo, I. Vasileiou, and A. Pradesha. 2019. “Striking a Balance: Managing El Nino and La Nina in Cambodia’s Agriculture.” *Striking a Balance: Managing El Nino and La Nina in Cambodia’s Agriculture*, 113-pp.

Sutton, William R., J. P. Srivastava, M. Rosegrant, J. Koo, and R. Robertson. 2019a. “Striking a Balance: Managing El Nino and La Nina in Lao PDR’s Agriculture.” *Striking a Balance: Managing El Nino and La Nina in Lao PDR’s Agriculture*, 107-pp.

———. 2019b. “Striking a Balance: Managing El Niño and La Niña in Lao PDR’s Agriculture | PreventionWeb.” 2019. <https://www.preventionweb.net/publication/striking-balance-managing-el-nino-and-la-nina-lao-pdrs-agriculture>.

Sutton, William R., J. P. Srivastava, M. Rosegrant, J. Thurlow, and L. Sebastian. 2019. “Striking a Balance: Managing El Nino and La Nina in Vietnam’s Agriculture.” *Striking a Balance: Managing El Nino and La Nina in Vietnam’s Agriculture*, 103-pp.

Sutton, William R., J. P. Srivastava, M. Rosegrant, R. Valmonte-Santos, and M. Ashwill. 2019. “Striking a Balance: Managing El Nino and La Nina in Philippines’ Agriculture.” *Striking a Balance: Managing El Nino and La Nina in Philippines’ Agriculture*, xxii-pp.

Sutton, William R., J. P. Srivastava, M. W. Rosegrant, J. Thurlow, and L. Sebastian. n.d. “Striking a Balance: Managing El Niño and La Niña in Vietnam’s Agriculture | IFPRI : International Food Policy Research Institute.” <https://www.ifpri.org/publication/striking-balance-managing-el-ni%C3%B1o-and-la-ni%C3%B1a-vietnams-agriculture>.

Sutton, William R., J. P. Srivastava, M. W. Rosegrant, J. Thurlow, and I. Vasileiou. 2019. “Striking a Balance: Managing El Nino and La Nina in Myanmar’s Agriculture.” *Striking a Balance: Managing El Nino and La Nina in Myanmar’s Agriculture*, 118-pp.

Syamsuddin, Mega, S. I. Saitoh, T. Hirawake, F. Syamsudin, and M. Zainuddin. 2016. “Interannual Variation of Bigeye Tuna (Thunnus Obesus) Hotspots in the Eastern Indian Ocean off Java.” *International Journal of Remote Sensing* 37 (9): 2087–2100. <https://doi.org/10.1080/01431161.2015.1136451>.

Syamsuddin, Mega, Sunarto, and Lintang Permata Sari Yuliadi. 2018. “How Do El Niño Southern Oscillation Events Impact on Small Pelagic Fish Catches in the West Java Sea.” *IOP Conference Series: Earth and Environmental Science* 176 (1): 012014-NA. <https://doi.org/10.1088/1755-1315/176/1/012014>.

Thai, Khoa T. D., B. Cazelles, N. van Nguyen, L. T. Vo, M. F. Boni, J. Farrar, C. P. Simmons, H. R. van Doorn, and P. J. de Vries. 2010. “Dengue Dynamics in Binh Thuan Province, Southern Vietnam: Periodicity, Synchronicity and Climate Variability.” *PLoS Neglected Tropical Diseases* 4 (7). <https://doi.org/10.1371/journal.pntd.0000747>.

Ubilava, David, and Maryam Abdolrahimi. 2019. “The El Niño Impact on Maize Yields Is Amplified in Lower Income Teleconnected Countries.” *Environmental Research Letters* 14 (5). <https://doi.org/10.1088/1748-9326/ab0cd0>.

Ubilava, David, and Nelson Villoria. 2013. “Do the Trade Winds Alter the Trade Flow? Assessing Impacts of ENSO Shocks on World Cereal Supply.” <https://doi.org/10.22004/AG.ECON.150516>.

Villoria, Nelson B., and Michael Delgado. 2017. “Worldwide Crop Supply Responses to El Niño Southern Oscillation.” <https://doi.org/10.22004/AG.ECON.258564>.

Zhang, Li, Yan Li, Sixin Yu, and Lu Wang. 2023. “Risk Transmission of El Niño-Induced Climate Change to Regional Green Economy Index.” *Economic Analysis and Policy* 79:860–72. <https://doi.org/10.1016/j.eap.2023.07.006>.

## Appendix D Calculating standardised effects

We computed standardized mean differences (SMDs) for continuous outcomes using the formulae by Borenstein 2009), also known as Cohen's d.
$d= \frac{x_{Tp+1}+ x_{Cp+1}}{SD}$

(FORMULA #1)

We then adjusted it to Hedges' g to deal with bias arising from small sample size using the formula by Ellis (2010):
$g\cong d \left( 1- \frac{3}{4\left( n_{T} + n_{C} \right)-9} \right)$

(FORMULA #2)

For studies reporting regression results, we followed the approach suggested by Keef and Roberts (2004) using the regression coefficient and the pooled standard deviation of the outcome. Where the pooled standard deviation of the outcome is unavailable, we will use regression coefficients and standard errors or t-statistics to do the following, where sample size information is available in each group:

(FORMULA #3)

$$d=t\sqrt{\frac{1}{n_{T}}+ \frac{1}{n_{C}}}$$

where *n* denotes the sample size of treatment group and control. We used the following where total sample size information (*N*) is available only (as suggested in (Polanin and Snilstveit 2016):

(FORMULA #4)

$$d= \frac{2t}{\surd N} {Var}_{d}=\frac{4}{N}+ \frac{d^{2}}{2N}$$

When necessary, we calculated the t-statistic (*t*) by dividing the coefficient by the standard error. If the authors only reported confidence intervals and no standard error, we calculated the standard error from the confidence intervals using the following:

$SD=\sqrt{N}\times\frac{\left( upper limit-lower limit \right)}{3.92}$

(FORMULA #5)

*For 90% confidence intervals 3.92 should be replaced by 3.29, and for 99% confidence intervals it should be replaced by 5.15.*

If the study did not report the standard error, but did report *t,* we extracted and used this as reported by the authors. In cases in which significance levels were reported as a range rather than *t* or se(b), then *t* was imputed as follows:

- Prob > 0.1:  *t* = 0.5
- Prob ≤ 0.1 to Prob > 0.05:  *t* = 1.8

(FORMULA #6)

- Prob ≤ 0.05 to Prob > 0.01:  *t* = 2.4
- Prob ≤ 0.01 :   *t* = 2.8.

If an exact p-value was reported, we used the following Excel function to determine the t-value.

(FORMULA #7)

=T.INV.2T(exact p value, (n-1))

Where outcomes are reported in proportions of individuals, we will calculate the Cox-transformed log odds ratio effect size (Sánchez-Meca, Marín-Martínez, and Chacón-Moscoso 2003):

(FORMULA #8)

$$d=LogOddsRatiov x \frac{\sqrt{3}}{\pi}$$

where OR is the odds ratio calculated from the two-by-two frequency table.

Where outcomes were reported based on proportions of events or days, we used the standardised proportion difference effect size:


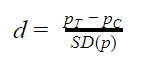


(FORMULA #9)

where *p_t_* is the proportion in the treatment group and *p_c_* the proportion in the comparison group, and the denominator is given by:


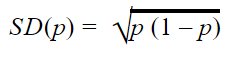


(FORMULA #10)

where p is the weighted average of *p_c_* and *p_t_*:

(FORMULA #11)

$$p= \frac{n_{T} p_{T}+ n_{C} p_{C}}{n_{T}+ n_{C}}$$

In all cases after synthesis, we converted pooled effect sizes to commonly used metrics such as percentage changes and mean differences in outcomes metrics typically used (e.g. weight in kg).

## Appendix E Risk of bias assessment tool

**Appendix Table 17. Risk of bias assessment tool**

| 1. Study design | | | | | |
| --- | --- | --- | --- | --- | --- |
| Was the analytical approach reasonable for the research question specific to the effect size extracted for this analysis? | Were tests supporting the identification strategy, selection of methods or results reported? | | Was there adequate adjustment for confounding in the analyses? | | Justification for the answers to the study design section. |
| 1=Yes, 2=No, 3=Unclear, 4=N/A  Code ‘yes’ if authors justify their choice of methods considering the data availability and the research question. Code ‘no’ if authors do not use panel data methods or time series methods when they have this data sets. | 1=Yes, 2=No, 3=Unclear, 4=N/A  Code ‘yes’ for studies that report tests of assumptions, use goodness-of-fit measures for model selection, conduct robustness checks, perform sensitivity tests, assess prediction power, or provide other evidence supporting the study design. | | 1=Yes, 2=No, 3=Unclear, 4=N/A  Code ‘yes’ if authors identify and control for potential confounding factors in their analysis. | | Free text. Include page numbers for verbatim or quotes from the text that help justify your coding. |
| **2. Data quality** | | | | | |
| Were outcome measures objective and free from measurement issues? | | Was the frequency and length of the data appropriate to answer the research question? | | Justification for the answers to the data quality section. | |
| 1=Yes, 2=No, 3=Unclear, 4=N/A  Code ‘yes’ if authors use publicly available data from official sources. Code ‘no’ if authors use self-reported outcomes from surveys design specifically for the purpose of the study. | | 1=Yes, 2=No, 3=Unclear, 4=N/A  Appropriate frequency: monthly or considering El Niño/+IOD cyclicity does not take place on a calendar year (the authors use indexes for specific months, or use monthly data). Code ‘no’ if calendar year averages of the climate driver index are used.  Appropriate length: At least two occurrences of the climate driver | | Free text. Include page numbers for verbatim or quotes from the text that help justify your coding. | |
| **3. Selective Reporting** | | | | | |
| Were conclusions consistent with the unit of analysis and reported results? | | Did all reported results correspond to all intended analyses, avoiding "data dredging" and selective reporting? | | Justification for the answers to the reporting section. | |
| 1=Yes, 2=No, 3=Unclear, 4=N/A | | 1=Yes, 2=No, 3=Unclear, 4=N/A | | Free text. Include page numbers for verbatim or quotes from the text that help justify your coding. | |

Source: Waddington, Snilstveit, et al. (2014)

## Appendix F Preferred Reporting Items for Systematic Reviews and Meta-Analysis (PRISMA diagram of systematic search and screening)

**
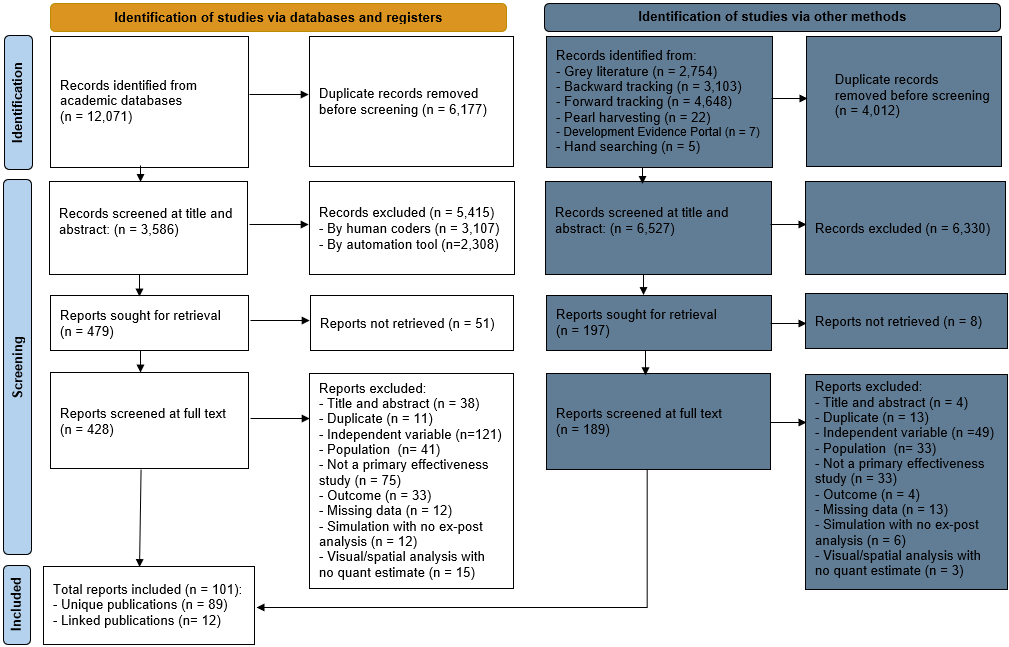
**

*Note: We identified several dozen studies with full text in a non-English language. After translating them by staff or external colleagues fluent in that language, only one (Utami et. al. 2011) met our inclusion criteria and was subsequently included in our analysis.*

## Appendix G Forest plots

#### **A.7.1 Forest plots of outcomes included in meta-analysis**

**Figure A.7. 1.1. Effects of El Niño on production**


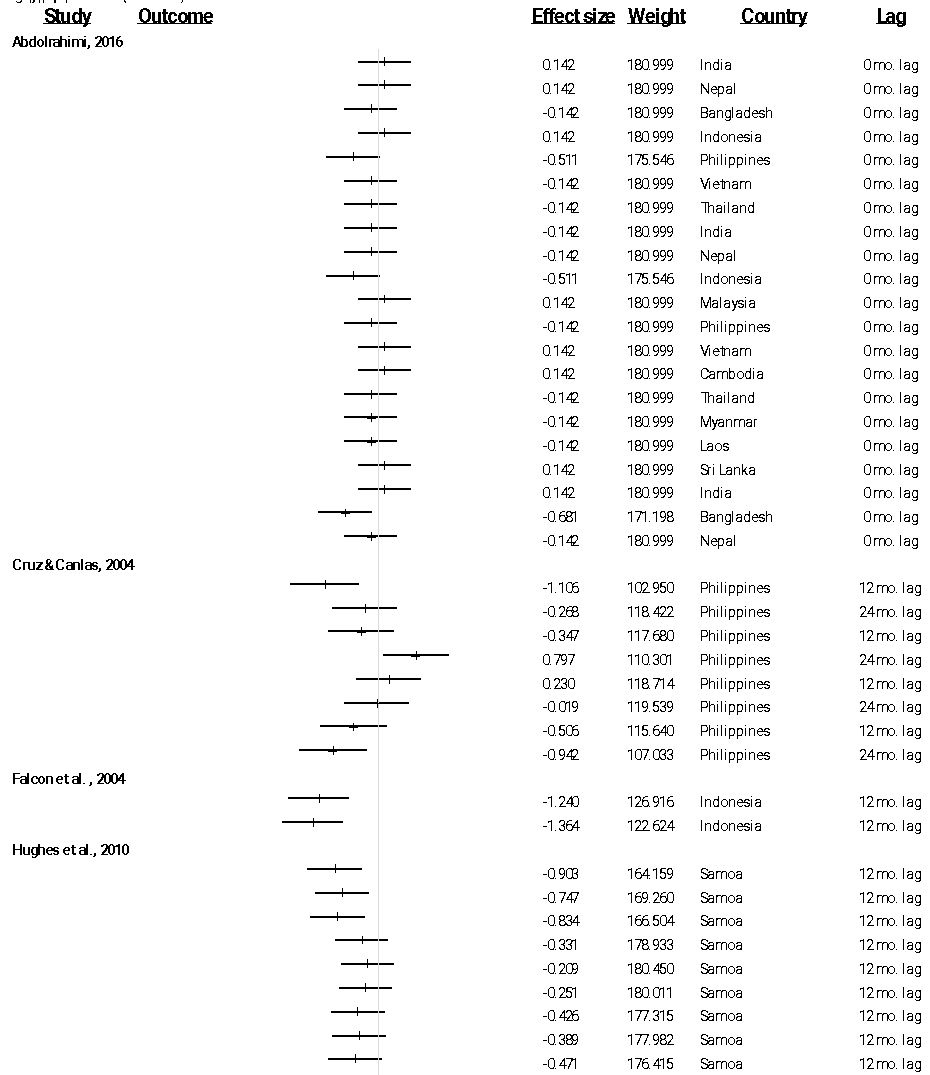


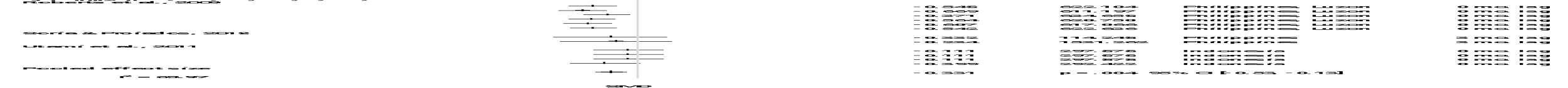


*Source: 3ie (2025)*

**Figure A.7.1.1 *continues***
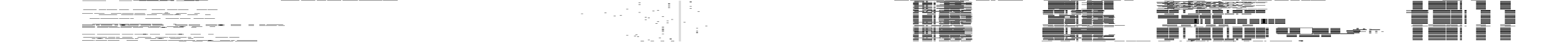

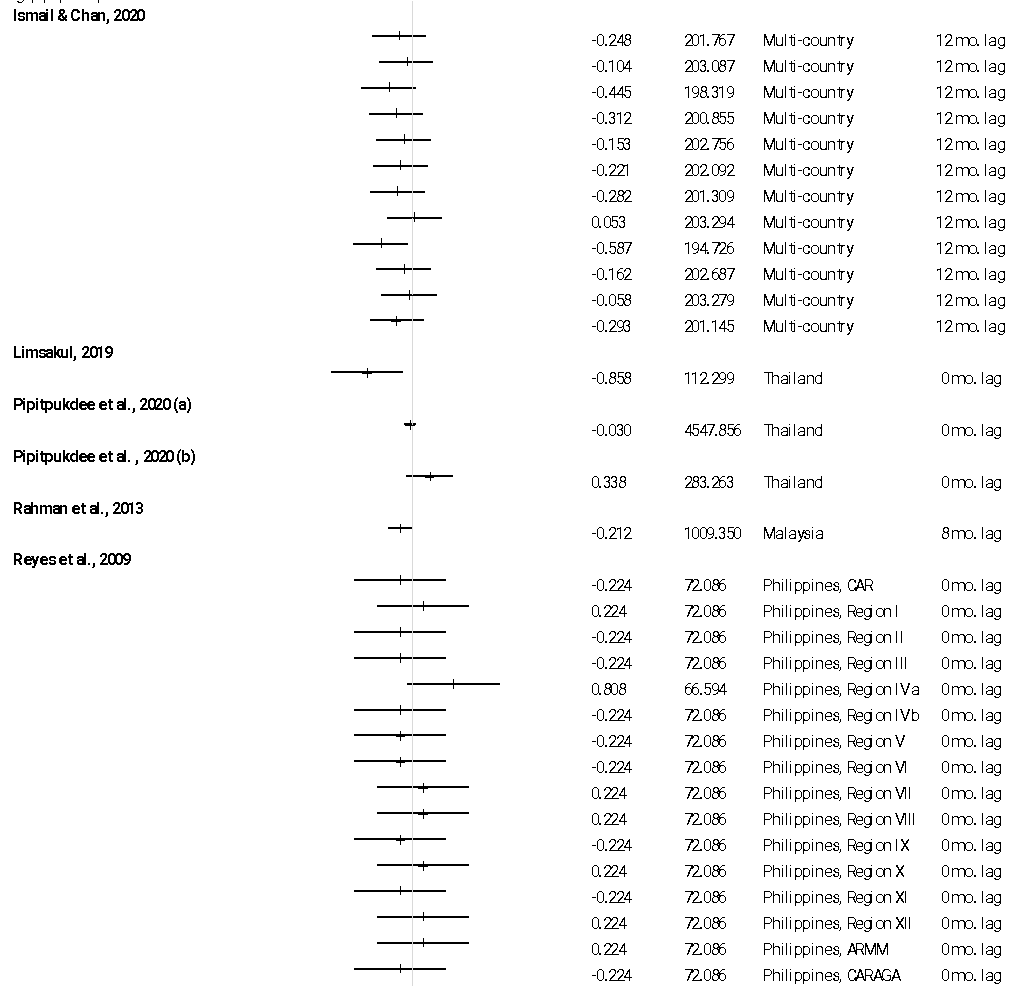

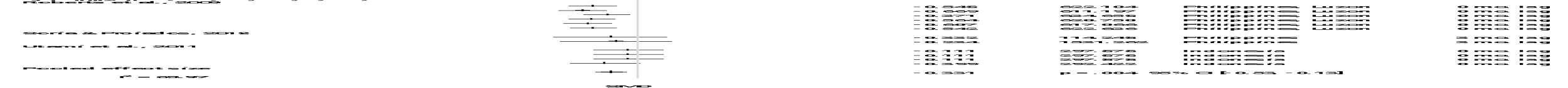


*Source: 3ie (2025)*

**Figure A.7.1.1 *continues***
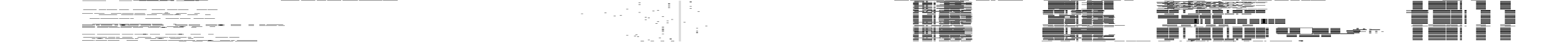


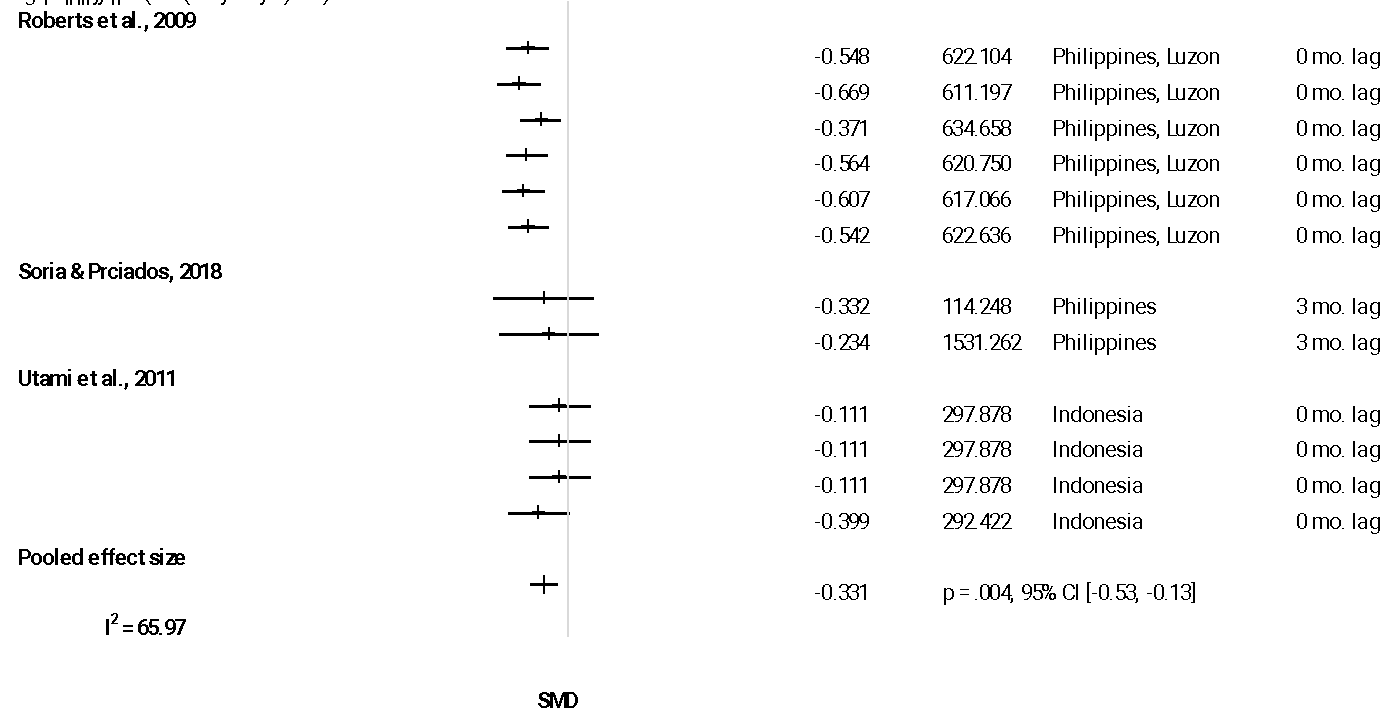


*Source: 3ie (2025)*

*Note: * indicates some concerns and low-risk of bias. Two studies using ANOVA to estimate the effect of El Niño on production were not included in the meta-analysis because of not reporting all the information needed to estimate the SMD (Suratno 2022; Bertrand et al. 2023).*

**Figure A.7.1. 2. Effects of El Niño on productivity** *
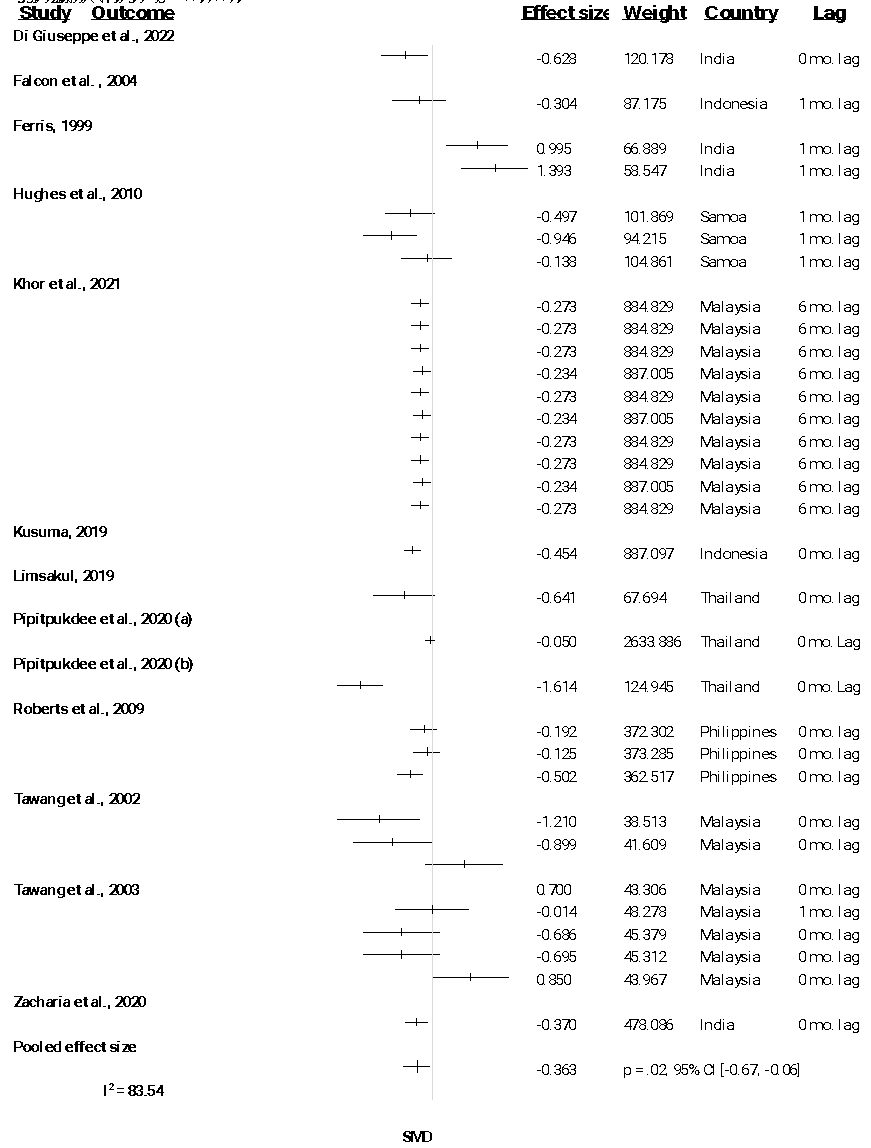
***
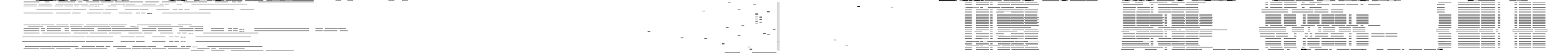
**

*Source: 3ie (2025)*

**Figure A.7.1.2 continues**

**
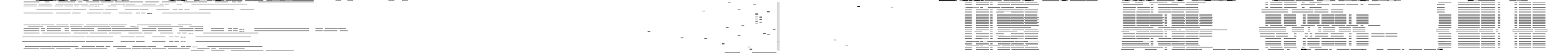
***
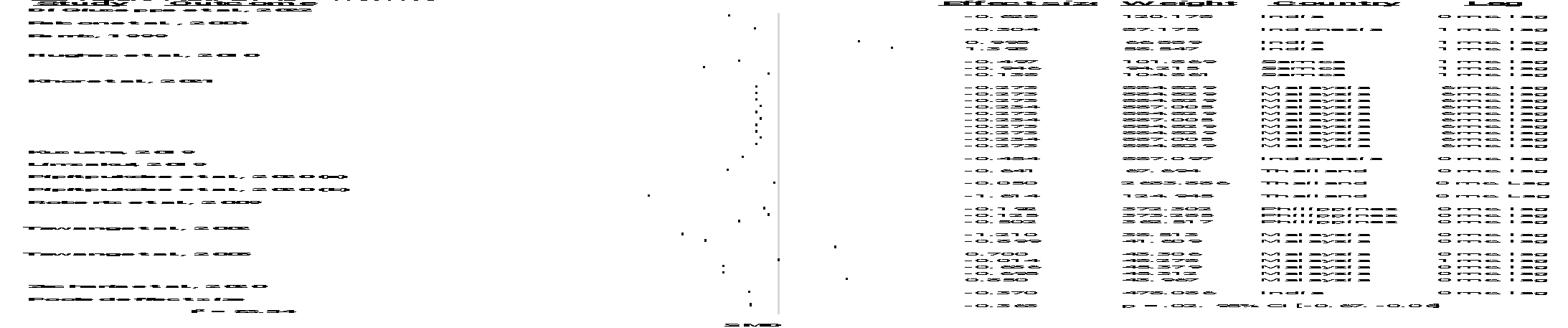
*

*Source: 3ie (2025)*

*Note: * indicates some concerns and low-risk of bias. Four studies using regression analysis to estimate the effect of El Niño on productivity outcomes were not included in the meta-analysis because of not reporting all the information needed to estimate the SMDs (Ghose et al. 2021; Nugroho 2013; Rao et al. 2012; Kusuma 2019).*

**Figure A.7.1.3. Effects of El Niño on prices**


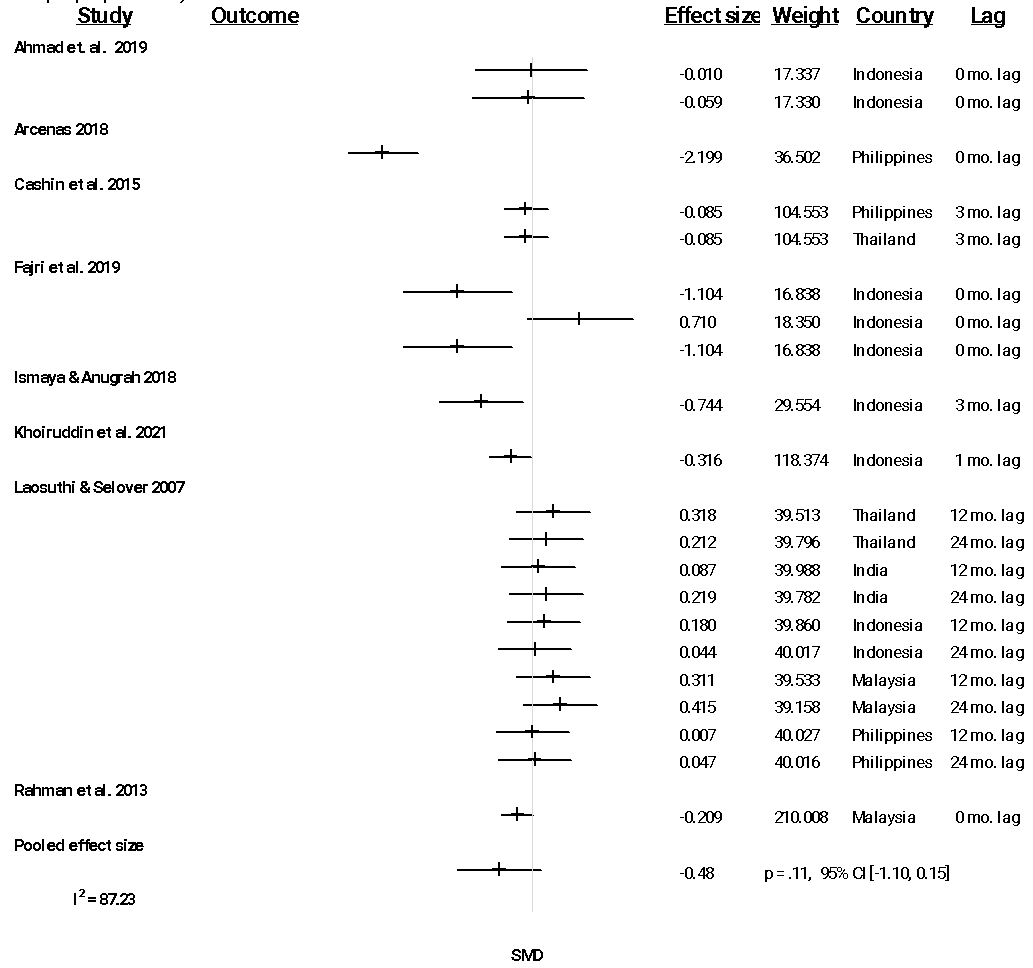


*Source: 3ie (2025)*

*Note: * indicates some concerns and low-risk of bias. One study using regression analysis to estimate the effect of El Niño on prices was not included in the meta-analysis because of not reporting all the information needed to estimate the SMDs (Selvaraju 2003).*

**Figure A.7.1.4. Effects of El Niño on vector-borne diseases**


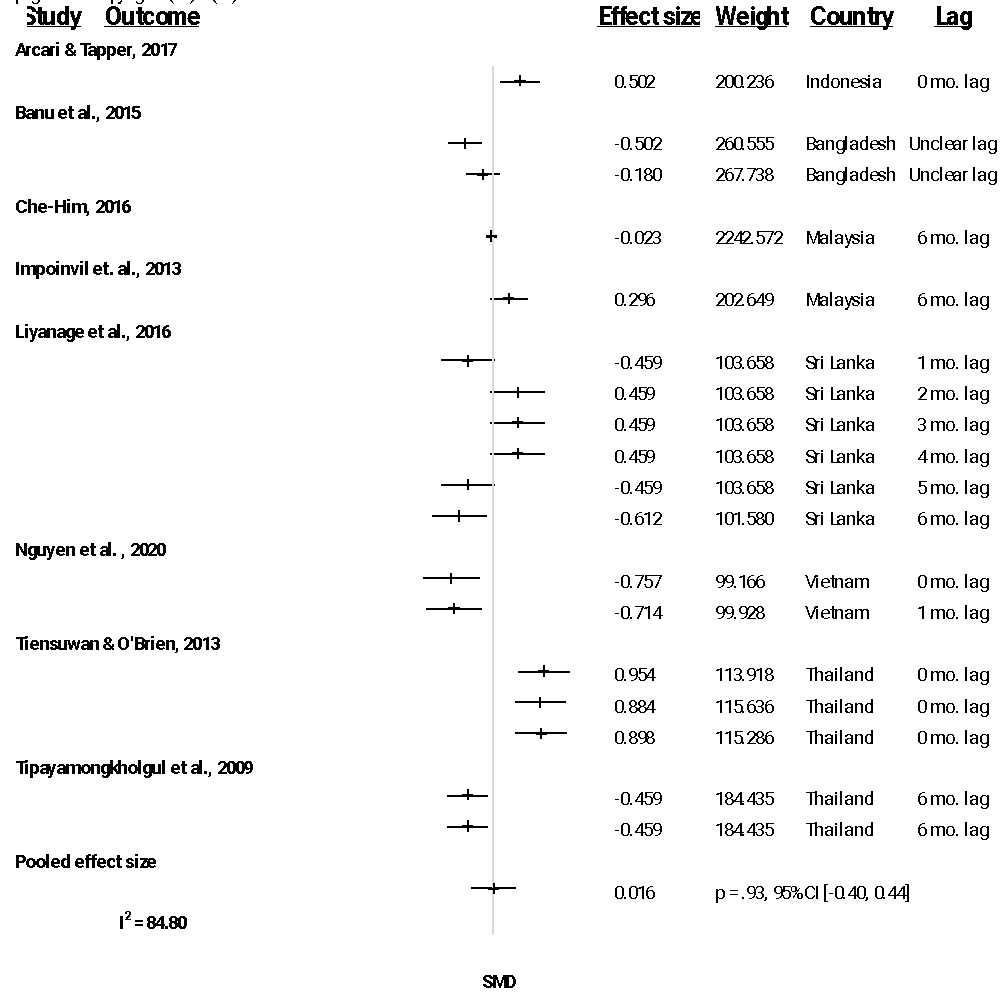


*Source: 3ie (2025)*

*Note: * indicates some concerns and low-risk of bias. Three studies using negative binomial models to estimate the effect of El Niño on vector-borne disease incidence were not included in the meta-analysis because of not reporting all the information needed to estimate the SMDs (Andhikaputra et al.2023; Sharmin et al. 2016; Yip et al. 2022).*

#### **A.7.2 Forest plots of outcomes not included in meta-analysis**

**Effects of El Niño on Economic Outcomes**

**Figure A.7.2.1. Effects of El Niño on aggregate production**


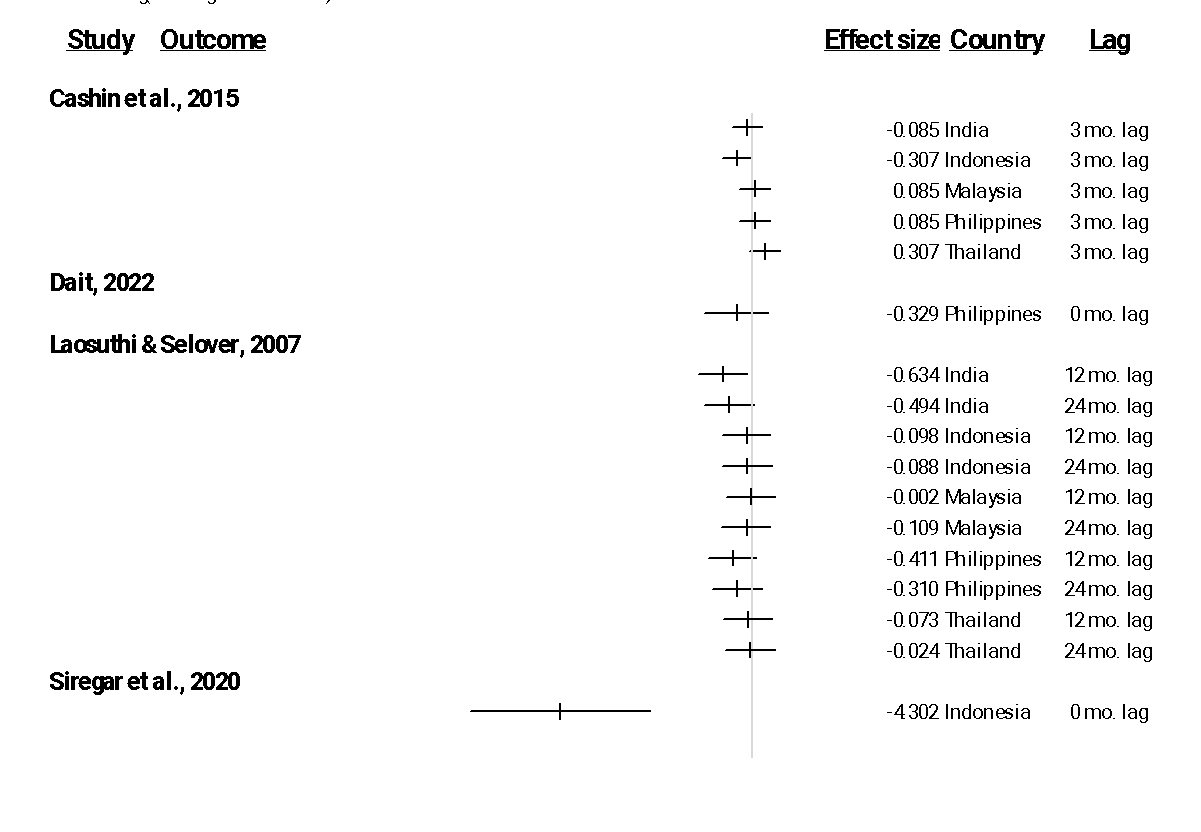


*Source: 3ie (2025)*

*Note: * indicates some concerns and low-risk of bias. Not enough degrees of freedom to estimate a pooled effect.*

**Figure A.7.2.2. Effects of El Niño on investments**


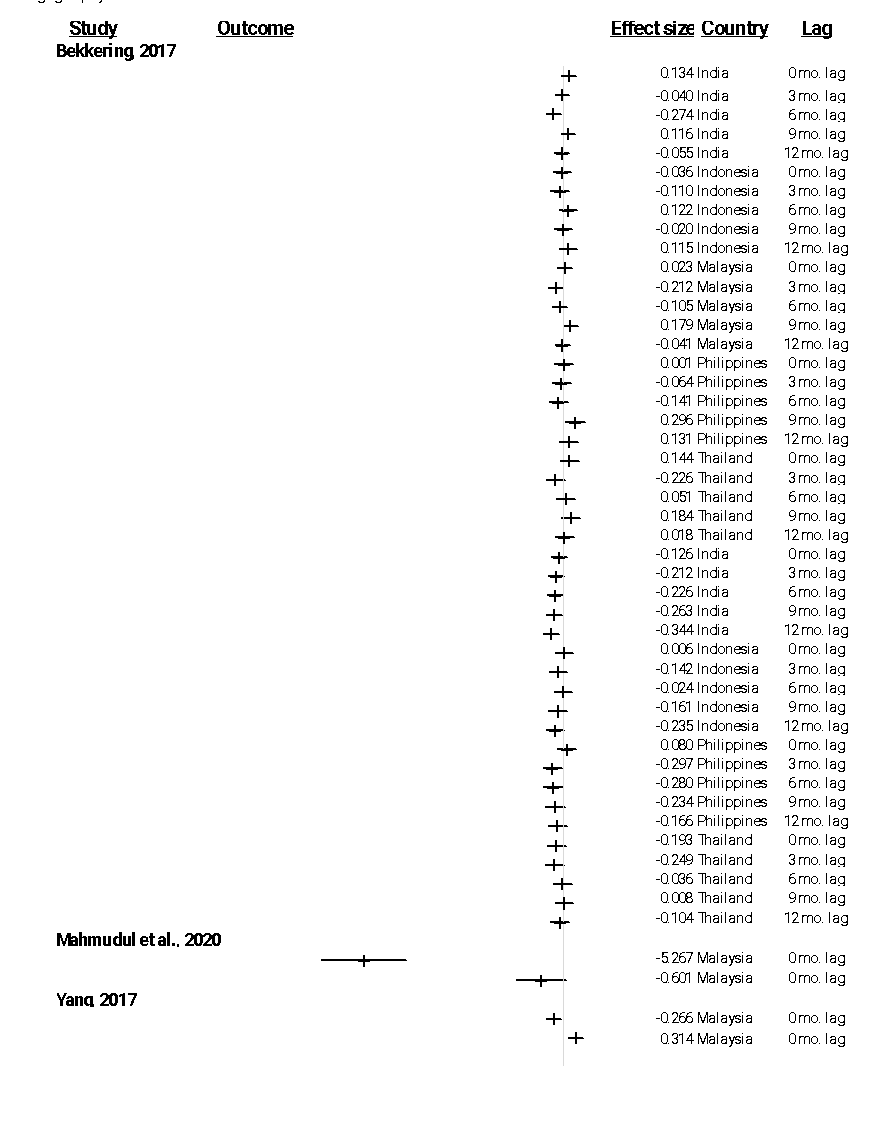


*Source: 3ie (2025)*

*Note: * indicates some concerns and low-risk of bias. Not enough degrees of freedom to estimate a pooled effect.*

**Figure A.7.2.3. Effects of El Niño on consumption and income**


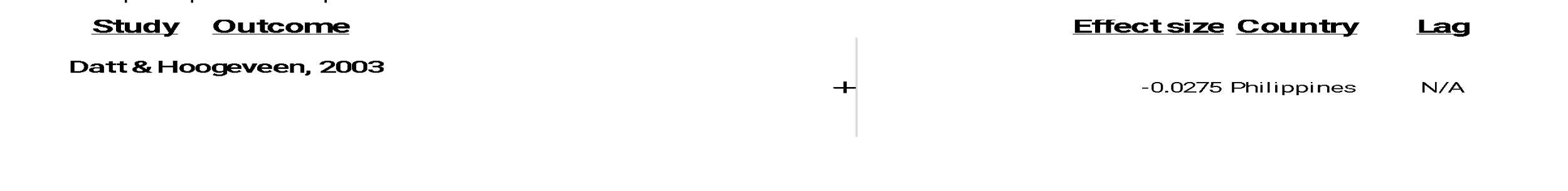


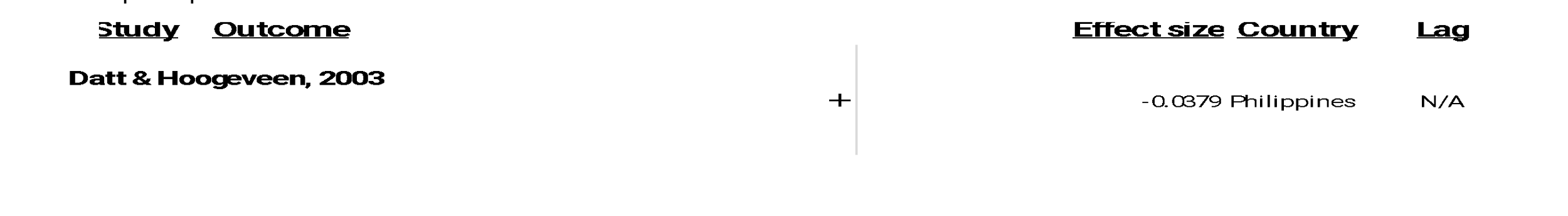


*Source: 3ie (2025)*

*Note: * indicates some concerns and low-risk of bias. Not enough degrees of freedom to estimate a pooled effect.*

**Figure A.7.2.4. Effects of El Niño on trade**


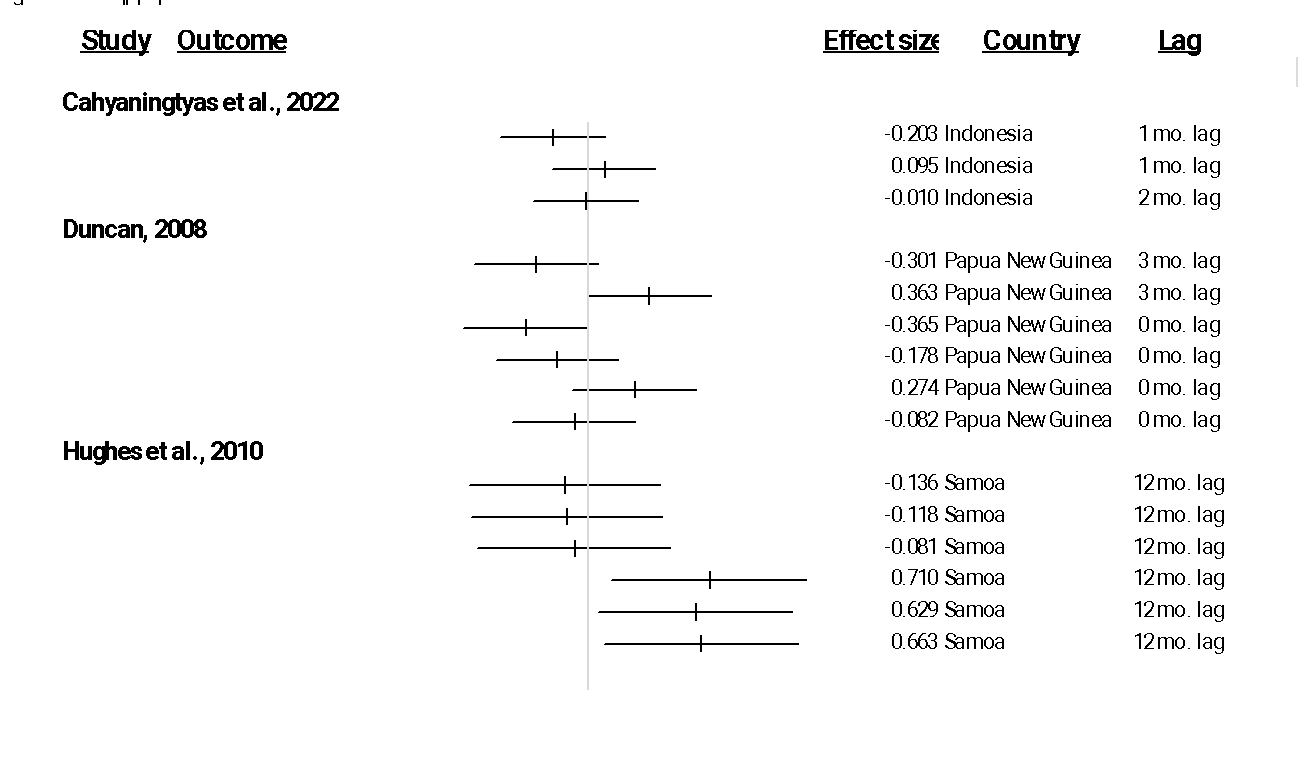


*Source: 3ie (2025)*

*Note: * indicates some concerns and low-risk of bias. Not enough degrees of freedom to estimate a pooled effect.*

**Effects of El Niño on Health Outcomes**

**Figure A.7.2.5. Effects of El Niño on enteric infections (other than cholera)**


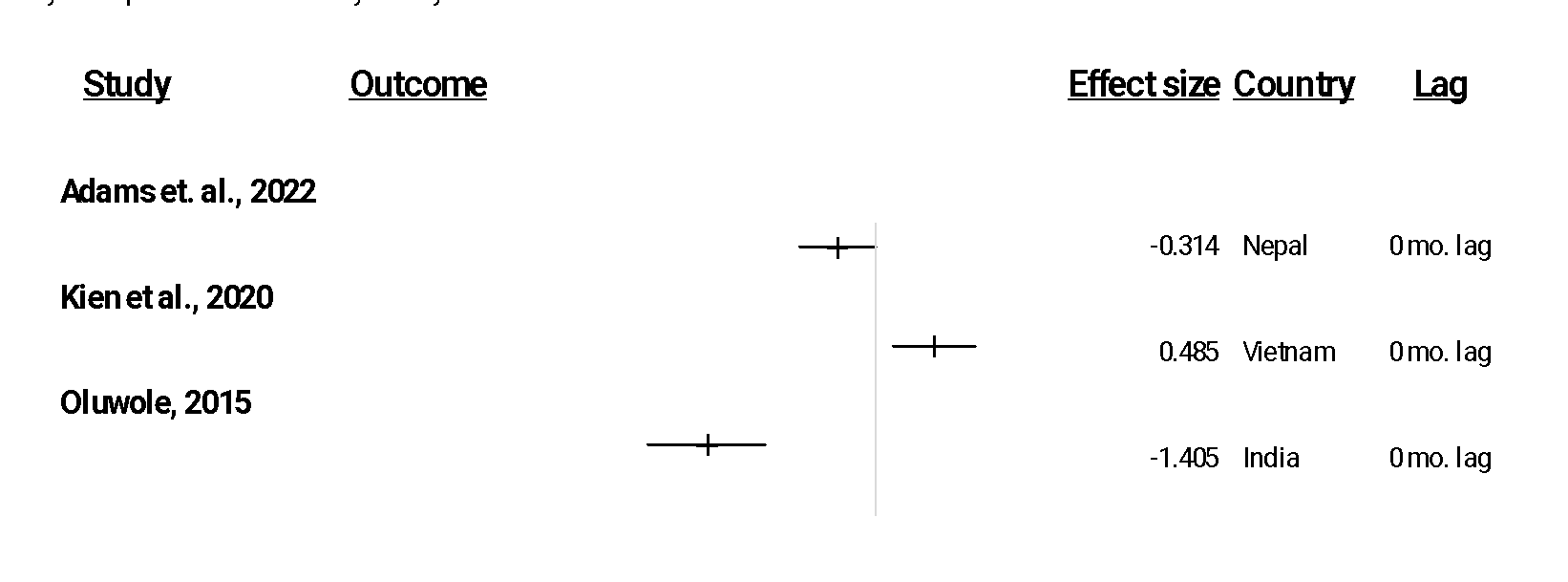


*Source: 3ie (2025)*

*Note: * indicates some concerns and low-risk of bias. Not enough degrees of freedom to estimate a pooled effect. One study using negative binomial modelling to estimate the effect of El Niño on enteric infections incidence was not included in the forest plot because of not reporting all the information needed to estimate the SMDs (Iyer et al. 2021).*

**Figure A.7.2.6. Effects of El Niño on direct injuries or fatalities**


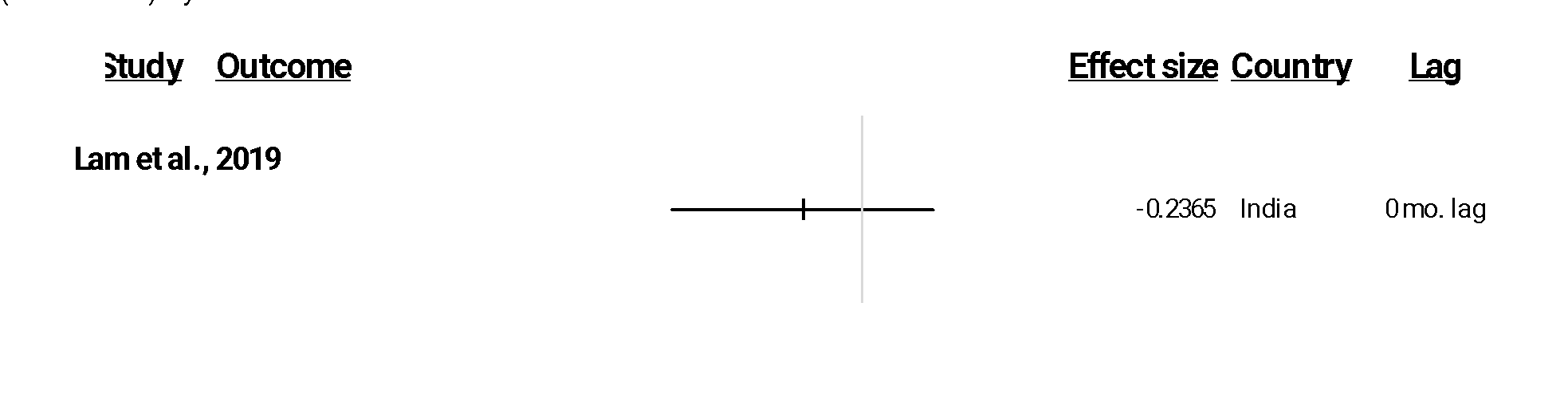


*Source: 3ie (2025)*

*Note: * indicates some concerns and low-risk of bias. Not enough degrees of freedom to estimate a pooled effect.*

**Figure A.7.2.7. Effects of El Niño on cholera**


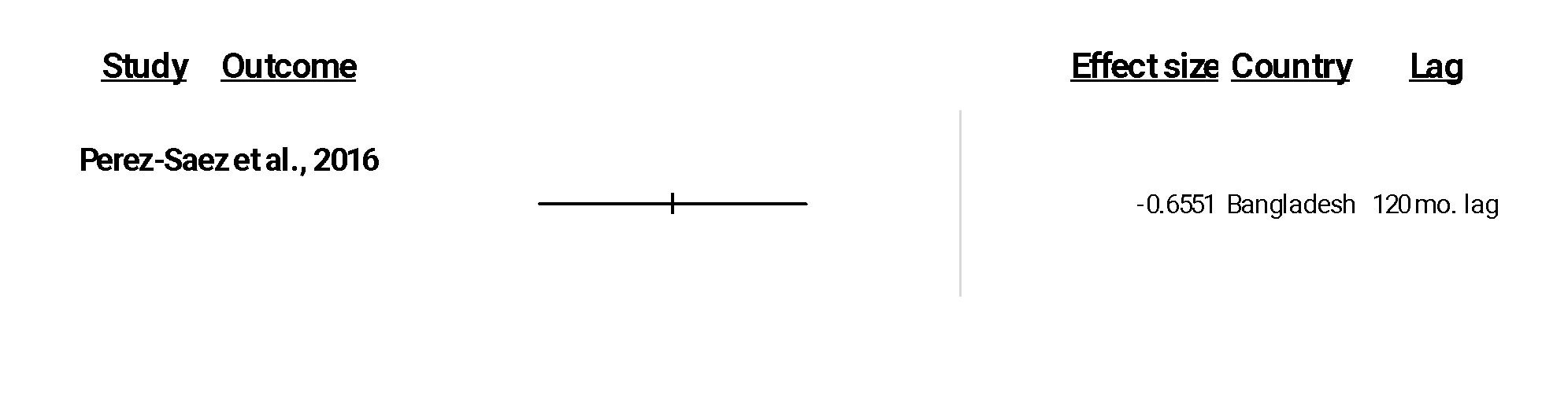


*Source: 3ie (2025)*

*Note: * indicates some concerns and low-risk of bias. Not enough degrees of freedom to estimate a pooled effect.*

**Figure A.7.2.8. Effects of El Niño on respiratory ailments**


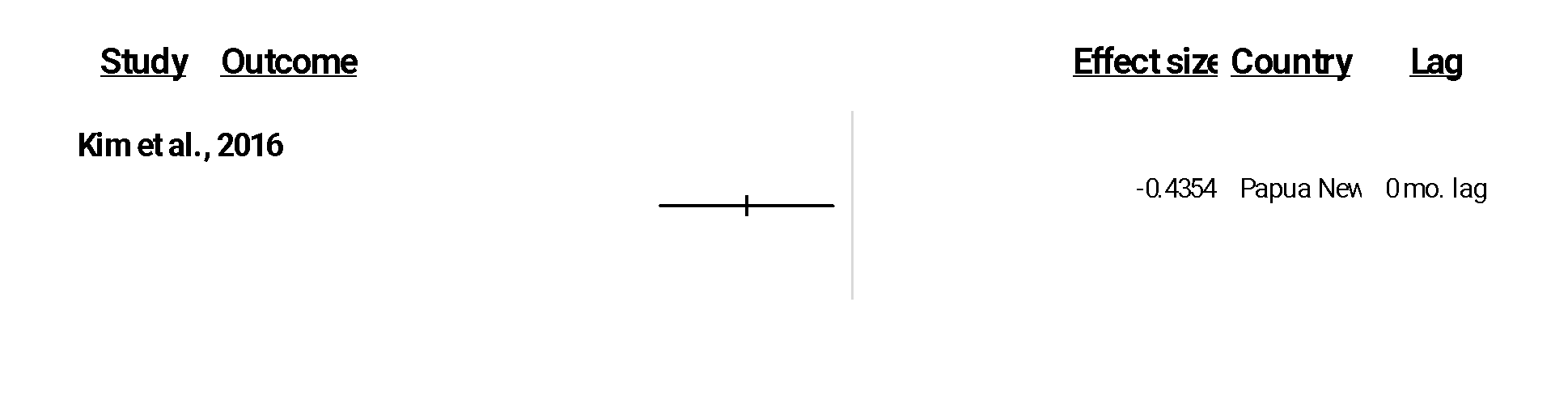


*Source: 3ie (2025)*

*Note: * indicates some concerns and low-risk of bias. Not enough degrees of freedom to estimate a pooled effect.*

**Figure A.7.2.9. Effects of +IOD on production**


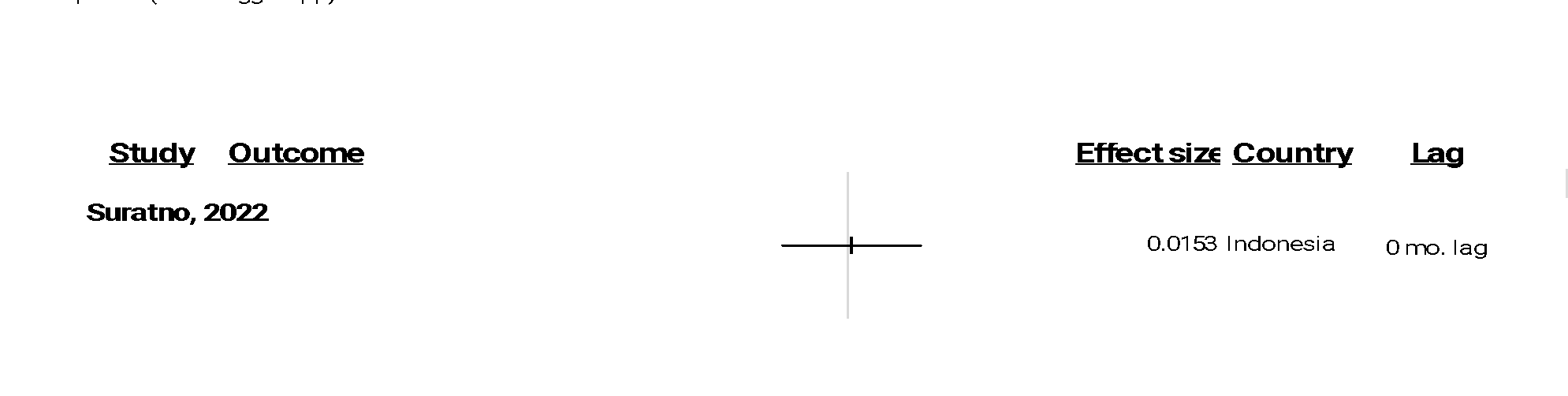


*Source: 3ie (2025)*

*Note: * indicates some concerns and low-risk of bias. Not enough degrees of freedom to estimate a pooled effect.*

**Figure A.7.2.10. Effects of +IOD on productivity**


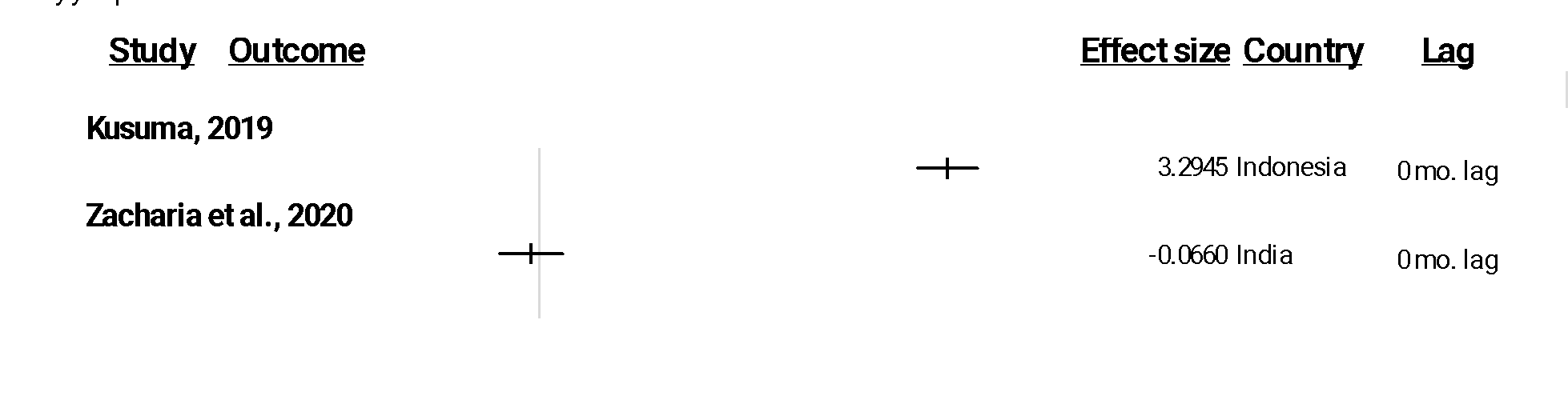


*Source: 3ie (2025)*

*Note: * indicates some concerns and low-risk of bias. Not enough degrees of freedom to estimate a pooled effect.*

**Figure A.7.2.11. Effects of +IOD on vector-borne diseases**


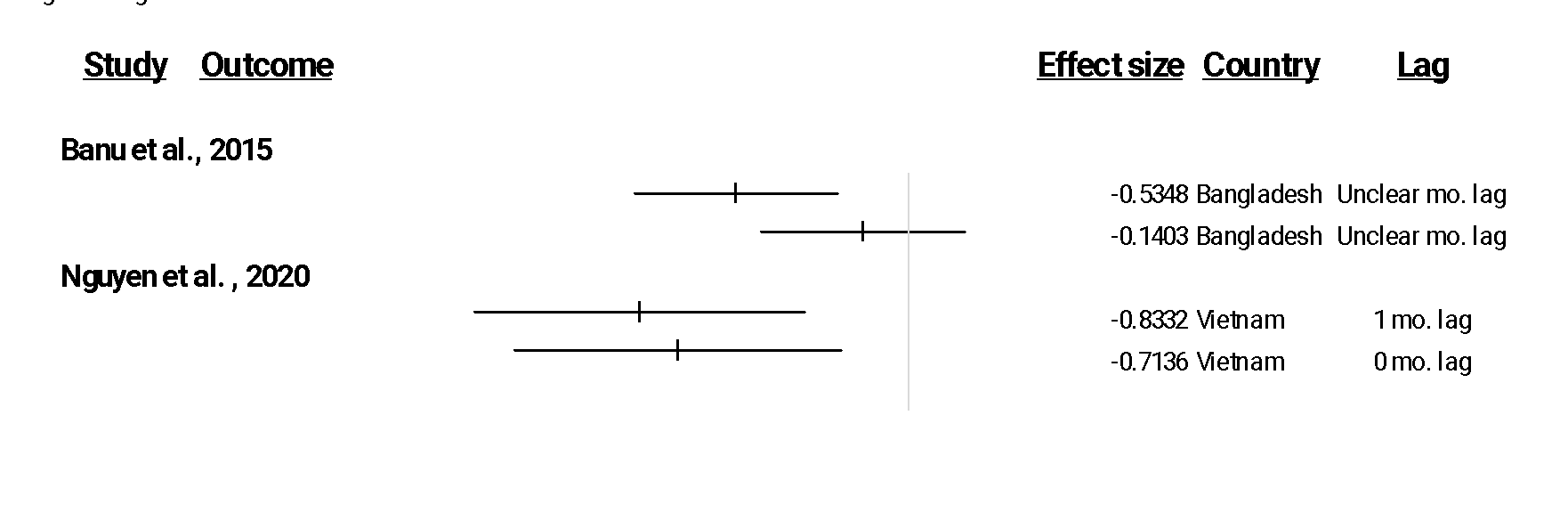


*Source: 3ie (2025)*

*Note: * indicates some concerns and low-risk of bias. Not enough degrees of freedom to estimate a pooled effect.*

**Figure A.7.2.12. Effects of +IOD on respiratory ailments**


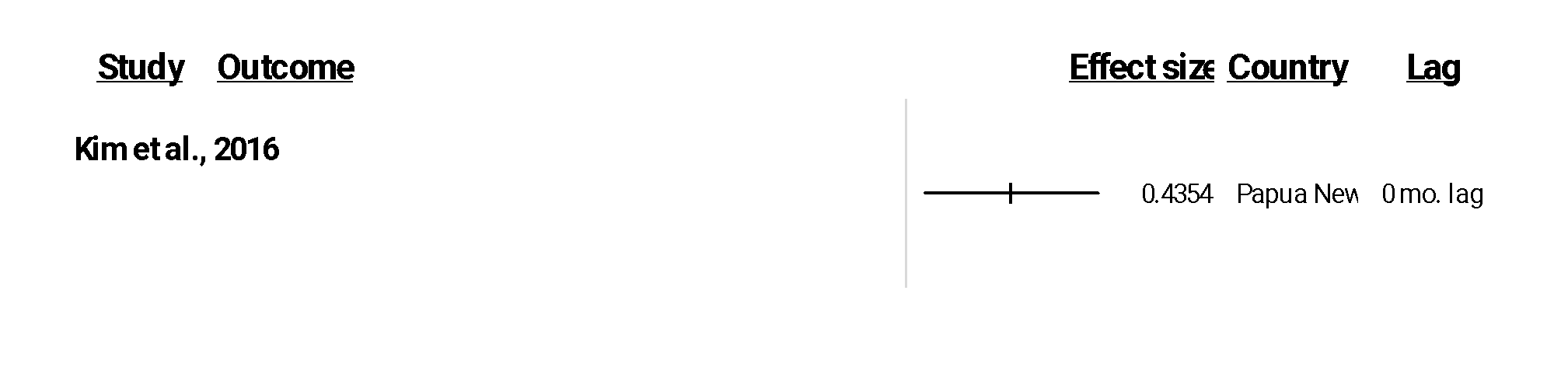


*Source: 3ie (2025)*

*Note: * indicates some concerns and low-risk of bias. Not enough degrees of freedom to estimate a pooled effect.*

**Figure A.7.2.13. Effects of +IOD on cholera**


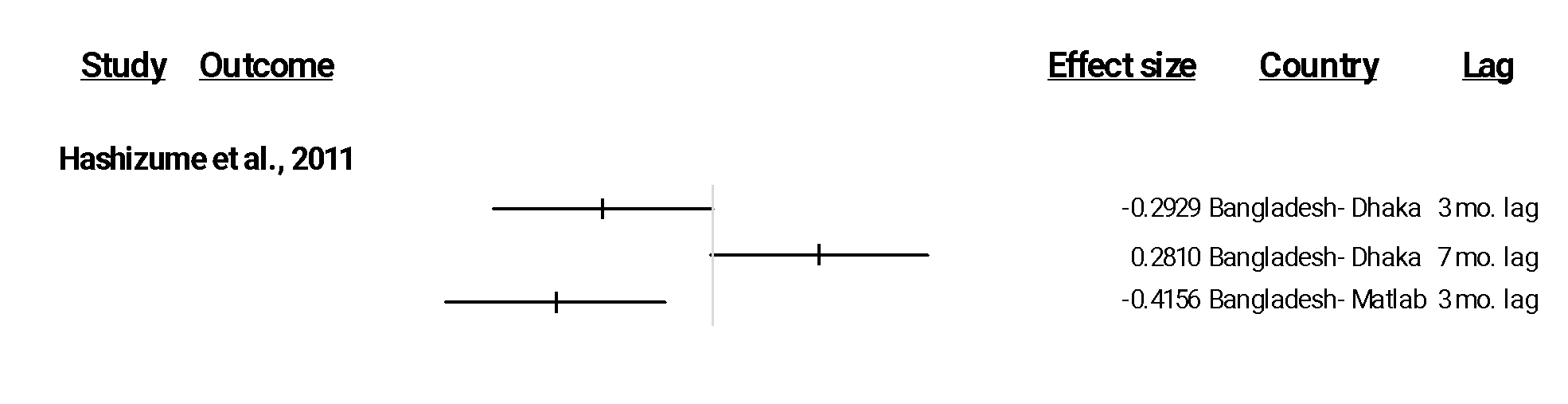


*Source: 3ie (2025)*

*Note: * indicates some concerns and low-risk of bias. Not enough degrees of freedom to estimate a pooled effect.*

## Appendix H Criteria determining selection of effect estimates for data extraction

Multiple climate driver metrics used: for studies displaying more than one estimate, we extracted one estimate per metric. Nino 4 and Nino 3.4 were prioritized over Nino 3 and Nino 1.2

For the following cases, we extracted all estimates available for:

- Studies displaying more than one estimate per country, region, province, city or any other unit of analysis;

- Studies reporting on more than one crop;

- Studies displaying results for multiple lags;

Estimates from the same study were then combined via 1) robust variance estimation meta-analysis; or 2) median values of correlation coefficients.

## Appendix I Distribution of correlation coefficients and standardized effect sizes by outcome

***Estimates for El Niño***

|  | **Correlation coefficients** | | | | |
| --- | --- | --- | --- | --- | --- |
|  | **Min** | **Median** | **Max** | **N. of studies** | **N. of estimates** |
| Aggregated production | -0.20 | -0.02 | 0.06 | 1 | 5 |
| Cholera | -0.54 | -0.23 | 0.84 | 3 | 5 |
| Direct injuries and fatalities | 0.00 | 0.00 | 0.00 | 0 | 0 |
| Enteric infections and diseases | -0.55 | 0.13 | 0.50 | 3 | 35 |
| Investments | 0.00 | 0.00 | 0.00 | 0 | 0 |
| Prices | -0.11 | 0.05 | 0.12 | 1 | 5 |
| Production | -0.77 | -0.20 | 0.56 | 11 | 143 |
| Productivity | -0.56 | 0.05 | 0.54 | 9 | 220 |
| Trade | 0.00 | 0.00 | 0.00 | 0 | 0 |
| Vector-borne diseases | -0.43 | 0.14 | 0.57 | 10 | 54 |
| Consumption and expenditures | 0.00 | 0.00 | 0.00 | 0 | 0 |
| Respiratory ailments | 0.00 | 0.00 | 0.00 | 0 | 0 |
| Total income and wealth | 0.00 | 0.00 | 0.00 | 0 | 0 |

|  | **SMD (Hedge’s g from regression coefficients)** | | | | |
| --- | --- | --- | --- | --- | --- |
|  | **Min** | **Median** | **Max** | **N. of studies** | **N. of estimates** |
| Aggregated production | -4.30 | -0.10 | 0.31 | 4 | 17 |
| Cholera | -0.66 | -0.66 | -0.66 | 1 | 1 |
| Direct injuries and fatalities | -0.24 | -0.24 | -0.24 | 1 | 1 |
| Enteric infections and diseases | -0.48 | -0.31 | -0.31 | 2 | 5 |
| Investments | -5.27 | -0.06 | 0.31 | 3 | 49 |
| Prices | -2.20 | 0.04 | 0.71 | 9 | 21 |
| Production | -1.36 | -0.22 | 0.80 | 13 | 84 |
| Productivity | -1.61 | -0.27 | 1.39 | 16 | 32 |
| Trade | -0.36 | -0.08 | 0.71 | 3 | 15 |
| Vector-borne diseases | -2.91 | -0.46 | 0.95 | 11 | 21 |
| Consumption and expenditures | -0.03 | -0.03 | -0.03 | 1 | 1 |
| Respiratory ailments | -0.44 | 1.22 | 1.31 | 1 | 7 |
| Total income and wealth | -0.04 | -0.04 | -0.04 | 1 | 1 |

***Estimates for +IOD***

|  | **Correlation coefficients** | | | | |
| --- | --- | --- | --- | --- | --- |
|  | **Min** | **Median** | **Max** | **N. of studies** | **N. of estimates** |
| Aggregated production | 0.00 | 0.00 | 0.00 | 0 | 0 |
| Cholera | 0.00 | 0.00 | 0.00 | 0 | 0 |
| Direct injuries and fatalities | 0.00 | 0.00 | 0.00 | 0 | 0 |
| Enteric infections | -0.24 | -0.24 | -0.24 | 1 | 2 |
| Investments | 0.00 | 0.00 | 0.00 | 0 | 0 |
| Prices | 0.00 | 0.00 | 0.00 | 0 | 0 |
| Production | -0.80 | 0.01 | 0.69 | 2 | 46 |
| Productivity | -0.68 | 0.28 | 0.76 | 1 | 12 |
| Trade | 0.00 | 0.00 | 0.00 | 0 | 0 |
| Vector-borne diseases | -0.24 | 0.36 | 0.46 | 5 | 5 |
| Consumption and expenditures | 0.00 | 0.00 | 0.00 | 0 | 0 |
| Respiratory ailments | 0.00 | 0.00 | 0.00 | 0 | 0 |
| Total income and wealth | 0.00 | 0.00 | 0.00 | 0 | 0 |

|  | **SMD (Hedge’s g from regression coefficients)** | | | | |
| --- | --- | --- | --- | --- | --- |
|  | **Min** | **Median** | **Max** | **N. of studies** | **N. of estimates** |
| Aggregated production | 0.00 | 0.00 | 0.00 | 0 | 0 |
| Cholera | -0.42 | -0.29 | 0.28 | 1 | 3 |
| Direct injuries and fatalities | 0.00 | 0.00 | 0.00 | 0 | 0 |
| Enteric infections | 0.00 | 0.00 | 0.00 | 0 | 0 |
| Investments | 0.00 | 0.00 | 0.00 | 0 | 0 |
| Prices | 0.00 | 0.00 | 0.00 | 0 | 0 |
| Production | 0.00 | 0.00 | 0.00 | 0 | 0 |
| Productivity | -0.07 | 1.61 | 3.29 | 2 | 2 |
| Trade | 0.00 | 0.00 | 0.00 | 0 | 0 |
| Vector-borne diseases | -0.83 | -0.62 | -0.14 | 2 | 4 |
| Consumption and expenditures | 0.00 | 0.00 | 0.00 | 0 | 0 |
| Respiratory ailments | -1.07 | -0.70 | 0.44 | 1 | 7 |
| Total income and wealth | 0.00 | 0.00 | 0.00 | 0 | 0 |

## Appendix J Distribution of correlation coefficients and standardized effect sizes by region and outcome

***Estimates for El Niño***

|  | **Correlation coefficients** | | | | |
| --- | --- | --- | --- | --- | --- |
|  | **Min** | **Median** | **Max** | **N. of studies** | **N. of estimates** |
| **Indian Subcontinent and Ocean** |  |  |  |  |  |
| Aggregated production | -0.15 | -0.15 | -0.15 | 1 | 1 |
| Enteric infections and diseases | -0.55 | 0.13 | 0.19 | 2 | 9 |
| Prices | -0.06 | -0.06 | -0.06 | 1 | 1 |
| Production | -0.77 | -0.19 | 0.56 | 7 | 137 |
| Productivity | -0.56 | 0.05 | 0.48 | 5 | 124 |
| Vector-borne diseases | -0.43 | 0.07 | 0.55 | 4 | 28 |
| Cholera | -0.54 | -0.23 | 0.84 | 3 | 5 |
| **South East Asia** |  |  |  |  |  |
| Aggregated production | -0.20 | 0.00 | 0.06 | 1 | 4 |
| Prices | -0.11 | 0.06 | 0.12 | 1 | 4 |
| Production | -0.62 | -0.29 | 0.45 | 4 | 6 |
| Productivity | -0.53 | 0.05 | 0.54 | 4 | 96 |
| Vector-borne diseases | -0.36 | 0.02 | 0.35 | 6 | 13 |
| **India** |  |  |  |  |  |
| Aggregated production | -0.15 | -0.15 | -0.15 | 1 | 1 |
| Prices | -0.11 | -0.11 | -0.11 | 1 | 1 |
| Production | -0.77 | -0.20 | 0.56 | 6 | 133 |
| Productivity | -0.56 | 0.05 | 0.48 | 5 | 124 |
| Vector-borne diseases | -0.43 | 0.18 | 0.55 | 3 | 17 |
| **Indonesia** |  |  |  |  |  |
| Aggregated production | -0.02 | -0.02 | -0.02 | 1 | 1 |
| Prices | -0.06 | -0.06 | -0.06 | 1 | 1 |
| Production | -0.50 | -0.50 | -0.50 | 1 | 1 |
| Productivity | -0.53 | 0.06 | 0.54 | 2 | 93 |
| Vector-borne diseases | -0.03 | 0.14 | 0.33 | 4 | 5 |
| **Philippines** |  |  |  |  |  |
| Aggregated production | -0.20 | -0.20 | -0.20 | 1 | 1 |
| Prices | 0.12 | 0.12 | 0.12 | 1 | 1 |
| Production | -0.20 | -0.20 | -0.20 | 1 | 2 |
| Productivity | -0.24 | -0.11 | 0.12 | 2 | 3 |
| Vector-borne diseases | 0.12 | 0.12 | 0.12 | 1 | 1 |
| **Malaysia** |  |  |  |  |  |
| Aggregated production | 0.06 | 0.06 | 0.06 | 1 | 1 |
| Prices | 0.07 | 0.07 | 0.07 | 1 | 1 |
| Production | -0.62 | -0.09 | 0.45 | 1 | 2 |
| Vector-borne diseases | -0.26 | -0.26 | -0.26 | 1 | 1 |
| **Bangladesh** |  |  |  |  |  |
| Cholera | -0.54 | -0.23 | 0.84 | 3 | 5 |
| Enteric infections and diseases | -0.55 | -0.38 | -0.21 | 1 | 2 |
| Vector-borne diseases | -0.22 | -0.07 | 0.01 | 2 | 3 |
| **Lower Mekong Region** |  |  |  |  |  |
| Aggregated production | 0.01 | 0.01 | 0.01 | 1 | 1 |
| Prices | 0.05 | 0.05 | 0.05 | 1 | 1 |
| Production | -0.38 | -0.38 | -0.38 | 1 | 1 |
| Vector-borne diseases | -0.36 | -0.18 | 0.01 | 1 | 2 |
| **Oceania** |  |  |  |  |  |
| Enteric infections and diseases | -0.55 | 0.35 | 0.50 | 1 | 26 |
| Vector-borne diseases | -0.18 | 0.34 | 0.57 | 1 | 13 |

|  | **SMD (Hedge’s g from regression coefficients)** | | | | |
| --- | --- | --- | --- | --- | --- |
|  | **Min** | **Median** | **Max** | **N. of studies** | **N. of estimates** |
| **Indian Subcontinent and Ocean** |  |  |  |  |  |
| Aggregated production | -0.63 | -0.49 | -0.09 | 2 | 3 |
| Direct injuries and fatalities | -0.24 | -0.24 | -0.24 | 1 | 1 |
| Enteric infections and diseases | -0.31 | -0.31 | -0.31 | 1 | 4 |
| Investments | -0.34 | -0.17 | 0.13 | 1 | 10 |
| Prices | 0.09 | 0.15 | 0.22 | 1 | 2 |
| Production | -0.68 | -0.14 | 0.14 | 1 | 9 |
| Productivity | -1.21 | -0.37 | 1.39 | 5 | 11 |
| Vector-borne diseases | -0.61 | -0.46 | 0.21 | 3 | 10 |
| Cholera | -0.66 | -0.66 | -0.66 | 1 | 1 |
| **South East Asia** |  |  |  |  |  |
| Aggregated production | -4.30 | -0.09 | 0.31 | 4 | 14 |
| Enteric infections and diseases | -0.48 | -0.48 | -0.48 | 1 | 1 |
| Investments | -5.27 | -0.04 | 0.31 | 3 | 39 |
| Prices | -2.20 | 0.01 | 0.71 | 8 | 19 |
| Production | -1.36 | -0.22 | 0.80 | 12 | 66 |
| Productivity | -1.61 | -0.27 | -0.05 | 7 | 18 |
| Trade | -0.20 | -0.01 | 0.10 | 1 | 3 |
| Vector-borne diseases | -2.91 | -0.02 | 0.95 | 7 | 11 |
| **India** |  |  |  |  |  |
| Aggregated production | -0.63 | -0.49 | -0.09 | 2 | 3 |
| Direct injuries and fatalities | -0.24 | -0.24 | -0.24 | 1 | 1 |
| Investments | -0.34 | -0.17 | 0.13 | 1 | 10 |
| Prices | 0.21 | 0.26 | 0.32 | 1 | 2 |
| Production | -0.14 | 0.14 | 0.14 | 1 | 3 |
| Productivity | -0.63 | 0.31 | 1.39 | 4 | 4 |
| **Indonesia** |  |  |  |  |  |
| Aggregated production | -4.30 | -0.20 | -0.09 | 3 | 4 |
| Investments | -0.24 | -0.03 | 0.12 | 1 | 10 |
| Prices | -1.10 | -0.01 | 0.71 | 5 | 9 |
| Production | -1.36 | -0.25 | 0.14 | 3 | 8 |
| Productivity | -0.45 | -0.38 | -0.30 | 2 | 2 |
| Trade | -0.20 | -0.01 | 0.10 | 1 | 3 |
| Vector-borne diseases | 0.50 | 0.50 | 0.50 | 1 | 1 |
| **Philippines** |  |  |  |  |  |
| Aggregated production | -0.41 | -0.32 | 0.09 | 3 | 4 |
| Consumption and expenditures | -0.03 | -0.03 | -0.03 | 1 | 1 |
| Investments | -0.30 | -0.10 | 0.30 | 1 | 10 |
| Prices | -2.20 | 0.11 | 0.41 | 3 | 4 |
| Production | -1.11 | -0.22 | 0.80 | 5 | 34 |
| Productivity | -0.50 | -0.19 | -0.13 | 1 | 3 |
| Total income and wealth | -0.04 | -0.04 | -0.04 | 1 | 1 |
| **Malaysia** |  |  |  |  |  |
| Aggregated production | -0.11 | 0.00 | 0.09 | 2 | 3 |
| Investments | -5.27 | -0.11 | 0.31 | 3 | 9 |
| Prices | -0.21 | 0.04 | 0.18 | 2 | 3 |
| Production | -0.21 | -0.04 | 0.14 | 2 | 2 |
| Productivity | -1.21 | -0.27 | 0.85 | 3 | 17 |
| Vector-borne diseases | -2.91 | -0.02 | 0.30 | 3 | 3 |
| **Bangladesh** |  |  |  |  |  |
| Cholera | -0.66 | -0.66 | -0.66 | 1 | 1 |
| Production | -0.68 | -0.41 | -0.14 | 1 | 2 |
| Vector-borne diseases | -0.50 | -0.21 | 0.21 | 2 | 4 |
| **Lower Mekong Region** |  |  |  |  |  |
| Aggregated production | -0.07 | -0.02 | 0.31 | 2 | 3 |
| Enteric infections and diseases | -0.48 | -0.48 | -0.48 | 1 | 1 |
| Investments | -0.25 | -0.01 | 0.18 | 1 | 10 |
| Prices | -0.09 | 0.01 | 0.05 | 2 | 3 |
| Production | -0.86 | -0.14 | 0.14 | 4 | 9 |
| Productivity | -1.61 | -0.64 | -0.05 | 3 | 3 |
| Vector-borne diseases | -0.76 | -0.46 | 0.95 | 3 | 7 |
| **Oceania** |  |  |  |  |  |
| Production | -0.90 | -0.43 | -0.21 | 1 | 9 |
| Productivity | -0.95 | -0.50 | -0.14 | 1 | 3 |
| Respiratory ailments | -0.44 | 1.22 | 1.31 | 1 | 7 |
| Trade | -0.36 | -0.08 | 0.71 | 2 | 12 |

## Appendix K Risk of bias of included studies

#### **K.1 Studies using regression analysis**

**Figure A. 11. 1. Summary risk of bias assessment for regression studies**

*Source: 3ie (2025)*

**Appendix Table 18. Risk of bias assessment for regression studies**


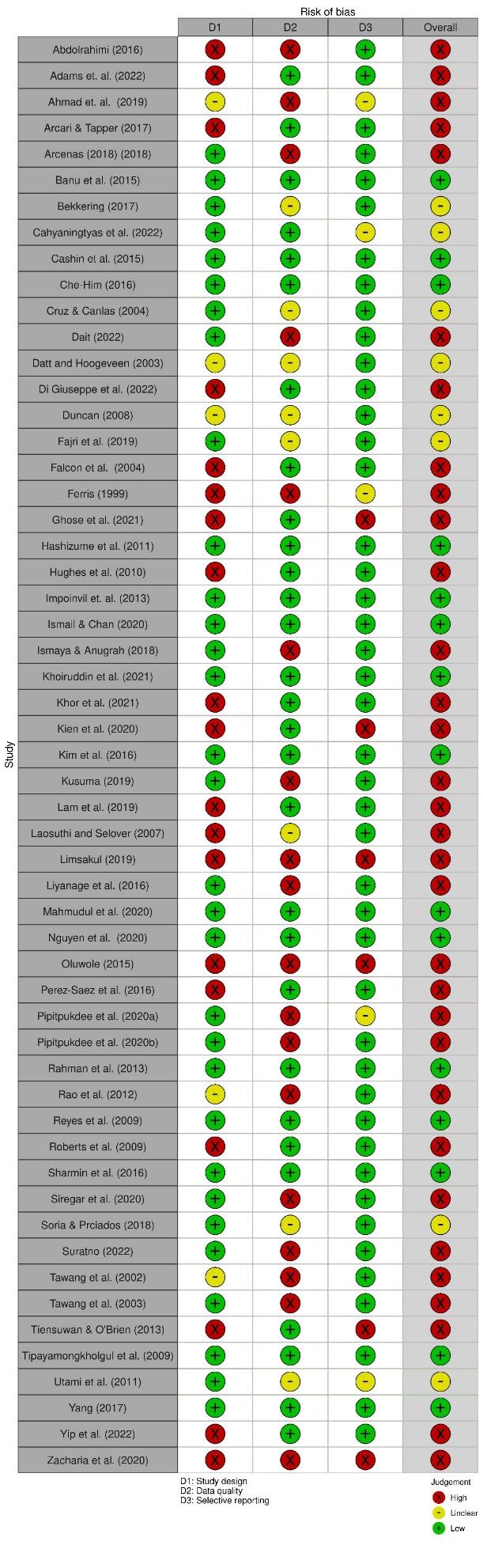

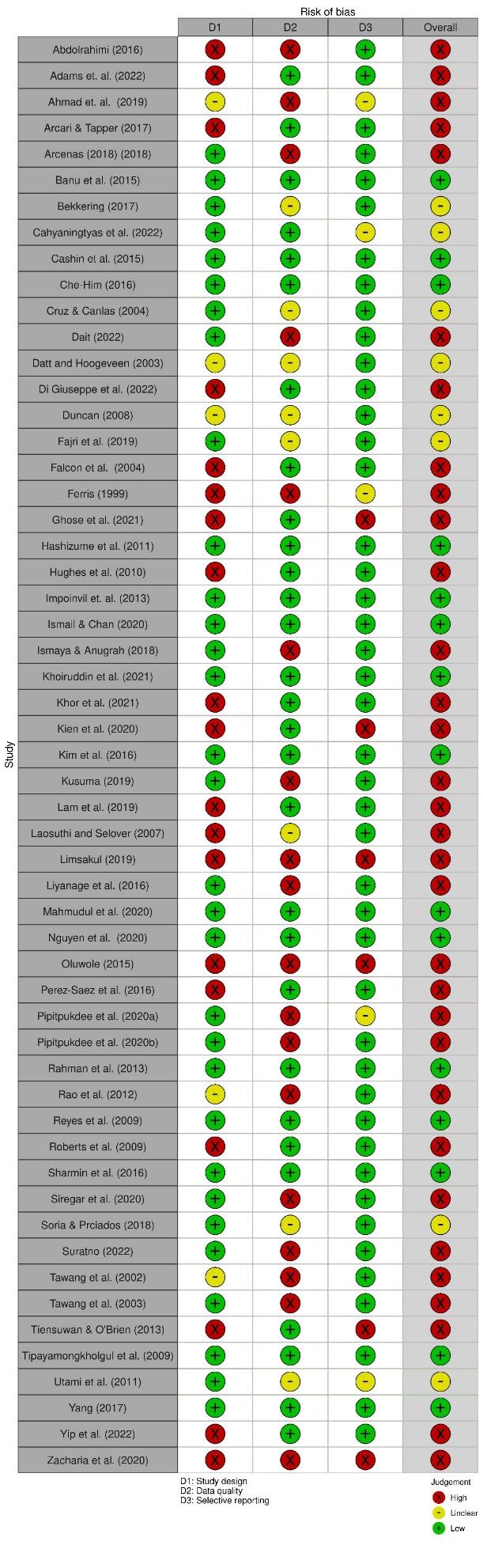


Appendix Table 18. continues…


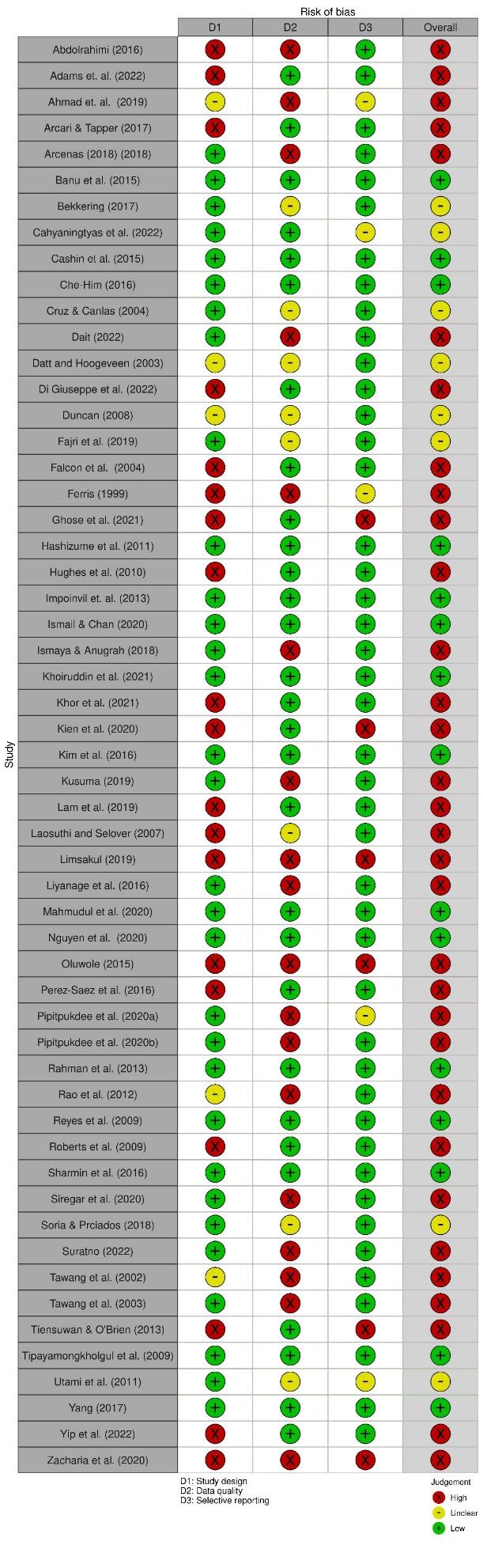

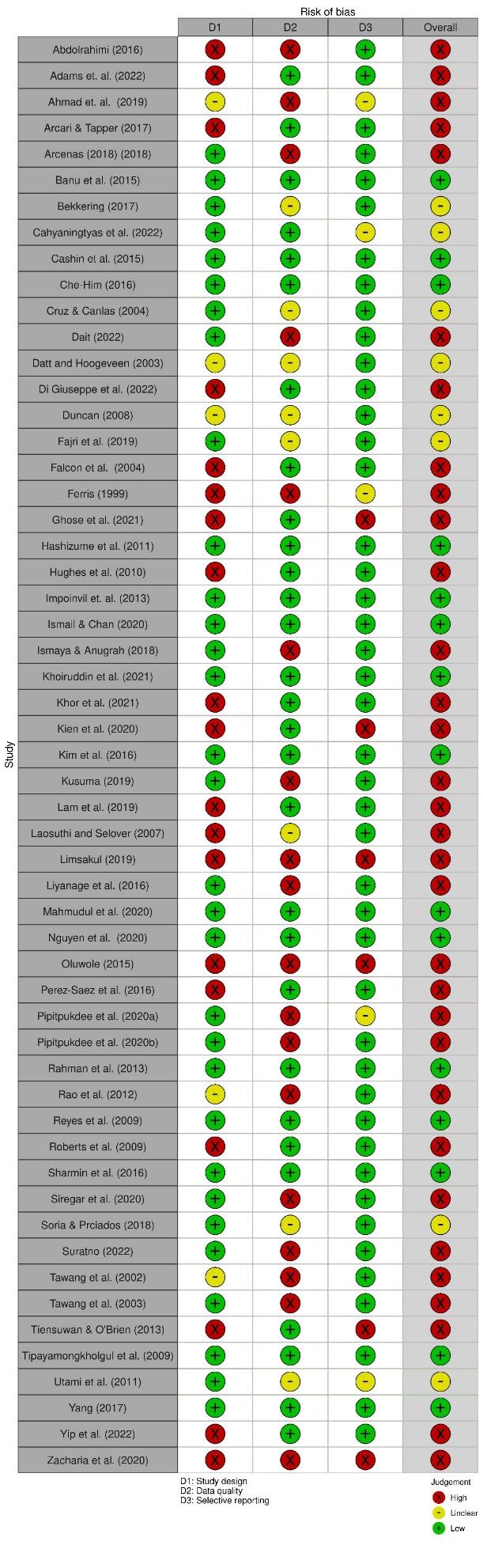

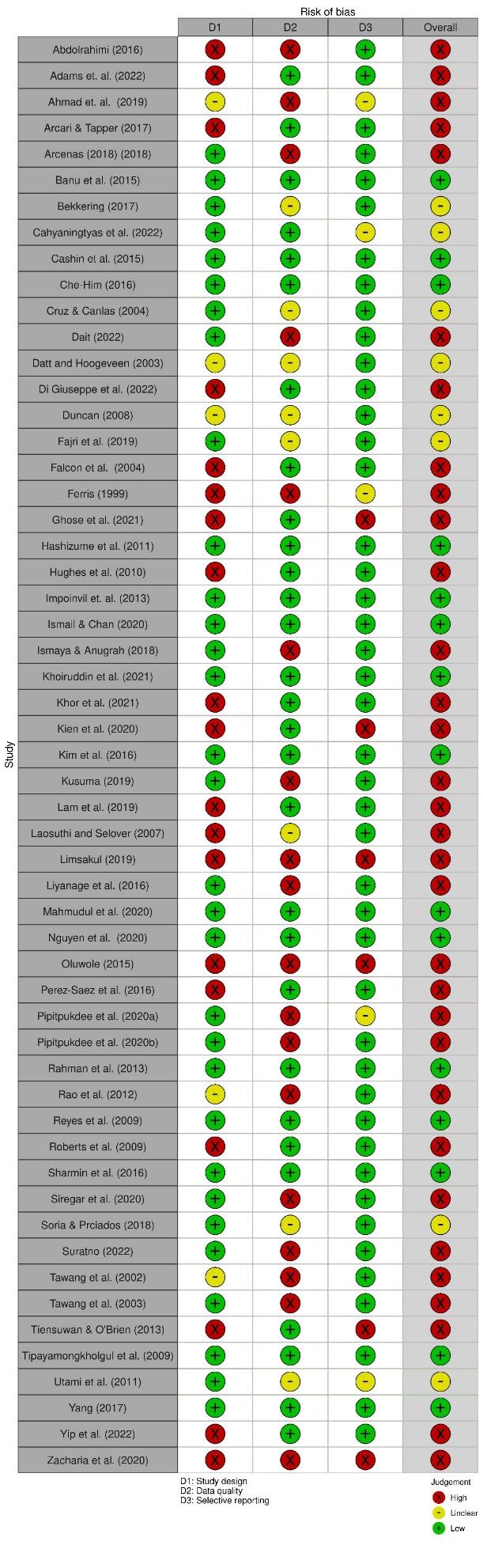


Appendix Table 18. continues…


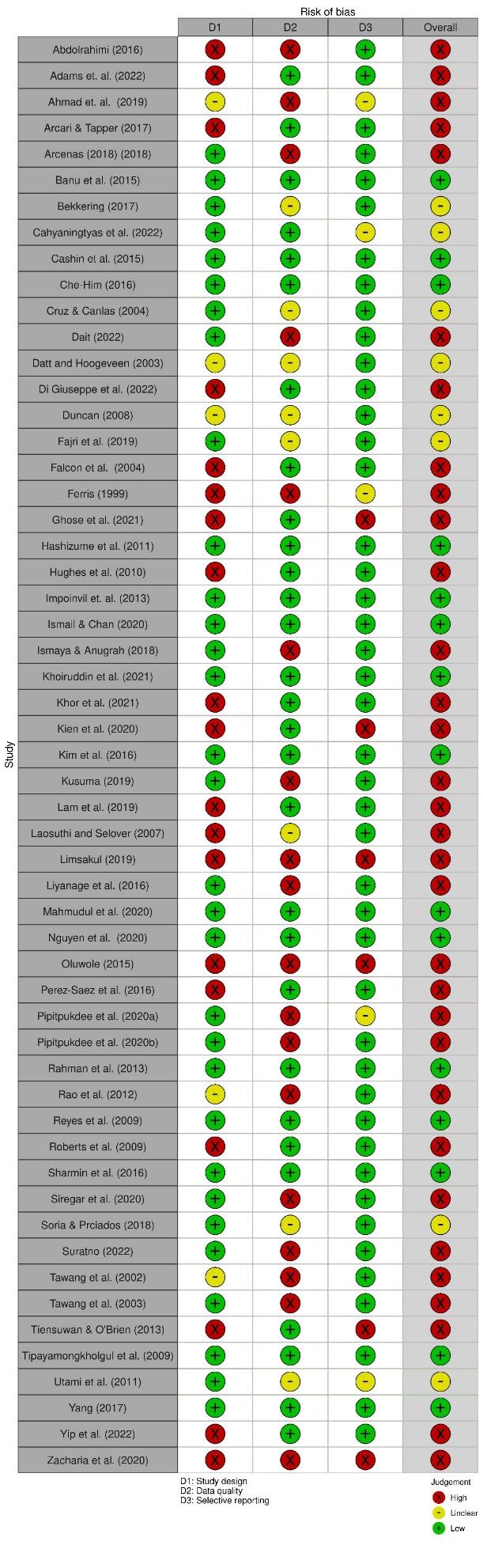

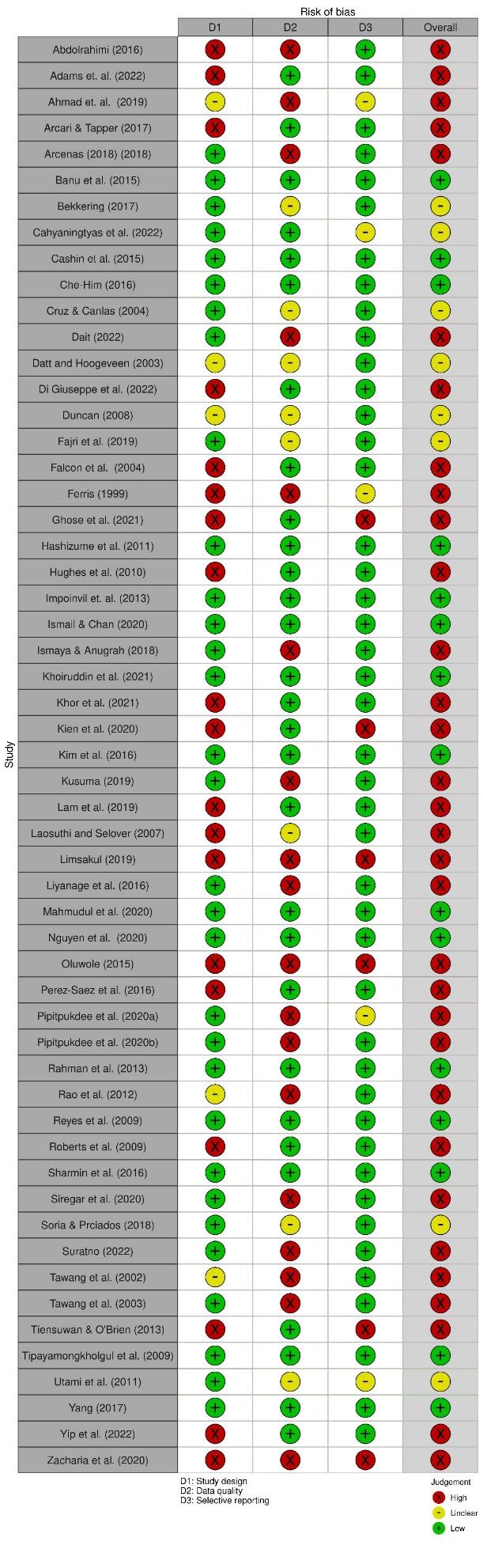


#### **K.2 Correlational studies**

**Figure A. 11. 2. Summary risk of bias assessment for correlation studies**

*Source: 3ie (2025)*

**Appendix Table 19. Risk of bias assessment for correlation studies**


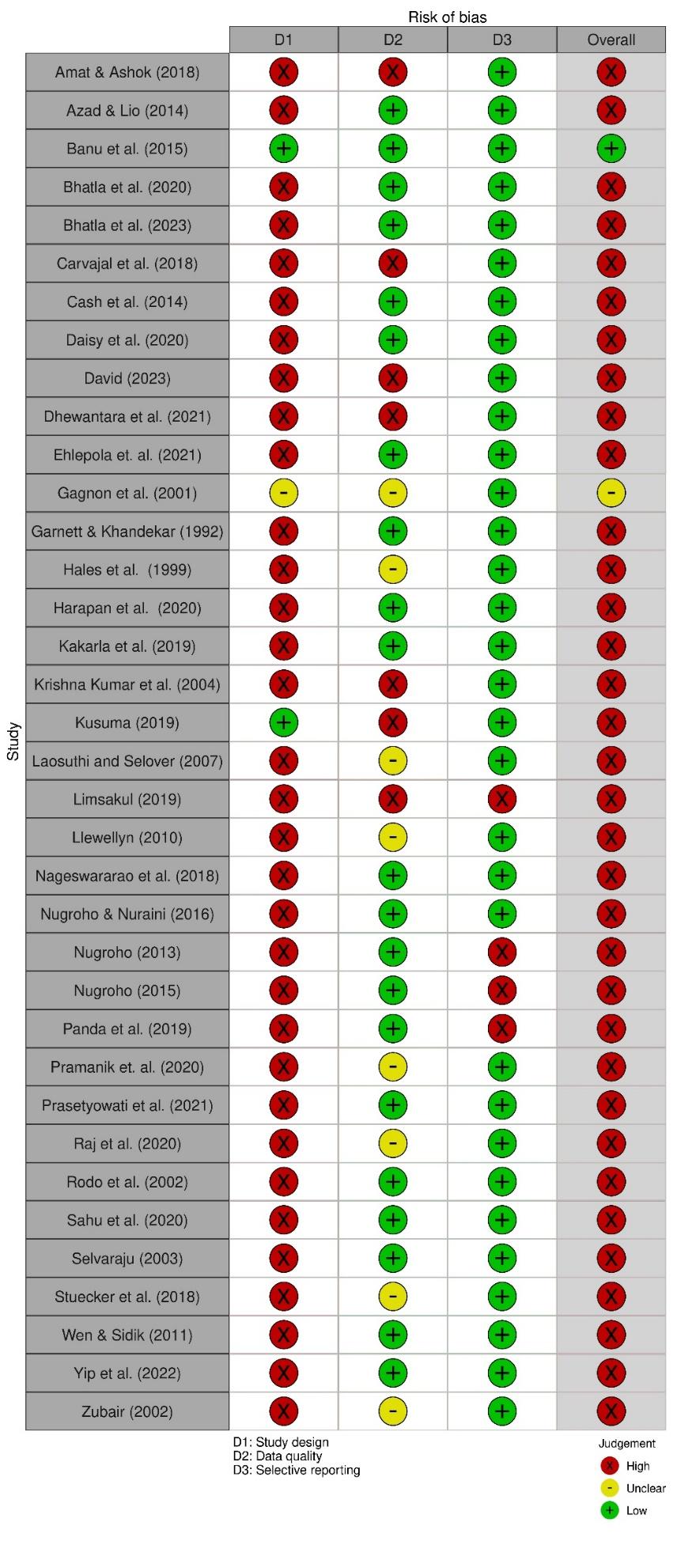


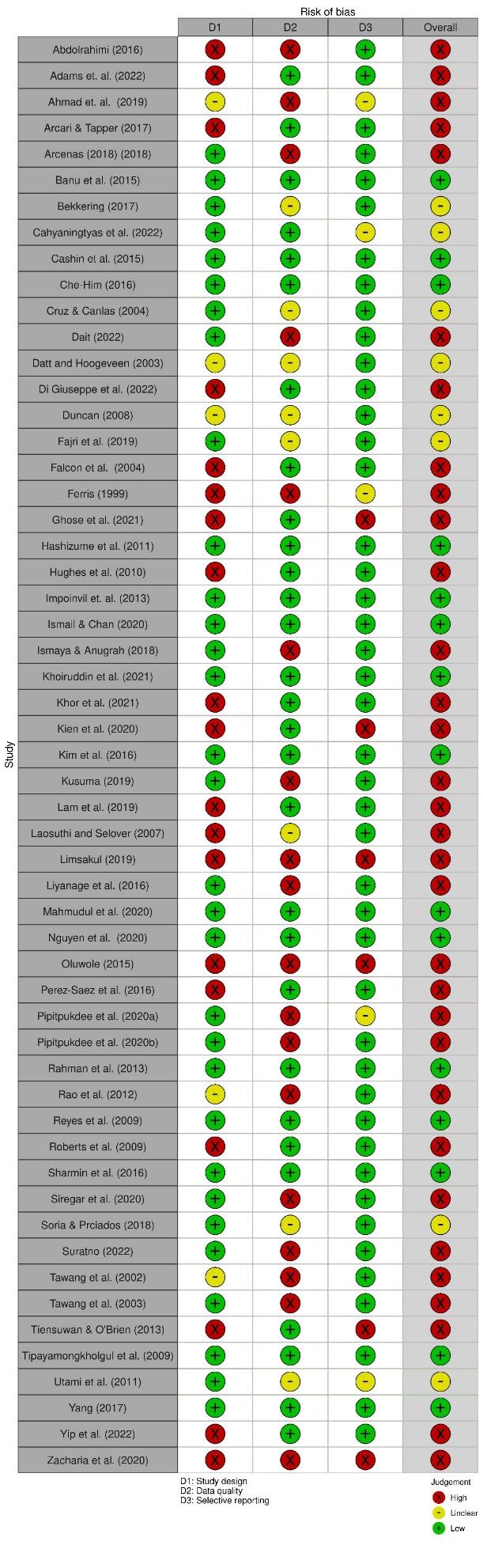


Appendix Table 19. *continues*…


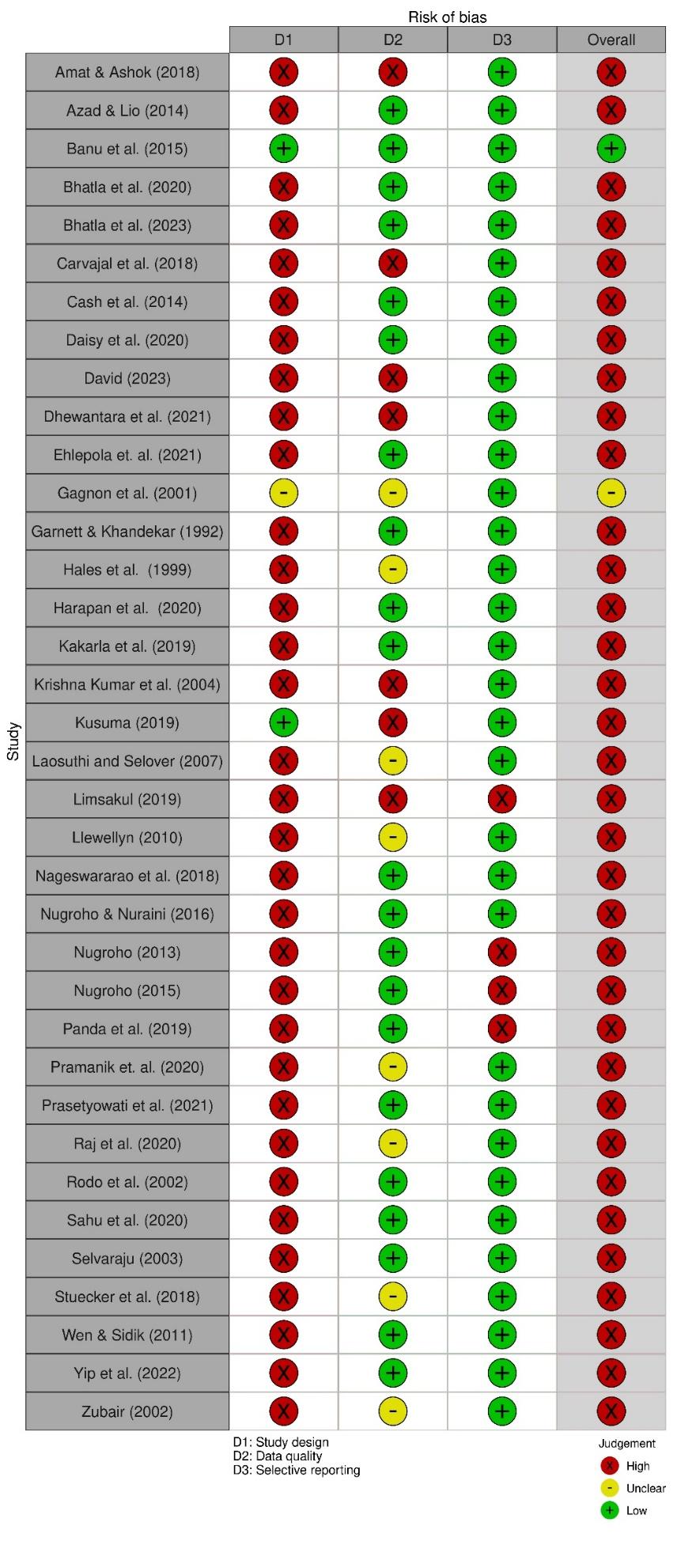

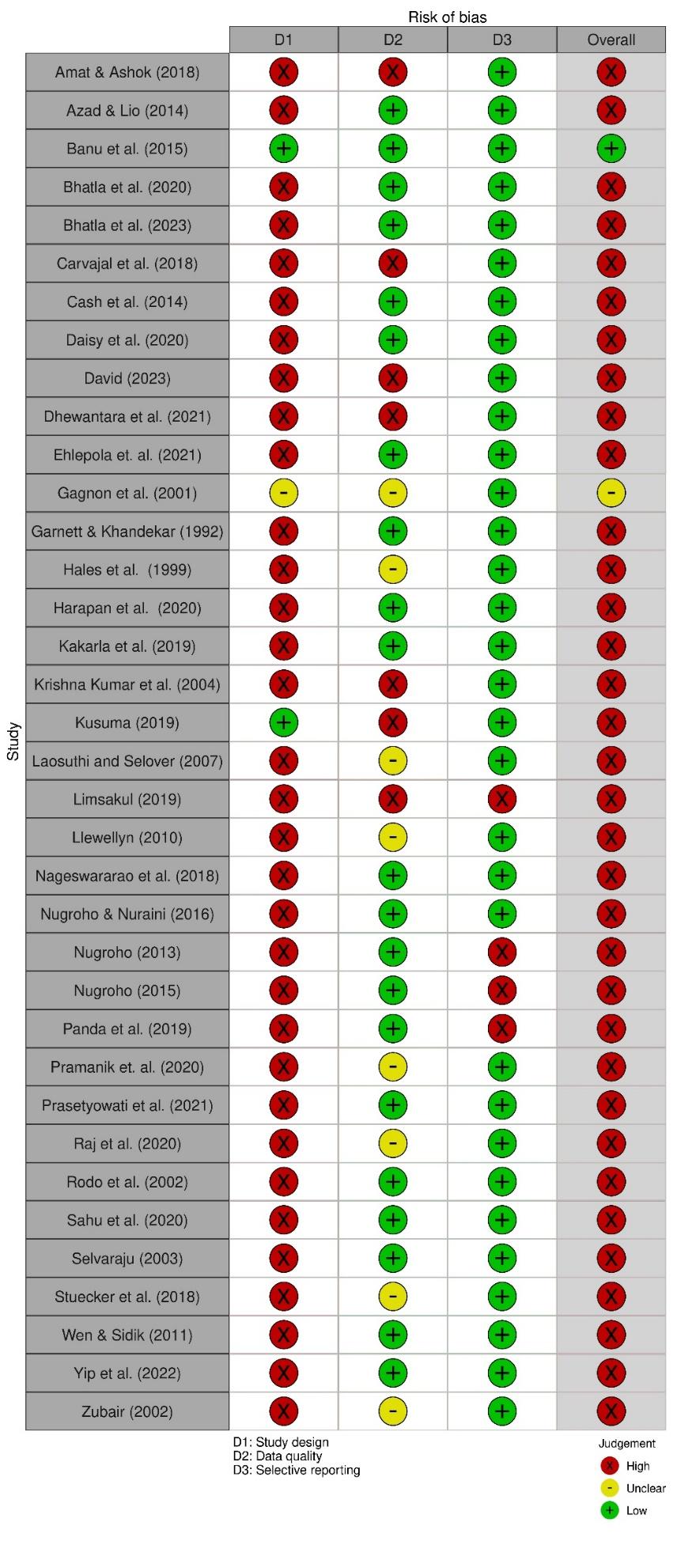


#### **K.3 Other studies**

**Appendix Table 20. Risk of bias assessment for other studies**

**
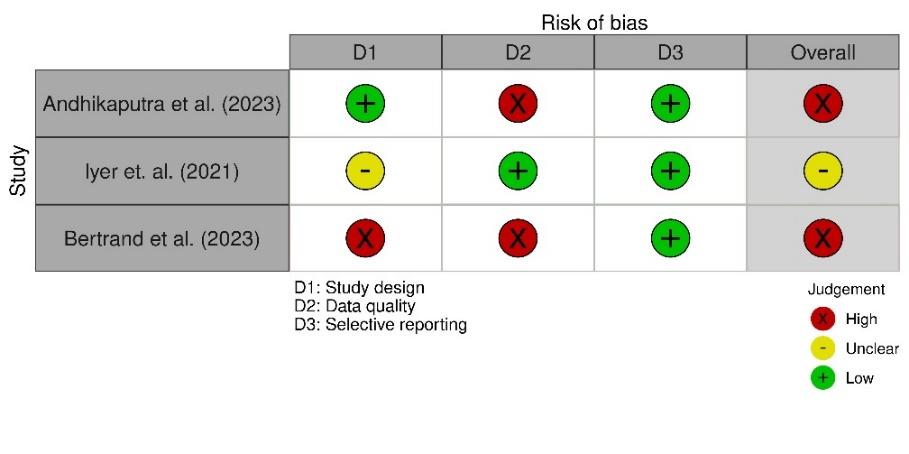
**

*Note: these studies report incidence rate ratios (Andhikapura et al. 2023 and Iyer et al. 2021) or ANOVA significance results (Bertrand et al. 2023).*

#### **K.4 Detailed assessment for each study**

**Appendix Table 21. Study quality table for all criteria**

| Year | Analytical approach | Identification strategy tests | Confounding | Outcome measurement | Data frequency and length | Consistency with unit of analysis and reported results | Selective reporting |
| --- | --- | --- | --- | --- | --- | --- | --- |
| Dait (2022) | Yes | Yes | Yes | Yes | No | Yes | Yes |
| Ferris (1999) | Yes | Yes | No | Yes | No | Yes | Unclear |
| Tipayamongkholgul et al. (2009) | Yes | Yes | Yes | Yes | Yes | Yes | Yes |
| Sharmin et al. (2016) | Yes | Yes | Yes | Yes | Yes | Yes | Yes |
| Datt & Hoogeveen (2003) | Yes | Unclear | Yes | Unclear | N/A | Yes | Yes |
| Duncan (2008) | Yes | Unclear | Yes | Yes | Unclear | Yes | Yes |
| Hashizume et al. (2011) | Yes | Yes | Yes | Yes | Yes | Yes | Yes |
| Ismail & Chan (2020) | Yes | Yes | Yes | Yes | Yes | Yes | Yes |
| Hughes et al. (2010) | Yes | No | No | Yes | Yes | Yes | Yes |
| Falcon et al. (2004) | Yes | No | No | Yes | Yes | Yes | Yes |
| Di Giuseppe et al. (2022) | Yes | Yes | No | Yes | Yes | Yes | Yes |
| Arcari & Tapper (2017) | Yes | Yes | No | Yes | Yes | Yes | Yes |
| Che-Him (2016) | Yes | Yes | Yes | Yes | Yes | Yes | Yes |
| Liyanage et al. (2016) | Yes | Yes | Yes | Yes | No | Yes | Yes |
| Oluwole (2015) | Unclear | No | No | Unclear | No | No | Yes |
| Pipitpukdee et al. (2020) | Yes | Yes | Yes | Yes | No | Yes | Unclear |
| Tawang et al. (2002) | Yes | Unclear | Yes | Yes | No | Yes | Yes |
| Tawang et al. (2003) | Yes | Yes | Yes | Yes | No | Yes | Yes |
| Reyes et al. (2009) | Yes | Yes | Yes | Yes | Yes | Yes | Yes |
| Nguyen et al. (2020) | Yes | Yes | Yes | Yes | Yes | Yes | Yes |
| Cruz & Canlas (2004) | Yes | Yes | Yes | Yes | Unclear | Yes | Yes |
| Zacharia et al. (2020) | Yes | Yes | No | Yes | No | No | Yes |
| Utami et al. (2011) | Yes | Yes | Yes | Yes | Unclear | Unclear | Yes |
| Abdolrahimi (2016) | Yes | No | Yes | Yes | No | Yes | Yes |
| Pipitpukdee et al. (2020) | Yes | Yes | Yes | Yes | No | Yes | Yes |
| Yang (2017) | Yes | Yes | Yes | Yes | Yes | Yes | Yes |
| Roberts et al. (2009) | Yes | No | No | Yes | Yes | Yes | Yes |
| Soria & Preciados (2018) | Yes | Yes | Yes | Yes | Unclear | Yes | Yes |
| Khor et al. (2021) | Yes | Yes | No | Yes | Yes | Yes | Yes |
| Suratno (2022) | Yes | Yes | Yes | Yes | No | Yes | Yes |
| Ahmad et. al. (2019) | Yes | Unclear | Yes | Unclear | No | Unclear | Yes |
| Arcenas (2018) (2018) | Yes | Yes | Yes | Unclear | No | Yes | Yes |
| Ismaya & Anugrah (2018) | Yes | Yes | Yes | Yes | No | Yes | Yes |
| Khoiruddin et al. (2021) | Yes | Yes | Yes | Yes | Yes | Yes | Yes |
| Kien et al. (2020) | Yes | Yes | No | Yes | Yes | No | Yes |
| Mahmudul et al. (2020) | Yes | Yes | Yes | Yes | Yes | Yes | Yes |
| Rahman et al. (2013) | Yes | Yes | Yes | Yes | Yes | Yes | Yes |
| Bekkering (2017) | Yes | Yes | Yes | Unclear | Yes | Yes | Yes |
| Adams et. al. (2022) | No | No | Yes | Yes | Yes | Yes | Yes |
| Impoinvil et. al. (2013) | Yes | Yes | Yes | Yes | Yes | Yes | Yes |
| Lam et al. (2019) | Yes | Yes | No | Yes | Yes | Yes | Yes |
| Cashin et al. (2015) | Yes | Yes | Yes | Yes | Yes | Yes | Yes |
| Cahyaningtyas et al. (2022) | Yes | Yes | Yes | Yes | Yes | Yes | Unclear |
| Kim et al. (2016) | Yes | Yes | Yes | Yes | Yes | Yes | Yes |
| Fajri et al. (2019) | Yes | Yes | Yes | Yes | Unclear | Yes | Yes |
| Siregar et al. (2020) | Yes | Yes | Yes | Yes | No | Yes | Yes |
| Perez-Saez et al. (2016) | Yes | Yes | No | Yes | Yes | Yes | Yes |
| Tiensuwan & O'Brien (2013) | No | No | No | Yes | Yes | Yes | No |
| Yip et al. (2022) | Yes | Yes | No | Yes | Yes | Yes | Yes |
| Banu et al. (2015) | Yes | Yes | Yes | Yes | Yes | Yes | Yes |
| Limsakul (2019) | Yes | No | Yes | Yes | No | Yes | No |
| Kusuma (2019) | Yes | Yes | Yes | Yes | No | Yes | Yes |
| Laosuthi & Selover (2007) | Yes | Yes | No | Yes | Unclear | Yes | Yes |
| Selvaraju (2003) | No | Unclear | No | Yes | Yes | Yes | Yes |
| David (2023) | No | No | No | Yes | No | Yes | Yes |
| Bhatla et al. (2023) | No | No | No | Yes | Yes | Yes | Yes |
| Prasetyowati et al. (2021) | Yes | No | No | Yes | Yes | Yes | Yes |
| Azad & Lio (2014) | Yes | No | No | Yes | Yes | Yes | Yes |
| Dhewantara et al. (2021) | Yes | No | No | Yes | No | Yes | Yes |
| Gagnon et al. (2001) | Yes | Unclear | Unclear | Yes | Unclear | Yes | Yes |
| Cash et al. (2014) | Yes | Yes | No | Yes | Yes | Yes | Yes |
| Daisy et al. (2020) | Yes | No | No | Yes | Yes | Yes | Yes |
| Wen & Sidik (2011) | Yes | No | No | Yes | Yes | Yes | Yes |
| Garnett & Khandekar (1992) | Yes | No | N/A | Yes | Yes | Yes | Yes |
| Nageswararao et al. (2018) | Yes | No | Yes | Yes | Yes | Yes | Yes |
| Carvajal et al. (2018) | Yes | No | No | Yes | No | Yes | Yes |
| Nugroho (2015) | No | No | N/A | Yes | Yes | Yes | No |
| Nugroho & Nuraini (2016) | Yes | No | N/A | Yes | Yes | Yes | Yes |
| Panda et al. (2019) | No | No | N/A | Yes | Yes | No | No |
| Nugroho (2013) | No | No | No | Yes | Yes | Yes | No |
| Krishna Kumar et al. (2004) | Yes | No | No | Yes | No | Yes | Yes |
| Hales et al. (1999) | Yes | No | No | Unclear | Yes | Yes | Yes |
| Harapan et al. (2020) | Yes | Yes | No | Yes | Yes | Yes | Yes |
| Kakarla et al. (2019) | Yes | Unclear | No | Yes | Yes | Yes | Yes |
| Pramanik et. al. (2020) | Yes | Unclear | No | Yes | Unclear | Yes | Yes |
| Sahu et al. (2020) | Yes | Unclear | No | Yes | Yes | Yes | Yes |
| Amat & Ashok (2018) | No | No | No | Yes | No | Yes | Yes |
| Bhatla et al. (2020) | No | No | No | Yes | Yes | Yes | Yes |
| Zubair (2002) | Yes | No | N/A | Unclear | Yes | Yes | Yes |
| Raj et al. (2020) | Yes | Yes | No | Yes | Unclear | Yes | Yes |
| Stuecker et al. (2018) | Yes | No | N/A | Yes | Unclear | Yes | Yes |
| Ehlepola et. al. (2021) | Yes | Yes | No | Yes | Yes | Yes | Yes |
| Rodo´ et al. (2002) | Yes | No | N/A | Yes | Yes | Yes | Yes |
| Llewellyn (2010) | Yes | No | No | Yes | Unclear | Yes | Yes |
| Andhikaputra et al. (2023) | Yes | Yes | Yes | Yes | No | Yes | Yes |
| Ghose et al. (2021) | Yes | Yes | No | Yes | Yes | Yes | No |
| Iyer et. al. (2021) | Yes | Unclear | Yes | Yes | Yes | Yes | Yes |
| Rao et al. (2012) | Unclear | Unclear | Yes | Yes | No | Yes | Yes |
| Bertrand et al. (2023) | No | No | No | Unclear | No | Yes | Yes |

## Appendix L Box plots presenting the distribution of the correlation coefficients for India and Indonesia

For two countries with the largest amount of correlational evidence, India and Indonesia, the box plots below showcase the distribution of correlation coefficient medians for each outcome. The box area spans from the first to the third quartile and represents the spread of the central half of the coefficients. The larger the box, the more spread the middle 50% of the coefficients are. The lines that extend from the edges of the box are called whiskers and indicate the range of the data, going as far as one and a half times the length of the box. The line inside the box indicates the median correlation coefficient or midpoint where 50% of the data values are below it and the other 50% above it. The “x” shows the mean of the coefficients. We decided to use the median in the analysis of the effects of El Niño and +IOD instead of the mean as the median is not affected by outliers and its signs roughly indicates whether most of the coefficients in included studies are above or below zero, suggesting either a positive of negative relation being reported more often. For outcomes where we identified only one estimate, the figure shows a line for that estimate.

**Figure A.12. 1. Distribution of coefficients for studies measuring the correlation between El Niño and economics and health outcomes in India**


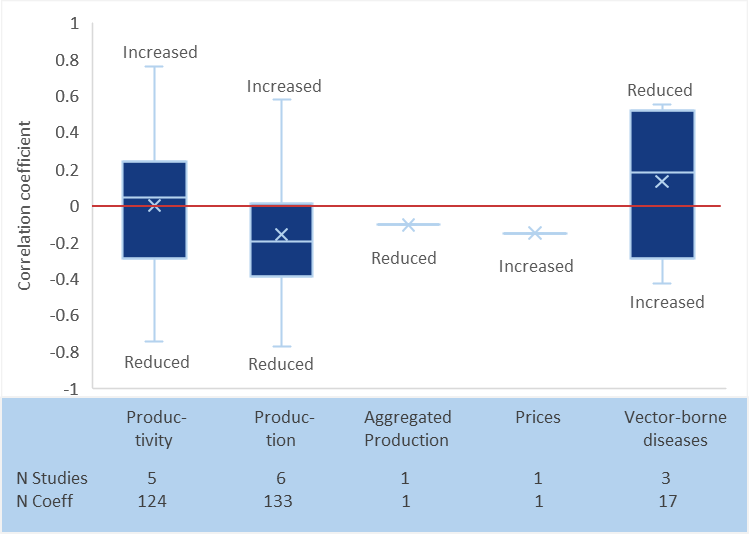


*Source: 3ie (2025)*

**Figure A.12. 2. Distribution of coefficients for studies measuring the correlation between +IOD on economic and health outcomes in India**


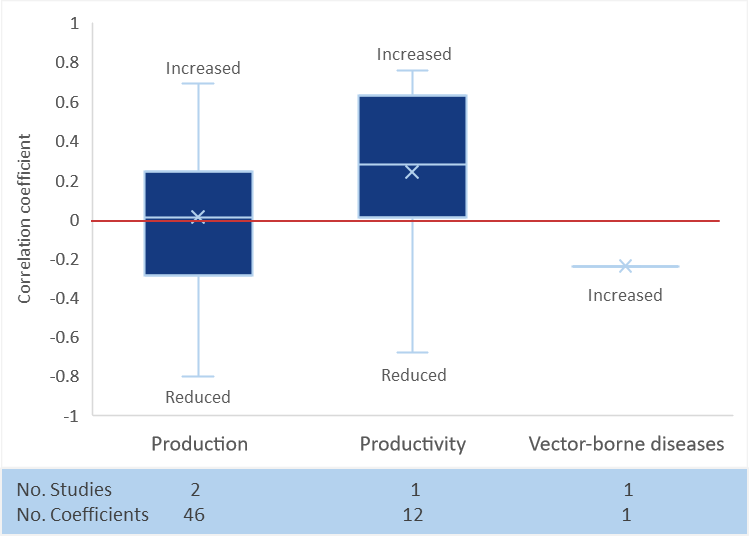


*Source: 3ie (2025)*

**Figure A.12. 3. Distribution of coefficients for studies measuring the correlation between El Niño and health and economic outcomes for Indonesia**


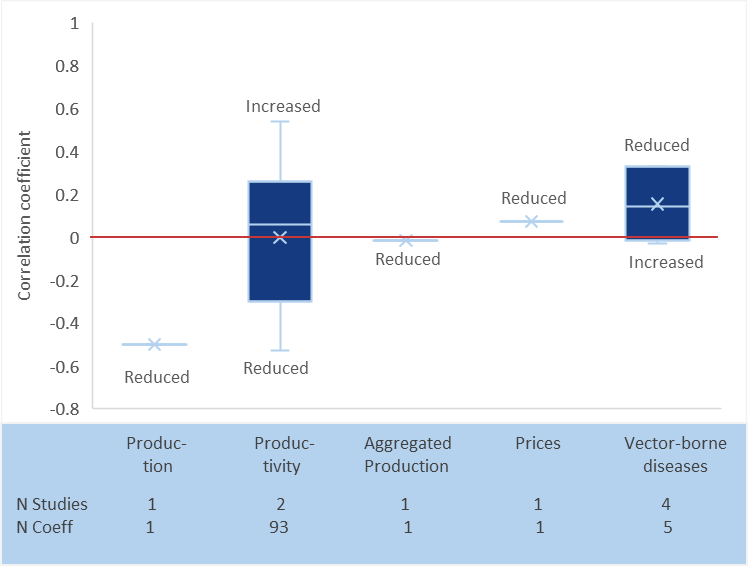


*Source: 3ie (2025)*

**Figure A.12. 4. Distribution of coefficients for studies measuring the correlation between +IOD and health outcomes for Indonesia**


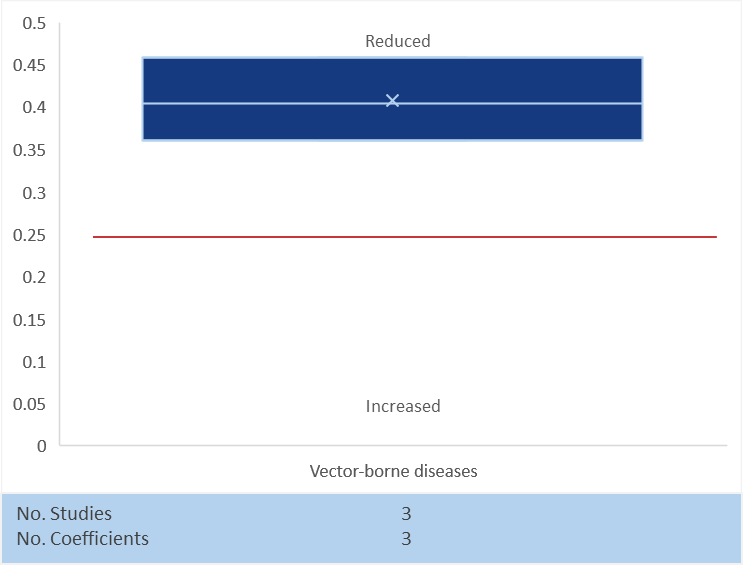


*Source: 3ie (2025)*

## Appendix M Potential biases in the review process

While efforts were made to mitigate biases, such as inclusion of both published and un-published literature, independent selection of studies by two reviewers, data-coding in duplicate, and risk of bias assessment of included studies, several potential sources of bias merit attention.

Firstly, the search strategy, while not constrained by language, was developed and executed exclusively in English. Consequently, there exists a risk of language bias, potentially limiting the inclusion of relevant literature published in languages other than English. This could result in a biased representation of evidence, favouring academic publications produced outside of the region of interest, which may not fully account for contextual factors.

Additionally, we report the risk of bias of all included studies and acknowledge the limitations identified in the literature throughout our analysis but were unable to present the results from low-risk of bias studies separately as we did not have enough data to do so.

Synthesizing the evidence from studies using a varied ray of analytical designs imposes some inherent challenges as estimates are not always comparable. For this reason, in a meta-analysis framework, estimates are converted to standard measures that can be pooled together. Regression studies were subjected to such techniques and used in meta-analysis. However, due to the nature of correlation studies and the inability to convert their results into effect sizes comparable to those from studies using regression methods, an analysis of medians was utilized for the synthesis of correlation coefficients. Narrative analysis is based on the direction and magnitude of estimates, trying to highlight important differences in results.

## Appendix N Strengths and limitations

Among the strengths of this systematic review, we benefited from a robust search methodology guided by expert input, encompassing both academic and grey literature sources. This comprehensive approach ensured thorough coverage of relevant studies that met our inclusion criteria by providing evidence of an association of either El Niño or +IOD and one of the outcomes of interest in the Indo-Pacific region. Further, any limitation in our academic and grey literature searches should have been mitigated by citation tracking as long as missed records were referenced by one of the included studies (or cited one of them) and scored by the machine learning classifier as the top 40% most relevant records (by comparing their title and abstract to those of other included studies). Systematic screening, selection and coding procedures were implemented, with independent reviewers conducting each stage in duplicate. This rigorous process enhanced the reliability and validity of the review's findings.

The integration of machine learning techniques in study selection streamlined the workload associated with title and abstract screening, optimizing efficiency without compromising rigor.

Despite the thorough search strategy, however, the review revealed a gap in the literature regarding the cascading effects of El Niño and +IOD. The limited evidence available hindered the derivation of definitive conclusions regarding the relationship between these climate drivers and the outcomes of interest.

Further, because of the short timeframe of the project, we were not able to contact authors of included studies or potentially includable ones to request missing data. For instance, studies lacking a quantitative estimate of the association between El Niño or +IOD and the outcomes of interest were excluded as we focussed only on quantitative measures that could be combined with statistical analysis. This criterion led to the exclusion of studies presenting results solely through visual means, such as spatial analyses used to explore the geographic variation in the effects of El Niño and +IOD. By doing so, we might have lost the nuance of spatial analysis. A list of excluded studies with concrete examples for each exclusion reason can be found in Appendix A.3.

Finally, a few included studies reporting regression coefficients missed other data needed to compute comparable effect sizes such as standard errors, t-statistics, p-values, or confidence intervals. Consequently, these results could not be incorporated into the meta-analysis and were analysed descriptively instead.
